# A multicentric, single arm, prospective, stratified clinical investigation to evaluate MammoWave’s ability in breast lesions detection

**DOI:** 10.1371/journal.pone.0288312

**Published:** 2023-07-14

**Authors:** Daniel Álvarez Sánchez-Bayuela, Navid Ghavami, Gianluigi Tiberi, Lorenzo Sani, Alessandro Vispa, Alessandra Bigotti, Giovanni Raspa, Mario Badia, Lorenzo Papini, Mohammad Ghavami, Cristina Romero Castellano, Daniela Bernardi, Massimo Calabrese, Alberto Stefano Tagliafico

**Affiliations:** 1 UBT—Umbria Bioengineering Technologies, Perugia, Italy; 2 Breast Imaging Department, Radiology Service, Complejo Hospitalario Universitario de Toledo, Spain; 3 Faculty of Chemical Science and Technology, Instituto Regional de Investigación Científica Aplicada, University of Castilla, La Mancha, Spain; 4 School of Engineering, London South Bank University, London, United Kingdom; 5 IRCCS Humanitas Research Hospital, Rozzano, Milan, Italy; 6 Humanitas University, Milan, Italy; 7 IRCCS Ospedale Policlinico San Martino, Genoa, Italy; 8 University of Genoa, Genoa, Italy; Tsinghua University, CHINA

## Abstract

Microwave imaging is a safe and promising new technology in breast radiology, avoiding discomfort of breast compression and usage of ionizing radiation. This paper presents the first prospective microwave breast imaging study during which both symptomatic and asymptomatic subjects were recruited. Specifically, a prospective multicentre international clinical trial was performed in 2020–2021, to investigate the capability of a microwave imaging device (MammoWave) in allowing distinction between breasts with no radiological finding (NF) and breasts with radiological findings (WF), i.e., with benign or malignant lesions. Each breast scan was performed with the volunteers lying on a dedicated examination table in a comfortable prone position. MammoWave output was compared to reference standard (i.e., radiologic study obtained within the last month and integrated with histological one if available and deemed necessary by responsible investigator) to classify breasts into NF/WF categories. MammoWave output consists of a selection of microwave images’ features (determined prior to trials’ start), which allow distinction between NF and WF breasts (using statistical significance p<0.05). 353 women were enrolled in the study (mean age 51 years ± 12 [SD], minimum age 19, maximum age 78); MammoWave data from the first 15 women of each site, all with NF breasts, were used for calibration. Following central assessor evaluation, 111 NF (48 dense) and 272 WF (136 dense) breasts were used for comparison with MammoWave output. 272 WF comprised 182 benign findings and 90 malignant histology-confirmed cancer. A sensitivity of 82.3% was achieved (95%CI: 0.78–0.87); sensitivity is maintained when limiting the investigation to histology-confirmed breasts cancer only (90 histology-confirmed breasts cancer have been included in this analysis, having sizes ranging from 3 mm to 60 mm). Specificity value of approximately 50% was achieved as expected, since thresholds were calculated (for each feature) using median value obtained after recruiting the first 15 women (of each site), all NF. This prospective trial may represent another step for introducing microwave imaging into clinical practice, for helping in breast lesion identification in asymptomatic women.

## Introduction

Current screening programs for early breast cancer (BC) detection have been a topic of worldwide discussion due to some drawbacks of the current gold standard technique, mammography [1–3]. It is widely known that mammography, and its latest developments (Digital Breast Tomosynthesis, DBT [4]), limits its use in population-based screening programs to both a very specific age range (usually 50 to 69 years old women), and a limited screening frequency, usually biennial [2]. Meanwhile, newer studies estimate that BC is diagnosed in 6.6% of women younger than 40 [5], and more than 20% of BC cases in Europe occur in women when they are below the age of 50 [6]. Usage of ionizing X-rays and its cumulative effect on women has led to many controversies, particularly when dealing with overdiagnosis. Moreover, in some cases lesions prove difficult to detect from mammography, especially when the breast is either highly dense [7–9] or comprises small, elongated salt-like microcalcification particles [10]. In addition, the discomfort caused by breast compression and the performance reduction in dense breasts have motivated many researchers to investigate novel safe techniques that overcome the mentioned limitations [11]. Consequently, microwave imaging has emerged as an interesting potential alternative to ionizing-based techniques [12,13], based on its ability to discriminate between healthy tissues and tissues with lesions, through the existing contrast in dielectric properties (permittivity and conductivity) within microwave frequency spectrum (1–10 GHz).

One of these microwave systems, named MammoWave (UBT Srl, Italy), functions in air with only 2 azimuthally rotating antennas (one transmitting and one receiving), operating within the frequency band 1–9 GHz [14].

This paper describes the first prospective microwave breast imaging clinical trial (multicentric and international), during which both symptomatic and asymptomatic women were recruited. In more detail, the primary objective of this clinical trial was to evaluate the ability of MammoWave in breast lesions detection (where a lesion may be benign or malignant). MammoWave microwave imaging was performed on subjects who have already gone through conventional exams’ radiologist review, to be used as reference standard. For each breast of each subject undergoing this trial, our algorithm based on Huygens’ principle was used to create a set of conductivity weighted microwave images, by varying the conductivity values in the algorithm. Next, several microwave image parameters, i.e., features were calculated and used to measure and quantify the images’ non-homogenous behaviour. A selection of these features permits distinction between breasts with no radiological finding (NF) and those with radiological findings (WF), i.e., with benign or malignant lesions. The results of this prospective multicentre international clinical trial study are presented and discussed.

## Materials and methods

### MammoWave device and imaging algorithm

**Fig 1:**
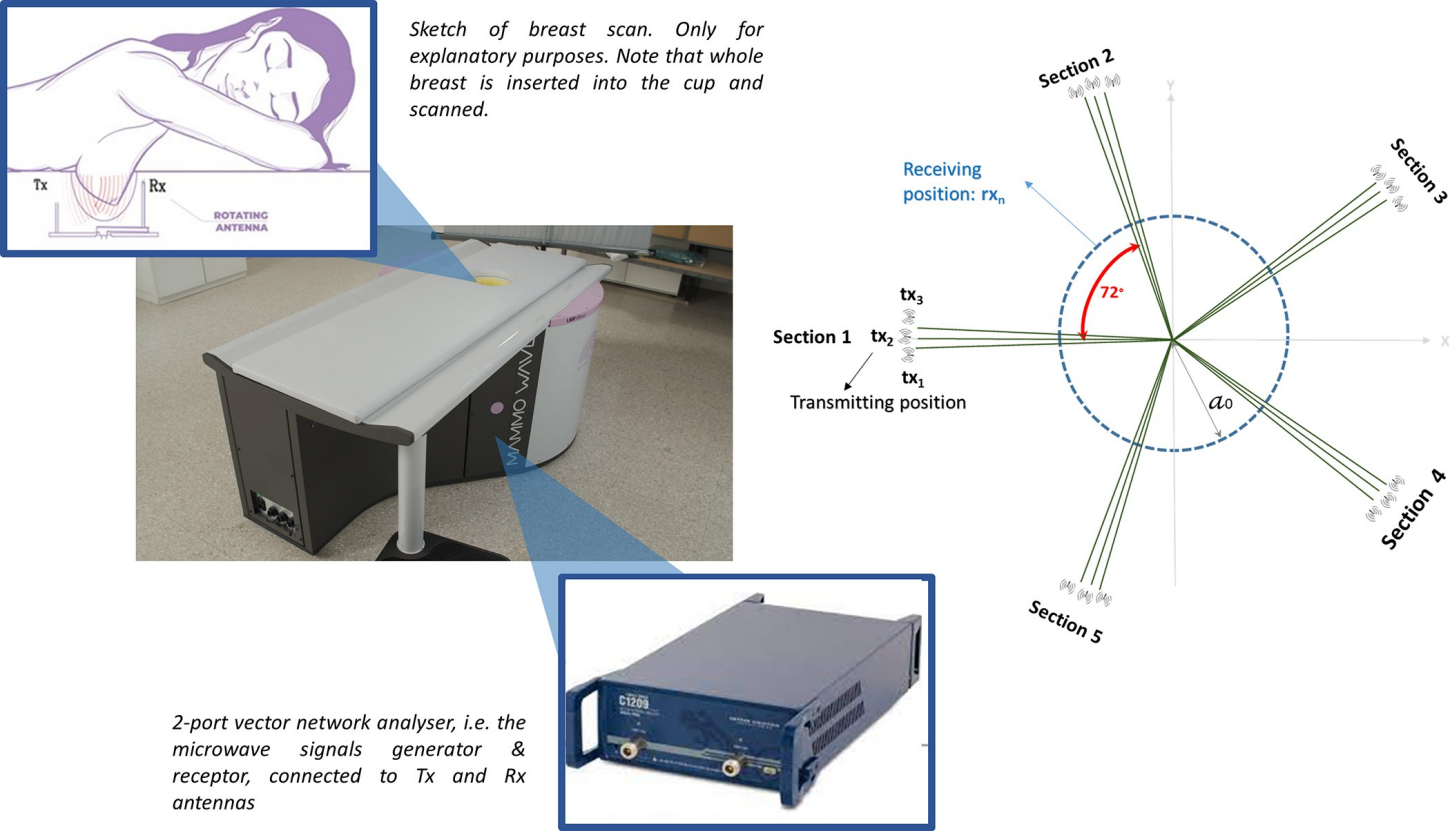
MammoWave system, sketch of the breast imaging configuration showing the cylindrical hub and the antennas (left). Transmitting and receiving antenna configuration, showing the five triplet sections (right).

MammoWave device’s configurations can be seen in Fig 1. It contains two antennas, one transmitting and one receiving the microwave signals. Both antennas operate in air (without any matching liquid/medium), within frequency range 1–9 GHz, and are positioned on the same height. Antennas are connected to a 2-port vector network analyzer (Copper Mountain Technologies, Indianapolis, IN) and are contained by a cylindrical hub internally surrounded by microwave absorbers. This hub includes a cup placed inside a hole, which permits insertion of the patient’s whole breast in a prone position. Both antennas rotate azimuthally, collecting the signals multi-bistatically in frequency domain. For each transmitting and receiving position, the complex S21 is collected from 1 to 9 GHz, with 5 MHz sampling. MammoWave acquisition time is approximately 10 minutes (per breast). To process the received signals, we use previously developed Huygens principle-based imaging algorithm [14–19].

### Clinical protocol

Three hospitals (2 in Italy and 1 in Spain) took part in this study, after obtaining the approvals from the correspondent Ethical Committees (ID: 2558; ID: 262/2019; ID: 440). The study was carried out in accordance with the protocol and principles of Declaration of Helsinki and the guidelines of Good Clinical Practice issued by ICH. All subjects included in the study consented to participate.

This prospective multicentre international clinical trial was activated in 2020 (ClinicalTrials.gov Identifier: NCT04253366) with the aim of quantifying MammoWave’s capability in distinguishing between breasts without (NF) and with radiological findings (WF), i.e., with breast lesions (BL) which may be benign or malignant.

This protocol’s primary objective is to generate empirical evidence for detection of WF breasts using MammoWave and evaluating its sensitivity against reference standard. Reference standard (i.e., radiologic study obtained within the last month and integrated with histological one if deemed necessary by responsible investigator and when available), is used to classify the breasts into two categories: NF and WF. NF and WF breasts can be defined using the American College of Radiology recommendations via their Breast Imaging Reporting And Data System (BI-RADS): breasts are classified as NF when their traditional radiologic assessment is BI-RADS 1; breasts are classified as WF when their final BI-RADS assessments are: either 2 for benign findings, 3 for follow-up findings or 6 for confirmed carcinoma (intermediate BI-RADS assessment, i.e. 4 and 5, is not included in this analysis as suspected findings in these breasts were eventually confirmed by additional imaging and/or histology).

Secondary objectives are:

- MammoWave’s specificity in NF breast detection;
- Percentage of correct BC detection;
- MammoWave’s sensitivity among different breast densities;
- Volunteers’ satisfaction.

**Fig 2:**
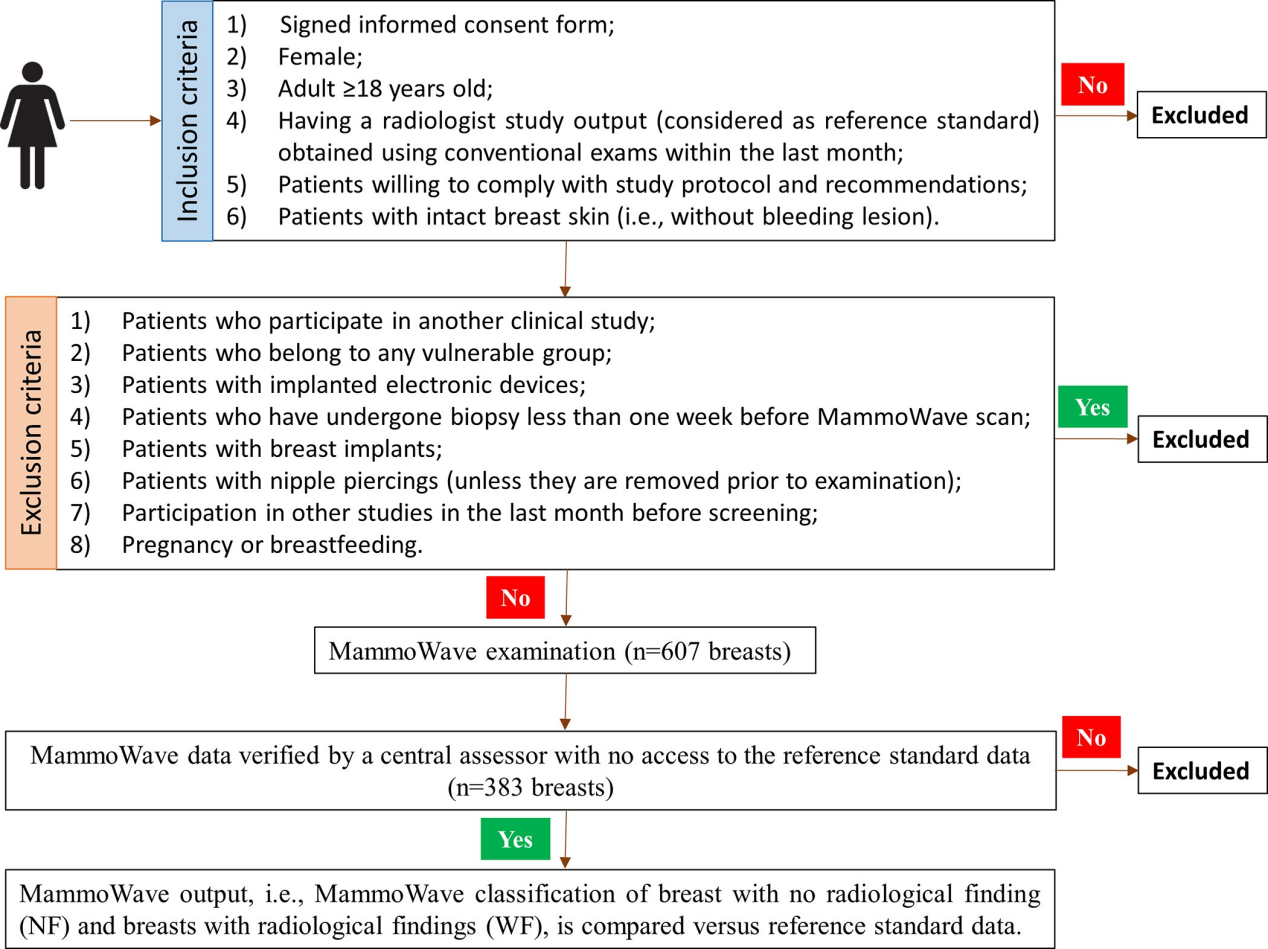
Clinical trial’s workflow of the second phase. Prior to the kick-off of the second phase, 15 participants in each centre, all having NF breasts, have been examined by MammoWave and data has been used to calibrate the image parameters’ thresholds. MammoWave images were reviewed by a central assessor (an independent external scientist), who had no access to the reference standard data; the central assessor discarded MammoWave outputs due to the presence of spurious peaks in MammoWave images. Subsequently, the microwave imaging output of MammoWave is compared to the output of the radiologist study review (from conventional exams).

The study includes two phases: in the *first phase*, 15 participants in each centre all having NF breasts are examined by MammoWave. These data are used to calibrate the image parameters’ thresholds for each apparatus. In the *second phase*, remaining participants are enrolled and examined by MammoWave, and results are compared in a prospective manner against reference standard as described in Fig 2.

WF breasts include both malignant and benign lesions. Lesions (including BC) may be palpable or non-palpable; lesions also include isolated clustered microcalcifications.

### MammoWave analysis

MammoWave acquisition is made just once, producing six conductivity-weighted microwave images, i.e., *methods*, for each breast, using conductivity values ranging from 0.3 to 0.8 S/m; such *methods* have been selected via a feasibility study [19].

Images obtained using MammoWave are intensity maps given in linear arbitrary units, representing the homogeneity of tissues’ dielectric properties. Images are maximum intensity projection coronal 2D maps of the entire breast volume. To allow inter and intra-subject comparison, all images are normalized to unitary average of the intensity.

For allowing a quantification of images’ non-homogenous behaviour, we introduced and selected dedicated parameters, i.e., *features*, detailed in the Appendix and in [19].

For each breast and for all selected *methods* and *features*, we introduce a binary score S defined as:

$$\{\begin{array}{l} \mathrm{if}\;\mathrm{feature}>D_{\mathrm{offset}|\mathrm{feature}}\;\mathrm{then}\;S=1\\ \mathrm{if}\;\mathrm{feature}\leq D_{\mathrm{offset}|\mathrm{feature}}\;\mathrm{then}\;S=0 \end{array}$$

The threshold *D*_*offset|feature*_ is calculated for each feature, using median value obtained in each site after recruiting first 15 subjects, all having breasts without lesion.

The binary score S is then used for establishing an empirical *rule-of-thumb* allowing assessment of MammoWave images between “WF Breast, i.e., with lesion (positive)” or “NF Breast, i.e., with no lesion (negative)”. More details are provided in the Appendix.

### Statistical analysis

According to sample size calculation, we enrol both subjects with WF breasts and NF breasts, with WF prevalence of ~70%. The required overall detection ability is 70% (for all breast densities). A minimum of 250 subjects (175 WF) is required to verify a sensitivity of 70% (H1) versus H0 = 60%, with an error of first type α = 0.05 and a power (1-β) = 80%. For all selected *features* of all *methods*, we apply Welch’s t-test (two-sample two-tailed unpooled variances t-test) with α = 0.05 to verify the statistical significance (p<0.05) using the reference standard, and we evaluate the receiver operating characteristic (ROC), calculating the area under the curve (AUC). Specifically, multiple positivity thresholds were applied to derive the ROC and AUC for the different *features*.

Performances of MammoWave’s proposed rule-of-thumb are evaluated calculating the true-positive (TP) rate, i.e., sensitivity, and true-negative (TN) rate, i.e., specificity, comparing MammoWave against reference standard (excluding first 15 subjects of each site).

## Results

From October 2020 to August 2021, 353 subjects were enrolled in the study (mean age 51 years ± 12 [SD], minimum age 19, maximum age 78), of which 180 were enrolled in hospital 1 (University Hospital of Toledo, former Hospital Virgen de la Salud, Spain), 110 in hospital 2 (Ospedale Policlinico San Martino, Genoa, Italy), and 63 in hospital 3 (Humanitas Research Hospital, Rozzano, Milan, Italy).

First 15 subjects of each site, all with NF breasts, were used for calculating the threshold *D*_*offset|feature*_ for each feature. In the second phase, MammoWave examination was done on 308 subjects (one subject refused due to discomfort), resulting in a total of 607 analyzed breasts. 299 subjects’ MammoWave examination was conducted on both breasts, while for 9 subjects it was only done on one breast (2 left, 7 right).

No protocol deviations were registered in inclusion/exclusion criteria.

For obtaining the reference standard, Mammography was used on 244 subjects (79.2%), ultrasound on 250 (81.2%), MRI on 71 (23.1%), and histology on 166 (53.9%).

According to the radiologist study review, 230 NF (93 dense) and 377 WF (197 dense) breasts were analyzed in the second phase. Lesions’ final assessment led to a total of 260 benign findings and 117 malignant histology-confirmed cancer. Benign findings included simple cysts, duct ectasia conditions, nodules of solid fibroadenoma, benign microcalcifications deposits, glandular asymmetries, and mammographic architectural distortions confirmed to be radial scars, sclerosing lesions, adenosis or fat necrosis. Malignant confirmed cancer included common malignant conditions (invasive lobular carcinoma, invasive ductal carcinoma and ductal carcinoma in situ) via nodules with or without associated microcalcifications, and mammographic architectural distortions. Summary of radiological study review is given in Table 1.

**Table 1:** Summary of radiological study review.

Summary of the radiological study review for the 607 breasts. Number of dense breasts indicated inside brackets.				
	**All breasts**	**NF Breasts**	**WF Breasts (including histology-confirmed cancer)**	**Breasts with histology-confirmed cancer**
***Hospital 1***	322 (157)	95 (34)	227 (123)	62 (23)
***Hospital 2***	187 (75)	82 (25)	105 (50)	41(14)
***Hospital 3***	98 (58)	53 (34)	45 (24)	20 (6)
**TOTAL**	607 (290)	230 (93)	377 (197)	123 (43)
Summary of the radiological study review of the cases included for comparison with MammoWave, i.e., 383 breasts. Number of dense breasts indicated inside brackets.				
	**All breasts**	**NF Breasts**	**WF Breasts (including histology-confirmed cancer)**	**Breasts with histology-confirmed cancer**
***Hospital 1***	217 (103)	48 (15)	169 (88)	48 (18)
***Hospital 2***	101 (40)	35 (11)	66 (29)	26 (9)
***Hospital 3***	65 (41)	28 (22)	37 (19)	16 (5)
**TOTAL**	383 (184)	111 (48)	272 (136)	90 (32)

Following the MammoWave exam, data was transferred for analysis; then MammoWave images and outputs (based on the *rule-of-thumb* described above) were sent back to the centre (all steps were performed automatically).

Subsequently, MammoWave images were reviewed by a central assessor (an independent external scientist), who had no access to the reference standard data. The central assessor discarded 225 MammoWave outputs due to the presence of spurious peaks in MammoWave images (i.e., a peak which is out of scale as defined in our previous clinical trials [19]). After such discard, we generated a new reference standard dataset (provided in Table 1 as well), from which: 111 NF (48 dense) and 272 WF (136 dense) breasts were used for comparison with MammoWave output. The 272 WF comprised 182 benign findings and 90 malignant histology-confirmed cancer.

Considering this new reference standard dataset, Table 2 summarizes the radiologist study review by finding typology and includes a summary of radiologists’ final BI-RADS assessment. Final BI-RADS assessment for each breast was given via ACR standard methodology: BI-RADS 1 for breasts with no radiological findings, BI-RADS 2 for breasts with clearly benign findings, BI-RADS 3 for breasts under follow-up, and BI-RADS 6 for histology-confirmed breasts with cancer. Concerning the size of the histology-confirmed breasts cancer, different findings (nodules, architectural distortions, microcalcifications, etc.) were assessed and characterized using conventional imaging leading to cancer sizes ranging from 3 mm to 60 mm.

**Table 2:** Radiologist study review’s finding typology and final BI-RADS assessment.

Summary of the radiological study review by mammographic and/or ultrasonographic finding typology (WF) of the cases included for comparison with MammoWave. Number of dense breasts indicated inside brackets.				
	**Hospital 1**	**Hospital 2**	**Hospital 3**	**TOTAL**
**Simple cysts and/or dispersed duct ectasia**	92 (51)	26 (16)	11 (8)	129 (75)
**Nodules of solid fibroadenoma**	16 (12)	6 (3)	8 (5)	30 (20)
**Dispersed/group microcalcifications**	8 (4)	9 (4)	3 (3)	20 (11)
**Spiculated nodules with/without associated microcalcifications**	43 (14)	16(5)	14 (3)	73 (22)
**Architectural distortions**	10 (7)	9 (1)	1 (0)	20 (8)
**TOTAL**	169 (88)	66 (29)	37 (19)	272 (136)
Summary of the radiologists’ final BI-RADS assessment considering mammography and/or ultrasonography and/or MRI and/or pathology output of breasts included in the study for comparison with MammoWave. Number of dense breasts indicated inside brackets.				
**BI-RADS 1**	48 (15)	35 (11)	28 (22)	111 (48)
**BI-RADS 2**	102 (55)	37 (20)	16 (10)	155 (85)
**BI-RADS 3**	19 (15)	3 (0)	5 (4)	27 (19)
**BI-RADS 6**	48 (18)	26 (9)	16 (5)	90 (32)
**TOTAL**	217 (103)	101 (40)	65 (41)	383 (184)

For all selected features, Table 3 lists the mean and standard deviation for the NF breasts, the mean and standard deviation for the WF breasts, Welch’s t-test score and p-value and the AUC.

**Table 3:** Calculated mean, standard deviation, Welch’s t-test score and p-value and the AUC for all the selected features. In the left column, we indicate the names of the features preceded by the names of the corresponding methods (where m1 indicates method1, m2 indicates method2, and so on).

	Mean (NF)	Std (NF)	Mean (WF)	Std (WF)	Welch’s t-test score	p-value	AUC
**m1_M2MEA_i**	2.08426	0.25486	2.21252	0.30536	1	<0.001	0.62 (95%CI: 0.56–0.68)
**m1_MAX_n**	2.08426	0.25486	2.21252	0.30536	1	<0.001	0.62 (95%CI: 0.56–0.68)
**m1_VAR_p**	0.14516	0.05658	0.17454	0.07173	1	<0.001	0.63 (95%CI: 0.57–0.69)
**m1_MAD0_p**	0.30042	0.06142	0.33132	0.07262	1	<0.001	0.63 (95%CI: 0.57–0.69)
**m1_VAR_r**	1.02773	0.38186	1.23916	0.54992	1	<0.001	0.61 (95%CI: 0.55–0.67)
**m2_M2MEA_i**	2.20549	0.28801	2.3523	0.34609	1	<0.001	0.62 (95%CI: 0.56–0.68)
**m2_MAX_n**	2.20549	0.28801	2.35238	0.34609	1	<0.001	0.62 (95%CI: 0.56–0.68)
**m2_VAR_p**	0.20317	0.06655	0.24043	0.08801	1	<0.001	0.63 (95%CI: 0.57–0.69)
**m2_MAD0_p**	0.35890	0.06099	0.39130	0.07493	1	<0.001	0.63 (95%CI: 0.57–0.69)
**m2_MAD0_r**	1.10997	0.19663	1.21062	0.25617	1	<0.001	0.62 (95%CI: 0.56–0.68)
**m3_ROS1_i**	2.38319	0.32300	2.54882	0.38860	1	<0.001	0.63 (95%CI: 0.57–0.69)
**m3_VAR_p**	0.25786	0.07930	0.30232	0.10500	1	<0.001	0.63 (95%CI: 0.57–0.69)
**m3_MAD0_p**	0.40751	0.06431	0.44143	0.07858	1	<0.001	0.63 (95%CI: 0.57–0.69)
**m3_MAD1_p**	0.34115	0.06879	0.37529	0.07926	1	<0.001	0.63 (95%CI: 0.57–0.69)
**m3_MAD0_r**	1.15997	0.19995	1.26127	0.26004	1	<0.001	0.61 (95%CI: 0.55–0.67)
**m4_M2MEA_i**	2.4254	0.33599	2.59749	0.40618	1	<0.001	0.64 (95%CI: 0.58–0.70)
**m4_ROS1_i**	2.47491	0.34319	2.64978	0.41440	1	<0.001	0.63 (95%CI: 0.57–0.69)
**m4_MAX_n**	2.4254	0.33599	2.59749	0.40618	1	<0.001	0.64 (95%CI: 0.58–0.70)
**m4_MAX_p**	2.4254	0.33599	2.59749	0.40618	1	<0.001	0.64 (95%CI: 0.58–0.70)
**m4_VAR_p**	0.31089	0.09175	0.36050	0.12186	1	<0.001	0.63 (95%CI: 0.57–0.69)
**m5_M2MEA_i**	2.61185	0.37597	2.80239	0.45161	1	<0.001	0.63 (95%CI: 0.57–0.69)
**m5_ROS1_i**	2.64289	0.38075	2.8352	0.45666	1	<0.001	0.63 (95%CI: 0.57–0.69)
**m5_ROS2_i**	2.77801	0.58382	3.05578	0.71240	1	<0.001	0.62 (95%CI: 0.56–0.68)
**m5_MAX_n**	2.61185	0.37597	2.80239	0.45161	1	<0.001	0.63 (95%CI: 0.57–0.69)
**m5_MAX_p**	2.61185	0.37597	2.80239	0.45161	1	<0.001	0.63 (95%CI: 0.57–0.69)
**m6_M2MEA_i**	2.18873	0.24168	2.23478	0.23678	1	0.037	0.55 (95%CI: 0.49–0.61)
**m6_ROS1_i**	2.23394	0.24719	2.28054	0.24292	1	0.039	0.55 (95%CI: 0.49–0.61)
**m6_MIN_n**	0.03716	0.00602	0.03608	0.00547	0	0.124	0.54 (95%CI: 0.48–0.60)
**m6_MAX_n**	2.18873	0.24168	2.23478	0.23678	1	0.037	0.55 (95%CI: 0.49–0.61)
**m6_MAX_p**	2.18873	0.24168	2.23478	0.23678	1	0.037	0.55 (95%CI: 0.49–0.61)

**Fig 3:**
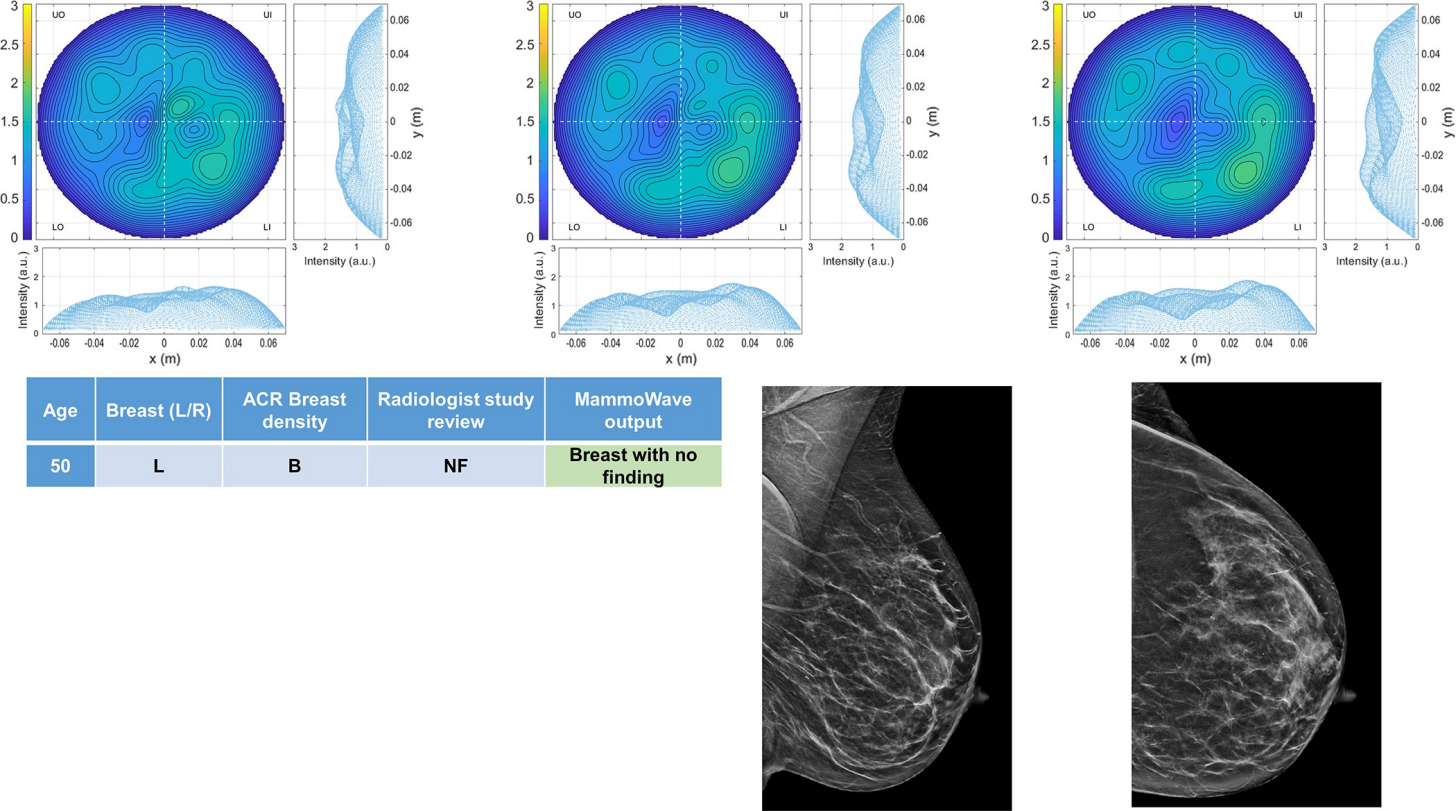
Example of NF breast: 50 years old woman, mammographic low-density (ACR B), left breast (DBT and ultrasonography BI-RADS 1). Microwave images, normalized to unitary average of the intensity, are given in the top row for three different conductivity weightings (from left to right: 0.3 S/m, 0.4 S/m and 0.5 S/m, respectively). Microwave images are given here as 2D images in the azimuthal, i.e., coronal plane. Moreover, 1D intensity projection on X and Y is displayed in the inserts. X and Y are given in meters; intensity is in arbitrary units. The proposed rule-of-thumb classifies this breast as negative (the values of the microwave images’ selected features are provided in the S1B Table).

**Fig 4:**
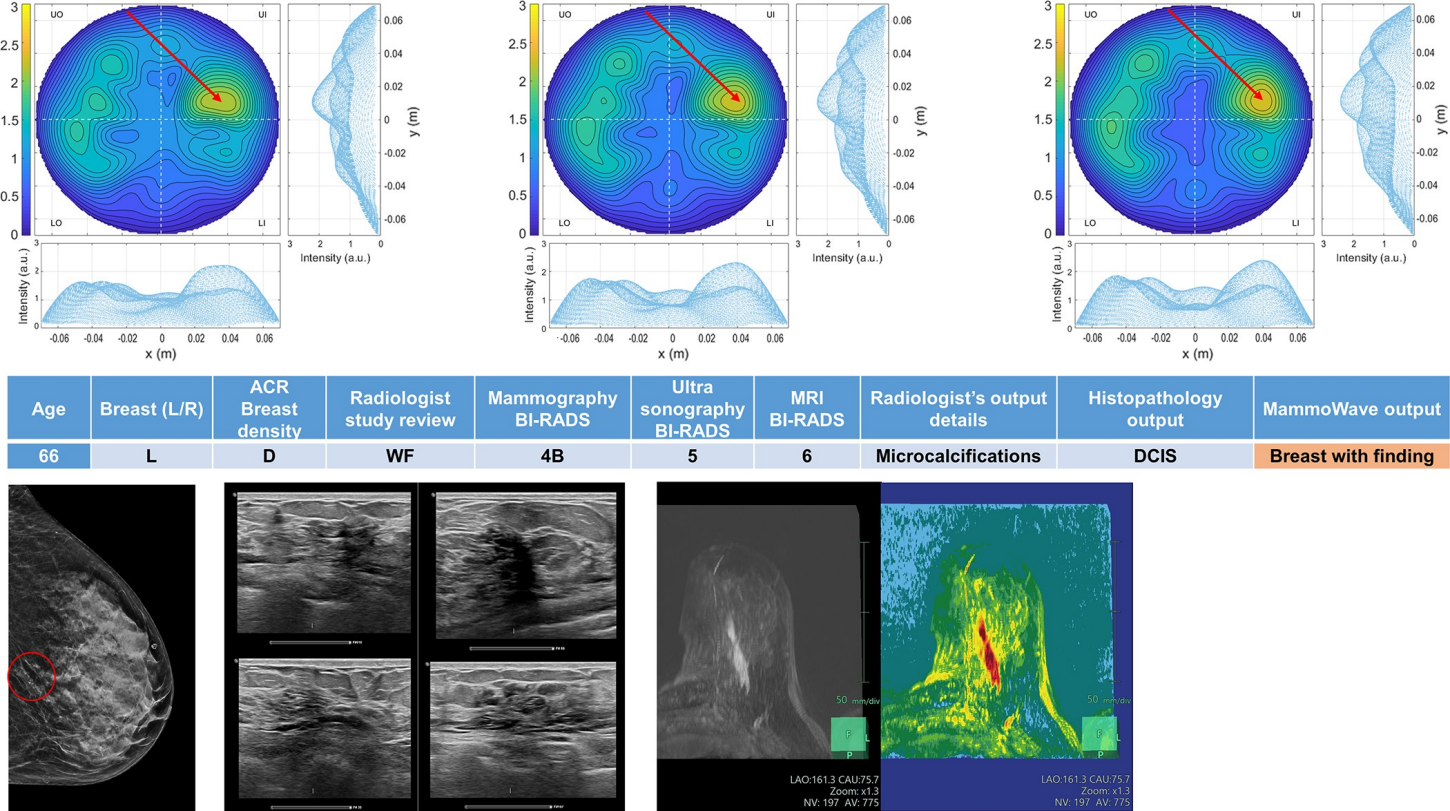
Example of WF breast: Mammographic high-density (ACR D), left breast of 66 years old woman, with a group of microcalcifications. Histopathology output is also given in the insert. Microwave images, normalized to unitary average of the intensity, are given in the top row for three different conductivity weightings (from left to right: 0.3 S/m, 0.4 S/m and 0.5 S/m, respectively). Microwave images are given here as 2D images in the azimuthal, i.e., coronal plane. Moreover, 1D intensity projection on X and Y is displayed in the inserts. X and Y are given in meters; intensity is in arbitrary units. All microwave images show a non-homogeneous behavior, with a main peak indicated by the red arrows. The proposed rule-of-thumb classifies this breast as positive (the values of the microwave images’ selected features are provided in the S1B Table).

**Fig 5:**
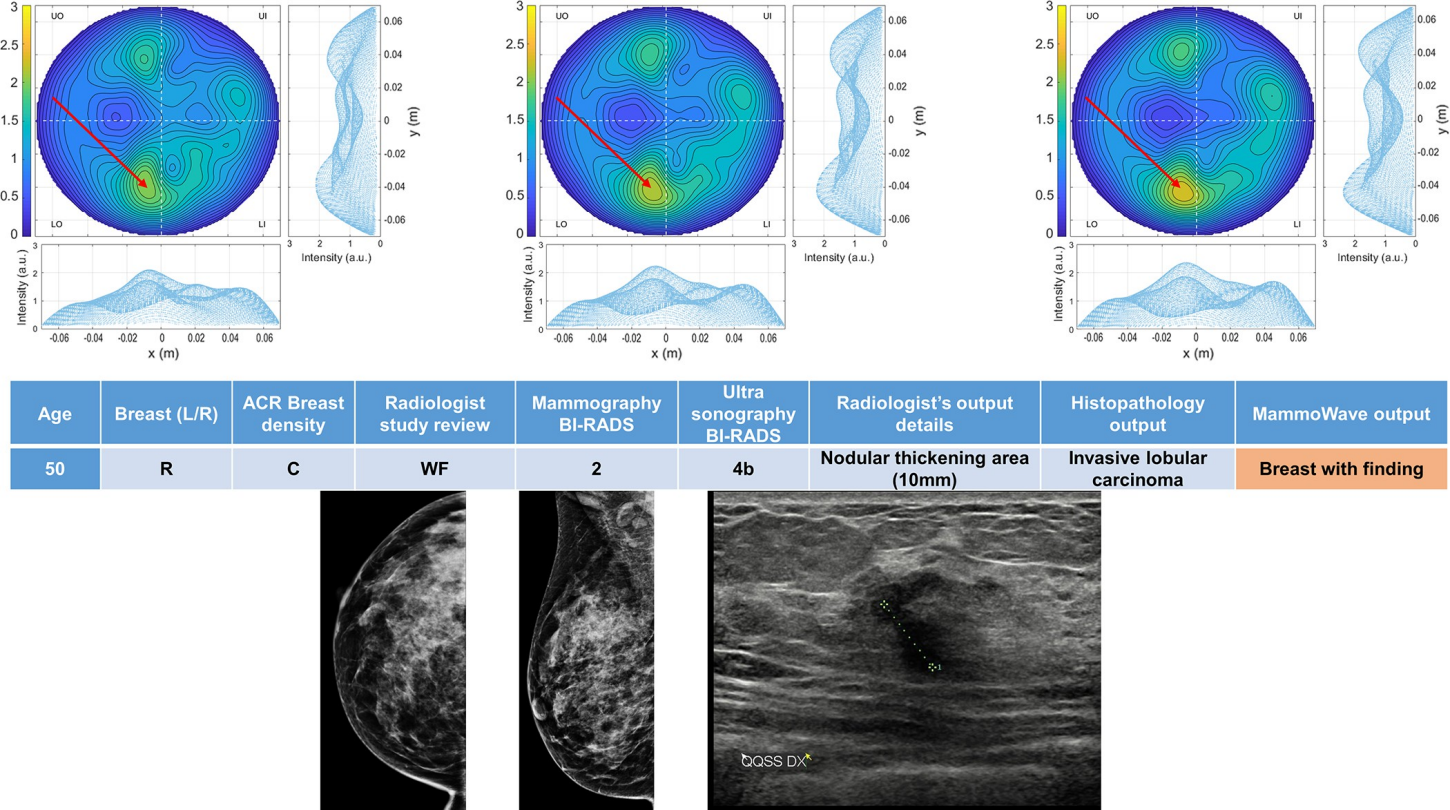
Example of WF breast: Mammographic heterogeneously dense (ACR C) right breast of 50 years old woman with a palpable nodule of 10 mm. Histopathology output is also given in the insert. Microwave images, normalized to unitary average of the intensity, are given in the top row for three different conductivity weightings (from left to right: 0.3 S/m, 0.4 S/m and 0.5 S/m, respectively). Microwave images are given here as 2D images in the azimuthal, i.e., coronal plane. Moreover, 1D intensity projection on X and Y is displayed in the inserts. X and Y are given in meters; intensity is in arbitrary units. All microwave images show a non-homogeneous behavior, with a main peak indicated by the red arrows. The proposed rule-of-thumb classifies this breast as positive (the values of the microwave images’ selected features are provided in the S1B Table).

**Fig 6:**
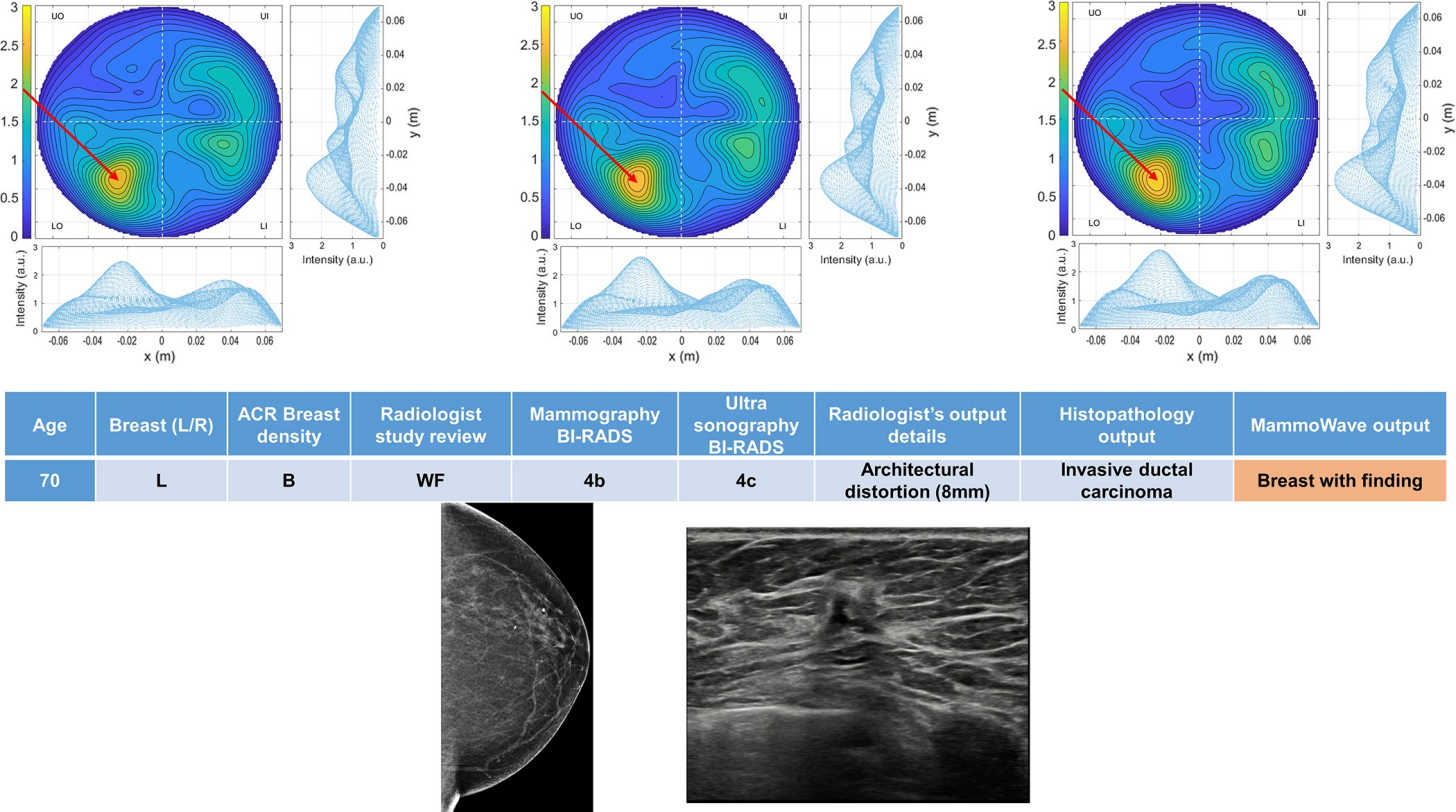
Example of WF breast: Mammographic low density (ACR B) left breast of 70 years old woman with a mammographic parenchymal distortion of 8 mm confirmed by DBT in the lower outer quadrant. Histopathology output is also given in the insert. Microwave images, normalized to unitary average of the intensity, are given in the top row for three different conductivity weightings (from left to right: 0.3 S/m, 0.4 S/m and 0.5 S/m, respectively). Microwave images are given here as 2D images in the azimuthal, i.e., coronal plane. Moreover, 1D intensity projection on X and Y is displayed in the inserts. X and Y are given in meters; intensity is in arbitrary units. All microwave images show a non-homogeneous behavior, with a main peak indicated by the red arrows. The proposed rule-of-thumb classifies this breast as positive (the values of the microwave images’ selected features are provided in the S1B Table).

Four breasts are shown here in more details as test cases, each with 3 of the selected conductivity weighed microwave images (obtained for conductivities values 0.3, 0.4 and 0.5 S/m, respectively). Fig 3 refers to NF breast, while Figs 4–6 refer to WF breasts. Microwave images, normalized to unitary average of the intensity, are given here as 2D images in the azimuthal, i.e., coronal plane. Moreover, 1D intensity projection on X and Y is displayed in the inserts. X and Y are given in meters; intensity is in arbitrary units. For each test case, the output and main findings of the radiologist study review, and correspondent conventional images, is also given. BI-RADS assessment of each available imaging technique is also given for WF breasts, together with the final assessment. In each figure, we also report the MammoWave *rule-of-thumb* output; the values of the microwave images’ selected features are given in the S1B Table.

In more details: Fig 3 refers to 50 years old woman, mammographic low-density (ACR B), left breast (DBT and ultrasonography BI-RADS 1). Fig 4 refers to mammographic high-density (ACR D), left breast of 66 years old woman, with a group of microcalcifications. Fig 5 refers to mammographic heterogeneously dense (ACR C) right breast of 50 years old woman with a palpable nodule of 10 mm. Fig 6 refers to mammographic low density (ACR B) left breast of 70 years old woman with a mammographic parenchymal distortion of 8 mm confirmed by DBT in the lower outer quadrant.

All WF microwave images show a non-homogeneous behaviour, with the main peak indicated by the red arrows.

MammoWave’s performance versus reference standard is given in Table 4.

**Table 4:** MammoWave’s performance in breast lesions detection against reference standard.

	All breast types
Accuracy	72.5% [278/383] (95%CI: 0.68–0.77)
Sensitivity	82.3% [224/272] (95%CI: 0.78–0.87)
Specificity	48.6% [54/111] (95%CI: 0.40–0.58).
	Low density breasts
Accuracy	72.0% [142/197] (95%CI: 0.66–0.78)
Sensitivity	89.6% [121/135] (95%CI: 0.84–0.95)
Specificity	33.8% [21/62] (95%CI: 0.22–0.45)
	High density breasts
Accuracy	73.3% [135/184] (95%CI: 0.67–0.80)
Sensitivity	75.0% [102/136] (95%CI: 0.68–0.82).
Specificity	68.7% [33/48] (95%CI: 0.56–0.82)
	Histology-confirmed breasts cancer
Sensitivity	82.2% [74/90] (95%CI: 0.78–0.87)

In addition, 351 women filled-in the questionnaire on their satisfaction related to MammoWave use in general and when compared to mammography: 99.5% showed willingness to recommend MammoWave scan; an average Net Promoter Score of 8.97 was achieved (score range 0 to 10); 94.9% felt reassured about MammoWave technology. More details on satisfaction scores are provided in Tables 5 and 6.

**Table 5:** Patients’ satisfaction related to MammoWave use in general.

	Not at all	A little	Moderately	Sufficiently	A lot
**Unpleasant**	308	33	7	3	0
**Painful**	348	2	1	0	0
**Uncomfortable**	188	91	42	21	9
**Long-lasting**	48	126	90	65	22
**Reassured**	4	3	11	80	253
**Informed**	1	2	3	73	272

**Table 6:** Patients’ satisfaction related to MammoWave when compared to mammography.

	Less	Equal	More
**Unpleasant**	314	28	9
**Painful**	340	10	1
**Uncomfortable**	250	52	49
**Long-lasting**	9	43	299

No adverse events have been recorded at the time of MammoWave procedures.

As supporting information, we also provide S1A Table where we give details of the radiological study review, MammoWave *rule-of-thumb* output, central assessor evaluation. The values of the MammoWave microwave images’ selected features are given in a S1B Table, as further supporting information, together with the number of S = 1 occurrences and MammoWave’s *rule-of-thumb* output.

## Discussion and conclusion

This first prospective breast microwave imaging study enrolled subjects having NF breasts and subjects having WF breasts. WF beasts included BC (both palpable and non-palpable). Before first volunteer enrolment, we defined the *rule-of-thumb* allowing MammoWave breast assessment, by introducing binary score S, such that “majority occurrence of 1” indicated WF breast, while a “majority occurrence of 0” indicated NF breast. Recruitment ended after achieving minimum number of subjects required to meet the primary endpoint. Sensitivity of 82%, greater than minimum 70% required as primary objective, was achieved for all breast types. This sensitivity was maintained when limiting the investigation to BC only. Obtained sensitivity values agree with our feasibility study [19], and with other studies which include measures of sensitivity [20]. In more details, obtained sensitivity values agree with [21,22], where only symptomatic patients were recruited, with [23], where only symptomatic patients with a palpable lump were recruited, and with [24], where only patients who were considered for biopsy (after routine imaging) were enrolled. Specifically, sensitivity of 74% and 76% for benign and malignant lesions, respectively are reported in [22], while [23] reports detection of 12 out of 13 benign breast lesions and 9 out of 11 breast cancers; in [24], an overall sensitivity of 63% was found.

Specificity value of approximately 50% was expected, since threshold *D*_*offset|feature*_ is calculated (for each feature) using median value obtained after recruiting first 15 subjects (in each site), all NF. Different threshold choices may lead to an increase in specificity. Specificity decreases in low-density breasts while it increases in high-density breasts; this may be related to our procedure to calculate the thresholds *D*_*offset|feature*_, i.e. using NF data regardless of breast density. Thus, for density-specific thresholds, higher specificity might be obtained. To enhance specificity, dedicated artificial intelligence (AI) algorithms may be implemented as reported in [25]; in this context, a retrospective analysis has been performed applying supervised machine learning (SML) algorithm on the data collected within the clinical trial here presented, achieving specificity >90% [26]. It should be noted that [21–24] did not address specificity.

MammoWave avoids using any subject-specific estimation, generating breast images without apriori knowledge of subject-specific breast dielectric properties, only utilizing free space dielectric properties in the algorithm. Concerning the conductivity, for each breast we produced five different conductivity values, in agreement with [19]. More specifically, feasibility investigation in [19] was used here to select the *methods*, *features*, and the *rule-of-thumb* (prospectively).

Visually inspecting the images, we can observe WF breasts have a more non-homogenous behaviour compared to NF breasts. This confirms our findings in [19], i.e., the contrast in dielectric properties between breast lesions and the surrounding tissues generates a peak in microwave images. From Table 3, we can verify that p-values of all selected features are <0.05 except for MIN_n of *method6*; thus, selected features for these prospective trials are statistically robust in discriminating between WF and NF breasts, confirming results presented in [19].

Interestingly, the proposed rule-of-thumb classifies the breasts given in Figs 4–6, as positive. In particular, Fig 4 refers to a 66 years old woman who noticed a sudden lump and asked new appointment in the Breast Unit. For instance, she followed opportunistic screening (she had previous mammography from screening program in less than two years) due to BI-RADS 4B new-appearance group of microcalcifications (overall diameter 30 mm) in lower quadrants. Following ultrasonography exam, core needle biopsy and MRI were performed to confirm interval ductal carcinoma in situ (DCIS).

Generally, trial participants found MammoWave examination pleasant and comfortable, highlighting their willingness to recommend MammoWave scan. Research is in progress to reduce acquisition time by ~50% without significantly impacting the performance.

A first limitation of this investigation is not considering subjects’ pre-menstrual information. Next trials will include such information, to check eventual correlation with MammoWave performances. Moreover, with the current set-up, it has not been possible to verify position of the breast and nipple inside the cup; slight hardware modifications are in process (such as transparent cups, fiber-optics cameras) to recover such information, which may reduce rate of discarded MammoWave outputs (probably due to bad subject positioning or subjects’ severe movements). In the next trials we plan to equip and test MammoWave with dedicated ergonomic cushion to facilitate subject positioning and reducing subjects’ severe movements. Another limitation we identified is that the thresholds *D*_*offset|feature*_ have been calculated using the first 15 subjects, all NF, regardless of breast density; this could explain the low specificity obtained for low-density breasts. We will investigate, retrospectively, if higher specificity might be obtained when using density-specific thresholds. Finally, the impact of *rule-of-thumb* on detection capabilities of the features/methods selection procedure, number of selected features/methods and features’ correlation has not been investigated; such investigation will be performed retrospectively. A dedicated retrospective analysis will be also performed to evaluate the possible correlation between lesion size, radiological features, histological, molecular cancer types (a preliminary analysis was presented in [27]), and detection in MammoWave imaging.

To conclude, microwave imaging is a promising new technology in breast radiology, avoiding discomfort and use of ionizing radiation. It employs low-power radio-frequency signals (<1mW), without any breast compression. Its safe nature allows the potential to become very relevant in screening for increasing coverage of female population and providing effective early breast lesion detection. Specifically, it can be seen as a complementary solution for making screening programs more inclusive, without safety restrictions such as age or frequency of use. Its impact and implication could be especially noticeable in increasing early-stage detection and reducing interval cancers. These prospective trials may represent another step for introducing microwave imaging into clinical practice, for helping in breast lesion identification in asymptomatic women. As such, further clinical trials are planned, also equipping MammoWave with dedicated AI algorithms, trained using data collected in the trials presented here.

## Supporting information

S1 Table Supporting information with patient and breast info (S1A Table) and methods/features (S1B Table). (PDF)

S1 Appendix (DOCX)
