# Supplementary material for: A multicentric, single arm, prospective, stratified clinical investigation to evaluate MammoWave’s ability in breast lesions detection
Source: PLoS One. 2023 Jul 14;18(7):e0288312. doi: 10.1371/journal.pone.0288312 (PMC10348515; doi:10.1371/journal.pone.0288312)
Supplement: S1 Table — (PDF) [file pone.0288312.s001.pdf]

Supporting information S1A Table: patient and breast info

Patient n°	Location	Breast (DX=right, SX=left)	Age	Breast Density (1=ACR A; 2=ACR B; 3=ACR C; 4=ACR D)	Discarded by Central Assessor (1=yes, 0=no)	Reference standard WF/NF (1=WF, 0=NF)	BIRADS	Info Lesions, if any	Pathology Output, if any	MammoWave rule-of-thumb output (1=WF, 0=NF)
01_016	TOLEDO	DX	34	2	0	1	BI-RADS 6	Spicular nodule inner interquadrant, 17x11mm	Invasive Duct Carcinoma II; triple -	1
01_016	TOLEDO	SX	34	2	0	0	BI-RADS 1	negative		0
01_017	TOLEDO	DX	47	4	1	1	BI-RADS 2	Duct ectasia. Cysts in UO Quadrant		1
01_017	TOLEDO	SX	47	4	0	1	BI-RADS 2	Duct ectasia. Cysts in upper interquadrant		1
01_018	TOLEDO	DX	39	3	0	0	BI-RADS 1	negative		0
01_018	TOLEDO	SX	39	3	0	1	BI-RADS 2	Duct ectasia in outer quadrants		1
01_019	TOLEDO	DX	33	3	0	1	BI-RADS 2	Cysts in upper quadrants		1
01_019	TOLEDO	SX	33	3	1	1	BI-RADS 2	Fibroadenomi in UI ,15 mm, and other in lower interquadrant, 2 cm.	Benign skin lesion	0
01_020	TOLEDO	DX	45	4	0	1	BI-RADS 3	Fibroadenomi (13 mm) in UO, and cyst (29 mm) in LO		1
01_020	TOLEDO	SX	45	4	0	1	BI-RADS 2	Cysts in upper interquadrant, ectasia Duct retroareolar and in lower interquadrant, cysts in lower interquadrant and in breast center		1
01_021	TOLEDO	DX	64	1	0	1	BI-RADS 6	Spicular nodule UO/outer interquadrant, 10 mm, with small Duct ectasia.	Invasive Lobular Carcinoma	1
01_022	TOLEDO	DX	32	4	0	1	BI-RADS 3	Nodule of 18 mm in outer interquadrant.	Benign skin lesion	1
01_023	TOLEDO	DX	43	4	1	0	BI-RADS 1	negative		1
01_023	TOLEDO	SX	43	4	0	1	BI-RADS 6	Noduli 32x34 mm UO quadrant, some ectasia Duct in UI.	Invasive Duct Carcinoma II	1
01_024	TOLEDO	DX	44	3	0	1	BI-RADS 3	Noduli 14x8 mm in upper interquadrant and some gangli.	Fibroadenomi	1
01_024	TOLEDO	SX	44	3	1	1	BI-RADS 2	Some milimetric cyst in upper interquadrant		1
01_025	TOLEDO	DX	46	3	1	1	BI-RADS 2	Minumum Duct ectasia in UO		1
01_025	TOLEDO	SX	46	3	0	1	BI-RADS 6	Distortion in UI quadrant. Minumum Duct ectasia in UO.	Invasive Lobular Carcinoma I; Int. Luminal	0
01_026	TOLEDO	DX	44	4	0	1	BI-RADS 2	Solid noduli of fibroadenomi (12 mm) in outer interquadrant, small cysts (retroareolar) and Duct ectasia		1
01_026	TOLEDO	SX	44	4	0	1	BI-RADS 2	Dispersed Duct ectasia		1
01_027	TOLEDO	DX	26	3	0	1	BI-RADS 3	Four solid fibroadenomi (12, 10, 14, 9 mm) in inner interquadrant retroareolar		1
01_027	TOLEDO	SX	26	3	1	1	BI-RADS 3	Solid fibroadenomi (7mm) in inner interquadrant		1

01_028	TOLEDO	DX	52	3	1	1	BI-RADS 2	Microcalcifications dispered around the breast, nodule (fibroadenomi calcificated of 5 mm) retroareolar inner quadrant.		1
01_028	TOLEDO	SX	52	3	0	1	BI-RADS 6	2 Spiculated nodules, of 10 mm and 8 mm, in UO quadrant (2-3 oclock), microcalcifications in UO.	Invasive Duct Carcinoma III	1
01_029	TOLEDO	DX	50	2	0	1	BI-RADS 6	Microcalcifications (carcinoma). Multifocal (3 locations) UO quadrant - Middle going to deepest part, near pectoralis.	Invasive Duct Carcinoma III; Luminal B	0
01_029	TOLEDO	SX	50	2	1	0	BI-RADS 1	negative		1
01_030	TOLEDO	DX	46	3	0	1	BI-RADS 3	Nodule of fibroadenomi in outer interquadrant		1
01_030	TOLEDO	SX	46	3	0	1	BI-RADS 2	Nodule of various fibroadenomi in UO		0
01_031	TOLEDO	DX	47	4	1	1	BI-RADS 3	Nodule of fibroadenomi of 26 mm in UO.	Fibroadenomi	1
01_031	TOLEDO	SX	47	4	1	0	BI-RADS 1	negative		1
01_032	TOLEDO	DX	55	3	1	1	BI-RADS 6	Mass in outer interquadrant 14x13x14mm.	Invasive Duct Carcinoma I	1
01_032	TOLEDO	SX	55	3	1	1	BI-RADS 6	Several masses in UO, outer interquadrant and LO 60x30x30mm.	Invasive Duct Carcinoma III	1
01_033	TOLEDO	DX	26	4	0	1	BI-RADS 2	Clearly bening findings: dispersed cysts		0
01_033	TOLEDO	SX	26	4	1	1	BI-RADS 2	Small fibroadenomi		1
01_034	TOLEDO	DX	24	4	0	1	BI-RADS 2	Duct ectasia		1
01_034	TOLEDO	SX	24	4	0	1	BI-RADS 2	Duct ectasia		1
01_035	TOLEDO	DX	43	3	0	1	BI-RADS 6	Carcinoma outer interquadrant and UO 31X26 mm, cyst in interquadrant 2 cm.	Intraductal Carcinoma	1
01_035	TOLEDO	SX	43	3	1	0	BI-RADS 1	negative		1
01_036	TOLEDO	DX	21	4	0	1	BI-RADS 2	Nodule Tail 12 mm		1
01_036	TOLEDO	SX	21	4	0	1	BI-RADS 2	Nodule in LO 21 mm.	Benign lesion; fibrosis	1
01_037	TOLEDO	DX	66	1	0	0	BI-RADS 1	negative		1
01_037	TOLEDO	SX	66	1	0	1	BI-RADS 6	Nodulo 19 mm retroareolar.	Invasive Duct Carcinoma II	1
01_038	TOLEDO	DX	54	2	0	1	BI-RADS 6	Big mass in outer interquadrant, associated gangli.	Invasive Duct Carcinoma II; Luminal	1
01_038	TOLEDO	SX	54	2	1	0	BI-RADS 1	negative		1
01_039	TOLEDO	DX	39	2	0	0	BI-RADS 1	negative		0
01_039	TOLEDO	SX	39	2	0	1	BI-RADS 3	Two noduli outer interquadrant (12 mm BIRAD 3, 14 mm BIRAD 4A)	Fibroadenomi	0
01_040	TOLEDO	DX	55	2	0	0	BI-RADS 1	negative		1
01_040	TOLEDO	SX	55	2	1	1	BI-RADS 6	Nodule with microcalcification in UO.	Invasive Duct Carcinoma III; Luminal A	1
01_041	TOLEDO	DX	67	2	0	1	BI-RADS 6	Nodule in lower interquadrant 6 mm.	Invasive Duct Carcinoma II; Luminal A	1
01_041	TOLEDO	SX	67	2	1	0	BI-RADS 1	negative		1
01_042	TOLEDO	DX	26	4	1	0	BI-RADS 1	negative		0

01_042	TOLEDO	SX	26	4	1	0	BI-RADS 1	negative		1
01_043	TOLEDO	DX	78	2	1	0	BI-RADS 1	negative		1
01_043	TOLEDO	SX	78	2	1	1	BI-RADS 6	3 mm noduli upper interquadrant.	Invasive Duct Carcinoma NA; Luminal A	1
01_044	TOLEDO	DX	48	3	0	0	BI-RADS 1	negative		0
01_044	TOLEDO	SX	48	3	1	1	BI-RADS 6	UO tail 14 mm and 6 mm with 8mm separation among them.	Invasive Duct Carcinoma III; Triple -	1
01_045	TOLEDO	DX	51	2	1	0	BI-RADS 1	negative		1
01_045	TOLEDO	SX	51	2	0	1	BI-RADS 6	Multiple noduli in outer quadrants with microcalcifications.	Invasive Duct Carcinoma II	1
01_046	TOLEDO	DX	62	2	1	0	BI-RADS 1	negative		1
01_046	TOLEDO	SX	62	2	0	1	BI-RADS 6	Noduli in UO quadrant 12x8 mm.	Invasive Duct Carcinoma II	1
01_047	TOLEDO	DX	60	2	1	0	BI-RADS 1	negative		1
01_047	TOLEDO	SX	60	2	1	1	BI-RADS 6	Noduli in UO quadrant 10x8x6mm.	Invasive Duct Carcinoma II; Luminal A	1
01_048	TOLEDO	DX	35	2	0	1	BI-RADS 6	Spiculated nodule in inner interquadrant.	Invasive Duct Carcinoma III; Luminal B	1
01_048	TOLEDO	SX	35	2	0	1	BI-RADS 2	Cyst in inner interquadrant		1
01_049	TOLEDO	DX	55	3	1	1	BI-RADS 3	Distortion in lower quadrants	Sclerosant Radial Scar	1
01_049	TOLEDO	SX	55	3	1	0	BI-RADS 1	negative		1
01_050	TOLEDO	DX	65	2	0	1	BI-RADS 6	Noduli in LO 15x13x10mm.	Invasive Duct Carcinoma II	1
01_050	TOLEDO	SX	65	2	0	0	BI-RADS 1	negative		1
01_051	TOLEDO	DX	47	3	0	1	BI-RADS 6	13x11 mm nodule in UO.	Invasive Duct Carcinoma I	1
01_051	TOLEDO	SX	47	3	0	0	BI-RADS 1	negative		0
01_052	TOLEDO	DX	45	4	1	1	BI-RADS 2	Low risk microcalcifications UO, outer interquadrant		0
01_052	TOLEDO	SX	45	4	1	1	BI-RADS 2	Dispersed Duct ectasia		1
01_053	TOLEDO	DX	46	2	0	1	BI-RADS 6	Nodule.	Invasive Lobular Carcinoma II, Int. Luminal	1
01_053	TOLEDO	SX	46	2	0	0	BI-RADS 1	negative		1
01_054	TOLEDO	DX	51	2	0	1	BI-RADS 2	Cyst inner interquadrant + dispersed Duct ectasia		1
01_054	TOLEDO	SX	51	2	0	1	BI-RADS 2	Cyst retroareolar + dispersed Duct ectasia		1
01_055	TOLEDO	DX	36	2	1	0	BI-RADS 1	negative		1
01_055	TOLEDO	SX	36	2	1	1	BI-RADS 3	10 mm noduli in outer interquadrant		1
01_056	TOLEDO	DX	46	2	1	1	BI-RADS 2	Duct ectasia		1
01_056	TOLEDO	SX	46	2	0	1	BI-RADS 2	2 cm cyst in Outer quadrants - Duct ectasia		1
01_057	TOLEDO	DX	56	3	1	1	BI-RADS 2	Duct ectasia retroareolar		1
01_057	TOLEDO	SX	56	3	1	1	BI-RADS 2	Duct ectasia retroareolar		1

01_058	TOLEDO	DX	53	2	0	1	BI-RADS 6	Nodule with microcalcification in upper quadrant 27x19x21.	Invasive Duct Carcinoma III; Luminal A	1
01_058	TOLEDO	SX	53	2	1	0	BI-RADS 1	negative		1
01_059	TOLEDO	DX	65	2	0	1	BI-RADS 2	Fibroadenomi in LI		1
01_059	TOLEDO	SX	65	2	0	1	BI-RADS 2	Angliolipoma noduli in UO quadrant		1
01_060	TOLEDO	DX	49	3	0	1	BI-RADS 3	16mm fibroadenomi retroareolar + dispersed cysts		0
01_060	TOLEDO	SX	49	3	0	1	BI-RADS 2	Dispersed cysts		1
01_061	TOLEDO	DX	50	4	0	1	BI-RADS 2	Duct ectasia, simple cyst 3cm retroareolar		1
01_061	TOLEDO	SX	50	4	0	1	BI-RADS 3	Distortion in UO quadrant.	Benign tissue; fibrocystic changes	1
01_062	TOLEDO	DX	46	2	0	1	BI-RADS 3	Distortion in outer quadrants.	Hyperplasia & adenonis + microcalcification s	1
01_062	TOLEDO	SX	46	2	1	0	BI-RADS 1	negative		1
01_063	TOLEDO	DX	59	2	1	1	BI-RADS 6	Duct ectasia + hidden cancer.	Invasive Duct Carcinoma III; Luminal A	1
01_063	TOLEDO	SX	59	2	1	0	BI-RADS 1	negative		1
01_064	TOLEDO	DX	49	1	0	1	BI-RADS 6	Spiculated nodule.	Invasive Duct Carcinoma II	1
01_064	TOLEDO	SX	49	1	1	0	BI-RADS 1	negative		1
01_065	TOLEDO	DX	41	2	0	1	BI-RADS 2	Clearly bening findings: dispersed cysts		0
01_065	TOLEDO	SX	41	2	0	1	BI-RADS 2	Cyst in UI		1
01_066	TOLEDO	DX	44	2	0	1	BI-RADS 3	Suspected fibroadenomi in outer interquadrant 20 mm. (Fibroadenomi)	Fibroadenomi	1
01_066	TOLEDO	SX	44	2	0	0	BI-RADS 1	negative		1
01_067	TOLEDO	DX	33	4	0	1	BI-RADS 2	cysts		0
01_067	TOLEDO	SX	33	4	0	1	BI-RADS 2	cysts		0
01_068	TOLEDO	DX	48	2	1	0	BI-RADS 1	negative		1
01_068	TOLEDO	SX	48	2	1	1	BI-RADS 6	Spiculated nodule 17x15mm retroareolar.	Invasive Duct Carcinoma II; Luminal B	1
01_069	TOLEDO	DX	38	3	0	0	BI-RADS 1	negative		0
01_069	TOLEDO	SX	38	3	0	1	BI-RADS 6	Nodule in UO quadrant, 44x26mm.	Invasive Duct Carcinoma II; Luminal B	0
01_070	TOLEDO	DX	42	2	0	1	BI-RADS 2	Some benign microcalcifications (less than in Left breast) + duct ectasia		1
01_070	TOLEDO	SX	42	2	0	1	BI-RADS 2	Cyst outer interquadrant, puntiform benign microcalcifications + duct ectasia		1
01_071	TOLEDO	DX	59	2	0	1	BI-RADS 2	Dispersed cysts + duct ectasia		1
01_071	TOLEDO	SX	59	2	0	1	BI-RADS 6	Nodule with microcalcifications 20x21 mm + cysts.	Invasive Duct Carcinoma III	1

01_072	TOLEDO	DX	38	3	0	0	BI-RADS 1	negative		0
01_072	TOLEDO	SX	38	3	1	0	BI-RADS 1	negative		1
01_073	TOLEDO	DX	42	3	1	0	BI-RADS 1	negative		1
01_073	TOLEDO	SX	42	3	1	0	BI-RADS 1	negative		1
01_074	TOLEDO	DX	39	2	0	1	BI-RADS 2	Clearly bening findings: dispersed cysts		0
01_074	TOLEDO	SX	39	2	0	1	BI-RADS 2	Smooth duct ectasia		1
01_075	TOLEDO	DX	60	3	0	1	BI-RADS 2	Duct ectasia, simple cysts		1
01_075	TOLEDO	SX	60	3	1	1	BI-RADS 2	Duct ectasia, simple cysts in LI		1
01_076	TOLEDO	DX	49	3	0	1	BI-RADS 2	Cysts + benign microcalcification in UO		1
01_076	TOLEDO	SX	49	3	1	1	BI-RADS 2	Cyst in UO		1
01_077	TOLEDO	DX	54	2	0	1	BI-RADS 2	Microcalcifications dispered around the breast		1
01_077	TOLEDO	SX	54	2	0	1	BI-RADS 6	Spiculated nodule with associated microcalcifications in upper quadrants.	Invasive Duct Carcinoma II; Int. Luminal	1
01_078	TOLEDO	DX	65	2	1	1	BI-RADS 2	Duct ectasia UO + cyst		1
01_078	TOLEDO	SX	65	2	0	1	BI-RADS 6	Spiculated nodule in upper quadrants, 16x14x10 mm.	Invasive Duct Carcinoma III	1
01_079	TOLEDO	DX	51	2	0	1	BI-RADS 2	Healthy distortion in UO		0
01_079	TOLEDO	SX	51	2	0	1	BI-RADS 2	Clearly bening findings: dispersed cysts		1
01_080	TOLEDO	DX	40	2	1	0	BI-RADS 1	negative		1
01_080	TOLEDO	SX	40	2	1	0	BI-RADS 1	negative		1
01_081	TOLEDO	DX	52	2	0	0	BI-RADS 1	negative		0
01_081	TOLEDO	SX	52	2	1	1	BI-RADS 6	2 Spiculated nodules, of 13 and 12 mm, with high risk microcalcifications in outer quadrants.	Invasive Duct Carcinoma II	0
01_082	TOLEDO	DX	51	3	0	1	BI-RADS 2	Duct ectasia		1
01_082	TOLEDO	SX	51	3	0	0	BI-RADS 1	negative		0
01_083	TOLEDO	DX	57	2	0	1	BI-RADS 6	5 mm nodule in Outer interquadrant	Invasive Ductal Carcinoma, HER2-positive	1
01_083	TOLEDO	SX	57	2	0	0	BI-RADS 1	negative		1
01_084	TOLEDO	DX	67	3	1	0	BI-RADS 1	negative		1
01_084	TOLEDO	SX	67	3	1	1	BI-RADS 6	Architectural distortion in UI/ upper interquadrant. Confirmed spiculated nodule with 2nd focus 8mm apart.	Invasive Lobular Carcinoma II; Int. Luminal	1
01_085	TOLEDO	DX	44	2	0	0	BI-RADS 1	negative		0
01_085	TOLEDO	SX	44	2	0	0	BI-RADS 1	negative		0
01_086	TOLEDO	DX	47	2	0	1	BI-RADS 2	Dispersed cysts + duct ectasia		1
01_086	TOLEDO	SX	47	2	0	1	BI-RADS 3	Distortion in outer interquadrant.	Sclerosant adenosis	1
01_087	TOLEDO	DX	63	2	1	0	BI-RADS 1	negative		1
01_087	TOLEDO	SX	63	2	0	1	BI-RADS 6	Two Irregular nodules in UO/outer quadrants:18x5 mm and 9 mm --> All hipoechoic region of 25x12 mm.	Carcinoma in situ II; Triple -	1
01_088	TOLEDO	DX	52	2	0	1	BI-RADS 6	9x11 mm nodule in upper quadrants.	Invasive Duct Carcinoma I	1

01_088	TOLEDO	SX	52	2	0	1	BI-RADS 2	Dispersed cysts + duct ectasia		1
01_089	TOLEDO	DX	43	2	1	0	BI-RADS 1	negative		1
01_089	TOLEDO	SX	43	2	1	0	BI-RADS 1	negative		1
01_090	TOLEDO	DX	40	3	1	0	BI-RADS 1	negative		1
01_090	TOLEDO	SX	40	3	1	0	BI-RADS 1	negative		1
01_091	TOLEDO	DX	60	2	0	1	BI-RADS 6	Distortion + microcalcifications in UO 20x22x16 mm.	Invasive Duct Carcinoma III; Luminal B	1
01_091	TOLEDO	SX	60	2	0	0	BI-RADS 1	negative		1
01_092	TOLEDO	DX	56	2	1	1	BI-RADS 2	Clearly bening findings: dispersed cysts		1
01_092	TOLEDO	SX	56	2	0	1	BI-RADS 2	Clearly bening findings: dispersed cysts		1
01_093	TOLEDO	DX	47	2	0	0	BI-RADS 1	negative		0
01_093	TOLEDO	SX	47	2	0	1	BI-RADS 2	milimetric cysts and microcalficications		1
01_094	TOLEDO	DX	63	2	0	1	BI-RADS 2	Dispersed benign microcalcifications		1
01_094	TOLEDO	SX	63	2	1	1	BI-RADS 2	Clearly bening findings: dispersed cysts		1
01_095	TOLEDO	DX	43	3	0	1	BI-RADS 2	Dispersed cysts + duct ectasia		1
01_095	TOLEDO	SX	43	3	0	1	BI-RADS 6	22mm nodule in Outer interquadrant, + dispersed cysts and duct ectasia.	Invasive Duct Carcinoma II; Int. Luminal	1
01_096	TOLEDO	DX	54	3	1	1	BI-RADS 2	Cysts		1
01_096	TOLEDO	SX	54	3	0	1	BI-RADS 6	21x10 mm nodule in UO.	Invasive Duct Carcinoma I; Luminal	1
01_097	TOLEDO	DX	46	2	0	1	BI-RADS 6	High risk microcalcifications in lower interquadrant. In RM the lesion seems duct ectasia in Lint and LI.	Duct Carcinoma in situ	1
01_097	TOLEDO	SX	46	2	1	1	BI-RADS 2	Duct ectasia + cyst retroareolar Inner interquadrant		1
01_098	TOLEDO	DX	45	4	0	1	BI-RADS 2	Dispersed cysts + duct ectasia		1
01_098	TOLEDO	SX	45	4	0	1	BI-RADS 2	Dispersed cysts + duct ectasia		1
01_099	TOLEDO	DX	66	3	1	0	BI-RADS 1	negative		1
01_099	TOLEDO	SX	66	3	0	1	BI-RADS 6	Distortion + microcalcifications in UO.	Duct Carcinoma in situ	1
01_100	TOLEDO	DX	42	2	0	0	BI-RADS 1	negative		1
01_100	TOLEDO	SX	42	2	0	0	BI-RADS 1	negative		0
01_101	TOLEDO	DX	44	2	0	0	BI-RADS 1	negative		1
01_101	TOLEDO	SX	44	2	0	1	BI-RADS 6	12mm Spiculated nodule in UI.	Invasive Duct Carcinoma II; Int. Luminal	1
01_102	TOLEDO	DX	35	2	1	1	BI-RADS 2	Dispersed cysts + duct ectasia		1
01_102	TOLEDO	SX	35	2	0	1	BI-RADS 2	Dispersed cysts + duct ectasia		1
01_103	TOLEDO	DX	61	2	0	1	BI-RADS 6	17x14x15 mm spiculated nodule in UI.	Invasive Apocrin Carcinoma II	1
01_103	TOLEDO	SX	61	2	0	1	BI-RADS 2	Dispersed cysts + duct ectasia		1
01_104	TOLEDO	DX	44	2	1	1	BI-RADS 2	Dispersed Duct ectasia		1
01_104	TOLEDO	SX	44	2	0	1	BI-RADS 2	Dispersed Duct ectasia (10 mm more focused on outer quadrants)		1

01_105	TOLEDO	DX	66	4	0	1	BI-RADS 2	Clearly bening findings: dispersed cysts		0
01_105	TOLEDO	SX	66	4	0	1	BI-RADS 6	Group of microcalcifications in lower quadrants (LI) 3cm diameter.	IntraDuct carcinoma	1
01_106	TOLEDO	DX	59	2	1	0	BI-RADS 1	negative		1
01_106	TOLEDO	SX	59	2	0	1	BI-RADS 6	20x18x18 mm spiculated nodule in innee quadrants LI.	Invasive Duct Carcinoma II; Luminal A	1
01_107	TOLEDO	DX	36	3	1	0	BI-RADS 1	negative		1
01_107	TOLEDO	SX	36	3	0	1	BI-RADS 2	Duct ectasia		1
01_108	TOLEDO	DX	43	2	0	0	BI-RADS 1	negative		0
01_108	TOLEDO	SX	43	2	1	0	BI-RADS 1	negative		1
01_109	TOLEDO	DX	54	3	0	1	BI-RADS 3	Asymmetry + cysts + duct ectasia		0
01_109	TOLEDO	SX	54	3	0	1	BI-RADS 2	Dispersed cysts + duct ectasia		1
01_110	TOLEDO	SX	39	2	1	0	BI-RADS 1	negative		1
01_111	TOLEDO	DX	44	2	1	0	BI-RADS 1	negative		1
01_111	TOLEDO	SX	44	2	1	1	BI-RADS 6	10x14x12 mm Spiculated nodule in UO.	Invasive Duct Carcinoma I; Luminal A	0
01_112	TOLEDO	DX	44	1	0	0	BI-RADS 1	negative		1
01_112	TOLEDO	SX	44	1	0	0	BI-RADS 1	negative		1
01_113	TOLEDO	DX	37	3	0	0	BI-RADS 1	negative		0
01_113	TOLEDO	SX	37	3	0	1	BI-RADS 6	High risk microcalcifications dispersed; prominent in 2 loci: 30mm upper interquadrant UO and 40 mm LI.	Duct Carcinoma in situ	1
01_114	TOLEDO	DX	63	1	0	1	BI-RADS 6	7 mm Spiculated nodule in outer interquadrant.	Invasive Duct Carcinoma I	1
01_114	TOLEDO	SX	63	1	1	0	BI-RADS 1	negative		1
01_115	TOLEDO	DX	20	3	0	1	BI-RADS 2	clearly bening finding		1
01_115	TOLEDO	SX	20	3	0	1	BI-RADS 3	(Benign skin lesion: fibrocystic mastopathy)	Benign skin lesion: fibrocystic mastopathy	0
01_116	TOLEDO	DX	64	2	1	1	BI-RADS 2	Duct ectasia in Outer quadrants		1
01_116	TOLEDO	SX	64	2	1	0	BI-RADS 1	negative		1
01_117	TOLEDO	DX	48	4	0	0	BI-RADS 1	negative		0
01_117	TOLEDO	SX	48	4	0	1	BI-RADS 2	9 mm cyst in outer interquadrant		1
01_118	TOLEDO	DX	45	3	1	0	BI-RADS 1	negative		1
01_119	TOLEDO	DX	41	2	1	0	BI-RADS 1	negative		1
01_119	TOLEDO	SX	41	2	0	0	BI-RADS 1	negative		1
01_120	TOLEDO	DX	40	2	1	1	BI-RADS 2	Cyst in UO		1
01_120	TOLEDO	SX	40	2	0	1	BI-RADS 2	Cyst in UO		1
01_121	TOLEDO	DX	66	1	1	1	BI-RADS 2	Clearly bening findings: dispersed cysts		1
01_121	TOLEDO	SX	66	1	0	1	BI-RADS 2	Clearly bening findings: dispersed cysts		1
01_122	TOLEDO	DX	40	1	0	0	BI-RADS 1	negative		1
01_122	TOLEDO	SX	40	1	0	0	BI-RADS 1	negative		1
01_123	TOLEDO	DX	55	2	0	1	BI-RADS 2	Dispersed milimetric cysts		1
01_123	TOLEDO	SX	55	2	0	1	BI-RADS 2	Dispersed milimetric cysts		1

01_124	TOLEDO	DX	56	1	0	1	BI-RADS 2	Cyst in lower quadrants		1
01_124	TOLEDO	SX	56	1	0	0	BI-RADS 1	negative		1
01_125	TOLEDO	DX	74	1	0	1	BI-RADS 2	clearly bening finding		1
01_125	TOLEDO	SX	74	1	0	1	BI-RADS 2	clearly bening finding		1
01_126	TOLEDO	DX	49	3	0	1	BI-RADS 6	Asymmetry UI/upper quadrants in mammography; UI nodule of 15 mm in US.	Invasive Duct Carcinoma II; Luminal	1
01_126	TOLEDO	SX	49	3	0	0	BI-RADS 1	negative		0
01_127	TOLEDO	DX	43	4	0	1	BI-RADS 2	Duct ectasia		1
01_127	TOLEDO	SX	43	4	0	1	BI-RADS 2	5 mm cyst in inner interquadrant + duct ectasia		1
01_128	TOLEDO	DX	41	2	1	1	BI-RADS 2	Smooth duct ectasia in UO quadrants		1
01_128	TOLEDO	SX	41	2	1	1	BI-RADS 2	Smooth duct ectasia in UO quadrants		0
01_129	TOLEDO	DX	42	2	0	0	BI-RADS 1	negative		1
01_130	TOLEDO	DX	40	2	0	1	BI-RADS 2	Dispersed Duct ectasia		1
01_130	TOLEDO	SX	40	2	0	1	BI-RADS 2	Dispersed Duct ectasia		1
01_131	TOLEDO	DX	61	2	0	0	BI-RADS 1	negative		1
01_131	TOLEDO	SX	61	2	0	1	BI-RADS 6	8x11x8 mm nodule in UO.	Invasive Duct Carcinoma II; Luminal B	1
01_132	TOLEDO	DX	60	3	0	1	BI-RADS 2	Cysts and duct ectasia		1
01_132	TOLEDO	SX	60	3	0	1	BI-RADS 6	10x10x8 mm nodule retroareolar.	Invasive Duct Carcinoma I; Int. Luminal	1
01_133	TOLEDO	DX	49	3	1	1	BI-RADS 3	Bi-rads 3 nodule in breast tail (30mm)		1
01_133	TOLEDO	SX	49	3	0	1	BI-RADS 3	Fibroadenomi 32x30 mm retroareolar		1
01_134	TOLEDO	DX	58	1	0	0	BI-RADS 1	negative		1
01_134	TOLEDO	SX	58	1	0	1	BI-RADS 6	8x7x7 mm nodule retroareolar.	Invasive Duct Carcinoma III; Luminal A	0
01_135	TOLEDO	DX	57	3	0	0	BI-RADS 1	negative		1
01_135	TOLEDO	SX	57	3	0	1	BI-RADS 6	8x4x7cm mass in Upper quadrants and outer interquadrants.	Invasive Duct Carcinoma III; Luminal B	1
01_136	TOLEDO	DX	48	4	0	0	BI-RADS 1	negative		1
01_136	TOLEDO	SX	48	4	0	1	BI-RADS 6	12x12x13mm mass in Outer interquadrant + 30mm fibroadenomi.	Invasive Lobular Carcinoma + Fibroadenoma	0
01_137	TOLEDO	DX	64	2	0	0	BI-RADS 1	negative		1
01_137	TOLEDO	SX	64	2	0	1	BI-RADS 6	4x4x5 mm mass in outer interquadrant and UO.	Invasive Duct Carcinoma I; Luminal A	1
01_138	TOLEDO	DX	65	4	0	1	BI-RADS 2	Dispersed cysts + duct ectasia		1
01_138	TOLEDO	SX	65	4	0	1	BI-RADS 6	6x4x5 mm spiculated nodule in tail and UO quadrant + Dispersed cysts + duct ectasia.	Invasive Duct Carcinoma II	1
01_139	TOLEDO	DX	45	4	0	1	BI-RADS 2	Dispersed cysts + duct ectasia		1

01_139	TOLEDO	SX	45	4	0	1	BI-RADS 2	Low risk microcalcifications in UO + dispersed cysts + duct ectasia --> Radial scar		1
01_140	TOLEDO	DX	46	3	0	1	BI-RADS 3	Low risk microcalcifications in UO + dispersed cysts + duct ectasia.	Fibrosis, microcalcification s	1
01_140	TOLEDO	SX	46	3	0	1	BI-RADS 2	Dispersed cysts		1
01_141	TOLEDO	DX	51	3	0	0	BI-RADS 1	negative		1
01_141	TOLEDO	SX	51	3	0	1	BI-RADS 2	Distortion in UO quadrant, echographically described as duct ectasia		0
01_142	TOLEDO	SX	69	2	0	1	BI-RADS 2	Asymmetry that was described echographically as Duct ectasia in axillary tail		1
01_143	TOLEDO	DX	46	3	1	0	BI-RADS 1	negative		1
01_143	TOLEDO	SX	46	3	1	1	BI-RADS 3	Low risk microcalcifications in LI quadrant + duct ectasia		1
01_144	TOLEDO	DX	68	3	1	0	BI-RADS 1	negative		1
01_144	TOLEDO	SX	68	3	1	1	BI-RADS 3	Distortion in outer interquadrant (probably radial scar),3mm hypoechoic area.		1
01_145	TOLEDO	DX	54	3	0	1	BI-RADS 6	Architectural distortion in UO 15x10x10 mm.	Lobular hyperplasia	0
01_145	TOLEDO	SX	54	3	0	1	BI-RADS 2	negative		0
01_146	TOLEDO	DX	52	3	0	1	BI-RADS 2	Distortion BIRAD 5 in upper interquadrant 17x20x22 mm.	Adenosis, microcalcification s and duct ectasia	1
01_146	TOLEDO	SX	52	3	1	0	BI-RADS 1	negative		1
01_147	TOLEDO	DX	47	2	0	0	BI-RADS 1	negative		1
01_147	TOLEDO	SX	47	2	1	1	BI-RADS 6	Architectural distortion in outer quadrants 7x7x7 mm.	Invasive Duct Carcinoma II; Luminal A	1
01_148	TOLEDO	DX	50	3	0	0	BI-RADS 1	Adenosis, papilomatosis and mastopatia in outer interquadrant 6x5x6 mm		1
01_148	TOLEDO	SX	50	3	0	1	BI-RADS 3	Adenosis, papilomatosis and mastopatia in outer interquadrant 6x5x6 mm DISTORTION + 15 mm fibroadenoma in UO.	Adenosis, papilomatosis and mastopatia	1
01_149	TOLEDO	DX	52	1	0	0	BI-RADS 1	negative		1
01_149	TOLEDO	SX	52	1	0	0	BI-RADS 1	negative		0
01_150	TOLEDO	DX	49	2	0	1	BI-RADS 2	Cyst in LI		1
01_150	TOLEDO	SX	49	2	1	0	BI-RADS 1	negative		1
01_151	TOLEDO	DX	49	2	1	1	BI-RADS 6	Microcalcifications in inner quadrants 20x20x16 mm + cysts + duct ectasia.	Duct Carcinoma in situ	1
01_151	TOLEDO	SX	49	2	1	1	BI-RADS 2	Dispersed low risk microcalcificatiions + duct ectasia + cysts		1
01_152	TOLEDO	DX	44	4	1	1	BI-RADS 2	Duct ectasia retroareolar		0
01_152	TOLEDO	SX	44	4	1	1	BI-RADS 2	Duct ectasia retroareolar		1
01_153	TOLEDO	DX	36	3	0	1	BI-RADS 2	Dispersed Duct ectasia (retroareolar)		1

01_153	TOLEDO	SX	36	3	1	1	BI-RADS 2	Dispersed Duct ectasia (retroareolar)		1
01_154	TOLEDO	DX	44	2	0	1	BI-RADS 2	Dispesed milimetric cysts		0
01_154	TOLEDO	SX	44	2	0	1	BI-RADS 2	Dispesed milimetric cysts		1
01_155	TOLEDO	DX	48	2	0	0	BI-RADS 1	negative		1
01_155	TOLEDO	SX	48	2	0	1	BI-RADS 2	Simple cyst in UO		1
01_156	TOLEDO	DX	37	3	1	0	BI-RADS 1	negative		1
01_157	TOLEDO	DX	37	3	0	1	BI-RADS 2	Milimetric cysts + duct ectasia in UO, upper quadrants		1
01_157	TOLEDO	SX	37	3	0	1	BI-RADS 2	Duct ectasia in UO		1
01_158	TOLEDO	DX	40	4	0	1	BI-RADS 3	18 mm noduli in LO.	Fibroadenomi	1
01_158	TOLEDO	SX	40	4	0	0	BI-RADS 1	negative		0
01_159	TOLEDO	DX	44	4	0	1	BI-RADS 2	Dispersed Duct ectasia		0
01_159	TOLEDO	SX	44	4	0	1	BI-RADS 2	Dispersed Duct ectasia		1
01_160	TOLEDO	DX	58	3	0	0	BI-RADS 1	negative		1
01_160	TOLEDO	SX	58	3	1	1	BI-RADS 2	Duct ectasia in LI quadrant		1
01_161	TOLEDO	DX	63	2	1	0	BI-RADS 1	negative		1
01_161	TOLEDO	SX	63	2	0	1	BI-RADS 6	Spiculated nodule 8x10x10 mm in UO.	Ductal Invasive Carcinoma I; Luminal A	1
01_162	TOLEDO	DX	42	2	1	0	BI-RADS 1	negative		1
01_162	TOLEDO	SX	42	2	0	0	BI-RADS 1	negative		1
01_163	TOLEDO	DX	52	3	0	1	BI-RADS 2	Dispersed cysts		1
01_163	TOLEDO	SX	52	3	0	1	BI-RADS 2	Dispersed cysts		1
01_164	TOLEDO	DX	43	3	0	1	BI-RADS 2	Dispersed cysts + duct ectasia		1
01_164	TOLEDO	SX	43	3	0	1	BI-RADS 2	Dispersed cysts + duct ectasia		1
01_165	TOLEDO	DX	37	2	0	1	BI-RADS 2	Dispersed cysts + duct ectasia		1
01_165	TOLEDO	SX	37	2	0	1	BI-RADS 2	Dispersed cysts + duct ectasia		1
01_166	TOLEDO	DX	58	3	0	1	BI-RADS 2	Marked duct ectasia		1
01_166	TOLEDO	SX	58	3	1	1	BI-RADS 2	Marked duct ectasia		1
01_167	TOLEDO	DX	57	2	0	1	BI-RADS 2	Microcalcifications in outer quadrants		1
01_168	TOLEDO	DX	52	4	1	1	BI-RADS 2	Puntiform microcalcifications + cysts + duct ectasia		1
01_168	TOLEDO	SX	52	4	0	1	BI-RADS 2	Puntiform microcalcifications + cysts + duct ectasia		1
01_169	TOLEDO	DX	46	3	1	1	BI-RADS 2	Dispersed cysts		1
01_169	TOLEDO	SX	46	3	0	1	BI-RADS 2	7 mm cyst retroareolar and UO + dispersed microcysts		1
01_170	TOLEDO	DX	49	4	0	1	BI-RADS 2	More dispersed cysts		1
01_170	TOLEDO	SX	49	4	0	1	BI-RADS 2	Dispersed cysts		1
01_171	TOLEDO	DX	35	3	0	1	BI-RADS 2	Noduli (duct ectasia) in upper quadrants		1
01_171	TOLEDO	SX	35	3	0	1	BI-RADS 3	10 mm noduli in LO BI-RADS 3		0
01_172	TOLEDO	DX	48	3	1	1	BI-RADS 2	Nodular area in outer quadrants (duct ectasia + hamartoma) + milimetric cysts		1
01_172	TOLEDO	SX	48	3	1	1	BI-RADS 2	Milimetric cysts + duct ecatasia		1
01_173	TOLEDO	DX	47	3	0	1	BI-RADS 2	Distortion + dispersed cysts		0
01_173	TOLEDO	SX	47	3	0	1	BI-RADS 2	Clearly bening findings: dispersed cysts		1

01_174	TOLEDO	DX	60	3	1	1	BI-RADS 6	Noduile in UI. No observable in US.	Papilar Carcinoma	1
01_174	TOLEDO	SX	60	3	1	1	BI-RADS 2	Dispersed cysts + duct ectasia		1
01_175	TOLEDO	DX	55	3	1	1	BI-RADS 2	Dispersed cysts		1
01_175	TOLEDO	SX	55	3	0	1	BI-RADS 6	Distortion + microcalcifications in upper quaderants + retroareolar.	Microcalcifications over lobular adenomatosis	1
01_176	TOLEDO	DX	55	1	0	0	BI-RADS 1	negative		1
01_176	TOLEDO	SX	55	1	0	1	BI-RADS 6	nodule in UO, 16 mm.	Ductal Invasive Carcinoma I; Luminal A	1
01_177	TOLEDO	DX	62	2	0	1	BI-RADS 2	Dispersed cysts		1
01_177	TOLEDO	SX	62	2	0	1	BI-RADS 2	Glandular asymmetry (Dispersed cysts + duct ectasia)		1
01_178	TOLEDO	DX	48	2	0	1	BI-RADS 2	Dispersed cysts + duct ectasia		1
01_178	TOLEDO	SX	48	2	0	1	BI-RADS 2	Dispersed cysts + duct ectasia		1
01_179	TOLEDO	DX	48	2	0	1	BI-RADS 2	Dispersed cysts + duct ectasia		1
01_179	TOLEDO	SX	48	2	0	1	BI-RADS 2	Dispersed cysts + duct ectasia		1
01_180	TOLEDO	DX	45	4	0	1	BI-RADS 2	Dispersed cysts + duct ectasia		1
01_180	TOLEDO	SX	45	4	0	1	BI-RADS 2	Dispersed cysts + duct ectasia		1
02_016	GENOVA	DX	62	2	0	1	BI-RADS 2	Architectural distortions Localisation: upper outer, anterior third Categories: benign		1
02_016	GENOVA	SX	62	2	0	1	BI-RADS 6	Architectural distortions	Invasive Ductal Carcinoma	1
02_017	GENOVA	DX	47	4	0	1	BI-RADS 2	Eco: Mass round, circumscribed, hypoechoic, no posterior features.		1
02_017	GENOVA	SX	47	4	0	1	BI-RADS 2	Eco: benign		1
02_018	GENOVA	DX	47	3	1	1	BI-RADS 2	Mammo: architectural distortions Eco: Mass round, circumscribed, hypoechoic, enhancement. Localisation upper outer, posterior third Categories benign		0
02_018	GENOVA	SX	47	3	0	1	BI-RADS 2	Mammo: architectural distortions Eco: Mass round, circumscribed, hypoechoic, no posterior features Localisation: upper outer, middle third Categories: suspicious	Corpuscolar Cyst	0
02_019	GENOVA	DX	51	1	0	0	BI-RADS 1	Mammo: negative		1
02_019	GENOVA	SX	51	1	1	0	BI-RADS 1	Mammo: negative		1
02_020	GENOVA	DX	56	2	0	0	BI-RADS 1	Mammo: negative		0
02_020	GENOVA	SX	56	2	1	0	BI-RADS 1	Mammo: negative		1
02_021	GENOVA	DX	54	2	1	0	BI-RADS 1	Mammo: negative		1

02_021	GENOVA	SX	54	2	0	0	BI-RADS 1	Mammo: negative		1
02_022	GENOVA	DX	55	2	1	0	BI-RADS 1	Mammo: negative		1
02_022	GENOVA	SX	55	2	1	0	BI-RADS 1	Mammo: negative		1
02_023	GENOVA	DX	59	2	1	0	BI-RADS 1	Mammo: negative		1
02_023	GENOVA	SX	59	2	0	0	BI-RADS 1	Mammo: negative		0
02_024	GENOVA	DX	69	1	1	0	BI-RADS 1	Mammo: negative		1
02_024	GENOVA	SX	69	1	1	0	BI-RADS 1	Mammo: negative		1
02_025	GENOVA	DX	64	2	0	1	BI-RADS 2	Mammo: Benign		1
02_025	GENOVA	SX	64	2	1	0	BI-RADS 1	Mammo: negative		1
02_026	GENOVA	DX	64	3	0	1	BI-RADS 6	Calcification, coarse heterogeneous, grouped	Ductal Carcinoma in situ, G3	1
02_026	GENOVA	SX	64	2	1	0	BI-RADS 1	Mammo: negative		1
02_027	GENOVA	DX	51	2	0	0	BI-RADS 1	Mammo: negative		0
02_027	GENOVA	SX	51	2	0	1	BI-RADS 3	Calcification, coarse heterogeneous, grouped		0
02_028	GENOVA	DX	65	1	0	1	BI-RADS 3	Calcification, coarse heterogeneous, grouped		1
02_028	GENOVA	SX	65	1	0	0	BI-RADS 1	Mammo: negative		1
02_029	GENOVA	DX	67	2	1	0	BI-RADS 1	Mammo: negative		1
02_029	GENOVA	SX	67	2	0	1	BI-RADS 6	Calcification	Cribiform Carcinoma, G2	1
02_030	GENOVA	DX	71	2	1	0	BI-RADS 1	Mammo: negative		1
02_030	GENOVA	SX	71	2	1	1	BI-RADS 6	Calcification, coarse heterogeneous, grouped	Neoplasia Infiltrante	1
02_031	GENOVA	DX	51	2	1	1	BI-RADS 2	Mammo: architectural distortions	Amartoma	0
02_031	GENOVA	SX	51	2	1	1	BI-RADS 2	Mammo: Benign		0
02_032	GENOVA	DX	24	4	1	1	BI-RADS 2	Eco: Mass round, circumscribed, complex cystic and solid, enhancement	Fibroadenoma	0
02_032	GENOVA	SX	24	4	1	1	BI-RADS 2	Eco: Benign		0
02_033	GENOVA	DX	53	2	0	0	BI-RADS 1	Mammo: negative		1
02_033	GENOVA	SX	53	2	0	1	BI-RADS 6	Mass round, circumscribed	Invasive Ductal Carcinoma, G2	1

02_035	GENOVA	DX	43	3	0	0	BI-RADS 1	Mammo: negative		1
02_035	GENOVA	SX	43	3	0	0	BI-RADS 1	Mammo: negative		1
02_036	GENOVA	DX	50	2	1	0	BI-RADS 1			1
02_036	GENOVA	SX	50	2	0	1	BI-RADS 2	Mammo: Calcification, amorphous, grouped		1
02_037	GENOVA	DX	59	2	0	0	BI-RADS 1	Mammo: negative		0
02_037	GENOVA	SX	59	2	0	1	BI-RADS 2	Mammo: Asymmetries, focal asymmetry	Benign Mastopathy	1
02_038	GENOVA	DX	43	2	1	1	BI-RADS 6	Mammo: Calcification, coarse heterogeneous, grouped<br>Eco: benign	Ductal Carcinoma in situ, G2	0
02_038	GENOVA	SX	43	2	0	1	BI-RADS 2	Mammo: benign<br>Eco: benign		1
02_039	GENOVA	DX	55	2	0	0	BI-RADS 1	Mammo: negative		1
02_039	GENOVA	SX	55	2	0	1	BI-RADS 2	Mammo: architectural distortions	Benign mastopathy B2: presenza di una lesione di natura benigna	1
02_040	GENOVA	DX	40	1	0	1	BI-RADS 2	Mammo: Benign		0
02_040	GENOVA	SX	40	1	0	0	BI-RADS 1	Mammo: negative		1
02_041	GENOVA	DX	51	2	0	1	BI-RADS 6	Mammo: Calcification, coarse heterogeneous, grouped	Ductal Carcinoma in situ, G2	1
02_041	GENOVA	SX	51	2	0	0	BI-RADS 1	Mammo: negative		1
02_042	GENOVA	DX	56	4	1	0	BI-RADS 1	Mammo: negative		1
02_042	GENOVA	SX	56	4	1	0	BI-RADS 1	Mammo: negative		1
02_043	GENOVA	DX	55	4	1	0	BI-RADS 1	Mammo: negative		1
02_043	GENOVA	SX	55	4	1	0	BI-RADS 1	Mammo: negative		1
02_044	GENOVA	DX	49	3	0	1	BI-RADS 2	Mammo: Benign<br>Eco: benign		1
02_044	GENOVA	SX	49	3	0	1	BI-RADS 6	Mammo: Calcification, coarse heterogeneous, linear Eco: mass	Invasive Ductal Carcinoma	0
02_045	GENOVA	DX	44	2	0	1	BI-RADS 6	Mammo: Architectural distortions, oval, circumscribed	Ductal Carcinoma in situ	1
02_045	GENOVA	SX	44	2	0	1	BI-RADS 6	Mass, oval, spiculated	Ductal Carcinoma in situ	1
02_046	GENOVA	DX	48	2	0	1	BI-RADS 2	Mammo: Benign<br>Eco: benign		1

02_046	GENOVA	SX	48	2	1	0	BI-RADS 1	Mammo: Mass, oval, obscured Eco: Mass, oval, circumscribed, heterogeneous	fibrosis, simple adenosis - B1: tessuto sano nessun tipo di anomalia)	0
02_047	GENOVA	DX	43	4	1	1	BI-RADS 2	Mammo: Benign		0
02_047	GENOVA	SX	43	4	1	0	BI-RADS 1	Mammo: negative		1
02_048	GENOVA	DX	59	2	0	0	BI-RADS 1	Mammo: negative		0
02_048	GENOVA	SX	59	2	0	0	BI-RADS 1	Mammo: negative		1
02_049	GENOVA	DX	54	2	0	0	BI-RADS 1	Mammo: negative		0
02_049	GENOVA	SX	54	2	0	0	BI-RADS 1	Mammo: negative		0
02_050	GENOVA	DX	45	4	1	0	BI-RADS 1	Mammo: negative		1
02_050	GENOVA	SX	45	4	0	0	BI-RADS 1	Mammo: negative		1
02_051	GENOVA	DX	43	4	0	0	BI-RADS 1	Mammo: negative		0
02_051	GENOVA	SX	43	4	0	0	BI-RADS 1	Mammo: negative		1
02_052	GENOVA	DX	46	2	1	0	BI-RADS 1	Mammo: negative		1
02_052	GENOVA	SX	46	2	1	0	BI-RADS 2	Mammo: negative		1
02_053	GENOVA	DX	42	4	1	0	BI-RADS 1	Mammo: negative		1
02_053	GENOVA	SX	42	4	1	0	BI-RADS 1	Mammo: negative		1
02_054	GENOVA	DX	57	2	1	0	BI-RADS 1	Mammo: negative		1
02_054	GENOVA	SX	57	2	0	0	BI-RADS 1	Mammo: negative		1
02_055	GENOVA	DX	54	2	1	1	BI-RADS 6	Mammo: Calcification, coarse heterogeneous, grouped	Ductal Carcinoma in situ	1
02_055	GENOVA	SX	54	2	1	0	BI-RADS 1	Mammo: negative		1
02_056	GENOVA	DX	54	2	1	0	BI-RADS 1	Mammo: negative		1
02_056	GENOVA	SX	54	2	1	1	BI-RADS 3	Mammo: Calcification, coarse heterogeneous, grouped	Iperplasia Duttale Atipica	0
02_057	GENOVA	DX	42	1	1	0	BI-RADS 1	Mammo: negative		1
02_057	GENOVA	SX	42	1	1	0	BI-RADS 1	Mammo: negative		1
02_058	GENOVA	DX	49	4	1	1	BI-RADS 2	Eco: Benign	Benign Cyst	0

02_058	GENOVA	SX	49	4	0	1	BI-RADS 2	Eco: Mass irregular, circumscribed, heterogeneous, combined pattern	Advanced Cystic Lesion	1
02_059	GENOVA	DX	60	4	0	1	BI-RADS 2	Eco: Mass oval, circumscribed, complex cystic and solid, combined pattern		1
02_059	GENOVA	SX	60	4	1	1	BI-RADS 2			1
02_060	GENOVA	DX	44	4	0	1	BI-RADS 2	Mammo: Benign		1
02_060	GENOVA	SX	44	4	0	1	BI-RADS 6	Mammo: Calcification, coarse heterogeneous, grouped	Invasive Ductal Carcinoma	1
02_061	GENOVA	DX	39	4	1	1	BI-RADS 2	Eco: Mass round, circumscribed, isoechoic, combined pattern		1
02_061	GENOVA	SX	39	4	0	1	BI-RADS 2	Eco: Benign		1
02_062	GENOVA	DX	67	4	1	0	BI-RADS 1	Eco: negative		1
02_062	GENOVA	SX	67	4	0	1	BI-RADS 2	Eco: Mass round, circumscribed, hypoechoic, enhancement	Fibrosis, adenosis, ductal ectasias	1
02_063	GENOVA	DX	39	4	0	0	BI-RADS 1	Eco: negative		0
02_063	GENOVA	SX	39	4	1	1	BI-RADS 2	Eco: Mass oval, circumscribed, heterogeneous	Fibroadenomi	0
02_064	GENOVA	DX	48	4	1	1	BI-RADS 2	Eco: Mass oval, circumscribed, complex cystic and solid	Monostrato di epitelio iperplastico micropapillare rivestito da metaplasia apocrina, verosimile cisti apocrina	1
02_064	GENOVA	SX	48	4	1	0	BI-RADS 1	Eco: negative		1
02_065	GENOVA	DX	46	2	1	0	BI-RADS 1	Mammo: negative Eco: negative		1
02_065	GENOVA	SX	46	2	1	1	BI-RADS 2	Mammo: Mass oval, circumscribed, high density Eco: Mass oval, circumscribed, heterogeneous		1
02_066	GENOVA	DX	55	4	0	1	BI-RADS 2	Eco: Benign		0
02_066	GENOVA	SX	55	4	0	1	BI-RADS 6	Eco: Mass oval, circumscribed, heterogeneous	Invasive Ductal Carcinoma	1
02_067	GENOVA	DX	62	3	0	1	BI-RADS 6	Mammo: Mass irregular, circumscribed, equal density	Invasive Ductal Carcinoma	0
02_067	GENOVA	SX	62	3	1	0	BI-RADS 1	Mammo: negative		1
02_068	GENOVA	DX	42	2	1	1	BI-RADS 2	Mammo: negative Eco: benign		0

02_068	GENOVA	SX	42	2	0	1	BI-RADS 2	Eco: Mass oval, circumscribed,hypoechoic, enhancement Mammo: Mass oval, circumscribed, high density		1
02_069	GENOVA	DX	19	4	0	0	BI-RADS 1	Eco: negative		0
02_069	GENOVA	SX	19	4	0	1	BI-RADS 2	Eco: Mass oval, circumscribed, hypoechoic	Fibroadenomi	1
02_070	GENOVA	DX	55	2	0	1	BI-RADS 6	Mammo: Mass oval, circumscribed, high density Eco: Mass oval, circumscribed,isoechoic	Invasive Ductal Carcinoma	1
02_070	GENOVA	SX	55	2	1	0	BI-RADS 1	Mammo: negative Eco: negative		1
02_071	GENOVA	DX	72	4	0	1	BI-RADS 2	Eco: associated features	Ductal Cyst with Columnar Iperplasia	0
02_071	GENOVA	SX	72	4	1	0	BI-RADS 1	Eco: negative		1
02_072	GENOVA	DX	53	4	1	1	BI-RADS 6	Eco: mass oval, circumscribed, isoechoic	Invasive Ductal Carcinoma	0
02_072	GENOVA	SX	53	4	1	1	BI-RADS 2	Eco: benign		1
02_073	GENOVA	DX	45	4	0	1	BI-RADS 2	Eco: benign		1
02_073	GENOVA	SX	45	4	0	1	BI-RADS 2	Eco: Mass oval, circumscribed, anaechoic	Fibroadenomi	0
02_074	GENOVA	DX	47	4	0	1	BI-RADS 2	Eco: Mass oval, circumscribed, hypoechoic	Mastopatia Fibrosa	1
02_074	GENOVA	SX	47	4	0	1	BI-RADS 3	Eco: Mass oval, spiculated, anaechoic	Fibroadenomi	1
02_075	GENOVA	DX	56	2	0	1	BI-RADS 2	Mammo: Benign		0
02_075	GENOVA	SX	56	2	0	1	BI-RADS 2	Mammo: architectural distortions	Fibroadenomi	0
02_076	GENOVA	DX	73	4	1	1	BI-RADS 2	Eco: mass	Mastopatia Fibrosa	0
02_076	GENOVA	SX	73	4	1	1	BI-RADS 2	Eco: Benign		0
02_077	GENOVA	DX	52	4	1	1	BI-RADS 6	Mammo: negative Eco: Benign	Invasive Lobular Carcinoma	0
02_077	GENOVA	SX	52	4	0	1	BI-RADS 2	Mammo: negative Eco: mass, oval, circumscribed, anaechoic		1
02_078	GENOVA	DX	63	2	1	1	BI-RADS 2	Mammo: mass oval, microlobulated, equal density		0
02_078	GENOVA	SX	63	2	0	0	BI-RADS 1	Mammo: negative		0
02_079	GENOVA	DX	47	4	0	0	BI-RADS 1	Eco: negative		0
02_079	GENOVA	SX	47	4	0	1	BI-RADS 3	Eco: Mass oval, circumscribed, anaechoic	Lesione sclerosante con microcalcificazioni	1

02_080	GENOVA	DX	41	2	1	1	BI-RADS 2	Mammo: architectural distortions Eco: Mass oval, circumscribed, anaechoic	Fibrosi	0
02_080	GENOVA	SX	41	2	1	0	BI-RADS 1	Mammo: negative Eco: negative		1
02_081	GENOVA	DX	62	2	0	0	BI-RADS 1	Mammo: negative		1
02_081	GENOVA	SX	62	2	0	1	BI-RADS 6	Mammo: mass oval, circumscribed, high density	Invasive Ductal Carcinoma	0
02_082	GENOVA	DX	55	2	1	0	BI-RADS 1	Mammo: negative		0
02_082	GENOVA	SX	55	2	0	1	BI-RADS 2	Mammo: mass oval, circumscribed, equal density	Lesione Papillare	1
02_083	GENOVA	DX	70	2	0	1	BI-RADS 2	Mammo: Mass oval, circumscribed, equal density Eco: mass oval, circumscribed,isoechoic	Fibroadenomi	1
02_083	GENOVA	SX	70	2	0	1	BI-RADS 2	Mammo: Mass oval, circumscribed, high density Eco: negative		1
02_084	GENOVA	DX	32	-	0	0	BI-RADS 1	Eco: negative		1
02_084	GENOVA	SX	32	-	0	1	BI-RADS 2	Eco: Mass oval, circumscribed, anaechoic	Fibroadenoma cellulato	1
02_085	GENOVA	DX	38	4	0	1	BI-RADS 6	Mammo: calcification (lower outer: known biopsy proven malignancy)	Invasive Ductal Carcinoma	1
02_085	GENOVA	SX	38	4	0	0	BI-RADS 1	Mammo: negative		0
02_086	GENOVA	DX	75	2	1	1	BI-RADS 6	Mammo: mass oval, circumscribed, high density, retro areolar tail (known biopsy proven malignancy)	Carcinoma Duttale Infiltrante + Metastasi Linfonodali	0
02_086	GENOVA	SX	75	2	0	1	BI-RADS 2	Mammo: Benign		1
02_087	GENOVA	DX	72	4	1	1	BI-RADS 6	Mammo: calcification coarse heterogeneous grouped, upper outer (known biopsy proven malignancy)	Ductal Carcinoma in situ, G2	1
02_087	GENOVA	SX	72	4	0	1	BI-RADS 2	Mammo: Benign		1
02_088	GENOVA	DX	69	2	1	0	BI-RADS 1	Mammo: negative		1
02_088	GENOVA	SX	69	2	0	1	BI-RADS 2	Mammo: mass oval, circumscribed, high density, upper inner (moderate suspicious for malignancy)	Fibrosis	1
02_089	GENOVA	DX	78	2	0	1	BI-RADS 6	Mammo: mass oval, circumscribed, high density, upper outer (known biopsy proven malignancy)	Invasive Ductal Carcinoma	1
02_090	GENOVA	DX	75	2	1	0	BI-RADS 1	Eco: negative		1
02_090	GENOVA	SX	75	2	1	1	BI-RADS 3	Mammo: architectural distortions, upper outer, middle third, highly suggestive of malignancy Eco: mass, upper outer		1

								middle third, highly suggestive of malignancy		
02_091	GENOVA	DX	66	2	1	1	BI-RADS 6	Mammo: architectural distortions, upper outer, anterior third, highly suggestive of malignancy. Eco: mass, indistinct, irregular, hypoechoic, upper outer middle third, highly suggestive of malignancy. (invasive lobular carcinoma)	Invasive Lobular Carcinoma	1
02_091	GENOVA	SX	66	2	1	0	BI-RADS 1	Mammo: negative Eco: negative		1
02_092	GENOVA	DX	52	4	0	1	BI-RADS 2	Eco: mass upper outer		1
02_092	GENOVA	SX	52	4	0	1	BI-RADS 6	Eco: mass upper outer, highly suggestive of malignancy.	Invasive Ductal Carcinoma	1
02_093	GENOVA	DX	50	3	0	1	BI-RADS 6	Mammo: Benign Eco: mass upper outer, moderate suspicious for malignancy.	Invasive Lobular Carcinoma	1
02_093	GENOVA	SX	50	3	0	1	BI-RADS 2	Mammo: Benign Eco: mass upper outer (benign)		1
02_094	GENOVA	DX	71	2	0	1	BI-RADS 6	Mammo: architectural distortions, upper inner, highly suggestive of malignancy. Eco: mass, upper inner, highly suggestive of malignancy.		1
02_094	GENOVA	SX	71	2	1	1	BI-RADS 6	Mammo: negative Eco: mass lower outer, highly suggestive of malignancy.		1
02_095	GENOVA	DX	56	2	1	0	BI-RADS 1	Mammo: negative Eco: negative		1
02_095	GENOVA	SX	56	2	0	1	BI-RADS 6	Mammo: mass, upper outer, high suspicious for malignancy. Eco: mass, upper outer, high suspicious for malignancy.	Invasive Ductal Carcinoma	1
02_096	GENOVA	DX	50	4	0	1	BI-RADS 6	Mammo: negative Eco: mass upper outer, high suspicious for malignancy. (invasive ductal carcinoma)	Invasive Ductal Carcinoma	0
02_096	GENOVA	SX	50	4	1	0	BI-RADS 1	Mammo: negative Eco: negative		1
02_097	GENOVA	DX	54	1	1	1	BI-RADS 6	Eco: mass, upper outer, highly suggestive of malignancy. MRI: mass, upper outer, highly suggestive of malignancy. (ADH)		1
02_097	GENOVA	SX	54	1	0	1	BI-RADS 6	Eco: negative MRI: mass, upper outer, known biopsy proven malignancy.		1
02_098	GENOVA	DX	61	2	1	1	BI-RADS 6	Mammo: mass, upper outer, highly suggestive of malignancy. Eco: mass, upper outer, highly suggestive of malignancy.	Invasive Ductal Carcinoma	1
02_098	GENOVA	SX	61	2	0	0	BI-RADS 1	Mammo: negative Eco: negative		1

02_099	GENOVA	DX	55	4	1	0	BI-RADS 1	Mammo: negative Eco: mass, retro areolar tail, low sospicious for malignancy.		0
02_099	GENOVA	SX	55	4	1	1	BI-RADS 6	Mammo: negative Eco: mass upper outer, high suspicious for malignancy.	Invasive Ductal Carcinoma, G3	0
02_100	GENOVA	DX	70	1	1	0	BI-RADS 1	Mammo: negative Eco: negative		1
02_100	GENOVA	SX	70	1	1	1	BI-RADS 6	Mammo: mass, upper outer, moderate suspicious for malignancy. Eco: mass, upper outer, highly suggestive of malignancy. (invasive ductal carcinoma)	Invasive Ductal Carcinoma	1
02_101	GENOVA	DX	70	2	0	0	BI-RADS 1	Mammo: negative Eco: negative		1
02_101	GENOVA	SX	70	2	0	1	BI-RADS 6	Mammo: architectural distortions, lower outer, moderate sospicious for malignancy. Eco: mass, central tail, highly suggestive of malignancy.	Invasive Ductal Carcinoma	1
02_102	GENOVA	DX	66	2	0	1	BI-RADS 6	Mammo: mass, upper outer, moderate suspicious for malignancy. Eco: mass, upper outer, highly suggestive of malignancy.	Invasive Ductal Carcinoma	1
02_102	GENOVA	SX	66	2	1	0	BI-RADS 1	Mammo: negative Eco: negative		1
02_103	GENOVA	DX	56	1	0	1	BI-RADS 6	Mammo: negative Eco: mass lower inner, highly suggestive of malignancy.	Invasive Ductal Carcinoma	0
02_103	GENOVA	SX	56	1	0	0	BI-RADS 1	Mammo: negative Eco: negative		0
02_104	GENOVA	DX	52	1	1	0	BI-RADS 1	Mammo: negative Eco: negative		1
02_104	GENOVA	SX	52	1	0	1	BI-RADS 2	Mammo: mass, lower outer, moderate suspicious for malignancy. Eco: mass, lower outer, moderate suspicious for malignancy.	Fibroadenoma	1
02_105	GENOVA	DX	52	2	0	0	BI-RADS 1	Mammo: negative		0
02_105	GENOVA	SX	52	2	0	1	BI-RADS 6	Mammo: mass, upper outer, high sospicious for malignancy.	Invasive Ductal Carcinoma	1
02_106	GENOVA	DX	75	2	0	0	BI-RADS 1	Mammo: negative Eco: negative		1
02_106	GENOVA	SX	75	2	0	1	BI-RADS 6	Mammo: mass, upper inner, high suspicious for malignancy. Eco: mass, upper inner, high suspicious for malignancy.	Invasive Ductal Carcinoma	1
02_107	GENOVA	DX	72	3	0	0	BI-RADS 1	Mammo: negative Eco: negative		1
02_107	GENOVA	SX	72	3	1	1	BI-RADS 2	Mammo: mass, retro areolar tail, low sospicious for malignancy. Eco: mass, retro areolar tail, low sospicious for malignancy.	Fibroadenoma	0

02_108	GENOVA	DX	49	1	1	1	BI-RADS 6	Eco: mass, retro areolar tail, high sospicious for malignancy.	Invasive Ductal Carcinoma	1
02_108	GENOVA	SX	49	1	1	0	BI-RADS 1	Eco: negative		1
02_109	GENOVA	DX	53	3	1	1	BI-RADS 6	Mammo: mass, round, obscured, high density, lower outer, middle third, moderate sospicious for malignancy. Eco: mass, irregular, spiculated, hypoechoic, lower outer, middle third, highly suggestive of malignancy.	Invasive Ductal Carcinoma	0
02_109	GENOVA	SX	53	3	0	0	BI-RADS 1	Mammo: negative Eco: negative		0
02_110	GENOVA	DX	44	4	1	1	BI-RADS 3	Eco: mass, oval, circumscribed, hypoechoic, lower inner, middle third, probably benign		0
02_110	GENOVA	SX	44	4	1	1	BI-RADS 2	Eco: mass, oval, circumscribed, hypoechoic, lower inner, middle third, benign.		1
03_016	MILANO	DX	60	2	1	0	BI-RADS 1	Mammo: negative Eco: negative		1
03_016	MILANO	SX	60	2	1	0	BI-RADS 1	Mammo: negative Eco: negative		1
03_017	MILANO	DX	54	3	0	1	BI-RADS 2	Mammo: mass, oval, circumscribed, high density, lower outer (benign) Eco: mass, oval, circumscribed, hypoechoic, no posterior features, lower outer		1
03_017	MILANO	SX	54	3	0	0	BI-RADS 1	Mammo: negative Eco: negative		0
03_018	MILANO	DX	36	4	1	0	BI-RADS 1	Eco: negative		1
03_018	MILANO	SX	36	4	0	1	BI-RADS 2	Eco: mass, oval, microlobulated, hypoechoic, lower outer, anterior third, low suspicious for malignancy.	Fibroadenoma (categoria B2 sec European Guidelines)	1
03_019	MILANO	DX	56	2	1	0	BI-RADS 1	Mammo: negative Eco: negative		1
03_019	MILANO	SX	56	2	1	0	BI-RADS 1	Mammo: negative Eco: negative		1
03_020	MILANO	DX	76	2	0	1	BI-RADS 6	Mammo: mass, round, spiculated high density, upper outer middle third, distance from the nipple 55mm (highly suggestive of malignancy) Eco: mass, round, spiculated, hypoechoic, upper outer middle third, distance from the nipple 55mm (highly suggestive of malignancy)	Carcinoma Infiltrante (categoria B5b sec. European Guidelines), con aspetti di tipo solidopapillare con associato muco extracellulare, grado G2 sec Nottingham Score.	1
03_020	MILANO	SX	76	2	1	0	BI-RADS 1	Mammo: negative Eco: negative		1
03_021	MILANO	DX	68	4	0	0	BI-RADS 1	Eco: negative		0
03_021	MILANO	SX	68	4	1	1	BI-RADS 2	Eco: mass, oval,circumscribed, hypoechoic,upper outer, anterior third,	Papillary Lesion	0

								distance from the nipple 10mm (suspicious)		
03_022	MILANO	DX	69	1	0	1	BI-RADS 6	Eco: mass, oval, indistinct, hypoechoic,upper inner, middle third (high suspicious for malignancy)	Iinvasive Ductal Carcinoma, G1	1
03_022	MILANO	SX	69	1	0	1	BI-RADS 2	Eco: mass, round,circumscribed, anaechoic, lower outer, anterior third		1
03_023	MILANO	DX	58	3	0	0	BI-RADS 1	Mammo: negative Eco: negative		0
03_023	MILANO	SX	58	3	0	1	BI-RADS 6	Mammo: calcification, fine pleomorphic, grouped, upper outer (highly suggestive of malignancy) Eco: calcification, upper outer (highly suggestive of malignancy)	Ductal Carcinoma in situ,G2	0
03_024	MILANO	DX	39	1	1	0	BI-RADS 1	Eco: negative		1
03_024	MILANO	SX	39	1	0	1	BI-RADS 6	Eco: mass, round, indistinct, hypoechoic,lower inner, (highly suggestive of malignancy)	Ductal Carcinoma in situ, G2	1
03_025	MILANO	DX	62	3	0	0	BI-RADS 1	Mammo: negative Eco: negative		1
03_025	MILANO	SX	62	3	0	0	BI-RADS 1	Mammo: negative Eco: negative		1
03_026	MILANO	DX	45	4	0	0	BI-RADS 1	Eco: negative		0
03_026	MILANO	SX	45	4	1	1	BI-RADS 2	Eco: mass, oval, circumscribed, hypoechoic,no posterior features, upper outer		1
03_027	MILANO	DX	61	1	1	0	BI-RADS 1	Eco: negative		1
03_027	MILANO	SX	61	1	0	1	BI-RADS 6	Eco: mass, irregular, indistinct, hypoechoic,no posterior features, lower inner (known biopsy proven malignancy)	Invasive Ductal Carcinoma, G3	1
03_028	MILANO	DX	22	4	0	0	BI-RADS 1	Eco: negative		1
03_028	MILANO	SX	22	4	0	0	BI-RADS 1	Eco: negative		0
03_029	MILANO	DX	49	4	0	0	BI-RADS 1	Eco: negative		0
03_029	MILANO	SX	49	4	0	1	BI-RADS 2	Eco: mass, oval, circumscribed, hypoechoic,no posterior features, upper outer		1
03_030	MILANO	DX	27	4	1	0	BI-RADS 1	Eco: negative		1
03_030	MILANO	SX	27	4	0	1	BI-RADS 2	Eco: mass, oval, circumscribed, hypoechoic,no posterior features, upper inner		0
03_031	MILANO	DX	51	1	0	0	BI-RADS 1	Eco: negative		1
03_031	MILANO	SX	51	1	0	0	BI-RADS 1	Eco: negative		0
03_032	MILANO	DX	26	4	0	1	BI-RADS 3	Eco: mass, oval, circumscribed, hypoechoic,no posterior features, upper outer (probably benign)		0
03_032	MILANO	SX	26	4	0	1	BI-RADS 3	Eco: mass, oval, circumscribed,hypoechoic, no posterior features, upper outer (probably benign)		0
03_033	MILANO	DX	29	4	1	1	BI-RADS 2	Eco: mass, oval, circumscribed, hypoechoic,no posterior features, uppe inner		0
03_033	MILANO	SX	29	4	1	1	BI-RADS 2	Eco: mass, lower inner		0

03_034	MILANO	DX	77	1	0	0	BI-RADS 1	Eco: negative		1
03_034	MILANO	SX	77	1	0	1	BI-RADS 6	Eco: mass, irregular, microlobulated, hypoechoic,no posterior features, upper outer (known biopsy proven malignancy)	Invasive Ductal Carcinoma, G3	1
03_035	MILANO	DX	60	1	1	0	BI-RADS 1	Eco: negative		1
03_035	MILANO	SX	60	1	0	1	BI-RADS 6	Eco: mass, irregular, microlobulated, hypoechoic,no posterior features, lower inner (high suspicious for malignancy)	Carcinoma papillare intraduttale con associati aspetti di infiltrazione stromale. Grado istologico (Nottingham Score): G2.	1
03_036	MILANO	DX	50	4	0	1	BI-RADS 2	Mammo: mass, oval, indistinct, equal density, axillary tail (4B: moderate suspicious for malignancy) Eco: mass, oval, indistinct, hypoechoic, no posterior features, axillary (4B: moderate suspicious for malignancy)	Fibrosi e focali aspetti di adenosi (categoria B2 sec European Guidelines)	0
03_036	MILANO	SX	50	4	0	0	BI-RADS 1	Mammo: negative Eco: negative		0
03_037	MILANO	DX	50	4	0	1	BI-RADS 6	Eco: mass, irregular, indistinct, hypoechoic,no posterior features, upper outer (know biopsy proven malignancy)	Invasive Ductal Carcinoma, G2	1
03_037	MILANO	SX	50	4	0	0	BI-RADS 1	Eco: negative		0
03_038	MILANO	DX	39	4	1	0	BI-RADS 1	Eco: negative		0
03_038	MILANO	SX	39	4	1	0	BI-RADS 1	Eco: negative		1
03_039	MILANO	DX	61	1	0	0	BI-RADS 1	Eco: negative		0
03_039	MILANO	SX	61	1	0	1	BI-RADS 6	Eco: mass, irregular, indistinct, hypoechoic,shadowing, upper inner (know biopsy proven malignancy	Invasive Ductal Carcinoma, G3	0
03_040	MILANO	DX	25	4	0	1	BI-RADS 2	Eco: mass, oval, circumscribed, hypoechoic,no posterior features, lower outer		0
03_040	MILANO	SX	25	4	0	1	BI-RADS 2	Eco: mass, oval, circumscribed, hypoechoic,no posterior features, lower inner		1
03_041	MILANO	DX	62	1	1	0	BI-RADS 1	Eco: negative		1
03_041	MILANO	SX	62	1	0	1	BI-RADS 6	Eco: mass, irregular, spiculated, hypoechoic,shadowing, upper inner (know biopsy proven malignancy)	Invasive Ductal Carcinoma, G3	1
03_042	MILANO	DX	50	4	0	1	BI-RADS 6	Eco: mass, irregular, indistinct, hypoechoic,no posterior features, upper inner (know biopsy proven malignancy)	Invasive Ductal Carcinoma, G2	0
03_042	MILANO	SX	50	4	0	0	BI-RADS 1	Eco: negative		0
03_043	MILANO	DX	49	1	0	1	BI-RADS 2	Eco: benign		1
03_043	MILANO	SX	49	1	0	1	BI-RADS 2	Eco: mass, round, circumscribed, hypoechoic,no posterior features, lower inner (low suspicious for malignancy)	Fibroadenoma (B2 sec. European Guidelines)	1

03_044	MILANO	DX	47	1	0	0	BI-RADS 1	Eco: negative		1
03_044	MILANO	SX	47	1	1	1	BI-RADS 6	Eco: mass, irregular, microlobulated, hypoechoic, shadowing, upper outer (known biopsy proven malignancy)	Invasive Ductal Carcinoma, G3	1
03_045	MILANO	DX	73	1	0	1	BI-RADS 2	Eco: mass, irregular, upper inner (moderate suspicious malignancy)	Tessuto Fibroadiposo con Infiltrato di Macrofagi Schiumosi (categoria B2 sec European guidelines)	1
03_045	MILANO	SX	73	1	0	1	BI-RADS 4B	Eco: mass, irregular, upper inner (moderate suspicious malignancy)		1
03_046	MILANO	DX	65	4	1	0	BI-RADS 1	Eco: negative		1
03_046	MILANO	SX	65	4	1	1	BI-RADS 6	Eco: mass, irregular, upper outer (highly suggestive of malignancy)	Carcinoma infiltrante (categoria B5b sec. European Guidelines). Nel materiale in esame, le caratteristiche morfofenotipiche favoriscono maggiormente l'istotipo non speciale, grado G2-G3 sec Nottingham Score.	0
03_047	MILANO	DX	46	1	1	0	BI-RADS 1	Eco: negative		1
03_047	MILANO	SX	46	1	1	0	BI-RADS 1	Eco: heterogeneous background echotexture		1
03_048	MILANO	DX	40	4	0	1	BI-RADS 2	Mammo: calcification, diffuse, central tail (benign) Eco: negative		0
03_048	MILANO	SX	40	4	0	1	BI-RADS 2	Mammo: calcification, diffuse, central tail (benign) Eco: negative		0
03_049	MILANO	DX	44	3	1	0	BI-RADS 1	Mammo: negative Eco: negative		1
03_049	MILANO	SX	44	3	1	0	BI-RADS 1	Mammo: negative Eco: negative		1
03_050	MILANO	DX	44	3	0	0	BI-RADS 1	Mammo: negative Eco: negative		0
03_050	MILANO	SX	44	3	0	1	BI-RADS 6	Mammo: calcification, amorphous, regional, upper outer, moderate suspicious for malignancy Eco: mass, indistinct, hypoechoic, no posterior features, upper outer, suspicious	Carcinoma duttale in situ, grado nucleare intermedio (G2), pattern architetturale prevalente cribriforme (categoria B5 sec.	1

									European Guidelines)	
03_051	MILANO	DX	52	4	0	0	BI-RADS 1	Eco: negative		0
03_051	MILANO	SX	52	4	0	1	BI-RADS 6	Eco: mass, oval, indistinct, hypoechoic, no posterior features , upper inner, known biopsy proven malignancy	Invasive Ductal Carcinoma, G2)	0
03_052	MILANO	DX	26	4	1	0	BI-RADS 1	Eco: negative		1
03_052	MILANO	SX	26	4	0	0	BI-RADS 1	Eco: negative		1
03_053	MILANO	DX	27	4	0	0	BI-RADS 1	Eco: negative		0
03_053	MILANO	SX	27	4	0	0	BI-RADS 1	Eco: negative		0
03_054	MILANO	DX	46	4	0	1	BI-RADS 2	Eco. mass, round, microlobulated, hypoechoic, shadowing, upper inner, low suspicious for malignancy	lesione con pattern nodulare caratterizzata da iperplasia duttale tipica ed adenosi cui si associa iperplasia dello stroma. Complessivamente il reperto è riferibile ad una lesione fibroepiteliale benigna (categoria B2 sec. European Guidelines)	1
03_054	MILANO	SX	46	4	0	0	BI-RADS 1	Eco: negative		0
03_055	MILANO	DX	59	1	1	0	BI-RADS 1	Eco: negative		1
03_055	MILANO	SX	59	1	0	1	BI-RADS 6	Eco: mass, irregular, indistinct, hypoechoic, shadowing, axillary tail, known biopsy proven malignancy	Invasive Lobular Carcinoma, G2	1
03_056	MILANO	DX	76	2	1	0	BI-RADS 1	Mammo: negative Eco: negative		1
03_056	MILANO	SX	76	2	0	1	BI-RADS 6	Mammo: mass, oval, indistinct, equal density, upper inner, known biopsy proven malignancy Eco: mass, oval, indistinct, hypoechoic, no posterior features, upper inner, known biopsy proven malignancy	Invasive Ductal Carcinoma, G3	1
03_057	MILANO	DX	50	1	0	0	BI-RADS 1	Eco: negative		1
03_057	MILANO	SX	50	1	1	1	BI-RADS 6	Eco: mass, irregular, indistinct, heterogeneous, shadowing, upper outer, known biopsy proven malignancy	Invasive Ductal Carcinoma, G2	0
03_058	MILANO	DX	54	4	1	0	BI-RADS 1	Eco: negative		1
03_058	MILANO	SX	54	4	0	0	BI-RADS 1	Eco: negative		1
03_059	MILANO	DX	57	1	0	1	BI-RADS 2	Eco: mass, round, circumscribed, hypoechoic, no posterior feaatures, lower outer, moderate suspicious for malignancy	parenchima mammario, con fibrosi stromale, focali aspetti di	1

									modificazioni a cellule colonnari, ad architettura fibroadenomatoide e con associata iperplasia pseugoangiomatosa stromale. (categoria B2 sec European Guidelines)	
03_059	MILANO	SX	57	1	0	1	BI-RADS 2	Eco: mass, oval, circumscribed, heterogeneous, no posterior features, upper outer (benign)		1
03_060	MILANO	DX	37	4	0	1	BI-RADS 2	Eco: mass, oval, circumscribed, hypechoic, no posterior features, lower inner (benign)		1
03_060	MILANO	SX	37	4	0	1	BI-RADS 2	Eco: mass, oval, circumscribed, hypechoic, no posterior features, lower inner (benign)		1
03_061	MILANO	DX	32	4	0	0	BI-RADS 1	Eco: negative		0
03_061	MILANO	SX	32	4	1	0	BI-RADS 1	Eco: negative		1
03_062	MILANO	DX	50	2	1	1	BI-RADS 6	Mammo: architectural distortions, upper outer, suspicious	Carcinoma infiltrante (categoria B5b sec. European Guidelines). Nel materiale in esame, le caratteristiche morfofenotipiche favoriscono maggiormente l'istotipo lobulare, grado G2 sec Nottingham Score.	1
03_062	MILANO	SX	50	2	0	1	BI-RADS 6	Mammo: architectural distortions, upper outer, suspicious	Carcinoma infiltrante (categoria B5b sec. European Guidelines). Nel materiale in esame, le caratteristiche morfofenotipiche favoriscono maggiormente l'istotipo lobulare, grado G2 sec	1

									Nottingham Score.	
03_063	MILANO	DX	28	4	1	0	BI-RADS 1	Eco: negative		1
03_063	MILANO	SX	28	4	1	0	BI-RADS 1	Eco: negative		1
03_064	MILANO	DX	31	4	0	0	BI-RADS 1	Eco: negative		0
03_064	MILANO	SX	31	4	0	0	BI-RADS 1	Eco: negative		0

Supporting information S1B Table: methods and features for Toledo

	method1						method2						method3						method4						method5						method6							
Threshold	1.85392049	1.85392	0.1267905	0.2819	0.9409446		1.9668103	1.9668103	0.1707615	0.3311456	1.0169718		2.1713449	0.2111719	0.3715834	0.3062202	1.0856392		2.1931068	2.2475867	2.1931068	2.19311	0.2557893		2.3920414	2.4227407	2.4287103	2.39204	2.39204		1.981961	2.0232131	0.041099	1.981961	1.981961			
N_PATIENT' (dx=right; sx=left)	M2MEA_i'	MAX_n'	VAR_P'	MAD0_p'	VAR_r'	number of S=1 occurrence	M2MEA_i'	MAX_n'	VAR_p'	MAD0_p'	MAD0_r'	number of S=1 occurrence	ROS1_i'	VAR_p'	MAD0_p'	MAD1_p'	MAD0_r'	number of S=1 occurrence	M2MEA_i'	ROS1_i'	MAX_n'	MAX_p'	VAR_p'	number of S=1 occurrence	M2MEA_i'	ROS1_i'	ROS2_i'	MAX_n'	MAX_p'	number of S=1 occurrence	M2MEA_i'	ROS1_i'	MIN_n'	MAX_n'	MAX_p'	number of S=1 occurrence	number of statements verified	MammoWave rule-of-thumb output
01_016 dx	1.7739	1.7739	0.1266	0.2827	1.1031	2	1.9299	1.9299	0.1631	0.3243	1.0592	1	2.1423	0.2091	0.3684	0.3046	1.0827	0	2.1971	2.2533	2.1971	2.1971	0.2462	4	2.4096	2.4464	2.3539	2.4096	2.4096	4	2.0580	2.1115	0.0481	2.0580	2.0580	4	3	WF
01_016 sx	1.8213	1.8213	0.1112	0.2498	0.9841	1	1.9201	1.9201	0.1515	0.2987	0.9969	0	2.0683	0.1897	0.3434	0.2524	1.0420	0	2.0834	2.1313	2.0834	2.0834	0.2241	0	2.2535	2.2847	2.2085	2.2535	2.2535	0	1.9364	1.9793	0.0438	1.9364	1.9364	0	0	NF
01_017 dx	2.1553	2.1553	0.1930	0.3617	1.2426	5	2.3340	2.3340	0.2565	0.4172	1.2213	5	2.5434	0.3101	0.4597	0.4097	1.2270	5	2.5981	2.6485	2.5981	2.5981	0.3631	5	2.7987	2.8290	3.0946	2.7987	2.7987	5	2.3164	2.3631	0.0342	2.3164	2.3164	5	6	WF
01_017 sx	2.7942	2.7942	0.2370	0.4031	1.9606	5	3.0387	3.0387	0.3670	0.4923	1.6230	5	3.3568	0.4950	0.5657	0.4757	1.7228	5	3.4940	3.5550	3.4940	3.4940	0.6039	5	3.8667	3.9051	4.5788	3.8667	3.8667	5	3.3528	3.4189	0.0273	3.3528	3.3528	5	6	WF
01_018 dx	1.9171	1.9171	0.1130	0.2701	0.7667	2	2.0029	2.0029	0.1556	0.3283	0.9885	2	2.1217	0.2114	0.3826	0.3394	1.0595	3	2.1551	2.1887	2.1551	2.1551	0.2520	0	2.3351	2.3563	2.4367	2.3351	2.3351	1	2.0981	2.1330	0.0307	2.0981	2.0981	4	2	NF
01_018 sx	2.5148	2.5148	0.2548	0.4119	2.0736	5	2.7898	2.7898	0.3451	0.4787	1.6425	5	3.1218	0.4302	0.5354	0.4292	1.6766	5	3.1882	3.2773	6.0000	3.1882	0.5097	5	3.4744	3.5304	3.8082	3.4744	3.4744	5	2.7341	2.8067	0.0402	2.7341	2.7341	5	6	WF
01_019 dx	1.9298	1.9298	0.1497	0.3066	1.0916	5	2.0502	2.0502	0.1867	0.3446	1.0645	5	2.2235	0.2352	0.3875	0.3280	1.1050	5	2.2668	2.3152	2.2668	2.2668	0.2738	5	2.4477	2.4791	2.3860	2.4477	2.4477	4	2.0182	2.0574	0.0370	2.0182	2.0182	4	6	WF
01_019 sx	2.0929	2.0929	0.0791	0.2129	0.6013	2	2.1049	2.1049	0.1110	0.2564	0.7864	2	2.1823	0.1400	0.2994	0.2444	0.8359	1	2.1350	2.1828	2.1350	2.1350	0.1958	0	2.1737	2.2008	2.1915	2.1737	2.1737	0	2.0683	2.1127	0.0399	2.0683	2.0683	4	1	NF
01_020 dx	1.9105	1.9105	0.0987	0.2521	0.5447	2	2.0547	2.0547	0.1887	0.3459	0.9333	4	2.2525	0.2283	0.3856	0.3191	0.9975	4	2.3351	2.3764	2.3351	2.3351	0.2916	5	2.5913	2.6187	2.8014	2.5913	2.5913	5	2.0989	2.1360	0.0327	2.0989	2.0989	5	4	WF
01_020 sx	1.9950	1.9950	0.1204	0.2685	0.7757	2	2.1222	2.1222	0.1827	0.3347	1.0137	4	2.2861	0.2234	0.3794	0.3122	1.0786	4	2.3205	2.3644	2.3205	2.3205	0.2743	5	2.4784	2.5068	2.5658	2.4784	2.4784	5	2.0190	2.0608	0.0395	2.0190	2.0190	5	4	WF
01_021 dx	2.1834	2.1834	0.1247	0.3029	1.0846	4	2.1091	2.1091	0.1539	0.3399	1.1225	4	2.1999	0.2250	0.3989	0.3840	1.1758	5	2.1984	2.2436	2.1984	2.1984	0.2734	4	2.4198	2.4497	2.5175	2.4198	2.4198	5	2.0953	2.1373	0.0370	2.0953	2.0953	5	4	WF
01_022 dx	2.4012	2.4012	0.1983	0.3670	1.2393	5	2.5264	2.5264	0.2773	0.4283	1.3031	5	2.7402	0.3350	0.4686	0.4001	1.3321	5	2.7877	2.8449	2.7877	2.7877	0.3861	5	2.9873	3.0222	3.3053	2.9873	2.9873	5	2.6694	2.7293	0.0346	2.6694	2.6694	5	6	WF
01_023 dx	2.0939	2.0939	0.1353	0.2977	0.9972	5	2.2081	2.2081	0.1968	0.3552	1.0947	5	2.3565	0.2391	0.3968	0.3468	1.0994	5	2.3730	2.4187	2.3730	2.3730	0.2866	5	2.5073	2.5368	2.6343	2.5073	2.5073	5	2.0991	2.1393	0.0353	2.0991	2.0991	5	6	WF
01_023 sx	2.3147	2.3147	0.2000	0.3794	1.3993	5	2.4439	2.4439	0.2571	0.4378	1.3706	5	2.6450	0.3492	0.5083	0.5037	1.4688	5	2.6880	2.7490	2.6880	2.6880	0.4233	5	2.9192	2.9586	3.1652	2.9192	2.9192	5	2.3553	2.4052	0.0355	2.3553	2.3553	5	6	WF
01_024 dx	2.3706	2.3706	0.2108	0.3713	1.5765	5	2.5269	2.5269	0.2903	0.4403	1.4250	5	2.7433	0.3464	0.4846	0.4271	1.4283	5	2.7911	2.8498	2.7911	2.7911	0.4127	5	3.0064	3.0430	3.1654	3.0064	3.0064	5	2.4078	2.4547	0.0323	2.4078	2.4078	5	6	WF
01_024 sx	2.7437	2.7437	0.2945	0.4340	2.3383	5	2.9672	2.9672	0.3809	0.4950	1.7429	5	3.2364	0.4636	0.5484	0.4495	1.8045	5	3.2889	3.3604	3.2889	3.2889	0.5432	5	3.5234	3.5667	3.9952	3.5234	3.5234	5	2.7480	2.8126	0.0357	2.7480	2.7480	5	6	WF
01_025 dx	2.4928	2.4928	0.2410	0.3975	1.8723	5	2.6996	2.6996	0.3046	0.4489	1.5334	5	2.9713	0.3749	0.5001	0.4298	1.5778	5	3.0266	3.1029	3.0266	3.0266	0.4443	5	3.2937	3.3426	3.7792	3.2937	3.2937	5	2.1471	2.2005	0.0445	2.1471	2.1471	4	6	WF
01_025 sx	1.8152	1.8152	0.1068	0.2462	0.9029	0	1.9590	1.9590	0.1465	0.2963	0.9724	0	2.1391	0.1937	0.3478	0.2860	1.0324	0	2.1852	2.2327	2.1852	2.1852	0.2312	0	2.3650	2.3953	2.3884	2.3650	2.3650	0	1.9857	2.0318	0.0447	1.9857	1.9857	4	1	NF
01_026 dx	2.1639	2.1639	0.1782	0.3323	1.3617	5	2.3232	2.3232	0.2270	0.3757	1.1999	5	2.5188	0.2790	0.4207	0.3513	1.2464	5	2.5673	2.6192	2.5673	2.5673	0.3510	5	2.7685	2.8016	2.9524	2.7685	2.7685	5	2.2011	2.2423	0.0332	2.2011	2.2011	5	6	WF
01_026 sx	2.0683	2.0683	0.0902	0.2502	0.6974	2	2.1928	2.1928	0.2234	0.3932	1.2102	5	2.3957	0.2774	0.4397	0.3937	1.2483	5	2.4445	2.4961	2.4445	2.4445	0.3180	5	2.6312	2.6633	2.6692	2.6312	2.6312	5	2.1162	2.1557	0.0341	2.1162	2.1162	5	5	WF
01_027 dx	2.4103	2.4103	0.2011	0.3640	1.3804	5	2.5758	2.5758	0.2838	0.4340	1.3779	5	2.7828	0.3442	0.4817	0.4121	1.4051	5	2.8273	2.8822	2.8273	2.8273	0.4120	5	3.0219	3.0553	3.2671	3.0219	3.0219	5	2.2085	2.2567	0.0384	2.2085	2.2085	5	6	WF
01_027 sx	2.7472	2.7472	0.2706	0.4245	2.0056	5	3.0399	3.0399	0.4147	0.5210	1.7146	5	3.3855	0.5116	0.5820	0.4753	1.8608	5	3.5078	3.5803	3.5078	3.5078	0.6121	5	3.8738	3.9202	4.4215	3.8738	3.8738	5	2.3028	2.3458	0.0319	2.3028	2.3028	5	6	WF
01_028 dx	2.0818	2.0818	0.1558	0.3081	0.9577	5	2.2840	2.2840	0.2106	0.3578	1.0556	5	2.5274	0.2631	0.4049	0.3265	1.1268	5	2.6103	2.6672	2.6103	2.6103	0.3336	5	2.8745	2.9122	2.9705	2.8745	2.8745	5	2.2337	2.2801	0.0362	2.2337	2.2337	5	6	WF
01_028 sx	1.9876	1.9876	0.1142	0.2650	0.9994	3	2.1245	2.1245	0.1873	0.3426	1.1701	5	2.3290	0.2330	0.3891	0.3401	1.1808	5	2.3573	2.4186	2.3573	2.3573	0.2869	5	2.5559	2.5940	2.6051	2.5559	2.5559	5	2.0406	2.0930	0.0480	2.0406	2.0406	4	5	WF
01_029 dx	1.8674	1.8674	0.0591	0.1930	0.4736	2	1.8086	1.8086	0.1444	0.2972	0.9643	0	1.9457	0.1741	0.3299	0.2201	0.9833	0	1.9736	2.0134	1.9736	1.9736	0.2278	0	2.1120	2.1375	2.0978	2.1120	2.1120	0	1.8989	1.9420	0.0458	1.8989	1.8989	0	0	NF
01_029 sx	2.1441	2.1441	0.0939	0.2549	0.9876	3	2.1762	2.1762	0.1371	0.3068	1.1379	3	2.3023	0.2095	0.3765	0.2757	1.2506	3	2.2834	2.3410	2.2834	2.2834	0.2725	5	2.4142	2.4487	2.4387	2.4142	2.4142	5	2.0987	2.1554	0.0490	2.0987	2.0987	4	4	WF

01_030 dx	2.2506	2.2506	0.2307	0.4029	1.7978	5	2.4546	2.4546	0.3023	0.4603	1.5262	5	2.7148	0.3711	0.5136	0.4788	1.5547	5	2.7758	2.8464	2.7758	2.7758	0.4342	5	3.0289	3.0743	3.4029	3.0289	3.0289	5	2.4659	2.5265	0.0397	2.4659	2.4659	5	6	WF
01_030 sx	1.7684	1.7684	0.1152	0.2604	1.0963	0	1.9367	1.9367	0.1571	0.3093	1.0567	1	2.1516	0.2037	0.3585	0.2940	1.0976	1	2.2243	2.2765	2.2243	2.2243	0.2509	4	2.4707	2.5056	2.5007	2.4707	2.4707	5	1.8635	1.9008	0.0415	1.8635	1.8635	0	2	NF
01_031 dx	2.5191	2.5191	0.2071	0.3926	1.5006	5	2.5915	2.5915	0.2848	0.4526	1.4263	5	2.7458	0.3501	0.4989	0.4705	1.4597	5	2.7682	2.8207	2.7682	2.7682	0.4284	5	2.9375	2.9702	3.3006	2.9375	2.9375	5	2.4954	2.5512	0.0360	2.4954	2.4954	5	6	WF
01_031 sx	2.9284	2.9284	0.2419	0.4117	2.0090	5	2.9130	2.9130	0.2837	0.4447	1.4822	5	3.0299	0.3498	0.4879	0.4430	1.5015	5	3.0157	3.0820	3.0157	3.0157	0.4447	5	3.2104	3.2506	3.6442	3.2104	3.2104	5	2.5186	2.5823	0.0402	2.5186	2.5186	5	6	WF
01_032 dx	2.5565	2.5565	0.2695	0.4211	2.2972	5	2.7817	2.7817	0.3405	0.4792	1.6355	5	3.0492	0.4207	0.5344	0.4459	1.6597	5	3.1168	3.1823	3.1168	3.1168	0.4953	5	3.3631	3.4046	3.8423	3.3631	3.3631	5	2.4524	2.5016	0.0328	2.4524	2.4524	5	6	WF
01_032 sx	2.6167	2.6167	0.2607	0.4138	2.0917	5	2.7846	2.7846	0.3409	0.4810	1.5429	5	3.0022	0.4276	0.5425	0.4932	1.5925	5	3.0457	3.1071	3.0457	3.0457	0.5015	5	3.2541	3.2938	3.8386	3.2541	3.2541	5	2.2166	2.2560	0.0314	2.2166	2.2166	5	6	WF
01_033 dx	1.9533	1.9533	0.0932	0.2599	0.7001	2	1.9701	1.9701	0.1332	0.3104	0.9436	2	2.0314	0.1644	0.3446	0.3309	0.9509	1	1.9977	2.0337	1.9977	1.9977	0.2122	0	2.0774	2.0989	2.1275	2.0774	2.0774	0	1.9785	2.0229	0.0434	1.9785	1.9785	0	0	NF
01_033 sx	2.2347	2.2347	0.1366	0.3057	0.8598	4	2.3829	2.3829	0.2199	0.3780	1.1204	5	2.5860	0.2910	0.4307	0.3569	1.1916	5	2.6335	2.6887	2.6335	2.6335	0.3369	5	2.8359	2.8702	3.1313	2.8359	2.8359	5	2.1209	2.1651	0.0380	2.1209	2.1209	5	5	WF
01_034 dx	2.3904	2.3904	0.1588	0.3343	1.6194	5	2.4010	2.4010	0.2091	0.3841	1.3522	5	2.5734	0.3245	0.4629	0.4047	1.4695	5	2.6132	2.6726	2.6132	2.6132	0.3724	5	2.8084	2.8446	2.9351	2.8084	2.8084	5	2.1442	2.1895	0.0381	2.1442	2.1442	5	6	WF
01_034 sx	2.1987	2.1987	0.2157	0.3949	1.4314	5	2.3975	2.3975	0.2674	0.4319	1.3172	5	2.6293	0.3232	0.4715	0.4133	1.3275	5	2.7041	2.7569	2.7041	2.7041	0.3921	5	2.9444	2.9773	3.0981	2.9444	2.9444	5	2.0517	2.0889	0.0342	2.0517	2.0517	5	6	WF
01_035 dx	1.9385	1.9385	0.1055	0.2656	0.7427	2	2.0073	2.0073	0.1729	0.3370	1.0104	4	2.1519	0.2164	0.3796	0.3188	1.0224	3	2.1798	2.2196	2.1798	2.1798	0.2475	0	2.4435	2.4711	2.5806	2.4435	2.4435	5	2.1157	2.1558	0.0347	2.1157	2.1157	5	3	WF
01_035 sx	2.1703	2.1703	0.1706	0.3321	1.1045	5	2.3646	2.3646	0.2570	0.4141	1.1880	5	2.5809	0.3095	0.4569	0.3970	1.1894	5	2.6542	2.6982	2.6542	2.6542	0.3608	5	2.8607	2.8876	3.1785	2.8607	2.8607	5	2.4182	2.4679	0.0339	2.4182	2.4182	5	6	WF
01_036 dx	1.8254	1.8254	0.1183	0.2703	0.7685	0	1.9813	1.9813	0.1626	0.3162	0.9326	2	2.1693	0.1962	0.3474	0.2792	0.9508	0	2.2265	2.2711	2.2265	2.2265	0.2352	4	2.4313	2.4603	2.4192	2.4313	2.4313	4	2.2235	2.2692	0.0360	2.2235	2.2235	5	3	WF
01_036 sx	1.8352	1.8352	0.1254	0.2820	0.8019	1	2.0156	2.0156	0.1684	0.3229	0.9489	2	2.2252	0.2237	0.3773	0.2914	1.0503	3	2.3001	2.3465	2.3001	2.3001	0.2619	5	2.5156	2.5459	2.6005	2.5156	2.5156	5	2.1443	2.1870	0.0360	2.1443	2.1443	5	4	WF
01_037 dx	1.9595	1.9595	0.1463	0.3096	1.0850	5	2.1160	2.1160	0.1999	0.3659	1.1673	5	2.2956	0.2473	0.4087	0.3528	1.1849	5	2.3518	2.3923	2.3518	2.3518	0.2941	5	2.5298	2.5559	2.6080	2.5298	2.5298	5	2.1275	2.1611	0.0289	2.1275	2.1275	5	6	WF
01_037 sx	2.2233	2.2233	0.1816	0.3598	1.4007	5	2.3063	2.3063	0.2458	0.4174	1.3804	5	2.5286	0.3119	0.4538	0.3899	1.4040	5	2.5943	2.6529	2.5943	2.5943	0.3679	5	2.8290	2.8663	3.0777	2.8290	2.8290	5	2.2942	2.3445	0.0375	2.2942	2.2942	5	6	WF
01_038 dx	2.4199	2.4199	0.2337	0.3907	1.2519	5	2.6462	2.6462	0.3080	0.4458	1.2953	5	2.8992	0.3712	0.4894	0.3969	1.3257	5	2.9972	3.0458	2.9972	2.9972	0.4332	5	3.2582	3.2882	3.7752	3.2582	3.2582	5	2.5040	2.5483	0.0286	2.5040	2.5040	5	6	WF
01_038 sx	2.7205	2.7205	0.3064	0.4628	1.3221	5	2.9476	2.9476	0.4159	0.5360	1.3689	5	3.2104	0.5093	0.5918	0.5225	1.4424	5	3.3094	3.3629	3.3094	3.3094	0.5874	5	3.5882	3.6215	4.6482	3.5882	3.5882	5	2.3424	2.3865	0.0318	2.3424	2.3424	5	6	WF
01_039 dx	1.8226	1.8226	0.0738	0.2102	0.5530	0	1.8436	1.8436	0.0921	0.2443	0.7640	0	1.9039	0.1818	0.3481	0.2765	1.0337	0	1.9556	1.9911	1.9556	1.9556	0.2138	0	2.1239	2.1473	2.0489	2.1239	2.1239	0	1.9838	2.0258	0.0409	1.9838	1.9838	5	1	NF
01_039 sx	1.6578	1.6578	0.1186	0.2754	0.8317	0	1.7939	1.7939	0.1489	0.3013	0.9022	0	1.9688	0.1793	0.3325	0.2460	0.9366	0	2.0257	2.0682	2.0257	2.0257	0.2114	0	2.2128	2.2408	2.1877	2.2128	2.2128	0	1.8934	1.9333	0.0427	1.8934	1.8934	0	0	NF
01_040 dx	1.9102	1.9102	0.1153	0.2771	0.8962	2	2.0469	2.0469	0.1833	0.3477	1.1009	5	2.2739	0.2427	0.3943	0.3231	1.1319	5	2.3796	2.4280	2.3796	2.3796	0.2921	5	2.6633	2.6957	2.8468	2.6633	2.6633	5	2.1384	2.1795	0.0348	2.1384	2.1384	5	5	WF
01_040 sx	2.0603	2.0603	0.1361	0.2914	1.0814	5	2.1766	2.1766	0.1739	0.3376	1.1142	5	2.3373	0.2400	0.4037	0.3531	1.2225	5	2.3725	2.4234	2.3725	2.3725	0.2934	5	2.5889	2.6212	2.6098	2.5889	2.5889	5	2.2473	2.2987	0.0396	2.2473	2.2473	5	6	WF
01_041 dx	2.0058	2.0058	0.1697	0.3257	1.2973	5	2.1488	2.1488	0.1984	0.3474	1.1158	5	2.3239	0.2393	0.3865	0.3141	1.1314	5	2.3657	2.4116	2.3657	2.3657	0.2811	5	2.5404	2.5699	2.5260	2.5404	2.5404	5	1.9624	2.0005	0.0381	1.9624	1.9624	1	5	WF
01_041 sx	2.0057	2.0057	0.1197	0.2903	0.7200	3	2.1651	2.1651	0.2218	0.3883	1.1261	5	2.3794	0.2710	0.4309	0.3695	1.1616	5	2.4494	2.4956	2.4494	2.4494	0.3201	5	2.6594	2.6878	2.8554	2.6594	2.6594	5	2.2821	2.3274	0.0341	2.2821	2.2821	5	5	WF
01_042 dx	1.8107	1.8107	0.1186	0.2791	0.7869	0	1.9042	1.9042	0.1590	0.3212	0.9580	0	2.0410	0.1898	0.3550	0.3013	0.9628	0	2.0886	2.1247	2.0886	2.0886	0.2127	0	2.2628	2.2860	2.3096	2.2628	2.2628	0	2.0389	2.0769	0.0352	2.0389	2.0389	5	1	NF
01_042 sx	2.3130	2.3130	0.1776	0.3246	1.2884	5	2.4646	2.4646	0.2239	0.3704	1.1832	5	2.6455	0.3000	0.4335	0.3471	1.2903	5	2.6899	2.7371	2.6899	2.6899	0.3571	5	2.8689	2.8986	3.1386	2.8689	2.8689	5	1.9803	2.0193	0.0383	1.9803	1.9803	1	5	WF
01_043 dx	2.8495	2.8495	0.3550	0.4953	1.4617	5	3.2198	3.2198	0.4974	0.5860	1.4234	5	3.5750	0.6247	0.6557	0.5647	1.5325	5	3.7770	3.8190	3.7770	3.7770	0.7470	5	4.1892	4.2163	5.6300	4.1892	4.1892	5	2.7654	2.8199	0.0299	2.7654	2.7654	5	6	WF
01_043 sx	2.5338	2.5338	0.2764	0.4346	1.1928	5	2.7987	2.7987	0.3844	0.5119	1.2561	5	3.0680	0.4725	0.5684	0.5073	1.3186	5	3.2020	3.2418	3.2020	3.2020	0.5709	5	3.5069	3.5319	4.3323	3.5069	3.5069	5	2.7734	2.8118	0.0212	2.7734	2.7734	5	6	WF
01_044 dx	1.8760	1.8760	0.1228	0.2806	1.0015	3	1.9721	1.9721	0.1732	0.3354	1.1022	5	2.1206	0.2067	0.3703	0.2997	1.0951	1	2.1418	2.1879	2.1418	2.1418	0.2514	0	2.2960	2.3258	2.3098	2.2960	2.2960	0	1.9866	2.0320	0.0440	1.9866	1.9866	4	2	NF
01_044 sx	2.7716	2.7716	0.2233	0.3827	1.5842	5	2.9257	2.9257	0.3245	0.4574	1.5231	5	3.1609	0.4461	0.5395	0.4609	1.6421	5	3.1942	3.2679	3.1942	3.1942	0.5093	5	3.3914	3.4367	3.9086	3.3914	3.3914	5	2.7861	2.8570	0.0382	2.7861	2.7861	5	6	WF
01_045 dx	2.7300	2.7300	0.2794	0.4246	2.2465	5	2.9817	2.9817	0.3727	0.4927	1.7309	5	3.2844	0.4632	0.5516	0.4458	1.8281	5	3.3514	3.4287	3.3514	3.3514	0.5505	5	3.6239	3.6704	3.9754	3.6239	3.6239	5	2.5096	2.5618	0.0334	2.5096	2.5096	5	6	WF
01_045 sx	2.2051	2.2051	0.1934	0.3515	1.3528	5	2.3948	2.3948	0.2487	0.3934	1.2951	5	2.6280	0.2986	0.4327	0.3493	1.3241	5	2.6890	2.7474	2.6890	2.6890	0.3535	5	2.9193	2.9560	3.0741	2.9193	2.9193	5	2.3641	2.4101	0.0326	2.3641	2.3641	5	6	WF
01_046 dx	2.7216	2.7216	0.3111	0.4635	1.7854	5	2.9771	2.9771	0.4069	0.5284	1.5177	5	3.2342	0.4947	0.5818	0.4842	1.5679	5	3.3410	3.3848	3.3410	3.3410	0.5770	5	3.6084	3.6348	4.2715	3.6084	3.6084	5	2.6265	2.6622	0.0215	2.6265	2.6265	5	6	WF
01_046 sx	2.7550	2.7550	0.3588	0.4988	1.9120	5	3.0602	3.0602	0.4754	0.5748	1.6259	5	3.3957	0.5839	0.6370	0.5141	1.7093	5	3.5341	3.5985	3.5341	3.5341	0.6965	5	3.9079	3.9490	4.8819	3.9079	3.9079	5	2.8760	2.9347	0.0303	2.8760	2.8760	5	6	WF

01_047 dx	2.5466	2.5466	0.2390	0.4134	0.9820	5	2.6902	2.6902	0.3702	0.5040	1.2013	5	2.8933	0.4239	0.5432	0.4953	1.2297	5	2.9423	2.9921	2.9423	2.9423	0.4734	5	3.1327	3.1627	3.9667	3.1327	3.1327	5	2.1945	2.2324	0.0308	2.1945	2.1945	4	6	WF
01_047 sx	2.6275	2.6275	0.2417	0.3991	1.4050	5	2.7766	2.7766	0.3308	0.4650	1.3210	5	2.9800	0.3929	0.5097	0.4347	1.3511	5	3.0290	3.0808	3.0290	3.0290	0.4551	5	3.2424	3.2747	3.8936	3.2424	3.2424	5	2.4180	2.4670	0.0334	2.4180	2.4180	4	6	WF
01_048 dx	2.6815	2.6815	0.3167	0.4596	2.3273	5	2.9422	2.9422	0.4033	0.5203	1.7208	5	3.2695	0.5103	0.5845	0.4731	1.8208	5	3.3718	3.4472	3.3718	3.3718	0.5993	5	3.7082	3.7544	4.1986	3.7082	3.7082	5	2.4419	2.5007	0.0392	2.4419	2.4419	4	6	WF
01_048 sx	2.5192	2.5192	0.2379	0.3854	1.9855	5	2.7345	2.7345	0.3184	0.4477	1.5096	5	3.0008	0.3907	0.4990	0.4017	1.5596	5	3.0611	3.1308	3.0611	3.0611	0.4669	5	3.3187	3.3621	3.6440	3.3187	3.3187	5	2.2476	2.2973	0.0383	2.2476	2.2476	4	6	WF
01_049 dx	2.4503	2.4503	0.2040	0.3666	1.3631	5	2.6120	2.6120	0.2589	0.4139	1.2021	5	2.8084	0.3386	0.4694	0.3969	1.2716	5	2.8633	2.9084	2.8633	2.8633	0.3955	5	3.0582	3.0852	3.5000	3.0582	3.0582	5	2.2332	2.2701	0.0290	2.2332	2.2332	5	6	WF
01_049 sx	2.8807	2.8807	0.3708	0.5059	2.8602	5	3.1373	3.1373	0.4793	0.5752	1.8704	5	3.4187	0.5760	0.6310	0.5504	1.9280	5	3.4972	3.5606	3.4972	3.4972	0.6637	5	3.7539	3.7924	4.4510	3.7539	3.7539	5	2.7265	2.7839	0.0322	2.7265	2.7265	5	6	WF
01_050 dx	2.2405	2.2405	0.1105	0.2796	0.8418	2	2.2560	2.2560	0.1676	0.3455	1.0827	4	2.4267	0.2930	0.4428	0.3937	1.2454	5	2.4818	2.5271	2.4818	2.4818	0.3625	5	2.6885	2.7167	2.9228	2.6885	2.6885	5	2.0559	2.0942	0.0351	2.0559	2.0559	5	4	WF
01_050 sx	1.8978	1.8978	0.1612	0.3227	1.1101	5	2.0508	2.0508	0.1959	0.3539	1.0689	5	2.2425	0.2356	0.3894	0.3096	1.0967	5	2.2994	2.3466	2.2994	2.2994	0.2731	5	2.5015	2.5317	2.4965	2.5015	2.5015	5	2.0289	2.0679	0.0365	2.0289	2.0289	5	6	WF
01_051 dx	2.0621	2.0621	0.1107	0.2692	0.7220	2	2.1068	2.1068	0.1451	0.3179	0.9470	2	2.2092	0.2098	0.3804	0.3387	1.0371	3	2.2141	2.2562	2.2141	2.2141	0.2554	4	2.3455	2.3720	2.4626	2.3455	2.3455	1	2.4645	2.5239	0.0389	2.4645	2.4645	5	3	WF
01_051 sx	1.6081	1.6081	0.1041	0.2538	0.9015	0	1.7040	1.7040	0.1251	0.2771	0.8828	0	1.8647	0.1550	0.3133	0.2481	0.9063	0	1.9015	1.9462	1.9015	1.9015	0.1880	0	2.0613	2.0902	1.9915	2.0613	2.0613	0	1.8514	1.8989	0.0529	1.8514	1.8514	0	0	NF
01_052 dx	2.0019	2.0019	0.1349	0.2904	0.8343	4	2.1000	2.1000	0.1609	0.3127	0.9406	2	2.2350	0.2173	0.3667	0.2978	1.0341	2	2.2609	2.2988	2.2609	2.2609	0.2617	5	2.3914	2.4145	2.4186	2.3914	2.3914	0	2.1070	2.1464	0.0344	2.1070	2.1070	5	2	NF
01_052 sx	2.1702	2.1702	0.1204	0.2969	0.9434	4	2.3151	2.3151	0.2690	0.4292	1.3423	5	2.5328	0.3150	0.4680	0.4089	1.3618	5	2.5995	2.6502	2.5995	2.5995	0.3746	5	2.8363	2.8685	2.9733	2.8363	2.8363	5	1.9835	2.0170	0.0329	1.9835	1.9835	4	5	WF
01_053 dx	1.9882	1.9882	0.1455	0.3026	0.9439	5	2.1223	2.1223	0.1958	0.3543	1.0527	5	2.2967	0.2375	0.3936	0.3311	1.0615	4	2.3298	2.3791	2.3298	2.3298	0.2835	5	2.5058	2.5366	2.6913	2.5058	2.5058	5	2.1942	2.2411	0.0378	2.1942	2.1942	5	6	WF
01_053 sx	1.7657	1.7657	0.1205	0.2735	0.8301	0	1.9579	1.9579	0.1575	0.3132	0.9462	0	2.1734	0.2020	0.3591	0.2959	1.0078	1	2.2550	2.2996	2.2550	2.2550	0.2465	4	2.4827	2.5114	2.5690	2.4827	2.4827	5	2.2163	2.2633	0.0372	2.2163	2.2163	5	3	WF
01_054 dx	2.5914	2.5914	0.2249	0.3998	1.9517	5	2.7490	2.7490	0.3072	0.4586	1.5185	5	2.9620	0.3864	0.5094	0.4452	1.5726	5	3.0115	3.0715	3.0115	3.0115	0.4585	5	3.2179	3.2542	3.5739	3.2179	3.2179	5	2.1545	2.1899	0.0297	2.1545	2.1545	5	6	WF
01_054 sx	1.9704	1.9704	0.1093	0.2671	0.7674	2	2.0564	2.0564	0.1482	0.3129	0.9717	2	2.2177	0.1927	0.3600	0.2625	1.0324	1	2.2485	2.3000	2.2485	2.2485	0.2584	5	2.4271	2.4599	2.5522	2.4271	2.4271	5	2.2138	2.2688	0.0434	2.2138	2.2138	4	3	WF
01_055 dx	2.5164	2.5164	0.1713	0.3459	0.8973	4	2.5851	2.5851	0.2424	0.4104	1.1679	5	2.7381	0.3303	0.4764	0.4339	1.2508	5	2.7871	2.8424	2.7871	2.7871	0.4281	5	3.0546	3.0897	3.9135	3.0546	3.0546	5	2.5517	2.6048	0.0331	2.5517	2.5517	5	6	WF
01_055 sx	2.5235	2.5235	0.2708	0.4334	1.5364	5	2.6559	2.6559	0.3364	0.4858	1.3815	5	2.8209	0.3995	0.5291	0.4916	1.4186	5	2.8376	2.8878	2.8376	2.8376	0.4620	5	2.9656	2.9959	3.6173	2.9656	2.9656	5	2.3565	2.4009	0.0317	2.3565	2.3565	5	6	WF
01_056 dx	2.2953	2.2953	0.2161	0.3821	1.1435	5	2.4975	2.4975	0.2908	0.4449	1.2596	5	2.7354	0.3571	0.4940	0.4014	1.3099	5	2.8195	2.8719	2.8195	2.8195	0.4250	5	3.0730	3.1057	3.6130	3.0730	3.0730	5	2.1032	2.1415	0.0335	2.1032	2.1032	5	6	WF
01_056 sx	2.1527	2.1527	0.1821	0.3405	1.2292	5	2.3897	2.3897	0.2537	0.4034	1.2614	5	2.6597	0.3261	0.4607	0.3926	1.3338	5	2.7700	2.8240	2.7700	2.7700	0.3982	5	3.0761	3.1111	3.3179	3.0761	3.0761	5	2.1793	2.2212	0.0343	2.1793	2.1793	5	6	WF
01_057 dx	2.4884	2.4884	0.2009	0.3509	0.8989	4	2.6880	2.6880	0.3468	0.4799	1.2702	5	2.9552	0.4377	0.5429	0.4701	1.3394	5	3.0410	3.1011	3.0410	3.0410	0.5117	5	3.3067	3.3453	4.1407	3.3067	3.3067	5	2.3545	2.4082	0.0382	2.3545	2.3545	5	5	WF
01_057 sx	2.4646	2.4646	0.2589	0.4352	1.2691	5	2.6066	2.6066	0.3263	0.4943	1.3476	5	2.8186	0.3964	0.5239	0.4400	1.1887	5	2.9373	2.9875	2.9373	2.9373	0.4762	5	3.2463	3.2791	4.1345	3.2463	3.2463	5	2.6208	2.6771	0.0336	2.6208	2.6208	5	6	WF
01_058 dx	2.2534	2.2534	0.1180	0.2774	0.7794	2	2.2948	2.2948	0.1532	0.3273	0.9998	2	2.3941	0.1983	0.3774	0.3601	1.0531	3	2.3381	2.3915	2.3381	2.3381	0.2637	5	2.4544	2.4860	2.6870	2.4544	2.4544	5	1.9916	2.0348	0.0417	1.9916	1.9916	4	4	WF
01_058 sx	2.2409	2.2409	0.1917	0.3577	1.4263	5	2.4053	2.4053	0.2461	0.4131	1.2527	5	2.6101	0.2964	0.4594	0.4219	1.2850	5	2.6380	2.6970	2.6380	2.6380	0.3645	5	2.8016	2.8378	3.1678	2.8016	2.8016	5	2.4156	2.4692	0.0365	2.4156	2.4156	5	6	WF
01_059 dx	2.6716	2.6716	0.3056	0.4504	1.5136	5	2.9653	2.9653	0.4261	0.5389	1.5268	5	3.2991	0.5329	0.6060	0.5163	1.6309	5	3.4233	3.4928	3.4233	3.4233	0.6381	5	3.7846	3.8279	4.7522	3.7846	3.7846	5	2.8707	2.9282	0.0298	2.8707	2.8707	5	6	WF
01_059 sx	1.9351	1.9351	0.1642	0.3334	1.2808	5	2.1007	2.1007	0.2120	0.3797	1.2307	5	2.3011	0.2607	0.4178	0.3579	1.2455	5	2.3730	2.4240	2.3730	2.3730	0.3081	5	2.6132	2.6469	2.7391	2.6132	2.6132	5	2.1623	2.2034	0.0342	2.1623	2.1623	5	6	WF
01_060 dx	1.8643	1.8643	0.1000	0.2557	0.5923	2	1.9374	1.9374	0.1358	0.3046	0.8511	0	2.0917	0.2379	0.4094	0.3738	1.1082	4	2.1689	2.1991	2.1689	2.1689	0.2737	1	2.3600	2.3794	2.4966	2.3600	2.3600	1	2.0860	2.1174	0.0281	2.0860	2.0860	5	2	NF
01_060 sx	2.5990	2.5990	0.2822	0.4416	1.7541	5	2.8087	2.8087	0.3813	0.5053	1.5311	5	3.0656	0.4582	0.5519	0.4688	1.5742	5	3.1459	3.2031	3.1459	3.1459	0.5241	5	3.3990	3.4341	4.0249	3.3990	3.3990	5	2.4011	2.4390	0.0263	2.4011	2.4011	5	6	WF
01_061 dx	2.1562	2.1562	0.1429	0.3262	1.0034	5	2.2261	2.2261	0.1949	0.3818	1.1979	5	2.3852	0.2637	0.4326	0.4018	1.2420	5	2.4094	2.4641	2.4094	2.4094	0.3159	5	2.6031	2.6372	2.7907	2.6031	2.6031	5	2.5328	2.5924	0.0374	2.5328	2.5328	5	6	WF
01_061 sx	2.1217	2.1217	0.1433	0.3046	1.3790	5	2.3124	2.3124	0.2075	0.3651	1.2685	5	2.5454	0.2553	0.4070	0.3095	1.2659	5	2.6060	2.6662	2.6060	2.6060	0.3130	5	2.8323	2.8710	2.8284	2.8323	2.8323	5	2.1113	2.1542	0.0371	2.1113	2.1113	5	6	WF
01_062 dx	2.4700	2.4700	0.2832	0.4467	1.4185	5	2.6776	2.6776	0.3672	0.5050	1.3886	5	2.9140	0.4460	0.5551	0.4699	1.4523	5	3.0097	3.0558	3.0097	3.0097	0.5214	5	3.2703	3.2986	3.6100	3.2703	3.2703	5	2.7932	2.8349	0.0227	2.7932	2.7932	5	6	WF
01_062 sx	2.6589	2.6589	0.2063	0.3839	0.9046	4	2.7798	2.7798	0.3130	0.4660	1.1558	5	2.9707	0.4063	0.5262	0.4676	1.2596	5	3.0468	3.0879	3.0468	3.0468	0.4829	5	3.2585	3.2836	4.1620	3.2585	3.2585	5	2.1581	2.1912	0.0278	2.1581	2.1581	5	5	WF
01_063 dx	2.5761	2.5761	0.3239	0.4755	1.7744	5	2.8092	2.8092	0.4078	0.5333	1.4797	5	3.0800	0.4709	0.5713	0.4824	1.5058	5	3.1681	3.2287	3.1681	3.1681	0.5393	5	3.4432	3.4814	4.2225	3.4432	3.4432	5	2.6532	2.7032	0.0293	2.6532	2.6532	5	6	WF
01_063 sx	2.4584	2.4584	0.2690	0.4380	1.0676	5	2.5280	2.5280	0.3061	0.4678	1.0898	5	2.6660	0.3846	0.5124	0.4685	1.1491	5	2.7301	2.7661	2.7301	2.7301	0.3858	5	2.9575	2.9807	3.8277	2.9575	2.9575	5	2.2316	2.2656	0.0268	2.2316	2.2316	5	6	WF

01_064 dx	1.9652	1.9652	0.1347	0.2742	1.0799	4	2.1619	2.1619	0.1881	0.3300	1.0739	4	2.3998	0.2505	0.3861	0.2997	1.1485	4	2.5022	2.5493	2.5022	2.5022	0.3030	5	2.7838	2.8139	2.8166	2.7838	2.7838	5	2.0708	2.1131	0.0380	2.0708	2.0708	5	4	WF
01_064 sx	2.1290	2.1290	0.1838	0.3521	1.4257	5	2.3674	2.3674	0.2581	0.4197	1.3192	5	2.6424	0.3330	0.4777	0.4107	1.3801	5	2.7543	2.8093	2.7543	2.7543	0.4071	5	3.0641	3.1004	3.3704	3.0641	3.0641	5	1.9966	2.0335	0.0356	1.9966	1.9966	5	6	WF
01_065 dx	1.7963	1.7963	0.0710	0.2002	0.5946	0	1.8925	1.8925	0.1103	0.2462	0.7832	0	2.0543	0.1620	0.3053	0.2131	0.8908	0	2.0797	2.1321	2.0797	2.0797	0.1888	0	2.2365	2.2703	2.1479	2.2365	2.2365	0	1.9203	1.9646	0.0460	1.9203	1.9203	0	0	NF
01_065 sx	2.0416	2.0416	0.1733	0.3375	1.4236	5	2.1773	2.1773	0.2217	0.3850	1.2638	5	2.3609	0.2684	0.4269	0.3500	1.2852	5	2.3957	2.4482	2.3957	2.3957	0.3121	5	2.5698	2.6027	2.6092	2.5698	2.5698	5	1.9652	2.0080	0.0425	1.9652	1.9652	0	5	WF
01_066 dx	2.0570	2.0570	0.1623	0.3215	1.0831	5	2.2397	2.2397	0.2255	0.3816	1.1623	5	2.4676	0.2743	0.4227	0.3408	1.1832	5	2.5251	2.5831	2.5251	2.5251	0.3215	5	2.7405	2.7769	2.8293	2.7405	2.7405	5	2.2356	2.2855	0.0388	2.2356	2.2356	5	6	WF
01_066 sx	1.9725	1.9725	0.1561	0.3062	1.3881	5	2.1452	2.1452	0.2015	0.3538	1.1820	5	2.3497	0.2676	0.4156	0.3415	1.2766	5	2.3977	2.4501	2.3977	2.3977	0.3159	5	2.5827	2.6159	2.7005	2.5827	2.5827	5	2.2706	2.3272	0.0426	2.2706	2.2706	4	6	WF
01_067 dx	1.6484	1.6484	0.0439	0.1686	0.4044	0	1.6595	1.6595	0.1068	0.2509	0.8324	0	1.8070	0.1506	0.3079	0.2301	0.9245	0	1.8410	1.8860	1.8410	1.8410	0.1830	0	2.0060	2.0363	1.9181	2.0060	2.0060	0	1.7639	1.8043	0.0502	1.7639	1.7639	0	0	NF
01_067 sx	1.5717	1.5717	0.0546	0.1801	0.4598	0	1.6233	1.6233	0.0923	0.2372	0.7522	0	1.7265	0.1351	0.2941	0.2315	0.8486	0	1.7461	1.7852	1.7461	1.7461	0.1799	0	1.9148	1.9418	1.8233	1.9148	1.9148	0	1.8145	1.8550	0.0473	1.8145	1.8145	0	0	NF
01_068 dx	2.8065	2.8065	0.3307	0.4708	1.7553	5	3.1395	3.1395	0.4531	0.5497	1.6073	5	3.4924	0.5714	0.6184	0.5058	1.6989	5	3.6314	3.6967	3.6314	3.6314	0.6836	5	3.9910	4.0311	5.0429	3.9910	3.9910	5	2.4361	2.4953	0.0396	2.4361	2.4361	5	6	WF
01_068 sx	3.1287	3.1287	0.3850	0.5275	2.6349	5	3.3377	3.3377	0.5301	0.6163	2.0490	5	3.6411	0.6595	0.6820	0.5359	2.1426	5	3.7316	3.8080	3.7316	3.7316	0.7796	5	4.0687	4.1168	4.9667	4.0687	4.0687	5	1.9950	2.0339	0.0376	1.9950	1.9950	5	6	WF
01_069 dx	1.7357	1.7357	0.1069	0.2610	0.9436	1	1.8603	1.8603	0.1573	0.3245	1.0816	1	2.0507	0.2180	0.3897	0.3652	1.1472	4	2.1009	2.1496	2.1009	2.1009	0.2626	1	2.2917	2.3224	2.3085	2.2917	2.2917	0	2.1068	2.1509	0.0384	2.1068	2.1068	5	2	NF
01_069 sx	1.8344	1.8344	0.0742	0.2125	0.6453	0	1.9387	1.9387	0.1238	0.2782	0.9138	0	2.0866	0.1748	0.3344	0.2903	0.9955	0	2.0934	2.1438	2.0934	2.0934	0.2083	0	2.2091	2.2403	2.1903	2.2091	2.2091	0	1.9151	1.9631	0.0498	1.9151	1.9151	0	0	NF
01_070 dx	1.9563	1.9563	0.1337	0.2948	0.8848	4	2.0693	2.0693	0.1772	0.3413	1.0018	4	2.2246	0.2224	0.3845	0.3247	1.0331	4	2.2541	2.2970	2.2541	2.2541	0.2602	5	2.4127	2.4396	2.5109	2.4127	2.4127	5	2.1876	2.2349	0.0383	2.1876	2.1876	5	4	WF
01_070 sx	2.3792	2.3792	0.2535	0.4180	1.6129	5	2.5975	2.5975	0.3137	0.4580	1.4318	5	2.8513	0.3772	0.4963	0.4269	1.4374	5	2.9272	2.9854	2.9272	2.9272	0.4403	5	3.1806	3.2168	3.5401	3.1806	3.1806	5	2.2926	2.3440	0.0383	2.2926	2.2926	5	6	WF
01_071 dx	1.9859	1.9859	0.1305	0.2788	0.8262	3	2.1324	2.1324	0.1695	0.3179	0.9104	2	2.3103	0.2230	0.3706	0.2872	0.9795	2	2.3524	2.3993	2.3524	2.3524	0.2625	5	2.5178	2.5474	2.6651	2.5178	2.5178	5	2.1288	2.1707	0.0358	2.1288	2.1288	5	3	WF
01_071 sx	2.2339	2.2339	0.1768	0.3397	1.0573	5	2.3667	2.3667	0.2299	0.3887	1.1210	5	2.5419	0.2853	0.4353	0.3738	1.1615	5	2.5943	2.6453	2.5943	2.5943	0.3665	5	2.8309	2.8634	3.2107	2.8309	2.8309	5	2.1814	2.2226	0.0337	2.1814	2.1814	5	6	WF
01_072 dx	1.8765	1.8765	0.1088	0.2716	0.6887	2	1.9567	1.9567	0.1499	0.3089	0.8938	0	2.0718	0.1942	0.3541	0.2808	0.9522	0	2.1146	2.1497	2.1146	2.1146	0.2402	0	2.2676	2.2902	2.2753	2.2676	2.2676	0	1.9616	1.9938	0.0324	1.9616	1.9616	1	0	NF
01_072 sx	2.2435	2.2435	0.1503	0.3008	1.1297	5	2.4289	2.4289	0.2846	0.4343	1.3338	5	2.6702	0.3670	0.4951	0.4204	1.4278	5	2.7661	2.8171	2.7661	2.7661	0.4257	5	3.0434	3.0762	3.2871	3.0434	3.0434	5	2.1721	2.2100	0.0314	2.1721	2.1721	5	6	WF
01_073 dx	2.3999	2.3999	0.1584	0.3381	1.2902	5	2.5512	2.5512	0.2933	0.4422	1.4441	5	2.7705	0.3555	0.4864	0.4161	1.4590	5	2.8095	2.8670	2.8095	2.8095	0.4063	5	3.0033	3.0370	3.1785	3.0033	3.0033	5	2.2411	2.2874	0.0359	2.2411	2.2411	5	6	WF
01_073 sx	2.4209	2.4209	0.2438	0.4019	0.9464	5	2.6053	2.6053	0.3124	0.4599	1.0965	5	2.8172	0.3785	0.5098	0.4411	1.1660	5	2.8890	2.9432	2.8890	2.8890	0.4315	5	3.1397	3.1736	4.0894	3.1397	3.1397	5	2.8137	2.8689	0.0295	2.8137	2.8137	5	6	WF
01_074 dx	2.2504	2.2504	0.1222	0.2888	0.9343	3	2.2242	2.2242	0.1473	0.3175	0.9821	2	2.2767	0.2110	0.3764	0.3057	1.0641	2	2.2535	2.2896	2.2535	2.2535	0.2479	4	2.3309	2.3528	2.3711	2.3309	2.3309	0	2.2665	2.3069	0.0309	2.2665	2.2665	5	2	NF
01_074 sx	2.3180	2.3180	0.1290	0.2838	0.8155	4	2.3200	2.3200	0.1819	0.3448	0.9656	4	2.4077	0.2660	0.4200	0.3122	1.0956	5	2.4229	2.4576	2.4229	2.4229	0.3259	5	2.5412	2.5621	2.8268	2.5412	2.5412	5	2.1437	2.1738	0.0256	2.1437	2.1437	5	4	WF
01_075 dx	2.0139	2.0139	0.1507	0.3041	0.9280	4	2.0972	2.0972	0.1836	0.3390	0.9825	4	2.1905	0.2207	0.3795	0.3325	1.0389	4	2.2167	2.2566	2.2167	2.2167	0.2993	5	2.4065	2.4319	2.6266	2.4065	2.4065	5	1.9222	1.9572	0.0366	1.9222	1.9222	1	3	WF
01_075 sx	2.8551	2.8551	0.3608	0.5020	1.2131	5	3.0886	3.0886	0.4479	0.5589	1.2551	5	3.3497	0.5488	0.6143	0.4849	1.3364	5	3.4666	3.5150	3.4666	3.4666	0.6433	5	3.7651	3.7976	5.2362	3.7651	3.7651	5	2.7204	2.7662	0.0259	2.7204	2.7204	5	6	WF
01_076 dx	2.1298	2.1298	0.1700	0.3411	1.1088	5	2.2801	2.2801	0.2298	0.3975	1.2018	5	2.4849	0.2964	0.4516	0.3769	1.2511	5	2.5383	2.5897	2.5383	2.5383	0.3422	5	2.7346	2.7669	2.8943	2.7346	2.7346	5	2.4816	2.5297	0.0315	2.4816	2.4816	5	6	WF
01_076 sx	2.6086	2.6086	0.2706	0.4301	1.6691	5	2.8149	2.8149	0.3524	0.4889	1.5026	5	3.0768	0.4289	0.5385	0.4428	1.5309	5	3.1221	3.1967	3.1221	3.1221	0.5077	5	3.3591	3.4049	3.8575	3.3591	3.3591	5	2.3396	2.3949	0.0397	2.3396	2.3396	5	6	WF
01_077 dx	2.3122	2.3122	0.2078	0.3671	1.4495	5	2.4755	2.4755	0.2670	0.4149	1.2218	5	2.6647	0.3211	0.4545	0.3811	1.2262	5	2.7129	2.7595	2.7129	2.7129	0.3768	5	2.9021	2.9311	3.3113	2.9021	2.9021	5	2.2719	2.3082	0.0277	2.2719	2.2719	5	6	WF
01_077 sx	2.5851	2.5851	0.2901	0.4470	1.8146	5	2.8903	2.8903	0.3906	0.5137	1.6032	5	3.2368	0.4917	0.5749	0.4863	1.7206	5	3.3604	3.4345	3.3604	3.3604	0.5914	5	3.7208	3.7681	4.3208	3.7208	3.7208	5	2.4655	2.5232	0.0355	2.4655	2.4655	5	6	WF
01_078 dx	1.8827	1.8827	0.1388	0.2978	1.0652	5	2.0053	2.0053	0.1742	0.3269	1.0105	3	2.1690	0.2010	0.3492	0.2526	0.9907	0	2.2063	2.2483	2.2063	2.2063	0.2303	4	2.3663	2.3924	2.3471	2.3663	2.3663	0	2.0604	2.0969	0.0333	2.0604	2.0604	5	3	WF
01_078 sx	2.2721	2.2721	0.1821	0.3374	1.3409	5	2.3915	2.3915	0.2410	0.3908	1.2270	5	2.5460	0.2963	0.4385	0.3753	1.2788	5	2.5784	2.6216	2.5784	2.5784	0.3542	5	2.7463	2.7735	2.9654	2.7463	2.7463	5	2.0717	2.1032	0.0286	2.0717	2.0717	5	6	WF
01_079 dx	1.8767	1.8767	0.1181	0.2712	0.8574	2	2.0050	2.0050	0.1648	0.3266	0.9694	2	2.1815	0.2046	0.3674	0.3112	0.9918	2	2.2199	2.2660	2.2199	2.2199	0.2506	4	2.3913	2.4203	2.4228	2.3913	2.3913	0	2.1174	2.1632	0.0394	2.1174	2.1174	5	2	NF
01_079 sx	2.3611	2.3611	0.1272	0.3042	0.9473	5	2.4251	2.4251	0.3162	0.4763	1.3612	5	2.6727	0.3586	0.5019	0.4613	1.3238	5	2.7789	2.8209	2.7789	2.7789	0.4135	5	3.0301	3.0564	3.3502	3.0301	3.0301	5	2.3071	2.3455	0.0285	2.3071	2.3071	5	6	WF
01_080 dx	2.5655	2.5655	0.2740	0.4348	1.1546	5	2.7806	2.7806	0.3498	0.4898	1.2027	5	2.9977	0.4344	0.5453	0.4975	1.2675	5	3.1062	3.1377	3.1062	3.1062	0.5110	5	3.3642	3.3837	4.1565	3.3642	3.3642	5	2.3798	2.4175	0.0266	2.3798	2.3798	5	6	WF
01_080 sx	2.7489	2.7489	0.3006	0.4483	2.0086	5	2.9030	2.9030	0.3853	0.5087	1.4906	5	3.1044	0.4505	0.5511	0.4839	1.5044	5	3.1423	3.1985	3.1423	3.1423	0.5131	5	3.3427	3.3766	3.8702	3.3427	3.3427	5	2.3830	2.4257	0.0299	2.3830	2.3830	5	6	WF

01_081 dx	1.8809	1.8809	0.1308	0.2820	0.7516	5	1.9907	1.9907	0.1645	0.3207	0.8594	2	2.1276	0.2025	0.3625	0.3180	0.8967	1	2.1600	2.1949	2.1600	2.1600	0.2486	0	2.3044	2.3261	2.4589	2.3044	2.3044	1	2.2051	2.2467	0.0334	2.2051	2.2051	5	2	NF
01_081 sx	1.9428	1.9428	0.1007	0.2419	0.6252	2	2.0057	2.0057	0.1481	0.3021	0.8836	2	2.1051	0.2092	0.3895	0.3760	0.9995	2	2.1487	2.1850	2.1487	2.1487	0.2457	0	2.2980	2.3204	2.4044	2.2980	2.2980	0	2.2855	2.3267	0.0311	2.2855	2.2855	5	1	NF
01_082 dx	2.1289	2.1289	0.1542	0.3252	0.8531	4	2.2651	2.2651	0.2149	0.3826	1.0467	5	2.4647	0.2795	0.4329	0.3813	1.1210	5	2.5396	2.5877	2.5396	2.5396	0.3295	5	2.7860	2.8175	2.9972	2.7860	2.7860	5	2.4291	2.4802	0.0345	2.4291	2.4291	5	5	WF
01_082 sx	2.1034	2.1034	0.1075	0.2661	0.6959	2	2.1317	2.1317	0.1593	0.3212	0.9278	2	2.2181	0.1885	0.3546	0.3053	0.9354	1	2.2072	2.2460	2.2072	2.2072	0.2431	3	2.2994	2.3222	2.4811	2.2994	2.2994	1	2.0340	2.0763	0.0393	2.0340	2.0340	5	2	NF
01_083 dx	2.4648	2.4648	0.2507	0.4051	1.4462	5	2.6993	2.6993	0.3399	0.4760	1.4134	5	2.9624	0.4233	0.5341	0.4684	1.4789	5	3.0524	3.1092	3.0524	3.0524	0.4877	5	3.3335	3.3688	3.8730	3.3335	3.3335	5	2.6539	2.7026	0.0286	2.6539	2.6539	5	6	WF
01_083 sx	2.2157	2.2157	0.1495	0.3108	0.9559	5	2.3393	2.3393	0.2237	0.3840	1.1250	5	2.4827	0.2805	0.4374	0.4015	1.1795	5	2.4920	2.5342	2.4920	2.4920	0.3378	5	2.6818	2.7079	3.0693	2.6818	2.6818	5	2.3870	2.4292	0.0296	2.3870	2.3870	5	6	WF
01_084 dx	2.7095	2.7095	0.3302	0.4854	2.8867	5	2.9354	2.9354	0.4392	0.5543	1.8505	5	3.1991	0.5117	0.5939	0.4879	1.8381	5	3.2914	3.3457	3.2914	3.2914	0.5852	5	3.5602	3.5934	3.8228	3.5602	3.5602	5	2.4862	2.5285	0.0277	2.4862	2.4862	5	6	WF
01_084 sx	2.7453	2.7453	0.3105	0.4686	1.5830	5	3.0096	3.0096	0.4515	0.5626	1.5859	5	3.3206	0.5625	0.6262	0.5590	1.7217	5	3.4358	3.4978	3.4358	3.4358	0.6528	5	3.7503	3.7895	4.6694	3.7503	3.7503	5	2.4663	2.5193	0.0349	2.4663	2.4663	5	6	WF
01_085 dx	1.7105	1.7105	0.0953	0.2431	0.7662	0	1.8212	1.8212	0.1318	0.2905	0.9206	0	1.9843	0.1670	0.3299	0.2609	0.9460	0	2.0293	2.0737	2.0293	2.0293	0.2132	0	2.2213	2.2505	2.1875	2.2213	2.2213	0	1.8854	1.9268	0.0447	1.8854	1.8854	0	0	NF
01_085 sx	1.9177	1.9177	0.0878	0.2395	0.6966	2	1.9776	1.9776	0.1637	0.3219	1.0091	2	2.1420	0.2143	0.3713	0.3038	1.0660	1	2.1738	2.2216	2.1738	2.1738	0.2413	0	2.3333	2.3632	2.2886	2.3333	2.3333	0	2.0352	2.0792	0.0408	2.0352	2.0352	5	1	NF
01_086 dx	1.9559	1.9559	0.1434	0.2953	0.9202	4	2.0992	2.0992	0.1862	0.3407	1.0420	5	2.2589	0.2293	0.3818	0.3120	1.0860	5	2.3180	2.3512	2.3180	2.3180	0.2714	5	2.4912	2.5123	2.5972	2.4912	2.4912	5	2.0028	2.0323	0.0286	2.0028	2.0028	5	5	WF
01_086 sx	2.4817	2.4817	0.2485	0.3992	1.9595	5	2.6392	2.6392	0.3183	0.4566	1.4621	5	2.8317	0.3802	0.4999	0.4249	1.4888	5	2.8569	2.9131	2.8569	2.8569	0.4385	5	3.0142	3.0483	3.4950	3.0142	3.0142	5	2.3523	2.4000	0.0340	2.3523	2.3523	5	6	WF
01_087 dx	2.6681	2.6681	0.2385	0.3922	1.0218	5	2.8578	2.8578	0.3400	0.4669	1.1202	5	3.0801	0.4144	0.5206	0.4461	1.1969	5	3.1502	3.1997	3.1502	3.1502	0.4978	5	3.3995	3.4299	4.5398	3.3995	3.3995	5	2.2363	2.2800	0.0341	2.2363	2.2363	5	6	WF
01_087 sx	2.3094	2.3094	0.1705	0.3360	1.7106	5	2.3247	2.3247	0.2057	0.3744	1.3070	5	2.4479	0.2814	0.4223	0.3673	1.2947	5	2.4967	2.5483	2.4967	2.4967	0.3342	5	2.6837	2.7159	2.7629	2.6837	2.6837	5	2.0338	2.0776	0.0406	2.0338	2.0338	5	6	WF
01_088 dx	1.9963	1.9963	0.1677	0.3389	1.0319	5	2.1514	2.1514	0.2065	0.3724	1.0845	5	2.3371	0.2562	0.4148	0.3569	1.1175	5	2.3829	2.4295	2.3829	2.3829	0.3029	5	2.5635	2.5918	2.6174	2.5635	2.5635	5	2.1402	2.1816	0.0350	2.1402	2.1402	5	6	WF
01_088 sx	1.9920	1.9920	0.0722	0.2267	0.4687	2	1.9939	1.9939	0.2155	0.3700	1.1165	5	2.1975	0.2499	0.4018	0.3503	1.1243	5	2.2749	2.3166	2.2749	2.2749	0.2761	5	2.4941	2.5206	2.5290	2.4941	2.4941	5	2.0117	2.0520	0.0383	2.0117	2.0117	5	5	WF
01_089 dx	2.5747	2.5747	0.2688	0.4241	1.4400	5	2.7931	2.7931	0.3749	0.5041	1.4064	5	3.0533	0.4538	0.5551	0.4818	1.4850	5	3.1328	3.1922	3.1328	3.1328	0.5289	5	3.3950	3.4313	4.2132	3.3950	3.3950	5	2.2195	2.2688	0.0389	2.2195	2.2195	5	6	WF
01_089 sx	2.3167	2.3167	0.1978	0.3734	1.4166	5	2.4701	2.4701	0.2758	0.4363	1.4097	5	2.6827	0.3445	0.4898	0.4567	1.4629	5	2.7342	2.7940	2.7342	2.7342	0.4203	5	2.9598	2.9968	3.2557	2.9598	2.9598	5	2.2813	2.3305	0.0370	2.2813	2.2813	5	6	WF
01_090 dx	2.6081	2.6081	0.1530	0.3200	1.3602	5	2.6632	2.6632	0.2043	0.3683	1.2997	5	2.8387	0.3109	0.4468	0.3875	1.4179	5	2.8478	2.9146	2.8478	2.8478	0.4037	5	3.0427	3.0840	3.3461	3.0427	3.0427	5	2.5385	2.6006	0.0388	2.5385	2.5385	5	6	WF
01_090 sx	3.0101	3.0101	0.2207	0.3767	1.5361	5	2.9921	2.9921	0.2656	0.4180	1.3540	5	3.0824	0.3169	0.4616	0.4155	1.3793	5	3.0422	3.1109	3.0422	3.0422	0.4461	5	3.2643	3.3062	4.0941	3.2643	3.2643	5	2.7492	2.8195	0.0386	2.7492	2.7492	5	6	WF
01_091 dx	2.2140	2.2140	0.1871	0.3519	1.3560	5	2.4169	2.4169	0.2675	0.4236	1.3412	5	2.6546	0.3320	0.4730	0.4087	1.3826	5	2.7292	2.7837	2.7292	2.7292	0.3912	5	2.9659	3.0010	3.2485	2.9659	2.9659	5	2.3283	2.3753	0.0342	2.3283	2.3283	5	6	WF
01_091 sx	2.0358	2.0358	0.1067	0.2615	1.0639	3	2.1386	2.1386	0.1756	0.3221	1.0761	4	2.3160	0.2366	0.3890	0.3263	1.2075	5	2.3688	2.4225	2.3688	2.3688	0.3026	5	2.5736	2.6086	2.5999	2.5736	2.5736	5	2.0996	2.1430	0.0379	2.0996	2.0996	5	4	WF
01_092 dx	2.1789	2.1789	0.1743	0.3263	1.5694	5	2.3686	2.3686	0.2358	0.3830	1.2793	5	2.6017	0.2925	0.4319	0.3478	1.3070	5	2.6715	2.7264	2.6715	2.6715	0.3416	5	2.9054	2.9400	2.9979	2.9054	2.9054	5	2.3144	2.3603	0.0338	2.3144	2.3144	5	6	WF
01_092 sx	2.4029	2.4029	0.2342	0.4038	1.4940	5	2.5624	2.5624	0.2919	0.4517	1.4062	5	2.7508	0.3535	0.4993	0.4665	1.4179	5	2.7649	2.8200	2.7649	2.7649	0.4127	5	2.9046	2.9379	3.2453	2.9046	2.9046	5	2.5353	2.5872	0.0327	2.5353	2.5353	5	6	WF
01_093 dx	2.2357	2.2357	0.1210	0.2840	0.8019	3	2.1952	2.1952	0.1509	0.3178	0.9446	2	2.2819	0.1895	0.3518	0.3106	0.9613	2	2.2784	2.3224	2.2784	2.2784	0.2485	4	2.3887	2.4149	2.3769	2.3887	2.3887	0	2.3497	2.3968	0.0337	2.3497	2.3497	5	2	NF
01_093 sx	2.1704	2.1704	0.1154	0.2754	0.6981	2	2.1775	2.1775	0.1301	0.2977	0.8275	2	2.2448	0.1645	0.3332	0.2943	0.8540	1	2.2804	2.3270	2.2804	2.2804	0.2688	5	2.4806	2.5113	2.6318	2.4806	2.4806	5	2.5592	2.6125	0.0331	2.5592	2.5592	5	3	WF
01_094 dx	2.4666	2.4666	0.2376	0.3959	1.2723	5	2.6226	2.6226	0.3325	0.4778	1.2559	5	2.7979	0.4041	0.5363	0.4820	1.2962	5	2.8659	2.9009	2.8659	2.8659	0.4579	5	3.0749	3.0965	3.7055	3.0749	3.0749	5	2.3535	2.3926	0.0281	2.3535	2.3535	5	6	WF
01_094 sx	2.9555	2.9555	0.3599	0.4948	1.1684	5	3.1632	3.1632	0.4863	0.5718	1.1702	5	3.3840	0.5727	0.6199	0.5482	1.2279	5	3.4962	3.5298	3.4962	3.4962	0.6561	5	3.7579	3.7794	5.8994	3.7579	3.7579	5	2.3100	2.3537	0.0323	2.3100	2.3100	5	6	WF
01_095 dx	2.2280	2.2280	0.1898	0.3587	1.5017	5	2.4354	2.4354	0.2472	0.4110	1.3591	5	2.6860	0.3257	0.4730	0.4039	1.4331	5	2.7598	2.8213	2.7598	2.7598	0.3831	5	3.0107	3.0500	3.2610	3.0107	3.0107	5	2.2288	2.2782	0.0386	2.2288	2.2288	5	6	WF
01_095 sx	2.4038	2.4038	0.2798	0.4527	1.4937	5	2.6367	2.6367	0.3435	0.4954	1.4057	5	2.8815	0.4047	0.5316	0.4983	1.4220	5	2.9809	3.0263	2.9809	2.9809	0.4646	5	3.2428	3.2713	3.7128	3.2428	3.2428	5	2.1614	2.1955	0.0285	2.1614	2.1614	5	6	WF
01_096 dx	2.8711	2.8711	0.3589	0.4891	2.1573	5	3.1575	3.1575	0.4728	0.5664	1.6494	5	3.4894	0.5839	0.6310	0.5075	1.7139	5	3.5785	3.6591	3.5785	3.5785	0.6856	5	3.8924	3.9436	5.1924	3.8924	3.8924	5	2.8628	2.9233	0.0315	2.8628	2.8628	5	6	WF
01_096 sx	2.4832	2.4832	0.2499	0.4213	1.7610	5	2.5853	2.5853	0.3129	0.4668	1.4801	5	2.7395	0.3951	0.5130	0.4475	1.5143	5	2.7979	2.8558	2.7979	2.7979	0.4607	5	3.0209	3.0575	3.6026	3.0209	3.0209	5	2.2893	2.3342	0.0336	2.2893	2.2893	5	6	WF
01_097 dx	2.1658	2.1658	0.1835	0.3561	1.5190	5	2.3260	2.3260	0.2384	0.3920	1.2841	5	2.5229	0.2871	0.4339	0.3575	1.2802	5	2.5652	2.6171	2.5652	2.5652	0.3341	5	2.7491	2.7799	2.7460	2.7491	2.7491	5	2.0437	2.0815	0.0349	2.0437	2.0437	5	6	WF
01_097 sx	2.1165	2.1165	0.1532	0.3203	1.2050	5	2.1920	2.1920	0.1923	0.3590	1.1119	5	2.3523	0.2484	0.4080	0.3397	1.1538	5	2.3693	2.4262	2.3693	2.3693	0.2941	5	2.5553	2.5917	2.6109	2.5553	2.5553	5	2.0165	2.0638	0.0446	2.0165	2.0165	5	6	WF

01_098 dx	2.2055	2.2055	0.1195	0.2853	0.8755	3	2.2339	2.2339	0.1648	0.3370	1.0470	4	2.3134	0.2390	0.4049	0.3447	1.1496	5	2.2918	2.3353	2.2918	2.2918	0.2737	5	2.3700	2.3954	2.5402	2.3700	2.3700	1	2.3105	2.3563	0.0338	2.3105	2.3105	5	3	WF
01_098 sx	1.8905	1.8905	0.1090	0.2563	0.7047	2	2.0092	2.0092	0.1552	0.3179	0.9441	2	2.1727	0.2128	0.3777	0.3239	1.0443	4	2.2186	2.2638	2.2186	2.2186	0.2730	5	2.4422	2.4728	2.4711	2.4422	2.4422	5	1.9449	1.9786	0.0344	1.9449	1.9449	5	3	WF
01_099 dx	1.9615	1.9615	0.1135	0.2841	0.7705	3	1.9989	1.9989	0.2178	0.3874	1.1791	5	2.1846	0.2556	0.4189	0.3853	1.1787	5	2.2424	2.2859	2.2424	2.2424	0.2881	5	2.4353	2.4632	2.4970	2.4353	2.4353	5	2.1660	2.2082	0.0349	2.1660	2.1660	5	5	WF
01_099 sx	1.9876	1.9876	0.1333	0.2966	0.9360	4	2.1259	2.1259	0.1871	0.3477	1.0277	5	2.3031	0.2341	0.3903	0.3213	1.0631	4	2.3564	2.3993	2.3564	2.3564	0.2745	5	2.5371	2.5657	2.6793	2.5371	2.5371	5	2.3289	2.3763	0.0344	2.3289	2.3289	5	5	WF
01_100 dx	1.8815	1.8815	0.1459	0.3082	0.9777	5	2.0500	2.0500	0.1880	0.3524	1.0689	5	2.2521	0.2401	0.3995	0.3404	1.1145	5	2.3139	2.3621	2.3139	2.3139	0.2885	5	2.5189	2.5500	2.5348	2.5189	2.5189	5	2.0153	2.0514	0.0343	2.0153	2.0153	5	6	WF
01_100 sx	1.8620	1.8620	0.0371	0.1542	0.2565	2	1.9153	1.9153	0.1131	0.2495	0.7061	0	2.0282	0.1488	0.2962	0.2031	0.7889	0	2.0313	2.0687	2.0313	2.0313	0.1747	0	2.1290	2.1517	2.1458	2.1290	2.1290	0	1.9099	1.9494	0.0415	1.9099	1.9099	0	0	NF
01_101 dx	2.1530	2.1530	0.1917	0.3542	1.0234	5	2.4501	2.4501	0.2723	0.4253	1.1402	5	2.7766	0.3585	0.4913	0.4047	1.2208	5	2.9145	2.9765	2.9145	2.9145	0.4418	5	3.2715	3.3120	3.9054	3.2715	3.2715	5	2.6639	2.7287	0.0375	2.6639	2.6639	5	6	WF
01_101 sx	2.8350	2.8350	0.3084	0.4470	2.6115	5	3.0837	3.0837	0.4092	0.5189	1.8398	5	3.3742	0.5087	0.5840	0.5181	1.9674	5	3.4267	3.5041	3.4267	3.4267	0.6028	5	3.6626	3.7101	4.3433	3.6626	3.6626	5	2.2301	2.2791	0.0383	2.2301	2.2301	5	6	WF
01_102 dx	2.5786	2.5786	0.3148	0.4627	1.9365	5	2.8786	2.8786	0.4182	0.5358	1.6318	5	3.2168	0.5097	0.5929	0.5362	1.7471	5	3.3327	3.4068	3.3327	3.3327	0.5998	5	3.6705	3.7174	4.6179	3.6705	3.6705	5	2.1795	2.2144	0.0287	2.1795	2.1795	5	6	WF
01_102 sx	2.5552	2.5552	0.2485	0.3886	1.9262	5	2.7503	2.7503	0.3136	0.4398	1.4172	5	3.0083	0.3805	0.4915	0.3808	1.4760	5	3.0596	3.1329	3.0596	3.0596	0.4480	5	3.3213	3.3677	3.6651	3.3213	3.3213	5	2.2820	2.3369	0.0411	2.2820	2.2820	5	6	WF
01_103 dx	2.5005	2.5005	0.2840	0.4436	1.8519	5	2.7576	2.7576	0.3926	0.5251	1.6090	5	3.0428	0.4941	0.5923	0.5551	1.7295	5	3.1389	3.1998	3.1389	3.1389	0.5891	5	3.4274	3.4656	4.0717	3.4274	3.4274	5	2.5130	2.5625	0.0317	2.5130	2.5130	5	6	WF
01_103 sx	2.1594	2.1594	0.1667	0.3282	1.2034	5	2.3089	2.3089	0.2276	0.3843	1.1771	5	2.4974	0.2748	0.4248	0.3564	1.1965	5	2.5365	2.5878	2.5365	2.5365	0.3201	5	2.7150	2.7481	2.9022	2.7150	2.7150	5	2.3087	2.3581	0.0364	2.3087	2.3087	5	6	WF
01_104 dx	2.0002	2.0002	0.1782	0.3549	1.3454	5	2.1977	2.1977	0.2371	0.4106	1.3336	5	2.4285	0.3117	0.4719	0.4224	1.4068	5	2.5281	2.5755	2.5281	2.5281	0.3752	5	2.8121	2.8430	2.8766	2.8121	2.8121	5	2.1741	2.2159	0.0344	2.1741	2.1741	5	6	WF
01_104 sx	2.2067	2.2067	0.1875	0.3421	1.1297	5	2.3985	2.3985	0.2487	0.3981	1.1644	5	2.6226	0.3101	0.4498	0.3803	1.2251	5	2.6988	2.7486	2.6988	2.6988	0.3707	5	2.9358	2.9674	3.2395	2.9358	2.9358	5	2.3390	2.3757	0.0266	2.3390	2.3390	5	6	WF
01_105 dx	1.9547	1.9547	0.0702	0.2188	0.4323	2	1.8946	1.8946	0.1035	0.2724	0.7716	0	2.0478	0.2084	0.3733	0.3220	0.9772	2	2.1327	2.1655	2.1327	2.1327	0.2394	0	2.3693	2.3910	2.5063	2.3693	2.3693	1	1.9890	2.0198	0.0303	1.9890	1.9890	4	1	NF
01_105 sx	2.2206	2.2206	0.1517	0.3241	1.0035	5	2.3217	2.3217	0.2128	0.3723	1.0813	5	2.4481	0.2658	0.4114	0.3527	1.0959	5	2.4728	2.5082	2.4728	2.4728	0.3027	5	2.5933	2.6147	2.8353	2.5933	2.5933	5	1.9870	2.0163	0.0289	1.9870	1.9870	4	6	WF
01_106 dx	2.4381	2.4381	0.2724	0.4326	1.2936	5	2.6680	2.6680	0.3472	0.4865	1.2779	5	2.9146	0.4326	0.5432	0.4945	1.3441	5	2.9956	3.0477	2.9956	2.9956	0.4950	5	3.2267	3.2592	3.9276	3.2267	3.2267	5	2.5314	2.5757	0.0281	2.5314	2.5314	5	6	WF
01_106 sx	2.2714	2.2714	0.1405	0.3054	0.8188	4	2.3253	2.3253	0.1774	0.3540	0.9957	4	2.4607	0.2791	0.4349	0.3714	1.1078	5	2.5250	2.5640	2.5250	2.5250	0.3413	5	2.7431	2.7680	3.0585	2.7431	2.7431	5	2.1816	2.2175	0.0295	2.1816	2.1816	5	4	WF
01_107 dx	2.1005	2.1005	0.1552	0.3250	1.0229	5	2.2664	2.2664	0.2194	0.3785	1.1513	5	2.4660	0.2683	0.4160	0.3493	1.1578	5	2.5128	2.5624	2.5128	2.5128	0.3089	5	2.6995	2.7297	2.7223	2.6995	2.6995	5	2.1297	2.1725	0.0364	2.1297	2.1297	5	6	WF
01_107 sx	2.2191	2.2191	0.1806	0.3393	1.2221	5	2.3533	2.3533	0.2290	0.3871	1.1690	5	2.5351	0.2774	0.4299	0.3619	1.1712	5	2.5617	2.6153	2.5617	2.5617	0.3310	5	2.7330	2.7651	2.8919	2.7330	2.7330	5	2.2038	2.2503	0.0372	2.2038	2.2038	5	6	WF
01_108 dx	1.8099	1.8099	0.0639	0.2139	0.4755	0	1.9369	1.9369	0.1796	0.3461	1.0994	3	2.1171	0.2165	0.3805	0.3214	1.0930	4	2.1742	2.2149	2.1742	2.1742	0.2512	0	2.3515	2.3771	2.3994	2.3515	2.3515	0	1.9829	2.0209	0.0372	1.9829	1.9829	4	2	NF
01_108 sx	2.2807	2.2807	0.1868	0.3431	1.3192	5	2.3462	2.3462	0.2462	0.3996	1.1611	5	2.4794	0.2978	0.4446	0.3532	1.1967	5	2.4925	2.5384	2.4925	2.4925	0.3392	5	2.6673	2.6958	3.0770	2.6673	2.6673	5	2.0998	2.1398	0.0351	2.0998	2.0998	5	6	WF
01_109 dx	1.9010	1.9010	0.0584	0.1953	0.3449	2	1.8921	1.8921	0.1910	0.3684	1.0122	2	2.0311	0.2185	0.3934	0.3683	1.0098	3	2.0783	2.1098	2.0783	2.0783	0.2447	0	2.2559	2.2763	2.3009	2.2559	2.2559	0	1.9969	2.0298	0.0320	1.9969	1.9969	5	2	NF
01_109 sx	2.0206	2.0206	0.1646	0.3351	0.9775	5	2.1796	2.1796	0.2141	0.3829	1.0881	5	2.3836	0.2592	0.4204	0.3554	1.1116	5	2.4303	2.4872	2.4303	2.4303	0.3088	5	2.6309	2.6664	2.6745	2.6309	2.6309	5	2.0736	2.1128	0.0352	2.0736	2.0736	5	6	WF
01_110 sx	2.6956	2.6956	0.3615	0.5039	1.4197	5	2.9682	2.9682	0.4631	0.5731	1.4174	5	3.2506	0.5560	0.6283	0.5678	1.4925	5	3.3640	3.4174	3.3640	3.3640	0.6449	5	3.6510	3.6848	5.0107	3.6510	3.6510	5	2.4884	2.5543	0.0424	2.4884	2.4884	4	6	WF
01_111 dx	1.9676	1.9676	0.1373	0.2895	1.0934	5	2.1605	2.1605	0.1970	0.3527	1.1212	5	2.3805	0.2595	0.4107	0.3618	1.1854	5	2.4425	2.4945	2.4425	2.4425	0.3153	5	2.6464	2.6796	2.6728	2.6464	2.6464	5	2.0595	2.1035	0.0399	2.0595	2.0595	5	6	WF
01_111 sx	1.8985	1.8985	0.1313	0.2836	0.8012	4	1.9962	1.9962	0.1578	0.3115	0.8780	2	2.1242	0.1862	0.3416	0.2631	0.8794	0	2.1592	2.1987	2.1592	2.1592	0.2375	0	2.3713	2.3973	2.4511	2.3713	2.3713	1	2.0053	2.0465	0.0394	2.0053	2.0053	5	1	NF
01_112 dx	2.5582	2.5582	0.2697	0.4405	1.7663	5	2.6936	2.6936	0.3509	0.5033	1.5039	5	2.8508	0.4073	0.5448	0.5281	1.5129	5	2.8657	2.9104	2.8657	2.8657	0.4624	5	2.9779	3.0038	3.5019	2.9779	2.9779	5	2.1966	2.2322	0.0289	2.1966	2.1966	5	6	WF
01_112 sx	2.3532	2.3532	0.1995	0.3587	1.4363	5	2.5413	2.5413	0.2535	0.4032	1.2242	5	2.7563	0.3046	0.4472	0.3716	1.2376	5	2.7957	2.8530	2.7957	2.7957	0.3648	5	2.9765	3.0117	3.1910	2.9765	2.9765	5	2.6101	2.6644	0.0326	2.6101	2.6101	5	6	WF
01_113 dx	1.8954	1.8954	0.0890	0.2422	0.6348	2	1.9329	1.9329	0.1581	0.3189	0.9491	0	2.0759	0.1948	0.3567	0.3055	0.9732	0	2.1206	2.1598	2.1206	2.1206	0.2587	1	2.2841	2.3084	2.2868	2.2841	2.2841	0	2.1361	2.1802	0.0374	2.1361	2.1361	5	1	NF
01_113 sx	2.2173	2.2173	0.1961	0.3522	1.7474	5	2.3904	2.3904	0.2523	0.4071	1.3823	5	2.6257	0.3303	0.4667	0.4065	1.4531	5	2.6807	2.7463	2.6807	2.6807	0.3889	5	2.9199	2.9609	2.9572	2.9199	2.9199	5	2.2840	2.3383	0.0406	2.2840	2.2840	5	6	WF
01_114 dx	2.5939	2.5939	0.2205	0.3668	1.0203	5	2.7941	2.7941	0.3104	0.4410	1.1836	5	3.0056	0.3821	0.5033	0.4237	1.2856	5	3.0728	3.1174	3.0728	3.0728	0.4594	5	3.2944	3.3215	4.0511	3.2944	3.2944	5	2.2857	2.3247	0.0295	2.2857	2.2857	5	6	WF
01_114 sx	2.8492	2.8492	0.3353	0.4796	1.3279	5	3.1033	3.1033	0.4468	0.5544	1.2951	5	3.3761	0.5542	0.6185	0.5577	1.3839	5	3.4886	3.5386	3.4886	3.4886	0.6578	5	3.7884	3.8195	5.7195	3.7884	3.7884	5	2.5115	2.5646	0.0339	2.5115	2.5115	5	6	WF
01_115 dx	1.9506	1.9506	0.1340	0.2904	0.8007	4	2.0670	2.0670	0.1818	0.3362	1.0010	4	2.2376	0.2199	0.3742	0.2795	1.0317	3	2.2727	2.3203	2.2727	2.2727	0.2653	5	2.4370	2.4669	2.5716	2.4370	2.4370	5	2.1659	2.2125	0.0384	2.1659	2.1659	5	4	WF

01_1 15 sx	1.9824	1.9824	0.0651	0.2113	0.5983	2	1.9284	1.9284	0.0981	0.2585	0.8692	0	2.1107	0.2168	0.3704	0.2629	1.1345	2	2.1462	2.1968	2.1462	2.1462	0.2436	0	2.2904	2.3224	2.2403	2.2904	2.2904	0	1.9645	2.0107	0.0457	1.9645	1.9645	0	0	NF
01_1 16 dx	1.9776	1.9776	0.1338	0.3056	0.7797	4	2.0902	2.0902	0.2129	0.3901	1.0508	5	2.2709	0.2780	0.4489	0.4206	1.0981	5	2.3736	2.4033	2.3736	2.3736	0.3305	5	2.6078	2.6274	2.8410	2.6078	2.6078	5	2.3463	2.3793	0.0240	2.3463	2.3463	5	5	WF
01_1 16 sx	2.2921	2.2921	0.2040	0.3745	1.1098	5	2.5002	2.5002	0.2856	0.4356	1.1865	5	2.7456	0.3512	0.4823	0.3947	1.2110	5	2.8418	2.8923	2.8418	2.8418	0.4238	5	3.1168	3.1496	3.6711	3.1168	3.1168	5	2.3468	2.3907	0.0316	2.3468	2.3468	5	6	WF
01_1 17 dx	2.1141	2.1141	0.0882	0.2505	0.7816	2	2.1088	2.1088	0.1200	0.2924	0.9705	2	2.2091	0.1920	0.3570	0.3019	1.1016	2	2.1904	2.2410	2.1904	2.1904	0.2282	0	2.2995	2.3302	2.2413	2.2995	2.2995	0	2.0078	2.0530	0.0429	2.0078	2.0078	3	1	NF
01_1 17 sx	1.8927	1.8927	0.1495	0.3076	1.0162	5	2.0444	2.0444	0.1800	0.3369	1.0114	4	2.2187	0.2172	0.3750	0.3129	1.0444	5	2.2726	2.3125	2.2726	2.2726	0.2556	4	2.4564	2.4818	2.4776	2.4564	2.4564	5	2.0927	2.1268	0.0302	2.0927	2.0927	5	5	WF
01_1 18 dx	2.2267	2.2267	0.1895	0.3538	1.2529	5	2.3938	2.3938	0.2494	0.4106	1.2231	5	2.6012	0.3140	0.4624	0.4162	1.2760	5	2.6608	2.7135	2.6608	2.6608	0.3679	5	2.8752	2.9077	3.1379	2.8752	2.8752	5	2.0111	2.0516	0.0385	2.0111	2.0111	5	6	WF
01_1 19 dx	2.1511	2.1511	0.0881	0.2411	0.5383	2	2.1207	2.1207	0.1232	0.2962	0.8470	2	2.2158	0.2443	0.4098	0.3571	1.0480	4	2.3129	2.3412	2.3129	2.3129	0.2914	5	2.5411	2.5595	2.6577	2.5411	2.5411	5	1.9646	1.9936	0.0291	1.9646	1.9646	1	3	WF
01_1 19 sx	2.0016	2.0016	0.1415	0.2955	0.9948	5	2.1910	2.1910	0.2026	0.3535	1.0911	5	2.3985	0.2526	0.3965	0.3269	1.1449	5	2.4716	2.5160	2.4716	2.4716	0.3123	5	2.6957	2.7238	2.7785	2.6957	2.6957	5	2.0414	2.0799	0.0357	2.0414	2.0414	5	6	WF
01_1 20 dx	2.4824	2.4824	0.2204	0.3834	1.5688	5	2.7009	2.7009	0.3051	0.4470	1.4070	5	2.9433	0.3863	0.5017	0.4210	1.4391	5	3.0242	3.0750	3.0242	3.0242	0.4603	5	3.2662	3.2968	3.7924	3.2662	3.2662	5	2.9338	2.9956	0.0310	2.9338	2.9338	5	6	WF
01_1 20 sx	2.3868	2.3868	0.1924	0.3609	1.6823	5	2.5988	2.5988	0.3023	0.4453	1.5008	5	2.8637	0.3712	0.4964	0.4212	1.5147	5	2.9694	3.0255	2.9694	2.9694	0.4618	5	3.2762	3.3120	3.6041	3.2762	3.2762	5	2.5563	2.6007	0.0278	2.5563	2.5563	5	6	WF
01_1 21 dx	2.1880	2.1880	0.1839	0.3457	1.2571	5	2.3198	2.3198	0.2266	0.3866	1.2021	5	2.4688	0.2669	0.4214	0.3534	1.2030	5	2.5003	2.5383	2.5003	2.5003	0.3140	5	2.6357	2.6584	2.7350	2.6357	2.6357	5	2.1912	2.2259	0.0283	2.1912	2.1912	5	6	WF
01_1 21 sx	2.5829	2.5829	0.2154	0.3908	1.0799	5	2.6771	2.6771	0.2785	0.4550	1.1950	5	2.8136	0.3577	0.5176	0.5253	1.2686	5	2.8083	2.8599	2.8083	2.8083	0.4300	5	2.9659	2.9974	3.6905	2.9659	2.9659	5	2.3412	2.3932	0.0374	2.3412	2.3412	5	6	WF
01_1 22 dx	2.6250	2.6250	0.2377	0.3871	1.2884	5	2.8135	2.8135	0.3185	0.4463	1.2159	5	3.0523	0.4062	0.5088	0.4171	1.3183	5	3.1052	3.1707	3.1052	3.1052	0.4834	5	3.3510	3.3917	4.1813	3.3510	3.3510	5	2.6102	2.6649	0.0328	2.6102	2.6102	5	6	WF
01_1 22 sx	2.3431	2.3431	0.1649	0.2971	1.0671	5	2.5495	2.5495	0.2259	0.3600	1.0821	5	2.8112	0.2921	0.4215	0.2958	1.1781	4	2.8620	2.9336	2.8620	2.8620	0.3580	5	3.0996	3.1440	3.6347	3.0996	3.0996	5	2.5505	2.6153	0.0402	2.5505	2.5505	5	6	WF
01_1 23 dx	1.9741	1.9741	0.0984	0.2581	0.6047	2	2.1050	2.1050	0.1912	0.3560	1.0374	5	2.2814	0.2352	0.3974	0.3390	1.0502	4	2.3351	2.3765	2.3351	2.3351	0.2790	5	2.5162	2.5424	2.7327	2.5162	2.5162	5	2.2589	2.3038	0.0344	2.2589	2.2589	5	5	WF
01_1 23 sx	2.2514	2.2514	0.2111	0.3786	1.7744	5	2.4470	2.4470	0.2794	0.4371	1.4632	5	2.6741	0.3453	0.4860	0.4338	1.4859	5	2.7275	2.7846	2.7275	2.7275	0.4108	5	2.9291	2.9642	3.2357	2.9291	2.9291	5	2.0924	2.1290	0.0324	2.0924	2.0924	5	6	WF
01_1 24 dx	2.1962	2.1962	0.1895	0.3409	1.4753	5	2.4489	2.4489	0.2654	0.4088	1.3061	5	2.7490	0.3400	0.4670	0.3593	1.3310	5	2.8597	2.9247	2.8597	2.8597	0.4119	5	3.1947	3.2367	3.4488	3.1947	3.1947	5	2.2905	2.3444	0.0401	2.2905	2.2905	5	6	WF
01_1 24 sx	2.9964	2.9964	0.2779	0.4231	1.9836	5	3.2052	3.2052	0.3703	0.5011	1.6173	5	3.4372	0.4616	0.5686	0.5195	1.7352	5	3.4256	3.5004	3.4256	3.4256	0.5515	5	3.5729	3.6170	4.5461	3.5729	3.5729	5	3.2691	3.3423	0.0312	3.2691	3.2691	5	6	WF
01_1 25 dx	2.0749	2.0749	0.2139	0.3935	1.5074	5	2.2545	2.2545	0.2783	0.4514	1.4258	5	2.4505	0.3360	0.4971	0.4914	1.4314	5	2.5034	2.5485	2.5034	2.5034	0.3886	5	2.6859	2.7137	3.0085	2.6859	2.6859	5	2.4446	2.4924	0.0321	2.4446	2.4446	5	6	WF
01_1 25 sx	2.6332	2.6332	0.3427	0.4908	1.6920	5	2.9393	2.9393	0.4756	0.5808	1.4751	5	3.2666	0.5865	0.6449	0.6151	1.5626	5	3.4128	3.4702	3.4128	3.4128	0.6890	5	3.7729	3.8100	5.3634	3.7729	3.7729	5	2.4347	2.4864	0.0348	2.4347	2.4347	5	6	WF
01_1 26 dx	2.3060	2.3060	0.2455	0.4092	1.4440	5	2.5272	2.5272	0.3122	0.4616	1.2944	5	2.7898	0.3791	0.5123	0.4586	1.3247	5	2.8699	2.9299	2.8699	2.8699	0.4417	5	3.1260	3.1632	3.8196	3.1260	3.1260	5	2.4005	2.4514	0.0350	2.4005	2.4005	5	6	WF
01_1 26 sx	1.9144	1.9144	0.0787	0.2179	0.7205	2	1.9438	1.9438	0.1150	0.2686	0.9120	0	2.0879	0.2209	0.3781	0.2949	1.1738	3	2.0986	2.1459	2.0986	2.0986	0.2496	0	2.2240	2.2531	2.1496	2.2240	2.2240	0	1.9087	1.9494	0.0429	1.9087	1.9087	0	1	NF
01_1 27 dx	2.1458	2.1458	0.1473	0.3052	0.7751	4	2.2630	2.2630	0.1781	0.3336	0.9502	4	2.4084	0.2184	0.3775	0.3334	0.9983	4	2.3990	2.4480	2.3990	2.3990	0.2719	5	2.5218	2.5515	2.8322	2.5218	2.5218	5	2.0421	2.0857	0.0402	2.0421	2.0421	5	4	WF
01_1 27 sx	2.3502	2.3502	0.1242	0.3010	1.0719	4	2.4139	2.4139	0.1809	0.3424	1.1470	5	2.5687	0.2630	0.4033	0.3272	1.2477	5	2.5742	2.6332	2.5742	2.5742	0.3322	5	2.7308	2.7667	2.7920	2.7308	2.7308	5	2.0688	2.1151	0.0415	2.0688	2.0688	4	5	WF
01_1 28 dx	2.2978	2.2978	0.2009	0.3656	1.2524	5	2.4764	2.4764	0.2610	0.4156	1.2215	5	2.6820	0.3125	0.4543	0.3733	1.2840	5	2.7305	2.7807	2.7305	2.7305	0.3838	5	2.9241	2.9546	3.1774	2.9241	2.9241	5	2.2874	2.3286	0.0310	2.2874	2.2874	5	6	WF
01_1 28 sx	1.9975	1.9975	0.1462	0.3310	0.7591	4	2.0585	2.0585	0.1973	0.3837	0.9474	4	2.1600	0.2447	0.4284	0.4331	0.9748	3	2.1913	2.2209	2.1913	2.1913	0.2822	1	2.3311	2.3502	2.5044	2.3311	2.3311	1	2.0943	2.1220	0.0247	2.0943	2.0943	5	2	NF
01_1 29 dx	2.5614	2.5614	0.2375	0.3935	1.1070	5	2.7282	2.7282	0.3285	0.4707	1.1404	5	2.9296	0.4078	0.5315	0.4583	1.1961	5	3.0064	3.0543	3.0064	3.0064	0.4950	5	3.2517	3.2827	4.1003	3.2517	3.2517	5	2.6124	2.6608	0.0291	2.6124	2.6124	5	6	WF
01_1 30 dx	2.3507	2.3507	0.1980	0.3605	1.3704	5	2.5191	2.5191	0.2618	0.4102	1.2373	5	2.7406	0.3241	0.4570	0.3874	1.2699	5	2.7918	2.8487	2.7918	2.7918	0.3820	5	3.0115	3.0465	3.4176	3.0115	3.0115	5	2.7813	2.8414	0.0326	2.7813	2.7813	5	6	WF
01_1 30 sx	2.0888	2.0888	0.1737	0.3376	1.2932	5	2.2096	2.2096	0.2184	0.3835	1.2074	5	2.3729	0.2610	0.4207	0.3605	1.2021	5	2.3858	2.4362	2.3858	2.3858	0.3034	5	2.5290	2.5594	2.7149	2.5290	2.5290	5	2.2208	2.2724	0.0405	2.2208	2.2208	5	6	WF
01_1 31 dx	2.1831	2.1831	0.1447	0.3026	0.8681	4	2.2699	2.2699	0.1953	0.3555	1.0315	5	2.3830	0.2392	0.3979	0.3409	1.0659	4	2.3749	2.4161	2.3749	2.3749	0.2806	5	2.4601	2.4845	2.6969	2.4601	2.4601	5	2.1120	2.1488	0.0321	2.1120	2.1120	5	5	WF
01_1 31 sx	2.6018	2.6018	0.2720	0.4332	1.5323	5	2.8138	2.8138	0.3545	0.4960	1.4175	5	3.0823	0.4533	0.5579	0.4514	1.5089	5	3.1579	3.2233	3.1579	3.1579	0.5293	5	3.4304	3.4709	3.9620	3.4304	3.4304	5	2.7585	2.8259	0.0369	2.7585	2.7585	5	6	WF
01_1 32 dx	2.7212	2.7212	0.3214	0.4718	2.7741	5	2.9967	2.9967	0.4334	0.5502	1.9473	5	3.3383	0.5371	0.6128	0.5005	2.0063	5	3.4259	3.5110	3.4259	3.4259	0.6326	5	3.7432	3.7955	4.2713	3.7432	3.7432	5	2.5149	2.5696	0.0349	2.5149	2.5149	5	6	WF
01_1 32 sx	2.2311	2.2311	0.1634	0.3278	0.8262	4	2.3443	2.3443	0.2156	0.3752	0.9729	4	2.5124	0.2807	0.4293	0.3461	1.0386	4	2.5808	2.6177	2.5808	2.5808	0.3456	5	2.7989	2.8229	3.1313	2.7989	2.7989	5	1.9753	2.0035	0.0282	1.9753	1.9753	1	3	WF
01_1 33 dx	2.2721	2.2721	0.1815	0.3567	1.3115	5	2.3207	2.3207	0.2371	0.4006	1.2514	5	2.4453	0.2996	0.4466	0.3818	1.2978	5	2.4588	2.5032	2.4588	2.4588	0.3354	5	2.5950	2.6217	2.6817	2.5950	2.5950	5	2.0791	2.1199	0.0364	2.0791	2.0791	5	6	WF

01_133 sx	2.0536	2.0536	0.1484	0.3160	1.2058	5	2.2087	2.2087	0.2188	0.3791	1.2384	5	2.4084	0.2583	0.4135	0.3616	1.2336	5	2.4433	2.4994	2.4433	2.4433	0.3100	5	2.6233	2.6590	2.6465	2.6233	2.6233	5	1.9683	2.0067	0.0382	1.9683	1.9683	4	5	WF
01_134 dx	2.3349	2.3349	0.2031	0.3510	1.2359	5	2.5453	2.5453	0.2791	0.4175	1.2551	5	2.7800	0.3560	0.4777	0.3981	1.3511	5	2.8757	2.9209	2.8757	2.8757	0.4295	5	3.1406	3.1688	3.6581	3.1406	3.1406	5	2.4335	2.4793	0.0310	2.4335	2.4335	5	6	WF
01_134 sx	1.8467	1.8467	0.0959	0.2531	0.5890	0	1.9507	1.9507	0.1515	0.3259	0.9156	0	2.0881	0.2081	0.3861	0.3524	1.0043	2	2.1404	2.1777	2.1404	2.1404	0.2693	1	2.3500	2.3750	2.4799	2.3500	2.3500	1	2.3343	2.3780	0.0317	2.3343	2.3343	5	1	NF
01_135 dx	2.0029	2.0029	0.0993	0.2660	0.7612	2	2.1409	2.1409	0.2189	0.3906	1.2248	5	2.3335	0.2659	0.4221	0.3445	1.2315	5	2.3848	2.4316	2.3848	2.3848	0.2970	5	2.5646	2.5943	2.6048	2.5646	2.5646	5	2.1773	2.2086	0.0259	2.1773	2.1773	5	5	WF
01_135 sx	2.2525	2.2525	0.2068	0.3665	0.9543	5	2.4454	2.4454	0.2646	0.4182	1.1493	5	2.6764	0.3316	0.4716	0.4016	1.2343	5	2.7581	2.8099	2.7581	2.7581	0.3927	5	3.0085	3.0420	3.6130	3.0085	3.0085	5	2.1690	2.2007	0.0264	2.1690	2.1690	5	6	WF
01_136 dx	1.9403	1.9403	0.0791	0.2113	0.6889	2	2.0014	2.0014	0.1230	0.2660	0.8674	2	2.1939	0.2250	0.3722	0.2780	1.1337	4	2.2746	2.3253	2.2746	2.2746	0.2794	5	2.5365	2.5705	2.4638	2.5365	2.5365	5	1.9120	1.9512	0.0412	1.9120	1.9120	0	3	WF
01_136 sx	1.7183	1.7183	0.0529	0.1718	0.4180	0	1.7762	1.7762	0.0847	0.2256	0.7069	0	1.8632	0.1166	0.2740	0.2030	0.7882	0	1.8560	1.8898	1.8560	1.8560	0.1664	0	1.9948	2.0158	1.9219	1.9948	1.9948	0	1.8752	1.9053	0.0332	1.8752	1.8752	0	0	NF
01_137 dx	2.4939	2.4939	0.2345	0.3870	1.4678	5	2.6904	2.6904	0.3088	0.4471	1.4472	5	2.9076	0.3813	0.4998	0.4293	1.4998	5	2.9901	3.0334	2.9901	2.9901	0.4515	5	3.2234	3.2503	3.6780	3.2234	3.2234	5	2.5395	2.5757	0.0229	2.5395	2.5395	5	6	WF
01_137 sx	2.1800	2.1800	0.1925	0.3505	1.1876	5	2.3244	2.3244	0.2439	0.3942	1.2146	5	2.4946	0.2829	0.4278	0.3644	1.2384	5	2.5249	2.5710	2.5249	2.5249	0.3323	5	2.6677	2.6956	2.7564	2.6677	2.6677	5	2.2734	2.3189	0.0345	2.2734	2.2734	5	6	WF
01_138 dx	1.9885	1.9885	0.1352	0.2815	0.9796	4	2.1014	2.1014	0.1935	0.3438	1.0642	5	2.2514	0.2224	0.3728	0.2996	1.0672	3	2.2769	2.3175	2.2769	2.2769	0.2612	5	2.4027	2.4267	2.3488	2.4027	2.4027	4	2.0493	2.0859	0.0337	2.0493	2.0493	5	5	WF
01_138 sx	2.4319	2.4319	0.2135	0.3810	1.4123	5	2.6147	2.6147	0.2989	0.4475	1.3547	5	2.8288	0.3831	0.5073	0.4245	1.4298	5	2.9158	2.9611	2.9158	2.9158	0.4484	5	3.1621	3.1908	3.5367	3.1621	3.1621	5	2.5335	2.5797	0.0292	2.5335	2.5335	5	6	WF
01_139 dx	2.0940	2.0940	0.1649	0.3160	0.9464	5	2.2483	2.2483	0.2130	0.3598	0.9711	4	2.4191	0.2502	0.3947	0.2913	0.9941	3	2.4646	2.5064	2.4646	2.4646	0.3025	5	2.6190	2.6463	2.8988	2.6190	2.6190	5	2.3611	2.4080	0.0333	2.3611	2.3611	5	5	WF
01_139 sx	2.2827	2.2827	0.2252	0.3950	1.5034	5	2.5084	2.5084	0.3026	0.4582	1.3731	5	2.7825	0.3727	0.5087	0.4555	1.4260	5	2.8642	2.9301	2.8642	2.8642	0.4459	5	3.1366	3.1792	3.6135	3.1366	3.1366	5	2.2623	2.3094	0.0359	2.2623	2.2623	5	6	WF
01_140 dx	2.2769	2.2769	0.1842	0.3641	1.0922	5	2.5292	2.5292	0.3473	0.4959	1.3911	5	2.8260	0.4108	0.5365	0.4737	1.3938	5	2.9569	3.0075	2.9569	2.9569	0.4770	5	3.2689	3.3016	3.7897	3.2689	3.2689	5	2.2714	2.3178	0.0352	2.2714	2.2714	5	6	WF
01_140 sx	2.6213	2.6213	0.2986	0.4560	2.1698	5	2.8766	2.8766	0.4104	0.5396	1.7991	5	3.1638	0.5143	0.6081	0.5746	1.8826	5	3.2474	3.3138	3.2474	3.2474	0.6189	5	3.5225	3.5641	4.0207	3.5225	3.5225	5	2.3523	2.4014	0.0350	2.3523	2.3523	5	6	WF
01_141 dx	2.1135	2.1135	0.1779	0.3361	1.7615	5	2.3247	2.3247	0.2512	0.4026	1.4008	5	2.5977	0.3128	0.4518	0.3781	1.4263	5	2.6615	2.7353	2.6615	2.6615	0.3729	5	2.9251	2.9726	2.9550	2.9251	2.9251	5	2.2566	2.3181	0.0467	2.2566	2.2566	4	6	WF
01_141 sx	1.8111	1.8111	0.1226	0.2794	1.1188	0	1.9507	1.9507	0.1596	0.3229	1.0943	1	2.1323	0.2015	0.3650	0.3089	1.1025	2	2.1758	2.2250	2.1758	2.1758	0.2393	0	2.3652	2.3968	2.4631	2.3652	2.3652	1	2.1907	2.2371	0.0375	2.1907	2.1907	5	1	NF
01_142 sx	2.1147	2.1147	0.1563	0.3176	0.7814	4	2.2414	2.2414	0.2193	0.3817	0.9625	4	2.4184	0.2696	0.4274	0.3730	1.0025	4	2.4857	2.5262	2.4857	2.4857	0.3194	5	2.7074	2.7330	3.0806	2.7074	2.7074	5	2.2691	2.3093	0.0308	2.2691	2.2691	5	4	WF
01_143 dx	2.2532	2.2532	0.2144	0.3904	1.3829	5	2.3807	2.3807	0.2775	0.4366	1.2990	5	2.5516	0.3164	0.4653	0.4237	1.2827	5	2.5955	2.6390	2.5955	2.5955	0.3700	5	2.7799	2.8063	3.0579	2.7799	2.7799	5	2.2338	2.2818	0.0374	2.2338	2.2338	5	6	WF
01_143 sx	2.4997	2.4997	0.1967	0.3701	1.4143	5	2.6634	2.6634	0.2851	0.4381	1.3930	5	2.8904	0.3813	0.5008	0.3993	1.5001	5	2.9491	3.0121	2.9491	2.9491	0.4499	5	3.1894	3.2291	3.4275	3.1894	3.1894	5	2.3263	2.3744	0.0350	2.3263	2.3263	5	6	WF
01_144 dx	2.2388	2.2388	0.1336	0.2872	0.9233	5	2.3093	2.3093	0.2028	0.3557	1.0966	5	2.4613	0.2640	0.4093	0.3279	1.1769	5	2.4923	2.5409	2.4923	2.4923	0.3052	5	2.6784	2.7091	2.7817	2.6784	2.6784	5	2.1020	2.1440	0.0367	2.1020	2.1020	5	6	WF
01_144 sx	2.6318	2.6318	0.2997	0.4510	1.9040	5	2.8303	2.8303	0.3910	0.5152	1.5123	5	3.0784	0.4668	0.5666	0.5139	1.5848	5	3.1523	3.2095	3.1523	3.1523	0.5408	5	3.4094	3.4447	4.2043	3.4094	3.4094	5	2.4661	2.5151	0.0324	2.4661	2.4661	5	6	WF
01_145 dx	1.9445	1.9445	0.0654	0.2180	0.5199	2	1.9103	1.9103	0.1001	0.2656	0.8327	0	2.0223	0.1934	0.3618	0.3407	1.0230	0	2.0412	2.0855	2.0412	2.0412	0.2424	0	2.1880	2.2164	2.1270	2.1880	2.1880	0	1.9540	1.9949	0.0411	1.9540	1.9540	0	0	NF
01_145 sx	2.0178	2.0178	0.0680	0.1990	0.5255	2	2.0320	2.0320	0.1138	0.2634	0.8128	2	2.1127	0.1495	0.3138	0.2695	0.8946	0	2.0786	2.1253	2.0786	2.0786	0.2127	0	2.1766	2.2048	2.1913	2.1766	2.1766	0	2.0544	2.0981	0.0398	2.0544	2.0544	5	1	NF
01_146 dx	2.1425	2.1425	0.1874	0.3462	0.9344	4	2.3015	2.3015	0.2387	0.3964	1.0176	5	2.4875	0.2896	0.4410	0.3846	1.0560	4	2.5502	2.5911	2.5502	2.5502	0.3394	5	2.7440	2.7696	3.1668	2.7440	2.7440	5	2.3938	2.4325	0.0270	2.3938	2.3938	5	5	WF
01_146 sx	2.1924	2.1924	0.2283	0.3957	1.3993	5	2.3919	2.3919	0.2787	0.4319	1.2665	5	2.6128	0.3272	0.4690	0.4129	1.2756	5	2.6909	2.7370	2.6909	2.6909	0.3715	5	2.9116	2.9404	3.1409	2.9116	2.9116	5	2.2328	2.2771	0.0347	2.2328	2.2328	5	6	WF
01_147 dx	2.4024	2.4024	0.1810	0.3578	1.2369	5	2.5278	2.5278	0.2504	0.4055	1.3535	5	2.6832	0.2940	0.4412	0.3776	1.3673	5	2.6644	2.7186	2.6644	2.6644	0.3436	5	2.7309	2.7624	2.9828	2.7309	2.7309	5	2.6057	2.6636	0.0348	2.6057	2.6057	5	6	WF
01_147 sx	2.6746	2.6746	0.2676	0.4105	1.0772	5	2.8660	2.8660	0.3323	0.4587	1.1216	5	3.0801	0.4034	0.5066	0.3918	1.1779	5	3.1510	3.1993	3.1510	3.1510	0.4806	5	3.3703	3.4002	4.4721	3.3703	3.3703	5	2.4497	2.4974	0.0318	2.4497	2.4497	5	6	WF
01_148 dx	2.1250	2.1250	0.1608	0.3403	1.1131	5	2.1701	2.1701	0.1973	0.3804	1.1586	5	2.3368	0.2766	0.4313	0.3912	1.2480	5	2.3850	2.4263	2.3850	2.3850	0.3385	5	2.5556	2.5808	2.7273	2.5556	2.5556	5	2.1515	2.1874	0.0302	2.1515	2.1515	5	6	WF
01_148 sx	3.2783	3.2783	0.4484	0.5631	2.9852	5	3.5837	3.5837	0.6092	0.6412	2.0616	5	3.9515	0.7295	0.6990	0.6042	2.1748	5	4.0694	4.1530	4.0694	4.0694	0.8759	5	4.4438	4.4963	6.3683	4.4438	4.4438	5	3.1423	3.2116	0.0313	3.1423	3.1423	5	6	WF
01_149 dx	2.1596	2.1596	0.1873	0.3456	1.6237	5	2.4012	2.4012	0.2607	0.4110	1.4133	5	2.7176	0.3409	0.4766	0.4041	1.5093	5	2.8236	2.9002	2.8236	2.8236	0.4228	5	3.1793	3.2300	3.4316	3.1793	3.1793	5	2.4507	2.5143	0.0420	2.4507	2.4507	4	6	WF
01_149 sx	1.7590	1.7590	0.0749	0.1990	0.7326	0	1.8577	1.8577	0.1174	0.2631	0.9306	0	2.0041	0.1573	0.3178	0.2604	1.0101	0	2.0015	2.0525	2.0015	2.0015	0.1931	0	2.1120	2.1428	2.0599	2.1120	2.1120	0	1.8407	1.8835	0.0485	1.8407	1.8407	0	0	NF
01_150 dx	2.2400	2.2400	0.2056	0.3693	1.4222	5	2.4881	2.4881	0.2829	0.4298	1.3432	5	2.7626	0.3443	0.4724	0.3989	1.3417	5	2.8386	2.9013	2.8386	2.8386	0.4038	5	3.0810	3.1198	3.5383	3.0810	3.0810	5	2.6050	2.6629	0.0348	2.6050	2.6050	5	6	WF
01_150 sx	2.9568	2.9568	0.3963	0.5265	3.0754	5	3.2813	3.2813	0.5160	0.5978	2.0468	5	3.6579	0.6271	0.6579	0.5598	2.1318	5	3.7702	3.8578	3.7702	3.7702	0.7413	5	4.1309	4.1858	5.1658	4.1309	4.1309	5	2.8664	2.9347	0.0353	2.8664	2.8664	5	6	WF

01_1 51 dx	2.144 5	2.1 445	0.11 76	0.28 27	0.72 08	3	2.19 82	2.19 82	0.19 14	0.35 14	0.98 62	4	2.33 78	0.24 18	0.39 49	0.33 76	1.00 56	4	2.39 67	2.43 97	2.39 67	2.3 967	0.30 62	5	2.61 31	2.64 07	2.87 54	2.6 131	2.6 131	5	2.14 93	2.18 91	0.03 34	2.14 93	2.14 93	5	4	WF
01_1 51 sx	2.531 1	2.5 311	0.25 85	0.41 09	1.69 97	5	2.69 26	2.69 26	0.31 72	0.45 46	1.46 32	5	2.90 72	0.39 05	0.50 49	0.37 87	1.50 96	5	2.94 37	3.00 67	2.94 37	2.9 437	0.44 45	5	3.14 87	3.18 74	3.27 57	3.1 487	3.1 487	5	2.25 03	2.28 84	0.02 96	2.25 03	2.25 03	5	6	WF
01_1 52 dx	1.794 2	1.7 942	0.09 71	0.24 28	0.58 85	0	1.86 11	1.86 11	0.13 57	0.29 23	0.82 49	0	2.00 52	0.20 74	0.36 61	0.29 12	0.95 53	0	2.07 91	2.10 92	2.07 91	2.0 791	0.23 76	0	2.26 67	2.28 63	2.39 06	2.2 667	2.2 667	0	1.95 46	1.98 73	0.03 31	1.95 46	1.95 46	1	0	NF
01_1 52 sx	2.507 1	2.5 071	0.18 27	0.37 78	1.25 85	5	2.50 47	2.50 47	0.23 64	0.42 93	1.28 59	5	2.62 85	0.34 72	0.49 32	0.45 06	1.40 99	5	2.65 74	2.70 75	2.65 74	2.6 574	0.42 29	5	2.82 34	2.85 40	3.24 18	2.8 234	2.8 234	5	2.59 04	2.65 59	0.03 96	2.59 04	2.59 04	5	6	WF
01_1 53 dx	2.021 4	2.0 214	0.14 11	0.31 84	0.79 16	4	2.08 51	2.08 51	0.20 05	0.36 60	1.00 23	4	2.20 15	0.25 16	0.41 08	0.36 10	1.03 16	4	2.23 16	2.26 22	2.23 16	2.2 316	0.26 69	5	2.36 35	2.38 22	2.59 33	2.3 635	2.3 635	1	2.18 15	2.22 03	0.03 18	2.18 15	2.18 15	5	3	WF
01_1 53 sx	2.226 9	2.2 269	0.13 58	0.29 94	0.57 34	4	2.36 46	2.36 46	0.20 70	0.36 34	0.85 16	4	2.52 85	0.28 60	0.42 85	0.33 71	0.95 56	4	2.58 60	2.62 44	2.58 60	2.5 860	0.33 66	5	2.77 40	2.79 82	3.41 70	2.7 740	2.7 740	5	2.28 20	2.32 03	0.02 90	2.28 20	2.28 20	5	4	WF
01_1 54 dx	2.108 6	2.1 086	0.09 12	0.25 15	0.80 09	2	2.09 27	2.09 27	0.11 33	0.27 41	0.89 21	2	2.14 25	0.15 41	0.31 96	0.27 65	0.94 81	0	2.10 42	2.15 04	2.10 42	2.1 042	0.25 21	0	2.22 91	2.25 79	2.17 11	2.2 291	2.2 291	0	1.88 64	1.92 65	0.04 33	1.88 64	1.88 64	0	0	NF
01_1 54 sx	1.886 8	1.8 868	0.06 50	0.20 58	0.51 85	2	2.00 05	2.00 05	0.15 21	0.29 83	0.91 33	2	2.20 39	0.19 20	0.34 23	0.26 89	0.96 98	1	2.25 26	2.30 65	2.25 26	2.2 526	0.25 11	4	2.45 71	2.49 13	2.44 19	2.4 571	2.4 571	5	2.09 34	2.14 76	0.04 72	2.09 34	2.09 34	4	3	WF
01_1 55 dx	2.671 7	2.6 717	0.28 09	0.43 65	1.71 47	5	2.82 76	2.82 76	0.35 05	0.49 05	1.50 77	5	3.04 78	0.44 31	0.55 02	0.47 95	1.55 43	5	3.08 83	3.15 26	3.08 83	3.0 883	0.50 54	5	3.30 10	3.34 03	3.66 12	3.3 010	3.3 010	5	2.35 80	2.40 71	0.03 49	2.35 80	2.35 80	5	6	WF
01_1 55 sx	2.145 5	2.1 455	0.15 41	0.33 26	1.06 15	5	2.21 39	2.21 39	0.20 12	0.38 12	1.13 95	5	2.33 20	0.25 16	0.42 62	0.40 18	1.16 90	5	2.30 80	2.35 79	2.30 80	2.3 080	0.28 74	5	2.39 58	2.42 54	2.46 51	2.3 958	2.3 958	5	2.15 77	2.20 70	0.04 09	2.15 77	2.15 77	5	6	WF
01_1 56 dx	2.445 6	2.4 456	0.17 08	0.32 58	1.18 17	5	2.46 75	2.46 75	0.20 06	0.37 19	1.12 47	5	2.52 12	0.25 14	0.42 76	0.41 40	1.20 58	5	2.43 01	2.47 60	2.43 01	2.4 301	0.30 07	5	2.60 43	2.63 27	2.84 23	2.6 043	2.6 043	5	2.09 57	2.13 47	0.03 44	2.09 57	2.09 57	5	6	WF
01_1 57 dx	2.233 6	2.2 336	0.20 39	0.37 27	1.88 72	5	2.39 93	2.39 93	0.26 55	0.42 35	1.43 54	5	2.62 13	0.32 22	0.46 91	0.42 49	1.42 55	5	2.65 96	2.72 20	2.65 96	2.6 596	0.37 75	5	2.85 56	2.89 38	2.99 66	2.8 556	2.8 556	5	2.16 91	2.22 06	0.04 22	2.16 91	2.16 91	4	6	WF
01_1 57 sx	2.253 5	2.2 535	0.21 20	0.36 72	1.54 50	5	2.46 23	2.46 23	0.27 58	0.42 53	1.32 64	5	2.71 41	0.33 74	0.47 58	0.40 75	1.36 46	5	2.77 15	2.83 63	2.77 15	2.7 715	0.39 60	5	2.99 73	3.03 78	3.22 82	2.9 973	2.9 973	5	1.97 87	2.01 69	0.03 75	1.97 87	1.97 87	1	5	WF
01_1 58 dx	2.074 8	2.0 748	0.16 76	0.32 09	1.16 30	5	2.31 98	2.31 98	0.23 45	0.38 27	1.17 93	5	2.59 41	0.29 63	0.43 41	0.31 93	1.24 01	5	2.69 91	2.75 47	2.69 91	2.6 991	0.36 24	5	2.99 38	3.02 86	3.16 03	2.9 938	2.9 938	5	1.91 66	1.95 23	0.03 74	1.91 66	1.91 66	1	5	WF
01_1 58 sx	1.883 8	1.8 838	0.13 59	0.29 08	1.07 43	5	1.99 97	1.99 97	0.16 69	0.32 09	0.97 27	2	2.15 01	0.19 62	0.34 98	0.28 26	0.95 23	0	2.16 82	2.21 20	2.16 82	2.1 682	0.22 33	0	2.28 95	2.31 55	2.23 90	2.2 895	2.2 895	0	2.13 92	2.18 21	0.03 63	2.13 92	2.13 92	5	2	NF
01_1 59 dx	1.788 0	1.7 880	0.06 34	0.21 91	0.39 51	0	1.84 13	1.84 13	0.16 03	0.32 56	0.90 59	0	1.97 50	0.18 73	0.35 55	0.29 23	0.90 99	0	2.01 42	2.04 81	2.01 42	2.0 142	0.22 84	0	2.16 04	2.18 18	2.23 45	2.1 604	2.1 604	0	2.09 61	2.13 69	0.03 58	2.09 61	2.09 61	5	1	NF
01_1 59 sx	2.328 9	2.3 289	0.20 83	0.36 49	1.14 52	5	2.46 24	2.46 24	0.26 04	0.41 49	1.09 31	5	2.62 58	0.31 14	0.45 47	0.39 39	1.13 15	5	2.65 17	2.69 67	2.65 17	2.6 517	0.35 89	5	2.80 09	2.82 79	3.23 65	2.8 009	2.8 009	5	2.22 44	2.26 23	0.03 01	2.22 44	2.22 44	5	6	WF
01_1 60 dx	2.359 2	2.3 592	0.14 23	0.31 10	0.80 17	4	2.44 87	2.44 87	0.21 90	0.38 41	1.10 97	5	2.60 33	0.29 69	0.44 82	0.38 94	1.18 96	5	2.66 77	2.71 19	2.66 77	2.6 677	0.36 66	5	2.90 98	2.93 81	3.30 99	2.9 098	2.9 098	5	2.36 45	2.40 70	0.03 02	2.36 45	2.36 45	5	5	WF
01_1 60 sx	2.355 4	2.3 554	0.20 37	0.36 44	1.32 08	5	2.53 63	2.53 63	0.28 18	0.42 45	1.27 99	5	2.75 61	0.35 57	0.48 16	0.41 23	1.35 64	5	2.81 72	2.86 93	2.81 72	2.8 172	0.41 76	5	3.02 12	3.05 35	3.61 53	3.0 212	3.0 212	5	2.20 82	2.24 78	0.03 17	2.20 82	2.20 82	5	6	WF
01_1 61 dx	2.079 2	2.0 792	0.16 85	0.32 26	1.16 90	5	2.33 63	2.33 63	0.24 43	0.39 41	1.24 36	5	2.61 65	0.31 89	0.45 25	0.37 72	1.32 31	5	2.73 54	2.78 95	2.73 54	2.7 354	0.39 30	5	3.04 43	3.07 90	3.28 77	3.0 443	3.0 443	5	2.40 61	2.45 08	0.03 08	2.40 61	2.40 61	5	6	WF
01_1 61 sx	2.489 7	2.4 897	0.23 88	0.39 50	1.44 25	5	2.64 22	2.64 22	0.29 15	0.43 23	1.31 31	5	2.83 08	0.34 18	0.46 85	0.38 40	1.30 45	5	2.86 14	2.91 56	2.86 14	2.8 614	0.40 22	5	3.02 84	3.06 15	3.30 56	3.0 284	3.0 284	5	2.54 89	2.60 13	0.03 28	2.54 89	2.54 89	5	6	WF
01_1 62 dx	2.096 8	2.0 968	0.12 34	0.28 40	0.86 75	3	2.15 74	2.15 74	0.16 64	0.34 03	1.05 95	4	2.27 09	0.22 40	0.39 61	0.36 75	1.13 90	5	2.26 30	2.30 68	2.26 30	2.2 630	0.27 72	5	2.38 46	2.41 03	2.44 56	2.3 846	2.3 846	1	2.18 47	2.22 96	0.03 65	2.18 47	2.18 47	5	3	WF
01_1 62 sx	2.213 1	2.2 131	0.17 63	0.34 43	1.10 04	5	2.33 82	2.33 82	0.22 72	0.39 15	1.17 08	5	2.52 63	0.28 97	0.44 03	0.37 10	1.21 53	5	2.55 99	2.61 28	2.55 99	2.5 599	0.32 91	5	2.73 20	2.76 43	2.86 69	2.7 320	2.7 320	5	2.24 53	2.29 99	0.04 20	2.24 53	2.24 53	4	6	WF
01_1 63 dx	2.019 8	2.0 198	0.16 51	0.32 94	1.28 13	5	2.14 60	2.14 60	0.21 51	0.37 86	1.22 78	5	2.32 68	0.26 98	0.42 30	0.36 75	1.24 96	5	2.36 93	2.42 15	2.36 93	2.3 693	0.31 30	5	2.56 00	2.59 27	2.56 61	2.5 600	2.5 600	5	2.16 14	2.21 09	0.04 09	2.16 14	2.16 14	5	6	WF
01_1 63 sx	2.347 1	2.3 471	0.21 65	0.37 17	1.77 37	5	2.54 54	2.54 54	0.28 60	0.43 09	1.40 75	5	2.80 15	0.35 50	0.48 42	0.42 34	1.46 39	5	2.84 47	2.91 85	2.84 47	2.8 447	0.42 17	5	3.07 27	3.11 83	3.32 71	3.0 727	3.0 727	5	2.29 10	2.34 48	0.04 00	2.29 10	2.29 10	5	6	WF
01_1 64 dx	2.348 0	2.3 480	0.25 26	0.40 81	2.40 29	5	2.57 51	2.57 51	0.32 14	0.46 23	1.65 40	5	2.87 62	0.38 96	0.51 07	0.43 51	1.64 69	5	2.95 22	3.03 24	2.95 22	2.9 522	0.45 75	5	3.25 75	3.30 89	3.53 70	3.2 575	3.2 575	5	2.39 00	2.45 41	0.04 41	2.39 00	2.39 00	5	6	WF
01_1 64 sx	1.953 7	1.9 537	0.15 58	0.32 45	1.08 44	5	2.08 66	2.08 66	0.20 36	0.37 08	1.18 16	5	2.27 06	0.25 24	0.41 05	0.35 21	1.21 96	5	2.31 65	2.36 68	2.31 65	2.3 165	0.31 00	5	2.50 32	2.53 53	2.62 11	2.5 032	2.5 032	5	2.08 98	2.13 94	0.04 36	2.08 98	2.08 98	5	6	WF
01_1 65 dx	2.375 7	2.3 757	0.23 31	0.39 34	2.06 71	5	2.58 37	2.58 37	0.30 83	0.45 54	1.58 97	5	2.83 08	0.38 07	0.50 73	0.44 89	1.62 11	5	2.89 87	2.96 03	2.89 87	2.8 987	0.45 04	5	3.14 07	3.18 07	3.42 75	3.1 407	3.1 407	5	2.52 92	2.58 90	0.03 77	2.52 92	2.52 92	5	6	WF
01_1 65 sx	2.139 1	2.1 391	0.12 52	0.27 22	1.04 22	3	2.23 42	2.23 42	0.18 09	0.33 52	1.09 03	5	2.39 82	0.23 53	0.38 87	0.31 76	1.14 69	5	2.42 66	2.47 81	2.42 66	2.4 266	0.29 46	5	2.62 71	2.65 95	2.75 32	2.6 271	2.6 271	5	2.23 69	2.28 37	0.03 65	2.23 69	2.23 69	5	5	WF
01_1 66 dx	1.930 8	1.9 308	0.14 44	0.30 15	1.07 25	5	2.06 86	2.06 86	0.18 81	0.34 36	1.11 09	5	2.24 29	0.23 27	0.38 63	0.31 57	1.14 36	5	2.28 41	2.33 08	2.28 41	2.2 841	0.28 06	5	2.45 74	2.48 59	2.46 84	2.4 574	2.4 574	5	2.04 58	2.08 66	0.03 75	2.04 58	2.04 58	5	6	WF
01_1 66 sx	2.331 7	2.3 317	0.18 08	0.35 96	0.96 01	5	2.39 16	2.39 16	0.23 12	0.40 59	1.08 48	5	2.54 00	0.34 83	0.48 37	0.42 52	1.23 08	5	2.61 29	2.65 53	2.61 29	2.6 129	0.39 60	5	2.82 19	2.84 87	3.32 87	2.8 219	2.8 219	5	2.25 56	2.30 11	0.03 50	2.25 56	2.25 56	5	6	WF
01_1 67 dx	2.225 5	2.2 255	0.19 53	0.35 79	1.47 64	5	2.34 32	2.34 32	0.23 37	0.39 60	1.22 91	5	2.50 02	0.28 17	0.43 52	0.38 20	1.24 12	5	2.51 36	2.56 25	2.51 36	2.5 136	0.32 29	5	2.64 93	2.67 85	2.73 77	2.6 493	2.6 493	5	2.32 44	2.36 64	0.03 07	2.32 44	2.32 44	5	6	WF
01_1 68 dx	2.467 4	2.4 674	0.25 72	0.41 70	1.39 99	5	2.64 34	2.64 34	0.32 21	0.46 32	1.36 40	5	2.86 06	0.37 49	0.49 90	0.41 80	1.35 99	5	2.90 33	2.96 17	2.90 33	2.9 033	0.42 68	5	3.11 66	3.15 22	3.55 32	3.1 166	3.1 166	5	2.25 57	2.30 28	0.03 62	2.25 57	2.25 57	5	6	WF
01_1 68 sx	2.038 6	2.0 386	0.15 99	0.32 59	1.06 99	5	2.21 25	2.21 25	0.21 01	0.37 77	1.14 46	5	2.42 78	0.26 61	0.42 81	0.38 32	1.19 80	5	2.46 83	2.52 81	2.46 83	2.4 683	0.30 89	5	2.65 88	2.69 64	2.72 17	2.6 588	2.6 588	5	2.52 39	2.58 77	0.04 02	2.52 39	2.52 39	5	6	WF

01_169 dx	2.4497	2.4497	0.2232	0.3814	2.1594	5	2.6567	2.6567	0.3064	0.4527	1.6032	5	2.9387	0.3843	0.5104	0.4229	1.6501	5	2.9912	3.0719	2.9912	2.9912	0.4742	5	3.2657	3.3167	3.4080	3.2657	3.2657	5	2.3584	2.4149	0.0399	2.3584	2.3584	5	6	WF
01_169 sx	2.2283	2.2283	0.1874	0.3381	1.5534	5	2.4308	2.4308	0.2489	0.3938	1.3076	5	2.6935	0.3144	0.4457	0.3690	1.3759	5	2.7838	2.8464	2.7838	2.7838	0.3808	5	3.0792	3.1203	3.1363	3.0792	3.0792	5	2.2802	2.3315	0.0385	2.2802	2.2802	5	6	WF
01_170 dx	2.2117	2.2117	0.1285	0.2932	1.1153	5	2.3174	2.3174	0.2089	0.3689	1.2075	5	2.5189	0.2712	0.4230	0.3533	1.2608	5	2.5557	2.6156	2.5557	2.5557	0.3323	5	2.7620	2.7993	2.7322	2.7620	2.7620	5	2.1371	2.1874	0.0423	2.1371	2.1371	4	6	WF
01_170 sx	2.1396	2.1396	0.1326	0.2941	0.8798	4	2.2542	2.2542	0.1904	0.3466	1.0722	5	2.4183	0.2370	0.3887	0.3210	1.1142	5	2.4414	2.4919	2.4414	2.4414	0.2994	5	2.5983	2.6293	2.6432	2.5983	2.5983	5	1.9716	2.0106	0.0387	1.9716	1.9716	1	4	WF
01_171 dx	2.0150	2.0150	0.1270	0.2781	1.1380	4	2.1312	2.1312	0.1819	0.3381	1.1469	5	2.3119	0.2372	0.3916	0.3207	1.2037	5	2.3526	2.4018	2.3526	2.3526	0.2829	5	2.5428	2.5738	2.5190	2.5428	2.5428	5	1.9062	1.9463	0.0424	1.9062	1.9062	0	4	WF
01_171 sx	1.5993	1.5993	0.0315	0.1416	0.2532	0	1.7167	1.7167	0.1221	0.2756	0.8456	0	1.8865	0.1521	0.3112	0.2183	0.8745	0	1.9343	1.9769	1.9343	1.9343	0.1833	0	2.1086	2.1364	2.0374	2.1086	2.1086	0	1.8753	1.9183	0.0468	1.8753	1.8753	0	0	NF
01_172 dx	1.9765	1.9765	0.1221	0.2785	0.6998	2	2.0807	2.0807	0.1802	0.3393	0.9396	4	2.2381	0.2382	0.4069	0.3692	0.9997	4	2.3057	2.3412	2.3057	2.3057	0.2801	5	2.4940	2.5174	2.7963	2.4940	2.4940	5	2.2746	2.3118	0.0283	2.2746	2.2746	5	4	WF
01_172 sx	2.1728	2.1728	0.1676	0.3455	1.1527	5	2.2695	2.2695	0.2328	0.4074	1.2069	5	2.4082	0.2757	0.4474	0.4249	1.2087	5	2.4248	2.4710	2.4248	2.4248	0.3321	5	2.5792	2.6072	2.8446	2.5792	2.5792	5	2.1016	2.1379	0.0319	2.1016	2.1016	5	6	WF
01_173 dx	1.6850	1.6850	0.0641	0.1978	0.5306	0	1.7864	1.7864	0.1083	0.2577	0.8155	0	1.9216	0.1372	0.2983	0.2689	0.8516	0	1.9322	1.9757	1.9322	1.9322	0.1895	0	2.0437	2.0712	1.9798	2.0437	2.0437	0	1.9861	2.0278	0.0406	1.9861	1.9861	5	1	NF
01_173 sx	2.0026	2.0026	0.1581	0.3254	0.9434	5	2.1623	2.1623	0.1958	0.3596	0.9927	5	2.3506	0.2401	0.3963	0.3310	1.0274	4	2.4095	2.4539	2.4095	2.4095	0.2902	4	2.6060	2.6338	2.8005	2.6060	2.6060	5	2.1239	2.1652	0.0355	2.1239	2.1239	5	6	WF
01_174 dx	2.5432	2.5432	0.2715	0.4230	1.3447	5	2.7905	2.7905	0.3601	0.4913	1.2503	5	3.0403	0.4440	0.5481	0.4409	1.3198	5	3.1629	3.1998	3.1629	3.1629	0.5348	5	3.4443	3.4668	4.1287	3.4443	3.4443	5	2.5571	2.5911	0.0214	2.5571	2.5571	5	6	WF
01_174 sx	3.3728	3.3728	0.5421	0.6191	3.7418	5	3.7868	3.7868	0.7023	0.6993	2.2819	5	4.2327	0.8777	0.7782	0.6451	2.4155	5	4.4177	4.5005	4.4177	4.4177	1.0321	5	4.8905	4.9437	6.8874	4.8905	4.8905	5	3.1526	3.2186	0.0297	3.1526	3.1526	5	6	WF
01_175 dx	3.0678	3.0678	0.3766	0.5034	2.2868	5	3.2967	3.2967	0.4827	0.5701	1.8022	5	3.5492	0.5783	0.6237	0.5143	1.8838	5	3.6113	3.6722	3.6113	3.6113	0.6664	5	3.8337	3.8718	4.7273	3.8337	3.8337	5	2.4452	2.4991	0.0360	2.4452	2.4452	5	6	WF
01_175 sx	2.4490	2.4490	0.2531	0.4087	1.8473	5	2.6435	2.6435	0.3401	0.4803	1.4883	5	2.8654	0.4225	0.5407	0.4840	1.5857	5	2.9322	2.9832	2.9322	2.9322	0.5004	5	3.1578	3.1893	3.5995	3.1578	3.1578	5	2.2255	2.2637	0.0302	2.2255	2.2255	5	6	WF
01_176 dx	2.1605	2.1605	0.1779	0.3350	1.0082	5	2.3286	2.3286	0.2341	0.3933	1.0653	5	2.5482	0.3041	0.4502	0.3681	1.1416	5	2.6281	2.6756	2.6281	2.6281	0.3605	5	2.8705	2.8998	3.2319	2.8705	2.8705	5	2.5750	2.6254	0.0310	2.5750	2.5750	5	6	WF
01_176 sx	2.5693	2.5693	0.2761	0.4220	1.9519	5	2.7944	2.7944	0.3486	0.4800	1.5042	5	3.0527	0.4239	0.5317	0.4361	1.5743	5	3.1245	3.1855	3.1245	3.1245	0.4995	5	3.3693	3.4072	3.7766	3.3693	3.3693	5	2.5562	2.6033	0.0294	2.5562	2.5562	5	6	WF
01_177 dx	2.9005	2.9005	0.3351	0.4922	1.6936	5	3.0101	3.0101	0.3996	0.5413	1.5224	5	3.1767	0.5144	0.6120	0.5998	1.6113	5	3.1909	3.2469	3.1909	3.1909	0.5909	5	3.3403	3.3750	4.3498	3.3403	3.3403	5	2.4592	2.5102	0.0337	2.4592	2.4592	5	6	WF
01_177 sx	2.6598	2.6598	0.2714	0.4158	1.4545	5	2.8401	2.8401	0.3462	0.4750	1.4046	5	3.0773	0.4180	0.5284	0.4033	1.4979	5	3.1282	3.1917	3.1282	3.1282	0.4957	5	3.3647	3.4037	4.3373	3.3647	3.3647	5	2.5953	2.6557	0.0365	2.5953	2.5953	5	6	WF
01_178 dx	2.1435	2.1435	0.1108	0.2778	0.6791	2	2.2216	2.2216	0.2253	0.3829	1.1376	5	2.4597	0.2814	0.4279	0.3531	1.1959	5	2.5372	2.5941	2.5372	2.5372	0.3376	5	2.7940	2.8310	2.9048	2.7940	2.7940	5	2.1722	2.2202	0.0393	2.1722	2.1722	5	5	WF
01_178 sx	2.3670	2.3670	0.2291	0.3908	1.3443	5	2.5903	2.5903	0.3124	0.4621	1.2779	5	2.8559	0.3886	0.5175	0.4557	1.3513	5	2.9557	3.0125	2.9557	2.9557	0.4737	5	3.2530	3.2896	3.9114	3.2530	3.2530	5	2.3492	2.3885	0.0283	2.3492	2.3492	5	6	WF
01_179 dx	2.2028	2.2028	0.1409	0.3074	0.7912	5	2.2671	2.2671	0.1968	0.3659	0.9669	4	2.3899	0.2676	0.4263	0.3736	1.0513	4	2.4185	2.4558	2.4185	2.4185	0.3007	4	2.5595	2.5816	2.9105	2.5595	2.5595	5	2.0921	2.1302	0.0337	2.0921	2.0921	5	5	WF
01_179 sx	2.5086	2.5086	0.2530	0.4324	1.7344	5	2.6337	2.6337	0.3463	0.4780	1.5963	5	2.8394	0.4053	0.5206	0.4648	1.6225	5	2.8891	2.9432	2.8891	2.8891	0.4897	5	3.1026	3.1360	3.4437	3.1026	3.1026	5	2.3757	2.4249	0.0345	2.3757	2.3757	5	6	WF
01_180 dx	2.5154	2.5154	0.1773	0.3376	1.6159	5	2.6885	2.6885	0.2694	0.4187	1.4674	5	2.9591	0.3822	0.4985	0.4147	1.5906	5	3.0205	3.0962	3.0205	3.0205	0.4616	5	3.3006	3.3492	3.6239	3.3006	3.3006	5	2.3663	2.4216	0.0389	2.3663	2.3663	5	6	WF
01_180 sx	2.1243	2.1243	0.1616	0.3103	1.5068	5	2.3180	2.3180	0.2153	0.3649	1.2930	5	2.5762	0.2710	0.4157	0.3403	1.3408	5	2.6219	2.6983	2.6219	2.6219	0.3273	5	2.8588	2.9091	2.8930	2.8588	2.8588	5	2.2295	2.2885	0.0458	2.2295	2.2295	5	6	WF

Supporting information S1B Table: methods and features for Genova

	method1						method2						method3						method4						method5						method6							
Threshold	2.01373	2.01373	0.12415	0.28449	0.8591		2.13573	2.13573	0.18652	0.35667	1.05491		2.3826	0.25856	0.41506	0.34179	1.11451		2.43816	2.48477	2.43816	2.43816	0.30592		2.61641	2.65247	2.73986	2.61641	2.61641		2.16856	2.21111	0.03711	2.16856	2.16856			
N_PATIENT' (dx=right; sx=left)	M2MEA_i'	MAX_n'	VAR_P'	MAD0_p'	VAR_r'	number of S=1 occurrence	M2MEA_i'	MAX_n'	VAR_p'	MAD0_p'	MAD0_r'	number of S=1 occurrence	ROS1_i'	VAR_p'	MAD0_p'	MAD1_p'	MAD0_r'	number of S=1 occurrence	M2MEA_i'	ROS1_i'	MAX_n'	MAX_p'	VAR_p'	number of S=1 occurrence	M2MEA_i'	ROS1_i'	ROS2_i'	MAX_n'	MAX_p'	number of S=1 occurrence	M2MEA_i'	ROS1_i'	MIN_n'	MAX_n'	MAX_p'	number of S=1 occurrence	number of statements verified	MammoWave rule-of-thumb output
02_016 dx	2.1879	2.1879	0.1806	0.3428	0.8355	4	2.3844	2.3844	0.2682	0.4254	1.0606	5	2.6114	0.3445	0.4878	0.4542	1.1433	5	2.7209	2.7609	2.7209	2.7209	0.4086	5	3.0000	3.0258	3.6864	3.0000	3.0000	5	2.2205	2.2527	0.0257	2.2205	2.2205	5	5	WF
02_016 sx	3.3008	3.3008	0.4490	0.5579	3.4621	5	3.6320	3.6320	0.6058	0.6452	2.1589	5	3.9746	0.7380	0.7131	0.6006	2.3299	5	4.1018	4.1700	4.1018	4.1018	0.8902	5	4.4460	4.4893	5.8390	4.4460	4.4460	5	2.5085	2.5644	0.0357	2.5085	2.5085	5	6	WF
02_017 dx	2.3250	2.3250	0.1292	0.2910	1.0777	5	2.4532	2.4532	0.2003	0.3476	1.1707	4	2.6305	0.2567	0.3964	0.3041	1.2067	2	2.6260	2.6882	2.6260	2.6260	0.3158	5	2.7627	2.7996	2.9565	2.7627	2.7627	5	2.0835	2.1288	0.0401	2.0835	2.0835	0	3	WF
02_017 sx	2.5867	2.5867	0.2439	0.4003	1.8885	5	2.7565	2.7565	0.3112	0.4408	1.4547	5	2.9859	0.3626	0.4740	0.3826	1.4254	5	3.0080	3.0774	3.0080	3.0080	0.4155	5	3.2050	3.2476	3.6783	3.2050	3.2050	5	2.1760	2.2248	0.0398	2.1760	2.1760	4	6	WF
02_018 dx	2.0807	2.0807	0.1363	0.2882	0.8826	5	2.1259	2.1259	0.1606	0.3183	0.9090	0	2.2259	0.1867	0.3480	0.3018	0.9035	0	2.2496	2.3003	2.2496	2.2496	0.2636	0	2.4361	2.4682	2.5261	2.4361	2.4361	0	2.2026	2.2552	0.0420	2.2026	2.2026	4	2	NF
02_018 sx	1.9372	1.9372	0.0821	0.2441	0.6498	0	1.9881	1.9881	0.1241	0.2857	0.9199	0	2.1179	0.1797	0.3374	0.2910	0.9809	0	2.1173	2.1624	2.1173	2.1173	0.2072	0	2.2232	2.2507	2.2428	2.2232	2.2232	0	2.1012	2.1462	0.0393	2.1012	2.1012	0	0	NF
02_019 dx	2.2237	2.2237	0.2046	0.3797	1.2225	5	2.3852	2.3852	0.2530	0.4251	1.1811	5	2.5577	0.3125	0.4750	0.4565	1.1975	5	2.6300	2.6625	2.6300	2.6300	0.3631	5	2.8459	2.8667	3.3192	2.8459	2.8459	5	2.3745	2.4130	0.0272	2.3745	2.3745	5	6	WF
02_019 sx	2.1753	2.1753	0.1624	0.3309	1.0109	5	2.3100	2.3100	0.2069	0.3793	1.0900	5	2.4750	0.2759	0.4413	0.4128	1.1564	5	2.5105	2.5544	2.5105	2.5105	0.3206	5	2.7076	2.7361	3.1624	2.7076	2.7076	5	2.2522	2.2962	0.0340	2.2522	2.2522	5	6	WF
02_020 dx	2.2239	2.2239	0.1300	0.2909	0.9313	5	2.2967	2.2967	0.1740	0.3484	1.1423	3	2.4121	0.2447	0.4138	0.3823	1.2517	3	2.3906	2.4363	2.3906	2.3906	0.3007	0	2.4905	2.5167	2.5770	2.4905	2.4905	0	2.0924	2.1359	0.0383	2.0924	2.0924	0	2	NF
02_020 sx	2.6504	2.6504	0.2711	0.4387	1.6412	5	2.7509	2.7509	0.3423	0.4940	1.4196	5	2.9066	0.3987	0.5375	0.5103	1.4383	5	2.8826	2.9422	2.8826	2.8826	0.4552	5	2.9771	3.0117	3.6086	2.9771	2.9771	5	2.5340	2.5909	0.0358	2.5340	2.5340	5	6	WF
02_021 dx	2.3357	2.3357	0.1950	0.3579	1.5284	5	2.4897	2.4897	0.2629	0.4169	1.4300	5	2.6809	0.3218	0.4653	0.4152	1.4654	5	2.7211	2.7716	2.7211	2.7211	0.3935	5	2.9054	2.9348	3.1394	2.9054	2.9054	5	2.2475	2.2913	0.0339	2.2475	2.2475	5	6	WF
02_021 sx	1.8922	1.8922	0.1395	0.2941	1.1533	3	2.1331	2.1331	0.1970	0.3524	1.1596	2	2.4156	0.2571	0.4069	0.3349	1.2146	2	2.5327	2.5917	2.5327	2.5327	0.3238	5	2.8574	2.8974	3.0643	2.8574	2.8574	5	2.4552	2.5108	0.0368	2.4552	2.4552	5	3	WF
02_022 dx	2.2370	2.2370	0.1349	0.3065	1.0913	5	2.2987	2.2987	0.1885	0.3648	1.1896	5	2.4282	0.2598	0.4257	0.3754	1.2566	5	2.4134	2.4681	2.4134	2.4134	0.3153	1	2.5643	2.5976	2.6125	2.5643	2.5643	0	2.2048	2.2541	0.0393	2.2048	2.2048	4	4	WF
02_022 sx	2.2257	2.2257	0.1611	0.3211	1.2765	5	2.3224	2.3224	0.2187	0.3840	1.2586	5	2.4813	0.2992	0.4537	0.4071	1.3522	5	2.5274	2.5817	2.5274	2.5274	0.3776	5	2.7389	2.7726	3.0213	2.7389	2.7389	5	2.0444	2.0855	0.0378	2.0444	2.0444	0	5	WF
02_023 dx	2.3949	2.3949	0.2066	0.3851	1.2466	5	2.5761	2.5761	0.3041	0.4542	1.3543	5	2.8066	0.3618	0.4898	0.4013	1.3887	5	2.8775	2.9320	2.8775	2.8775	0.4212	5	3.1233	3.1570	3.4426	3.1233	3.1233	5	2.5717	2.6201	0.0298	2.5717	2.5717	5	6	WF
02_023 sx	2.0079	2.0079	0.1534	0.3289	0.8072	2	2.0596	2.0596	0.1850	0.3648	0.9423	1	2.1558	0.2075	0.3678	0.2978	0.9133	0	2.1937	2.2266	2.1937	2.1937	0.2523	0	2.3351	2.3555	2.5915	2.3351	2.3351	0	2.1178	2.1523	0.0300	2.1178	2.1178	1	0	NF
02_024 dx	2.3531	2.3531	0.1952	0.3681	1.0140	5	2.5340	2.5340	0.3008	0.4511	1.2181	5	2.8085	0.4276	0.5439	0.5065	1.3608	5	2.9635	3.0077	2.9635	2.9635	0.5042	5	3.3140	3.3429	4.0300	3.3140	3.3140	5	2.3782	2.4182	0.0282	2.3782	2.3782	5	6	WF
02_024 sx	2.3417	2.3417	0.1625	0.3314	0.8637	5	2.4626	2.4626	0.2238	0.3779	1.0906	5	2.6674	0.3165	0.4600	0.3631	1.2644	5	2.7173	2.7728	2.7173	2.7173	0.3798	5	2.9283	2.9629	3.3497	2.9283	2.9283	5	2.0899	2.1376	0.0419	2.0899	2.0899	0	5	WF

02_025 dx	2.0497	2.0497	0.1584	0.3187	1.2542	5	2.1865	2.1865	0.2101	0.3719	1.2288	5	2.3607	0.2420	0.4050	0.3212	1.2046	1	2.3744	2.4291	2.3744	2.3744	0.2773	0	2.5160	2.5491	2.5175	2.5160	2.5160	0	2.2276	2.2771	0.0388	2.2276	2.2276	4	3	WF
02_025 sx	3.0699	3.0699	0.4028	0.5268	3.4934	5	3.3527	3.3527	0.5293	0.6042	2.0548	5	3.6379	0.6370	0.6645	0.5701	2.0768	5	3.7341	3.7920	3.7341	3.7341	0.7370	5	3.9923	4.0273	4.7381	3.9923	3.9923	5	2.7952	2.8396	0.0241	2.7952	2.7952	5	6	WF
02_026 dx	2.3409	2.3409	0.1194	0.2630	0.8151	2	2.4159	2.4159	0.1838	0.3313	1.0309	2	2.5559	0.2404	0.3883	0.3137	1.1051	1	2.5481	2.6026	2.5481	2.5481	0.3061	5	2.6908	2.7234	2.8655	2.6908	2.6908	5	2.3026	2.3542	0.0381	2.3026	2.3026	4	3	WF
02_026 sx	2.3618	2.3618	0.2224	0.3820	1.1968	5	2.5329	2.5329	0.2587	0.4007	1.1172	5	2.7439	0.3298	0.4496	0.3554	1.1606	5	2.7955	2.8497	2.7955	2.7955	0.3815	5	2.9987	3.0317	3.6019	2.9987	2.9987	5	2.1788	2.2202	0.0340	2.1788	2.1788	5	6	WF
02_027 dx	1.8509	1.8509	0.0457	0.1799	0.3801	0	1.8232	1.8232	0.1460	0.3048	0.9818	0	1.9521	0.1657	0.3300	0.2304	0.9785	0	2.0022	2.0433	2.0022	2.0022	0.2354	0	2.1971	2.2252	2.1491	2.1971	2.1971	0	1.9376	1.9826	0.0458	1.9376	1.9376	0	0	NF
02_027 sx	1.6368	1.6368	0.0840	0.2166	0.5826	0	1.7439	1.7439	0.1268	0.2755	0.8075	0	1.8821	0.1505	0.3059	0.2327	0.8203	0	1.9262	1.9621	1.9262	1.9262	0.1877	0	2.0943	2.1194	2.0696	2.0943	2.0943	0	1.9141	1.9568	0.0447	1.9141	1.9141	0	0	NF
02_028 dx	3.0698	3.0698	0.3292	0.4683	3.9541	5	3.3138	3.3138	0.4372	0.5421	2.2029	5	3.6588	0.5991	0.6281	0.5026	2.3221	5	3.7358	3.8383	3.7358	3.7358	0.7047	5	4.1128	4.1794	4.8266	4.1128	4.1128	5	2.6801	2.7553	0.0428	2.6801	2.6801	4	6	WF
02_028 sx	2.1418	2.1418	0.2007	0.3799	1.5432	5	2.2910	2.2910	0.2558	0.4322	1.4239	5	2.5010	0.3188	0.4839	0.4802	1.4454	5	2.5305	2.5964	2.5305	2.5305	0.3808	5	2.7219	2.7631	2.9417	2.7219	2.7219	5	2.1933	2.2445	0.0412	2.1933	2.1933	4	6	WF
02_029 dx	2.5188	2.5188	0.1339	0.2985	0.6784	4	2.5000	2.5000	0.1548	0.3271	0.8177	2	2.6568	0.4080	0.5505	0.5390	1.1879	5	2.7382	2.7760	2.7382	2.7382	0.4668	5	2.9603	2.9852	3.6414	2.9603	2.9603	5	2.0110	2.0353	0.0235	2.0110	2.0110	1	3	WF
02_029 sx	2.4683	2.4683	0.2296	0.3914	1.4779	5	2.6328	2.6328	0.2971	0.4399	1.3405	5	2.8421	0.3637	0.4847	0.3844	1.3563	5	2.8904	2.9443	2.8904	2.8904	0.4133	5	3.0798	3.1138	3.3505	3.0798	3.0798	5	2.4888	2.5361	0.0308	2.4888	2.4888	5	6	WF
02_030 dx	2.4878	2.4878	0.2632	0.4282	1.0689	5	2.7253	2.7253	0.3538	0.4947	1.1847	5	2.9769	0.4315	0.5444	0.4764	1.2450	5	3.0975	3.1387	3.0975	3.0975	0.5155	5	3.4056	3.4320	4.3545	3.4056	3.4056	5	2.1868	2.2313	0.0361	2.1868	2.1868	5	6	WF
02_030 sx	3.2230	3.2230	0.4703	0.5625	1.5738	5	3.5621	3.5621	0.6902	0.6857	1.6177	5	3.9223	0.8225	0.7493	0.6815	1.7241	5	4.0817	4.1457	4.0817	4.0817	0.9461	5	4.4954	4.5379	6.7495	4.4954	4.4954	5	2.9133	2.9847	0.0360	2.9133	2.9133	5	6	WF
02_031 dx	1.8015	1.8015	0.1401	0.3069	0.9819	3	1.9545	1.9545	0.1867	0.3507	1.0853	2	2.1483	0.2264	0.3872	0.3336	1.0824	0	2.1952	2.2465	2.1952	2.1952	0.2650	0	2.3830	2.4159	2.3888	2.3830	2.3830	0	2.0111	2.0497	0.0368	2.0111	2.0111	1	0	NF
02_031 sx	2.1177	2.1177	0.1513	0.3181	0.7950	4	2.1885	2.1885	0.2071	0.3721	0.9534	4	2.3398	0.2603	0.4189	0.3264	0.9981	2	2.3996	2.4401	2.3996	2.3996	0.2976	0	2.5819	2.6075	3.0307	2.5819	2.5819	1	2.2856	2.3319	0.0347	2.2856	2.2856	5	1	NF
02_032 dx	1.9499	1.9499	0.0762	0.2192	0.4993	0	1.9736	1.9736	0.1508	0.3151	0.8952	0	2.0661	0.1761	0.3451	0.3160	0.8973	0	2.0606	2.0935	2.0606	2.0606	0.2164	0	2.1360	2.1560	2.2072	2.1360	2.1360	0	1.9109	1.9412	0.0323	1.9109	1.9109	1	0	NF
02_032 sx	2.0073	2.0073	0.1434	0.3258	0.9700	3	2.0482	2.0482	0.2133	0.4014	1.1626	3	2.1568	0.2690	0.4521	0.4402	1.1961	4	2.1766	2.2117	2.1766	2.1766	0.3065	0	2.3496	2.3724	2.4543	2.3496	2.3496	0	2.0208	2.0509	0.0287	2.0208	2.0208	1	1	NF
02_033 dx	2.4687	2.4687	0.2757	0.4341	1.6470	5	2.7265	2.7265	0.3740	0.5050	1.6100	5	3.0340	0.4757	0.5716	0.5231	1.6991	5	3.1326	3.2041	3.1326	3.1326	0.5613	5	3.4516	3.4973	3.9693	3.4516	3.4516	5	2.5518	2.6099	0.0361	2.5518	2.5518	5	6	WF
02_033 sx	2.4214	2.4214	0.2395	0.3968	1.8490	5	2.6357	2.6357	0.2978	0.4428	1.4867	5	2.8909	0.3700	0.4954	0.4098	1.5348	5	2.9623	3.0221	2.9623	2.9623	0.4389	5	3.2001	3.2370	3.5234	3.2001	3.2001	5	2.3471	2.3959	0.0349	2.3471	2.3471	5	6	WF
02_035 dx	2.4713	2.4713	0.2607	0.4183	1.7665	5	2.6922	2.6922	0.3382	0.4777	1.4556	5	2.9580	0.4173	0.5309	0.4442	1.4835	5	3.0342	3.0980	3.0342	3.0342	0.4911	5	3.2930	3.3340	3.8422	3.2930	3.2930	5	2.4602	2.5198	0.0392	2.4602	2.4602	4	6	WF
02_035 sx	2.3701	2.3701	0.2691	0.4391	1.5438	5	2.5491	2.5491	0.3281	0.4707	1.4355	5	2.7786	0.4018	0.5224	0.4696	1.4886	5	2.8354	2.8945	2.8354	2.8354	0.4698	5	3.0549	3.0924	3.7229	3.0549	3.0549	5	2.3209	2.3679	0.0344	2.3209	2.3209	5	6	WF
02_036 dx	2.2466	2.2466	0.1375	0.3083	1.0750	5	2.3979	2.3979	0.2156	0.3707	1.2005	5	2.6115	0.2873	0.4234	0.3422	1.2791	5	2.6660	2.7249	2.6660	2.6660	0.3357	5	2.8962	2.9328	2.9154	2.8962	2.8962	5	2.0364	2.0741	0.0350	2.0364	2.0364	1	5	WF
02_036 sx	2.3592	2.3592	0.1809	0.3330	1.4614	5	2.5554	2.5554	0.2637	0.4035	1.3380	5	2.8004	0.3333	0.4566	0.3657	1.4100	5	2.8902	2.9429	2.8902	2.8902	0.3977	5	3.1657	3.1994	3.3469	3.1657	3.1657	5	2.4794	2.5335	0.0353	2.4794	2.4794	5	6	WF
02_037 dx	2.0294	2.0294	0.1312	0.2944	0.8018	4	2.1189	2.1189	0.1823	0.3466	1.0017	0	2.2564	0.2260	0.3875	0.3240	1.0266	0	2.2672	2.3131	2.2672	2.2672	0.2543	0	2.3785	2.4067	2.4359	2.3785	2.3785	0	2.2909	2.3425	0.0384	2.2909	2.2909	4	1	NF

02_03 7 sx	2.78 04	2.7 804	0.3 288	0.47 45	2.1 12 5	5	2.95 12	2.9 512	0.4 107	0.52 82	1.60 12	5	3.1 843	0.4 751	0.57 02	0.50 41	1.59 84	5	3.21 06	3.2 787	3.2 106	3.2 106	0.5 377	5	3.40 50	3.4 456	3.9 597	3.4 050	3.4 050	5	2.57 43	2.6 387	0.0 393	2.5 743	2.5 743	4	6	WF
02_03 8 dx	1.87 57	1.8 757	0.1 133	0.26 31	0.7 36 4	0	1.98 93	1.9 893	0.1 590	0.31 61	0.92 05	0	2.1 237	0.1 950	0.35 60	0.28 51	0.95 42	0	2.15 12	2.1 877	2.1 512	2.1 512	0.2 469	0	2.27 84	2.3 009	2.4 338	2.2 784	2.2 784	0	2.11 21	2.1 522	0.0 348	2.1 121	2.1 121	1	0	NF
02_03 8 sx	2.14 47	2.1 447	0.1 964	0.35 58	1.3 40 5	5	2.37 06	2.3 706	0.2 658	0.41 44	1.28 86	5	2.6 404	0.3 270	0.45 92	0.39 29	1.35 02	5	2.74 76	2.8 045	2.7 476	2.7 476	0.3 942	5	3.05 46	3.0 915	3.3 302	3.0 546	3.0 546	5	2.08 87	2.1 320	0.0 382	2.0 887	2.0 887	0	5	WF
02_03 9 dx	2.15 81	2.1 581	0.1 879	0.34 94	1.2 83 6	5	2.37 93	2.3 793	0.2 628	0.42 02	1.31 02	5	2.6 480	0.3 321	0.47 65	0.42 62	1.40 29	5	2.74 68	2.8 068	2.7 468	2.7 468	0.4 045	5	3.05 22	3.0 919	3.5 695	3.0 522	3.0 522	5	2.48 20	2.5 411	0.0 384	2.4 820	2.4 820	4	6	WF
02_03 9 sx	2.68 63	2.6 863	0.2 716	0.42 72	1.6 00 6	5	2.94 85	2.9 485	0.3 740	0.50 18	1.45 94	5	3.2 703	0.5 146	0.58 78	0.50 64	1.58 14	5	3.40 65	3.4 745	3.4 065	3.4 065	0.6 396	5	3.80 68	3.8 513	4.9 996	3.8 068	3.8 068	5	2.23 37	2.2 778	0.0 345	2.2 337	2.2 337	5	6	WF
02_04 0 dx	1.96 68	1.9 668	0.0 763	0.22 41	0.4 52 8	0	2.13 75	2.1 375	0.2 065	0.36 85	1.13 16	5	2.3 525	0.2 555	0.41 04	0.35 25	1.15 06	2	2.39 98	2.4 570	2.3 998	2.3 998	0.3 006	0	2.59 59	2.6 328	2.7 686	2.5 959	2.5 959	1	2.32 97	2.3 879	0.0 420	2.3 297	2.3 297	4	2	NF
02_04 0 sx	2.57 42	2.5 742	0.1 870	0.36 19	1.3 74 1	5	2.67 94	2.6 794	0.2 521	0.42 48	1.38 74	5	2.8 101	0.3 364	0.49 18	0.46 66	1.46 20	5	2.79 51	2.8 434	2.7 951	2.7 951	0.4 111	5	2.87 74	2.9 055	3.4 531	2.8 774	2.8 774	5	2.68 01	2.7 300	0.0 289	2.6 801	2.6 801	5	6	WF
02_04 1 dx	2.48 41	2.4 841	0.1 975	0.35 72	1.9 07 9	5	2.60 56	2.6 056	0.2 762	0.42 01	1.45 30	5	2.7 841	0.3 301	0.46 41	0.40 12	1.45 29	5	2.79 05	2.8 507	2.7 905	2.7 905	0.3 887	5	2.95 64	2.9 939	3.1 679	2.9 564	2.9 564	5	2.32 04	2.3 699	0.0 361	2.3 204	2.3 204	5	6	WF
02_04 1 sx	2.46 08	2.4 608	0.1 802	0.34 32	1.3 02 6	5	2.56 71	2.5 671	0.2 629	0.41 24	1.30 43	5	2.7 828	0.3 706	0.48 75	0.38 07	1.42 50	5	2.87 05	2.9 300	2.8 705	2.8 705	0.4 339	5	3.15 46	3.1 938	3.6 426	3.1 546	3.1 546	5	2.42 16	2.4 662	0.0 304	2.4 216	2.4 216	5	6	WF
02_04 2 dx	3.00 84	3.0 084	0.3 545	0.48 34	1.0 18 5	5	3.23 91	3.2 391	0.4 531	0.55 53	1.05 92	5	3.4 694	0.5 443	0.61 49	0.54 43	1.11 18	4	3.55 16	3.5 947	3.5 516	3.5 516	0.6 302	5	3.80 91	3.8 352	6.4 376	3.8 091	3.8 091	5	2.55 76	2.6 148	0.0 354	2.5 576	2.5 576	5	6	WF
02_04 2 sx	3.27 98	3.2 798	0.3 984	0.52 87	1.7 52 0	5	3.50 37	3.5 037	0.5 838	0.64 18	1.73 70	5	3.7 839	0.7 183	0.71 60	0.61 04	1.85 84	5	3.85 66	3.9 249	3.8 566	3.8 566	0.8 813	5	4.18 95	4.2 323	5.9 087	4.1 895	4.1 895	5	3.05 78	3.1 218	0.0 302	3.0 578	3.0 578	5	6	WF
02_04 3 dx	2.27 89	2.2 789	0.2 356	0.39 02	2.0 82 9	5	2.45 95	2.4 595	0.2 782	0.42 23	1.45 35	5	2.6 816	0.3 304	0.46 37	0.39 77	1.49 81	5	2.72 87	2.7 872	2.7 287	2.7 287	0.4 131	5	2.92 97	2.9 658	3.1 445	2.9 297	2.9 297	5	2.38 48	2.4 373	0.0 365	2.3 848	2.3 848	5	6	WF
02_04 3 sx	2.14 18	2.1 418	0.1 730	0.33 26	1.1 72 5	5	2.29 26	2.2 926	0.2 210	0.37 74	1.10 58	5	2.4 878	0.2 642	0.41 57	0.35 05	1.11 33	4	2.52 28	2.5 770	2.5 228	2.5 228	0.3 081	5	2.70 87	2.7 420	3.0 312	2.7 087	2.7 087	5	2.28 46	2.3 347	0.0 376	2.2 846	2.2 846	4	6	WF
02_04 4 dx	2.65 63	2.6 563	0.2 134	0.35 33	1.9 24 9	5	2.82 89	2.8 289	0.2 904	0.41 80	1.45 63	5	3.0 621	0.3 591	0.47 22	0.38 97	1.51 22	5	3.08 98	3.1 620	3.0 898	3.0 898	0.4 465	5	3.30 65	3.3 523	3.8 335	3.3 065	3.3 065	5	3.01 62	3.0 940	0.0 372	3.0 162	3.0 162	4	6	WF
02_04 4 sx	1.94 88	1.9 488	0.0 858	0.23 14	0.6 07 9	0	2.00 48	2.0 048	0.1 197	0.28 72	0.86 24	0	2.0 943	0.1 808	0.35 34	0.33 37	0.96 93	0	2.07 48	2.1 097	2.0 748	2.0 748	0.2 234	0	2.22 96	2.2 514	2.4 661	2.2 296	2.2 296	0	2.00 84	2.0 424	0.0 326	2.0 084	2.0 084	1	0	NF
02_04 5 dx	2.78 81	2.7 881	0.2 428	0.41 83	2.0 43 6	5	2.87 54	2.8 754	0.3 178	0.47 67	1.63 36	5	3.0 264	0.3 839	0.52 34	0.50 30	1.66 90	5	3.01 58	3.0 841	3.0 158	3.0 158	0.5 429	5	3.32 97	3.3 736	3.8 782	3.3 297	3.3 297	5	2.31 25	2.3 607	0.0 354	2.3 125	2.3 125	5	6	WF
02_04 5 sx	3.04 20	3.0 420	0.3 981	0.51 68	2.3 22 2	5	3.24 01	3.2 401	0.5 071	0.58 80	1.62 56	5	3.4 856	0.6 048	0.64 88	0.58 75	1.70 02	5	3.55 63	3.6 159	3.5 563	3.5 563	0.6 993	5	3.82 82	3.8 660	4.9 868	3.8 282	3.8 282	5	2.29 68	2.3 439	0.0 351	2.2 968	2.2 968	5	6	WF
02_04 6 dx	2.71 02	2.7 102	0.3 022	0.46 21	2.8 20 4	5	2.89 58	2.8 958	0.3 548	0.49 62	1.73 18	5	3.1 656	0.4 401	0.54 26	0.45 12	1.76 01	5	3.18 92	3.2 741	3.1 892	3.1 892	0.4 955	5	3.42 54	3.4 758	3.5 049	3.4 254	3.4 254	5	2.43 82	2.4 954	0.0 383	2.4 382	2.4 382	4	6	WF
02_04 6 sx	1.96 15	1.9 615	0.0 813	0.23 34	0.4 89 1	0	2.05 91	2.0 591	0.1 570	0.31 42	0.90 74	0	2.2 178	0.2 189	0.37 20	0.32 06	1.01 13	0	2.25 93	2.3 036	2.2 593	2.2 593	0.2 599	0	2.42 84	2.4 565	2.4 596	2.4 284	2.4 284	0	2.10 29	2.1 429	0.0 351	2.1 029	2.1 029	1	0	NF
02_04 7 dx	1.87 66	1.8 766	0.1 153	0.26 68	0.7 16 8	0	1.97 41	1.9 741	0.1 630	0.32 20	1.00 21	0	2.1 008	0.2 016	0.36 82	0.31 62	1.06 37	0	2.10 23	2.1 442	2.1 023	2.1 023	0.2 641	0	2.24 55	2.2 718	2.2 780	2.2 455	2.2 455	0	1.94 85	1.9 856	0.0 376	1.9 485	1.9 485	0	0	NF
02_04 7 sx	2.17 17	2.1 717	0.1 644	0.32 66	1.3 94 2	5	2.32 57	2.3 257	0.2 329	0.38 66	1.30 05	5	2.5 288	0.2 799	0.42 45	0.35 47	1.30 01	5	2.57 03	2.6 279	2.5 703	2.5 703	0.3 343	5	2.75 79	2.7 936	2.8 058	2.7 579	2.7 579	5	2.04 89	2.0 939	0.0 411	2.0 489	2.0 489	0	5	WF
02_04 8 dx	1.95 81	1.9 581	0.1 269	0.28 54	0.9 07 7	3	2.06 92	2.0 692	0.1 865	0.34 64	1.07 11	2	2.2 398	0.2 310	0.39 08	0.34 58	1.09 70	1	2.25 87	2.3 112	2.2 587	2.2 587	0.2 725	0	2.41 86	2.4 512	2.4 387	2.4 186	2.4 186	0	2.19 38	2.2 493	0.0 444	2.1 938	2.1 938	4	1	NF
02_04 8 sx	2.05 70	2.0 570	0.1 783	0.33 91	1.1 12 0	5	2.20 93	2.2 093	0.2 212	0.37 85	1.07 80	5	2.3 966	0.2 644	0.41 52	0.36 23	1.09 87	4	2.43 79	2.4 877	2.4 379	2.4 379	0.3 074	2	2.62 50	2.6 570	2.8 287	2.6 250	2.6 250	5	2.25 30	2.2 985	0.0 350	2.2 530	2.2 530	5	5	WF

02_049 dx	1.9708	1.9708	0.1294	0.2911	0.9677	3	2.0779	2.0779	0.1951	0.3600	1.1432	3	2.2506	0.2340	0.3993	0.3548	1.1498	2	2.2738	2.3282	2.2738	2.2738	0.2907	0	2.4398	2.4743	2.5288	2.4398	2.4398	0	1.9974	2.0468	0.0472	1.9974	1.9974	0	0	NF
02_049 sx	2.0311	2.0311	0.1429	0.2997	1.0443	5	2.1498	2.1498	0.1931	0.3469	1.0835	4	2.3226	0.2243	0.3769	0.3086	1.0663	0	2.3311	2.3871	2.3311	2.3311	0.2573	0	2.4669	2.5010	2.5369	2.4669	2.4669	0	2.1985	2.2561	0.0458	2.1985	2.1985	4	2	NF
02_050 dx	2.2542	2.2542	0.1663	0.3244	1.1409	5	2.4049	2.4049	0.2334	0.3846	1.1684	5	2.6017	0.2953	0.4374	0.3684	1.2277	5	2.6434	2.6986	2.6434	2.6434	0.3565	5	2.8477	2.8819	3.0603	2.8477	2.8477	5	2.1637	2.2144	0.0417	2.1637	2.1637	1	5	WF
02_050 sx	2.0178	2.0178	0.1618	0.3257	1.1873	5	2.1862	2.1862	0.2131	0.3779	1.1774	5	2.3967	0.2633	0.4223	0.3545	1.2002	5	2.4469	2.5026	2.4469	2.4469	0.3112	5	2.6589	2.6946	2.7115	2.6589	2.6589	4	2.1845	2.2336	0.0398	2.1845	2.1845	4	6	WF
02_051 dx	1.9158	1.9158	0.1538	0.3166	1.2282	3	2.0125	2.0125	0.1847	0.3481	1.1169	1	2.1418	0.2174	0.3829	0.3390	1.1189	1	2.1287	2.1759	2.1287	2.1287	0.2495	0	2.2328	2.2608	2.1990	2.2328	2.2328	0	1.9280	1.9688	0.0421	1.9280	1.9280	0	0	NF
02_051 sx	2.5394	2.5394	0.2846	0.4423	2.6026	5	2.8186	2.8186	0.3857	0.5124	1.8270	5	3.1485	0.4730	0.5657	0.4474	1.8531	5	3.2500	3.3265	3.2500	3.2500	0.5575	5	3.5759	3.6245	3.8586	3.5759	3.5759	5	2.4932	2.5522	0.0380	2.4932	2.4932	4	6	WF
02_052 dx	2.5764	2.5764	0.2109	0.3784	2.1494	5	2.6712	2.6712	0.2790	0.4266	1.4968	5	2.8399	0.3317	0.4612	0.3971	1.4491	5	2.8138	2.8841	2.8138	2.8138	0.3687	5	2.9465	2.9885	2.9826	2.9465	2.9465	5	2.7082	2.7777	0.0391	2.7082	2.7082	4	6	WF
02_052 sx	2.6619	2.6619	0.2328	0.4025	2.3762	5	2.7606	2.7606	0.2860	0.4472	1.6059	5	2.9477	0.3695	0.4996	0.4396	1.6131	5	2.9376	3.0106	2.9376	2.9376	0.4397	5	3.1133	3.1583	3.2141	3.1133	3.1133	5	2.8446	2.9211	0.0398	2.8446	2.8446	4	6	WF
02_053 dx	2.4137	2.4137	0.2096	0.3773	1.3395	5	2.6068	2.6068	0.2748	0.4266	1.3637	5	2.8491	0.3502	0.4792	0.4049	1.4427	5	2.8996	2.9670	2.8996	2.8996	0.4186	5	3.1455	3.1868	3.4199	3.1455	3.1455	5	2.0229	2.0708	0.0448	2.0229	2.0229	0	5	WF
02_053 sx	2.8002	2.8002	0.2256	0.3980	1.9489	5	2.8869	2.8869	0.2876	0.4437	1.5232	5	3.0726	0.3583	0.4887	0.4254	1.5338	5	3.0754	3.1496	3.0754	3.0754	0.4425	5	3.2847	3.3297	3.7626	3.2847	3.2847	5	2.6811	2.7511	0.0399	2.6811	2.6811	4	6	WF
02_054 dx	2.5958	2.5958	0.2365	0.4064	1.2589	5	2.7174	2.7174	0.3242	0.4813	1.2435	5	2.8901	0.3977	0.5342	0.5000	1.2764	5	2.9685	3.0064	2.9685	2.9685	0.4736	5	3.2050	3.2288	4.0086	3.2050	3.2050	5	2.3043	2.3399	0.0266	2.3043	2.3043	5	6	WF
02_054 sx	2.1779	2.1779	0.1485	0.3120	1.0425	5	2.3319	2.3319	0.2233	0.3837	1.1709	5	2.5469	0.2921	0.4417	0.3610	1.2143	5	2.6268	2.6763	2.6268	2.6268	0.3503	5	2.8738	2.9052	3.1299	2.8738	2.8738	5	2.3371	2.3845	0.0342	2.3371	2.3371	5	6	WF
02_055 dx	2.3274	2.3274	0.2096	0.3675	1.2001	5	2.5347	2.5347	0.2879	0.4329	1.2042	5	2.7411	0.3634	0.4901	0.4190	1.2481	5	2.8431	2.8751	2.8431	2.8431	0.4260	5	3.0828	3.1034	3.5707	3.0828	3.0828	5	2.3844	2.4213	0.0260	2.3844	2.3844	5	6	WF
02_055 sx	2.3136	2.3136	0.1731	0.3478	1.1114	5	2.5767	2.5767	0.3669	0.5082	1.4473	5	2.8819	0.4507	0.5599	0.5317	1.4751	5	3.0322	3.0773	3.0322	3.0322	0.5283	5	3.3689	3.3981	3.9122	3.3689	3.3689	5	2.4490	2.4853	0.0244	2.4490	2.4490	5	6	WF
02_056 dx	1.9667	1.9667	0.1561	0.3177	1.2323	3	2.1724	2.1724	0.2124	0.3712	1.2341	5	2.4210	0.2666	0.4160	0.3399	1.2653	4	2.4893	2.5530	2.4893	2.4893	0.3188	5	2.7390	2.7803	2.7157	2.7390	2.7390	4	2.2654	2.3188	0.0405	2.2654	2.2654	4	5	WF
02_056 sx	1.9124	1.9124	0.1703	0.3447	1.3100	3	2.0977	2.0977	0.2249	0.4000	1.2470	3	2.3248	0.2808	0.4504	0.4255	1.2851	4	2.3929	2.4483	2.3929	2.3929	0.3345	1	2.6307	2.6667	2.7662	2.6307	2.6307	5	2.0747	2.1218	0.0420	2.0747	2.0747	0	2	NF
02_057 dx	2.4357	2.4357	0.2488	0.3969	2.2928	5	2.6987	2.6987	0.3216	0.4484	1.5950	5	3.0152	0.3993	0.5010	0.3979	1.6138	5	3.0991	3.1781	3.0991	3.0991	0.4732	5	3.4017	3.4520	3.6822	3.4017	3.4017	5	2.5474	2.6124	0.0403	2.5474	2.5474	4	6	WF
02_057 sx	2.3063	2.3063	0.1154	0.2791	0.6453	2	2.2946	2.2946	0.1537	0.3151	0.9033	2	2.4551	0.3038	0.4590	0.4031	1.1371	5	2.5190	2.5671	2.5190	2.5190	0.3512	5	2.7339	2.7643	3.2409	2.7339	2.7339	5	2.2196	2.2676	0.0379	2.2196	2.2196	4	4	WF
02_058 dx	1.9501	1.9501	0.1381	0.2979	0.9216	3	2.0744	2.0744	0.1767	0.3401	1.0517	0	2.2370	0.2222	0.3830	0.3291	1.0828	0	2.2583	2.3067	2.2583	2.2583	0.2687	0	2.4209	2.4506	2.4978	2.4209	2.4209	0	2.0822	2.1243	0.0375	2.0822	2.0822	0	0	NF
02_058 sx	2.4343	2.4343	0.1858	0.3464	0.9739	5	2.5497	2.5497	0.2662	0.4138	1.0678	5	2.7278	0.3131	0.4524	0.3881	1.0855	4	2.7505	2.8040	2.7505	2.7505	0.3613	5	2.9248	2.9584	3.7305	2.9248	2.9248	5	2.4021	2.4514	0.0340	2.4021	2.4021	5	6	WF
02_059 dx	2.5681	2.5681	0.2448	0.3933	1.6818	5	2.7548	2.7548	0.3148	0.4516	1.3796	5	2.9765	0.3963	0.5079	0.4386	1.4815	5	3.0406	3.0936	3.0406	3.0406	0.4769	5	3.2818	3.3144	3.8001	3.2818	3.2818	5	2.3557	2.3940	0.0275	2.3557	2.3557	5	6	WF
02_059 sx	2.2325	2.2325	0.2025	0.3803	1.2688	5	2.3055	2.3055	0.2418	0.4169	1.1616	5	2.4220	0.3056	0.4625	0.4333	1.2018	5	2.4195	2.4610	2.4195	2.4195	0.3466	1	2.5027	2.5266	2.7936	2.5027	2.5027	1	2.1615	2.2040	0.0353	2.1615	2.1615	1	3	WF
02_060 dx	2.3231	2.3231	0.1817	0.3579	1.2147	5	2.3956	2.3956	0.2343	0.3995	1.2274	5	2.5401	0.2785	0.4331	0.3835	1.2038	5	2.5231	2.5822	2.5231	2.5231	0.3320	5	2.6356	2.6705	2.8647	2.6356	2.6356	5	2.1778	2.2265	0.0397	2.1778	2.1778	4	6	WF

02_060 sx	2.0257	2.0257	0.1437	0.2921	1.1934	5	2.2023	2.2023	0.2055	0.3503	1.1703	4	2.4291	0.2516	0.3945	0.3175	1.2208	2	2.4822	2.5435	2.4822	2.4822	0.3045	4	2.7119	2.7513	2.8158	2.7119	2.7119	5	1.9720	2.0169	0.0442	1.9720	1.9720	0	3	WF
02_061 dx	2.1770	2.1770	0.1977	0.3557	1.3201	5	2.3615	2.3615	0.2637	0.4077	1.2167	5	2.5991	0.3137	0.4440	0.3672	1.2367	5	2.6581	2.7218	2.6581	2.6581	0.3638	5	2.8940	2.9356	3.1568	2.8940	2.8940	5	2.1805	2.2310	0.0410	2.1805	2.1805	4	6	WF
02_061 sx	2.5356	2.5356	0.2595	0.4073	1.8889	5	2.7930	2.7930	0.3422	0.4672	1.5610	5	3.0910	0.4426	0.5319	0.4380	1.6611	5	3.1925	3.2592	3.1925	3.1925	0.5284	5	3.5021	3.5445	4.0902	3.5021	3.5021	5	2.3566	2.4120	0.0392	2.3566	2.3566	4	6	WF
02_062 dx	2.3052	2.3052	0.1554	0.3153	0.9653	5	2.4353	2.4353	0.2292	0.3773	1.1081	5	2.5870	0.2790	0.4228	0.3640	1.1281	5	2.6193	2.6581	2.6193	2.6193	0.3398	5	2.7691	2.7933	3.1059	2.7691	2.7691	5	2.2018	2.2340	0.0261	2.2018	2.2018	5	6	WF
02_062 sx	2.5711	2.5711	0.2436	0.3929	1.8336	5	2.7559	2.7559	0.3139	0.4472	1.4588	5	2.9808	0.3811	0.4958	0.4038	1.4874	5	3.0191	3.0802	3.0191	3.0191	0.4457	5	3.2098	3.2465	3.7046	3.2098	3.2098	5	2.4408	2.4913	0.0339	2.4408	2.4408	5	6	WF
02_063 dx	2.0043	2.0043	0.1430	0.3084	0.8263	2	2.1470	2.1470	0.2004	0.3537	1.1389	4	2.3496	0.2518	0.4053	0.3493	1.2023	2	2.3976	2.4498	2.3976	2.3976	0.3026	0	2.6088	2.6411	2.7630	2.6088	2.6088	1	2.2856	2.3393	0.0400	2.2856	2.2856	4	1	NF
02_063 sx	2.1427	2.1427	0.0838	0.2344	0.5033	2	2.1768	2.1768	0.1966	0.3475	1.0159	3	2.3548	0.2361	0.3865	0.3183	1.0525	0	2.3902	2.4385	2.3902	2.3902	0.2776	0	2.5656	2.5957	2.7142	2.5656	2.5656	0	2.1899	2.2361	0.0374	2.1899	2.1899	4	1	NF
02_064 dx	1.9955	1.9955	0.1684	0.3306	1.0973	3	2.1718	2.1718	0.2183	0.3723	1.1261	5	2.3738	0.2670	0.4166	0.3295	1.1835	3	2.4433	2.4881	2.4433	2.4433	0.3137	5	2.6536	2.6830	2.8102	2.6536	2.6536	5	2.2756	2.3206	0.0340	2.2756	2.2756	5	5	WF
02_064 sx	2.6110	2.6110	0.2379	0.3893	1.8819	5	2.7765	2.7765	0.3049	0.4467	1.4964	5	2.9861	0.3784	0.5059	0.4360	1.5644	5	3.0064	3.0704	3.0064	3.0064	0.4458	5	3.1959	3.2364	3.5595	3.1959	3.1959	5	2.4152	2.4779	0.0424	2.4152	2.4152	4	6	WF
02_065 dx	2.7631	2.7631	0.2907	0.4188	2.1908	5	2.9847	2.9847	0.3496	0.4613	1.6154	5	3.2702	0.4492	0.5305	0.4269	1.7219	5	3.3091	3.3918	3.3091	3.3091	0.5455	5	3.5528	3.6032	4.3385	3.5528	3.5528	5	2.9194	3.0128	0.0464	2.9194	2.9194	4	6	WF
02_065 sx	2.3253	2.3253	0.1957	0.3422	1.4346	5	2.4668	2.4668	0.2330	0.3785	1.1772	5	2.6486	0.2827	0.4236	0.3358	1.2117	4	2.6660	2.7202	2.6660	2.6660	0.3268	5	2.8120	2.8449	2.9053	2.8120	2.8120	5	2.8986	2.9705	0.0365	2.8986	2.8986	5	6	WF
02_066 dx	2.0111	2.0111	0.1362	0.2900	1.0459	3	2.1101	2.1101	0.1628	0.3108	0.9481	0	2.2576	0.2026	0.3458	0.2710	0.9568	0	2.2645	2.3156	2.2645	2.2645	0.2354	0	2.3977	2.4281	2.3572	2.3977	2.3977	0	2.2098	2.2612	0.0408	2.2098	2.2098	4	1	NF
02_066 sx	2.0773	2.0773	0.1243	0.2957	0.9814	5	2.1865	2.1865	0.2197	0.3819	1.2431	5	2.3684	0.2686	0.4227	0.3777	1.2602	4	2.4083	2.4565	2.4083	2.4083	0.3166	1	2.5816	2.6114	2.6807	2.5816	2.5816	0	1.9935	2.0331	0.0383	1.9935	1.9935	0	3	WF
02_067 dx	2.0122	2.0122	0.1156	0.2653	0.5687	0	2.0835	2.0835	0.1604	0.3129	0.8237	0	2.1715	0.1975	0.3561	0.3061	0.8898	0	2.1828	2.2130	2.1828	2.1828	0.2338	0	2.3492	2.3685	2.5195	2.3492	2.3492	0	2.3646	2.4070	0.0302	2.3646	2.3646	5	1	NF
02_067 sx	2.3512	2.3512	0.1805	0.3237	1.3911	5	2.4895	2.4895	0.2310	0.3736	1.1841	5	2.6607	0.2922	0.4268	0.3474	1.2252	5	2.6951	2.7419	2.6951	2.6951	0.3524	5	2.8659	2.8941	3.2056	2.8659	2.8659	5	2.0576	2.0919	0.0314	2.0576	2.0576	1	5	WF
02_068 dx	1.9401	1.9401	0.1426	0.2914	0.8684	3	2.0892	2.0892	0.1804	0.3290	0.9395	0	2.2692	0.2242	0.3752	0.2829	0.9982	0	2.3079	2.3553	2.3079	2.3079	0.2649	0	2.4777	2.5067	2.7040	2.4777	2.4777	0	2.0721	2.1199	0.0426	2.0721	2.0721	0	0	NF
02_068 sx	2.1555	2.1555	0.1454	0.3161	1.2605	5	2.2503	2.2503	0.2172	0.3772	1.2519	5	2.4101	0.2734	0.4248	0.3746	1.2829	5	2.4191	2.4737	2.4191	2.4191	0.3152	1	2.5526	2.5852	2.5683	2.5526	2.5526	0	2.1642	2.2083	0.0365	2.1642	2.1642	0	3	WF
02_069 dx	2.2963	2.2963	0.1068	0.2739	0.8146	2	2.2471	2.2471	0.1274	0.3009	0.9754	2	2.3267	0.1770	0.3500	0.2664	1.0209	0	2.2997	2.3540	2.2997	2.2997	0.2520	0	2.4347	2.4677	2.5663	2.4347	2.4347	0	1.9313	1.9714	0.0413	1.9313	1.9313	0	0	NF
02_069 sx	2.3084	2.3084	0.1711	0.3265	1.5877	5	2.4020	2.4020	0.2386	0.3866	1.3346	5	2.5713	0.2838	0.4281	0.3609	1.3322	5	2.5893	2.6437	2.5893	2.5893	0.3285	5	2.7509	2.7832	2.7895	2.7509	2.7509	5	2.1934	2.2408	0.0382	2.1934	2.1934	4	6	WF
02_070 dx	2.6959	2.6959	0.2214	0.3864	1.7909	5	2.8273	2.8273	0.3044	0.4535	1.5656	5	3.0259	0.4025	0.5164	0.4264	1.6429	5	3.0509	3.1186	3.0509	3.0509	0.4718	5	3.2554	3.2959	3.4948	3.2554	3.2554	5	2.4471	2.5057	0.0389	2.4471	2.4471	4	6	WF
02_070 sx	2.0213	2.0213	0.1201	0.2992	0.6453	3	2.1569	2.1569	0.2285	0.3901	1.1483	5	2.3945	0.2833	0.4381	0.3400	1.2120	4	2.5135	2.5535	2.5135	2.5135	0.3491	5	2.8026	2.8290	3.0781	2.8026	2.8026	5	2.0388	2.0748	0.0335	2.0388	2.0388	1	4	WF
02_071 dx	1.6663	1.6663	0.0988	0.2474	0.7647	0	1.8079	1.8079	0.1502	0.3079	0.9836	0	1.9795	0.1835	0.3441	0.2885	1.0088	0	2.0347	2.0742	2.0347	2.0347	0.2200	0	2.2226	2.2484	2.1586	2.2226	2.2226	0	1.9205	1.9521	0.0331	1.9205	1.9205	1	0	NF
02_071 sx	2.6287	2.6287	0.2931	0.4434	1.7660	5	2.8467	2.8467	0.3610	0.4895	1.3891	5	3.0852	0.4448	0.5432	0.4647	1.4664	5	3.1864	3.2318	3.1864	3.1864	0.5443	5	3.4569	3.4850	4.1059	3.4569	3.4569	5	2.4375	2.4810	0.0294	2.4375	2.4375	5	6	WF

02_072 dx	1.8740	1.8740	0.1519	0.3207	0.9508	3	2.0085	2.0085	0.1952	0.3585	1.0103	2	2.1809	0.2289	0.3860	0.3270	0.9978	0	2.2293	2.2729	2.2293	2.2293	0.2627	0	2.4088	2.4365	2.5871	2.4088	2.4088	0	2.0140	2.0557	0.0395	2.0140	2.0140	0	0	NF
02_072 sx	1.9716	1.9716	0.1343	0.3020	1.0136	3	2.1379	2.1379	0.1997	0.3668	1.1557	5	2.3653	0.2656	0.4236	0.3698	1.2360	4	2.4406	2.4967	2.4406	2.4406	0.3269	5	2.7145	2.7515	2.8375	2.7145	2.7145	5	2.0968	2.1435	0.0409	2.0968	2.0968	0	4	WF
02_073 dx	2.4586	2.4586	0.2241	0.3827	1.1401	5	2.6224	2.6224	0.2698	0.4125	1.1586	5	2.8051	0.3167	0.4500	0.3899	1.1946	5	2.8207	2.8747	2.8207	2.8207	0.3812	5	2.9517	2.9837	3.4467	2.9517	2.9517	5	2.2846	2.3318	0.0354	2.2846	2.2846	5	6	WF
02_073 sx	2.0973	2.0973	0.1464	0.2924	1.1211	5	2.2035	2.2035	0.1937	0.3501	1.1078	4	2.3394	0.2415	0.4000	0.3498	1.1446	2	2.3406	2.3841	2.3406	2.3406	0.2894	0	2.4299	2.4554	2.6134	2.4299	2.4299	0	2.2065	2.2506	0.0353	2.2065	2.2065	5	2	NF
02_074 dx	2.3740	2.3740	0.2312	0.3907	1.3051	5	2.5998	2.5998	0.3105	0.4541	1.2866	5	2.8538	0.3754	0.5019	0.4210	1.3056	5	2.9264	2.9856	2.9264	2.9264	0.4350	5	3.1671	3.2044	3.7649	3.1671	3.1671	5	2.5642	2.6226	0.0360	2.5642	2.5642	5	6	WF
02_074 sx	1.9913	1.9913	0.1684	0.3283	0.8498	2	2.1813	2.1813	0.2253	0.3895	1.0099	4	2.3791	0.2867	0.4436	0.3942	1.0646	3	2.4722	2.5072	2.4722	2.4722	0.3464	5	2.7049	2.7283	3.0658	2.7049	2.7049	5	2.2368	2.2734	0.0287	2.2368	2.2368	5	4	WF
02_075 dx	1.9860	1.9860	0.1131	0.2799	1.0859	1	2.0494	2.0494	0.1537	0.3274	1.1197	1	2.1981	0.2161	0.3791	0.3404	1.1835	1	2.2131	2.2707	2.2131	2.2131	0.2524	0	2.3974	2.4351	2.3611	2.3974	2.3974	0	2.0681	2.1175	0.0442	2.0681	2.0681	0	0	NF
02_075 sx	1.9038	1.9038	0.1464	0.3013	1.2238	3	2.0148	2.0148	0.1836	0.3418	1.1705	1	2.1769	0.2270	0.3845	0.3390	1.1880	0	2.1925	2.2468	2.1925	2.1925	0.2690	0	2.3418	2.3759	2.3227	2.3418	2.3418	0	1.9585	2.0000	0.0414	1.9585	1.9585	0	0	NF
02_076 dx	1.7143	1.7143	0.1047	0.2508	0.7652	0	1.7652	1.7652	0.1198	0.2746	0.8224	0	1.8649	0.1681	0.3302	0.2466	0.9174	0	1.8907	1.9210	1.8907	1.8907	0.1917	0	2.0240	2.0430	1.9733	2.0240	2.0240	0	1.8490	1.8815	0.0368	1.8490	1.8490	1	0	NF
02_076 sx	2.0073	2.0073	0.0842	0.2277	0.5952	0	2.0918	2.0918	0.1622	0.3209	0.9661	0	2.2400	0.2157	0.3747	0.3120	1.0562	0	2.2699	2.3158	2.2699	2.2699	0.2587	0	2.4179	2.4464	2.3691	2.4179	2.4179	0	1.8955	1.9301	0.0372	1.8955	1.8955	0	0	NF
02_077 dx	1.9351	1.9351	0.1520	0.3232	1.0308	3	2.0756	2.0756	0.1884	0.3592	1.0724	3	2.2442	0.2378	0.3997	0.3386	1.0841	0	2.2946	2.3356	2.2946	2.2946	0.2740	0	2.4688	2.4946	2.5419	2.4688	2.4688	0	2.0931	2.1403	0.0414	2.0931	2.0931	0	0	NF
02_077 sx	2.1339	2.1339	0.1995	0.3683	1.4913	5	2.3830	2.3830	0.2597	0.4128	1.2835	5	2.6449	0.3210	0.4569	0.3766	1.3036	5	2.7445	2.7965	2.7445	2.7445	0.3815	5	3.0188	3.0520	3.2261	3.0188	3.0188	5	2.2390	2.2864	0.0369	2.2390	2.2390	5	6	WF
02_078 dx	2.0706	2.0706	0.1505	0.3114	1.1003	5	2.1752	2.1752	0.1998	0.3591	1.1193	5	2.3214	0.2329	0.3891	0.3332	1.1215	1	2.3316	2.3764	2.3316	2.3316	0.2671	0	2.4521	2.4786	2.4267	2.4521	2.4521	0	2.1330	2.1714	0.0328	2.1330	2.1330	1	2	NF
02_078 sx	1.8477	1.8477	0.1184	0.2812	0.7767	0	1.9624	1.9624	0.1654	0.3308	0.9728	0	2.1244	0.2020	0.3669	0.3026	0.9877	0	2.1625	2.2081	2.1625	2.1625	0.2396	0	2.3259	2.3547	2.2962	2.3259	2.3259	0	2.1227	2.1638	0.0353	2.1227	2.1227	0	0	NF
02_079 dx	2.0513	2.0513	0.1345	0.2753	1.0411	4	2.2081	2.2081	0.1664	0.3041	0.9923	2	2.4062	0.2097	0.3521	0.2833	1.0662	1	2.4381	2.4965	2.4381	2.4381	0.2877	1	2.6245	2.6603	2.7836	2.6245	2.6245	5	1.9903	2.0387	0.0466	1.9903	1.9903	0	1	NF
02_079 sx	2.2583	2.2583	0.1585	0.3385	1.1752	5	2.3564	2.3564	0.2193	0.3980	1.2463	5	2.4940	0.2902	0.4533	0.4257	1.3123	5	2.5125	2.5580	2.5125	2.5125	0.3410	5	2.6509	2.6788	2.8015	2.6509	2.6509	5	2.1046	2.1446	0.0350	2.1046	2.1046	1	5	WF
02_080 dx	1.9365	1.9365	0.0913	0.2414	0.5639	0	2.0595	2.0595	0.1813	0.3392	0.9802	0	2.2362	0.2148	0.3740	0.3102	0.9905	0	2.2765	2.3216	2.2765	2.2765	0.2557	0	2.4444	2.4724	2.5870	2.4444	2.4444	0	2.0625	2.1084	0.0414	2.0625	2.0625	0	0	NF
02_080 sx	2.5867	2.5867	0.1789	0.3469	1.3398	5	2.7165	2.7165	0.2869	0.4334	1.4551	5	2.9282	0.4065	0.5159	0.4611	1.5800	5	2.9996	3.0628	2.9996	2.9996	0.5065	5	3.2913	3.3315	4.1936	3.2913	3.2913	5	2.4990	2.5529	0.0348	2.4990	2.4990	5	6	WF
02_081 dx	2.0878	2.0878	0.1600	0.3121	1.4494	5	2.2626	2.2626	0.2040	0.3521	1.1852	4	2.4994	0.2502	0.3923	0.2969	1.1993	2	2.5464	2.6164	2.5464	2.5464	0.3117	5	2.7797	2.8244	2.7426	2.7797	2.7797	5	2.2412	2.3013	0.0462	2.2412	2.2412	5	4	WF
02_081 sx	2.0232	2.0232	0.1492	0.3044	1.1164	5	2.1420	2.1420	0.1937	0.3513	1.1390	4	2.3086	0.2359	0.3928	0.3128	1.1642	1	2.3168	2.3720	2.3168	2.3168	0.2763	0	2.4459	2.4801	2.5847	2.4459	2.4459	0	2.1628	2.2160	0.0438	2.1628	2.1628	2	2	NF
02_082 dx	2.0399	2.0399	0.1649	0.3231	0.9425	5	2.1852	2.1852	0.2131	0.3707	1.0418	4	2.3597	0.2599	0.4119	0.3545	1.0725	2	2.4046	2.4492	2.4046	2.4046	0.3050	0	2.5713	2.6008	2.8615	2.5713	2.5713	1	2.1817	2.2243	0.0348	2.1817	2.1817	4	2	NF
02_082 sx	1.9280	1.9280	0.1445	0.2980	0.9840	3	2.1300	2.1300	0.1949	0.3523	1.0702	2	2.3701	0.2515	0.4063	0.3383	1.1312	1	2.4558	2.5094	2.4558	2.4558	0.3080	5	2.7160	2.7519	2.8583	2.7160	2.7160	5	2.2603	2.3077	0.0363	2.2603	2.2603	4	3	WF
02_083 dx	2.1749	2.1749	0.1710	0.3195	1.0900	5	2.3637	2.3637	0.2262	0.3740	1.1102	5	2.5658	0.2821	0.4229	0.3489	1.1353	5	2.6557	2.6913	2.6557	2.6557	0.3377	5	2.8813	2.9041	3.2035	2.8813	2.8813	5	2.2134	2.2454	0.0257	2.2134	2.2134	4	6	WF

02_083 sx	2.7230	2.7230	0.3332	0.4765	2.3349	5	3.0061	3.0061	0.4286	0.5406	1.6496	5	3.3064	0.5247	0.5982	0.5169	1.7144	5	3.4152	3.4731	3.4152	3.4152	0.6138	5	3.7017	3.7375	4.4480	3.7017	3.7017	5	2.5024	2.5448	0.0274	2.5024	2.5024	4	6	WF
02_084 dx	2.3855	2.3855	0.2188	0.3625	1.6645	5	2.5638	2.5638	0.2687	0.4064	1.2975	5	2.7778	0.3268	0.4529	0.3664	1.3546	5	2.8263	2.8812	2.8263	2.8263	0.3891	5	3.0299	3.0633	3.2364	3.0299	3.0299	5	2.2419	2.2875	0.0355	2.2419	2.2419	4	6	WF
02_084 sx	2.2014	2.2014	0.1170	0.2887	0.8450	3	2.2439	2.2439	0.2017	0.3661	1.1254	5	2.4066	0.2758	0.4256	0.3669	1.2189	5	2.4584	2.4993	2.4584	2.4584	0.3361	5	2.6415	2.6680	2.7829	2.6415	2.6415	5	2.1009	2.1361	0.0310	2.1009	2.1009	0	4	WF
02_085 dx	2.2703	2.2703	0.1523	0.3313	1.1584	5	2.4494	2.4494	0.2959	0.4586	1.4837	5	2.7015	0.3578	0.5064	0.4750	1.5076	5	2.7791	2.8387	2.7791	2.7791	0.4368	5	3.0324	3.0700	3.2531	3.0324	3.0324	5	1.9565	1.9933	0.0371	1.9565	1.9565	0	5	WF
02_085 sx	2.1124	2.1124	0.0880	0.2436	0.6062	1	2.1395	2.1395	0.1414	0.2986	0.8908	2	2.2887	0.1979	0.3516	0.2930	0.9736	0	2.3238	2.3774	2.3238	2.3238	0.2662	0	2.5176	2.5518	2.6151	2.5176	2.5176	0	1.9953	2.0386	0.0417	1.9953	1.9953	0	0	NF
02_086 dx	1.9363	1.9363	0.1096	0.2543	0.9018	1	2.0457	2.0457	0.1610	0.3196	1.0680	1	2.2091	0.2017	0.3665	0.3020	1.1021	0	2.2366	2.2890	2.2366	2.2366	0.2557	0	2.4236	2.4569	2.4771	2.4236	2.4236	0	2.2269	2.2815	0.0426	2.2269	2.2269	4	1	NF
02_086 sx	2.6748	2.6748	0.2890	0.4362	1.8435	5	2.8952	2.8952	0.3812	0.5005	1.4306	5	3.1572	0.4541	0.5498	0.4836	1.4406	5	3.2142	3.2805	3.2142	3.2142	0.5367	5	3.4487	3.4886	4.2008	3.4487	3.4487	5	2.4340	2.4885	0.0366	2.4340	2.4340	5	6	WF
02_087 dx	2.5925	2.5925	0.2079	0.3820	1.8655	5	2.6638	2.6638	0.2656	0.4280	1.4381	5	2.8769	0.3304	0.4689	0.3978	1.4372	5	2.8707	2.9478	2.8707	2.8707	0.3895	5	3.0424	3.0884	3.1471	3.0424	3.0424	5	1.9645	2.0052	0.0405	1.9645	1.9645	0	5	WF
02_087 sx	2.3661	2.3661	0.1831	0.3309	1.3375	5	2.4982	2.4982	0.2337	0.3839	1.2138	5	2.6888	0.3144	0.4552	0.3870	1.3083	5	2.7228	2.7829	2.7228	2.7228	0.3748	5	2.9373	2.9744	3.1134	2.9373	2.9373	5	2.3091	2.3656	0.0413	2.3091	2.3091	4	6	WF
02_088 dx	2.3876	2.3876	0.2170	0.3731	1.1594	5	2.5894	2.5894	0.2922	0.4371	1.1837	5	2.8409	0.3563	0.4857	0.4185	1.2417	5	2.9163	2.9779	2.9163	2.9163	0.4364	5	3.1854	3.2239	3.7107	3.1854	3.1854	5	2.2902	2.3425	0.0390	2.2902	2.2902	5	6	WF
02_088 sx	2.0465	2.0465	0.1529	0.3172	0.9374	5	2.2072	2.2072	0.2129	0.3726	1.1122	5	2.4136	0.2537	0.4076	0.3187	1.1320	2	2.4619	2.5167	2.4619	2.4619	0.2951	4	2.6699	2.7047	2.7454	2.6699	2.6699	5	2.4136	2.4717	0.0395	2.4136	2.4136	4	5	WF
02_089 dx	2.1451	2.1451	0.1563	0.3267	0.9060	5	2.3133	2.3133	0.2386	0.3996	1.2174	5	2.5195	0.2959	0.4495	0.4063	1.3018	5	2.5920	2.6377	2.5920	2.5920	0.3497	5	2.8385	2.8683	3.0274	2.8385	2.8385	5	2.1581	2.1987	0.0339	2.1581	2.1581	1	5	WF
02_090 dx	2.4271	2.4271	0.2489	0.4126	1.1996	5	2.6717	2.6717	0.3390	0.4837	1.2753	5	2.9518	0.4419	0.5519	0.4975	1.3706	5	3.0617	3.1171	3.0617	3.0617	0.5194	5	3.3589	3.3944	4.2394	3.3589	3.3589	5	2.6316	2.6886	0.0338	2.6316	2.6316	5	6	WF
02_090 sx	2.4581	2.4581	0.2321	0.3934	1.8516	5	2.6268	2.6268	0.3063	0.4560	1.5003	5	2.8402	0.3774	0.5099	0.4684	1.5363	5	2.8771	2.9372	2.8771	2.8771	0.4476	5	3.0784	3.1154	3.5996	3.0784	3.0784	5	2.1800	2.2236	0.0356	2.1800	2.1800	5	6	WF
02_091 dx	2.7192	2.7192	0.3561	0.4957	1.4889	5	3.0046	3.0046	0.4731	0.5715	1.4347	5	3.2928	0.5575	0.6214	0.5496	1.4583	5	3.4187	3.4671	3.4187	3.4187	0.6491	5	3.7127	3.7438	4.8719	3.7127	3.7127	5	2.5927	2.6450	0.0318	2.5927	2.5927	5	6	WF
02_091 sx	2.1742	2.1742	0.1454	0.3140	0.7703	4	2.2847	2.2847	0.2245	0.3798	1.1017	5	2.4828	0.2923	0.4399	0.3735	1.0990	4	2.5788	2.6208	2.5788	2.5788	0.3504	5	2.8310	2.8588	3.1399	2.8310	2.8310	5	2.1850	2.2170	0.0263	2.1850	2.1850	5	5	WF
02_092 dx	2.6687	2.6687	0.2838	0.4602	1.7196	5	2.8072	2.8072	0.3902	0.5382	1.5907	5	3.0095	0.4847	0.6004	0.6024	1.6446	5	3.0536	3.1105	3.0536	3.0536	0.5368	5	3.2711	3.3063	3.9265	3.2711	3.2711	5	2.7061	2.7600	0.0306	2.7061	2.7061	5	5	WF
02_092 sx	2.4800	2.4800	0.1474	0.2983	0.9209	5	2.5741	2.5741	0.2224	0.3662	1.0577	5	2.7390	0.3168	0.4443	0.3504	1.2060	5	2.7711	2.8215	2.7711	2.7711	0.3656	5	2.9516	2.9836	3.3997	2.9516	2.9516	5	2.4653	2.5090	0.0289	2.4653	2.4653	5	6	WF
02_093 dx	2.1090	2.1090	0.1097	0.2669	0.8353	2	2.2406	2.2406	0.1963	0.3504	1.1311	4	2.4350	0.2415	0.3953	0.3306	1.1724	2	2.4653	2.5232	2.4653	2.4653	0.3025	4	2.6431	2.6788	2.7194	2.6431	2.6431	4	2.1861	2.2317	0.0370	2.1861	2.1861	5	3	WF
02_093 sx	2.1523	2.1523	0.1544	0.3099	0.9766	5	2.2341	2.2341	0.2100	0.3690	1.1056	5	2.3901	0.2543	0.4083	0.3548	1.1480	3	2.4111	2.4641	2.4111	2.4111	0.3086	1	2.5805	2.6132	2.6819	2.5805	2.5805	0	2.3463	2.3981	0.0371	2.3463	2.3463	5	4	WF
02_094 dx	2.4782	2.4782	0.2607	0.4069	1.1814	5	2.6635	2.6635	0.3172	0.4511	1.0849	5	2.8762	0.4056	0.5168	0.4398	1.1790	5	2.9643	3.0097	2.9643	2.9643	0.4639	5	3.2083	3.2383	3.8543	3.2083	3.2083	5	2.3590	2.3974	0.0275	2.3590	2.3590	5	6	WF
02_094 sx	2.6570	2.6570	0.2911	0.4461	1.1495	5	2.8240	2.8240	0.3522	0.4976	1.1165	5	2.9872	0.4458	0.5584	0.5052	1.1977	5	3.0594	3.0885	3.0594	3.0594	0.5047	5	3.2351	3.2531	4.2548	3.2351	3.2351	5	2.4280	2.4544	0.0182	2.4280	2.4280	5	6	WF
02_095 dx	2.7653	2.7653	0.2667	0.4245	1.2741	5	2.9981	2.9981	0.3782	0.5043	1.3897	5	3.2643	0.4828	0.5701	0.4779	1.4971	5	3.3333	3.3981	3.3333	3.3333	0.5696	5	3.5971	3.6376	4.6331	3.5971	3.5971	5	2.3925	2.4479	0.0383	2.3925	2.3925	4	6	WF

02_095 sx	2.4001	2.4001	0.2001	0.3387	1.5135	5	2.6201	2.6201	0.2726	0.4009	1.2354	5	2.8923	0.3495	0.4619	0.3476	1.3267	5	3.0071	3.0631	3.0071	3.0071	0.4400	5	3.3519	3.3882	3.8349	3.3519	3.3519	5	2.3610	2.4095	0.0344	2.3610	2.3610	5	6	WF
02_096 dx	1.7881	1.7881	0.0698	0.2015	0.5395	0	1.8351	1.8351	0.1306	0.2805	0.8676	0	1.9504	0.1744	0.3324	0.2485	0.9569	0	1.9711	2.0088	1.9711	1.9711	0.2179	0	2.1352	2.1598	2.0775	2.1352	2.1352	0	2.0407	2.0844	0.0404	2.0407	2.0407	0	0	NF
02_096 sx	2.0438	2.0438	0.1587	0.3189	0.9427	5	2.2025	2.2025	0.2102	0.3696	1.1128	5	2.3946	0.2641	0.4169	0.3502	1.1841	5	2.4584	2.5044	2.4584	2.4584	0.3200	5	2.6693	2.6987	2.7963	2.6693	2.6693	5	2.1326	2.1785	0.0390	2.1326	2.1326	0	5	WF
02_097 dx	2.1266	2.1266	0.1703	0.3250	0.9502	5	2.2680	2.2680	0.2199	0.3760	0.9442	4	2.4732	0.2867	0.4306	0.3481	1.0186	4	2.5638	2.6018	2.5638	2.5638	0.3377	5	2.7982	2.8218	3.2591	2.7982	2.7982	5	2.4539	2.4984	0.0297	2.4539	2.4539	5	5	WF
02_097 sx	2.0689	2.0689	0.1714	0.3304	1.5201	5	2.1750	2.1750	0.2134	0.3742	1.2858	5	2.3242	0.2652	0.4205	0.3739	1.3096	4	2.3386	2.3862	2.3386	2.3386	0.3082	1	2.4721	2.5012	2.5402	2.4721	2.4721	0	1.9841	2.0222	0.0372	1.9841	1.9841	0	3	WF
02_098 dx	2.5160	2.5160	0.2477	0.4042	1.2178	5	2.6783	2.6783	0.3357	0.4795	1.2064	5	2.8682	0.3944	0.5248	0.4707	1.2168	5	2.9263	2.9703	2.9263	2.9263	0.4603	5	3.1329	3.1594	3.8558	3.1329	3.1329	5	2.3563	2.4035	0.0336	2.3563	2.3563	5	6	WF
02_098 sx	2.4842	2.4842	0.2719	0.4280	1.2616	5	2.8001	2.8001	0.3771	0.5040	1.3858	5	3.1423	0.4851	0.5735	0.4842	1.5168	5	3.3023	3.3644	3.3023	3.3023	0.5931	5	3.7090	3.7518	4.4706	3.7090	3.7090	5	2.5880	2.6292	0.0253	2.5880	2.5880	5	6	WF
02_099 dx	1.7340	1.7340	0.1024	0.2442	0.7357	0	1.8693	1.8693	0.1358	0.2835	0.8519	0	2.0326	0.1794	0.3328	0.2613	0.9314	0	2.0726	2.1160	2.0726	2.0726	0.2103	0	2.2274	2.2551	2.2131	2.2274	2.2274	0	1.9246	1.9656	0.0424	1.9246	1.9246	0	0	NF
02_099 sx	1.7873	1.7873	0.1097	0.2527	0.7055	0	1.8664	1.8664	0.1507	0.3051	0.8675	0	1.9863	0.1769	0.3378	0.2417	0.8925	0	2.0102	2.0452	2.0102	2.0102	0.2114	0	2.1525	2.1754	2.2267	2.1525	2.1525	0	1.8728	1.9077	0.0385	1.8728	1.8728	0	0	NF
02_100 dx	2.4840	2.4840	0.2147	0.3706	1.1341	5	2.6869	2.6869	0.2915	0.4297	1.2711	5	2.9107	0.3582	0.4809	0.3950	1.3516	5	2.9955	3.0436	2.9955	2.9955	0.4570	5	3.2638	3.2937	3.7484	3.2638	3.2638	5	2.5977	2.6419	0.0269	2.5977	2.5977	5	6	WF
02_100 sx	2.1161	2.1161	0.1634	0.3266	1.1280	5	2.2808	2.2808	0.2253	0.3865	1.2326	5	2.4528	0.2925	0.4435	0.3991	1.3186	5	2.4967	2.5370	2.4967	2.4967	0.3565	5	2.7879	2.8149	3.1180	2.7879	2.7879	5	2.0461	2.0815	0.0327	2.0461	2.0461	1	5	WF
02_101 dx	2.2232	2.2232	0.1966	0.3590	1.5228	5	2.4305	2.4305	0.2467	0.3953	1.2427	5	2.6811	0.3011	0.4342	0.3542	1.2594	5	2.7473	2.8111	2.7473	2.7473	0.3551	5	2.9909	3.0312	3.3338	2.9909	2.9909	5	2.3463	2.3977	0.0368	2.3463	2.3463	5	6	WF
02_101 sx	2.4785	2.4785	0.2009	0.3600	1.4859	5	2.6378	2.6378	0.2798	0.4278	1.3622	5	2.8626	0.3490	0.4814	0.3989	1.4122	5	2.9036	2.9704	2.9036	2.9036	0.4270	5	3.1434	3.1846	3.5161	3.1434	3.1434	5	2.4967	2.5597	0.0404	2.4967	2.4967	4	6	WF
02_102 dx	2.5227	2.5227	0.2011	0.3526	1.4906	5	2.6284	2.6284	0.2816	0.4236	1.3237	5	2.7558	0.3416	0.4756	0.3926	1.3812	5	2.7893	2.8224	2.7893	2.7893	0.3939	5	2.9393	2.9591	3.1585	2.9393	2.9393	5	2.2236	2.2484	0.0199	2.2236	2.2236	5	6	WF
02_102 sx	2.8327	2.8327	0.3241	0.4683	1.7606	5	3.0838	3.0838	0.4791	0.5683	1.4634	5	3.3656	0.6053	0.6392	0.5767	1.5135	5	3.5216	3.5617	3.5216	3.5216	0.7193	5	3.8662	3.8914	4.7376	3.8662	3.8662	5	2.5923	2.6254	0.0203	2.5923	2.5923	5	6	WF
02_103 dx	1.9954	1.9954	0.1174	0.2533	1.0361	1	2.1174	2.1174	0.1545	0.2979	1.0402	0	2.2888	0.1952	0.3463	0.2889	1.0800	0	2.2808	2.3424	2.2808	2.2808	0.2384	0	2.3931	2.4310	2.4061	2.3931	2.3931	0	2.1191	2.1756	0.0481	2.1191	2.1191	0	0	NF
02_103 sx	1.9842	1.9842	0.1274	0.2775	1.2906	2	2.1373	2.1373	0.1680	0.3184	1.1599	3	2.3535	0.1999	0.3471	0.2588	1.1062	0	2.3615	2.4335	2.3615	2.3615	0.2326	0	2.5256	2.5699	2.5944	2.5256	2.5256	0	2.0179	2.0786	0.0563	2.0179	2.0179	0	0	NF
02_104 dx	2.4320	2.4320	0.2888	0.4508	1.3204	5	2.6977	2.6977	0.3758	0.5089	1.2419	5	2.9801	0.4464	0.5511	0.4936	1.2804	5	3.0994	3.1509	3.0994	3.0994	0.5114	5	3.3980	3.4314	4.2679	3.3980	3.3980	5	2.7654	2.8305	0.0356	2.7654	2.7654	5	6	WF
02_104 sx	3.0767	3.0767	0.3354	0.4606	2.8780	5	3.2995	3.2995	0.4502	0.5396	1.7953	5	3.5890	0.5801	0.6182	0.5314	1.8866	5	3.6462	3.7312	3.6462	3.6462	0.7115	5	3.9872	4.0405	5.1414	3.9872	3.9872	5	2.5368	2.6031	0.0414	2.5368	2.5368	4	6	WF
02_105 dx	1.8677	1.8677	0.1206	0.2676	1.1704	0	1.9796	1.9796	0.1575	0.3104	1.0571	0	2.1502	0.1941	0.3498	0.2964	1.0663	0	2.1832	2.2367	2.1832	2.1832	0.2474	0	2.3742	2.4093	2.2921	2.3742	2.3742	0	1.9470	1.9913	0.0447	1.9470	1.9470	0	0	NF
02_105 sx	2.1535	2.1535	0.1727	0.3324	1.5958	5	2.3148	2.3148	0.2247	0.3848	1.2992	5	2.5373	0.2750	0.4312	0.3684	1.3019	5	2.5468	2.6218	2.5468	2.5468	0.3239	5	2.7204	2.7685	2.8105	2.7204	2.7204	5	2.1736	2.2397	0.0533	2.1736	2.1736	4	6	WF
02_106 dx	2.2367	2.2367	0.1554	0.3178	0.6919	5	2.3580	2.3580	0.2273	0.3851	0.9400	4	2.5448	0.2874	0.4357	0.3576	1.0018	4	2.5948	2.6445	2.5948	2.5948	0.3465	5	2.7958	2.8264	3.3365	2.7958	2.7958	5	2.1871	2.2356	0.0393	2.1871	2.1871	4	5	WF
02_106 sx	2.4343	2.4343	0.2143	0.3932	1.5456	5	2.5709	2.5709	0.3602	0.5140	1.5098	5	2.7365	0.4154	0.5512	0.5368	1.4683	5	2.7815	2.8238	2.7815	2.7815	0.4805	5	2.9464	2.9731	3.4316	2.9464	2.9464	5	2.3971	2.4428	0.0317	2.3971	2.3971	5	6	WF

02_107 dx	2.6282	2.6282	0.2932	0.4411	1.8901	5	2.8207	2.8207	0.3669	0.4951	1.4725	5	3.0608	0.4275	0.5343	0.4825	1.4856	5	3.0963	3.1644	3.0963	3.0963	0.4843	5	3.3104	3.3524	3.8261	3.3104	3.3104	5	2.4934	2.5495	0.0362	2.4934	2.4934	5	6	WF
02_107 sx	2.0001	2.0001	0.1008	0.2528	0.7021	0	2.1073	2.1073	0.1851	0.3398	1.0695	1	2.3145	0.2410	0.3910	0.3033	1.1325	1	2.3775	2.4305	2.3775	2.3775	0.2794	0	2.6050	2.6393	2.5919	2.6050	2.6050	0	2.1982	2.2465	0.0387	2.1982	2.1982	4	1	NF
02_108 dx	2.2075	2.2075	0.1438	0.3213	0.7979	4	2.2604	2.2604	0.2132	0.3970	1.0614	5	2.3697	0.2936	0.4640	0.4574	1.1349	4	2.4222	2.4643	2.4222	2.4222	0.3536	1	2.6264	2.6525	3.1325	2.6264	2.6264	5	2.1148	2.1563	0.0359	2.1148	2.1148	1	3	WF
02_108 sx	2.1281	2.1281	0.2089	0.3788	1.7661	5	2.3277	2.3277	0.2632	0.4215	1.3912	5	2.5606	0.3145	0.4596	0.3914	1.3705	5	2.6177	2.6758	2.6177	2.6177	0.3499	5	2.8284	2.8640	2.9426	2.8284	2.8284	5	2.3228	2.3711	0.0352	2.3228	2.3228	5	6	WF
02_109 dx	1.9142	1.9142	0.1016	0.2539	0.7814	0	2.0608	2.0608	0.1764	0.3291	1.0448	0	2.2638	0.2242	0.3713	0.2928	1.0798	0	2.3197	2.3704	2.3197	2.3197	0.2581	0	2.5182	2.5496	2.4920	2.5182	2.5182	0	2.1744	2.2228	0.0396	2.1744	2.1744	4	1	NF
02_109 sx	1.8210	1.8210	0.0802	0.2278	0.5211	0	1.9551	1.9551	0.1735	0.3447	1.0697	1	2.1508	0.2207	0.3849	0.3341	1.0762	0	2.2007	2.2524	2.2007	2.2007	0.2561	0	2.4004	2.4333	2.4181	2.4004	2.4004	0	1.9609	2.0046	0.0436	1.9609	1.9609	0	0	NF
02_110 dx	1.6936	1.6936	0.0662	0.2101	0.4723	0	1.8110	1.8110	0.1370	0.2880	0.8947	0	1.9615	0.1720	0.3313	0.2718	0.9461	0	2.0058	2.0434	2.0058	2.0058	0.2207	0	2.1638	2.1884	2.1333	2.1638	2.1638	0	1.8575	1.8941	0.0410	1.8575	1.8575	0	0	NF
02_110 sx	2.1750	2.1750	0.1720	0.3430	1.2561	5	2.2768	2.2768	0.2067	0.3745	1.1731	5	2.4251	0.2638	0.4209	0.3691	1.2037	5	2.4246	2.4790	2.4246	2.4246	0.2973	0	2.5584	2.5909	2.5910	2.5584	2.5584	0	2.6078	2.6794	0.0426	2.6078	2.6078	4	4	WF

Supporting information S1B Table: methods and features for Milano

	method1						method2						method3						method4						method5						method6							
Threshold	2.09146	2.09146	0.13497	0.30213	1.02272		2.22412	2.22412	0.19834	0.35557	1.10007		2.39383	0.25098	0.39995	0.33319	1.15431		2.44675	2.48716	2.44675	2.44675	0.29952		2.63743	2.67012	2.75343	2.63743	2.63743		2.13661	2.17559	0.03682	2.13661	2.13661			
N_PATIENT' (dx=right; sx=left)	M2MEA_i'	MAX_n'	VAR_P'	MAD0_p'	VAR_r'	number of S=1 occurrence	M2MEA_i'	MAX_n'	VAR_p'	MAD0_p'	MAD0_r'	number of S=1 occurrence	ROS1_i'	VAR_p'	MAD0_p'	MAD1_p'	MAD0_r'	number of S=1 occurrence	M2MEA_i'	ROS1_i'	MAX_n'	MAX_p'	VAR_p'	number of S=1 occurrence	M2MEA_i'	ROS1_i'	ROS2_i'	MAX_n'	MAX_p'	number of S=1 occurrence	M2MEA_i'	ROS1_i'	MIN_n'	MAX_n'	MAX_p'	number of S=1 occurrence	number of statements verified	MammoWave rule-of-thumb output
03_016 dx	2.3769	2.3769	0.2058	0.3700	0.7792	4	2.5450	2.5450	0.2645	0.4220	0.9598	4	2.7096	0.3210	0.4688	0.4148	1.0136	4	2.7728	2.8026	2.7728	2.7728	0.3854	5	2.9478	2.9660	3.8530	2.9478	2.9478	5	2.3697	2.4046	0.0248	2.3697	2.3697	5	4	WF
03_016 sx	2.2952	2.2952	0.2131	0.3827	1.4379	5	2.4783	2.4783	0.2663	0.4235	1.3188	5	2.7122	0.3250	0.4644	0.3967	1.3366	5	2.7703	2.8301	2.7703	2.7703	0.3769	5	2.9948	3.0323	3.2849	2.9948	2.9948	5	2.2587	2.3116	0.0404	2.2587	2.2587	4	6	WF
03_017 dx	2.3103	2.3103	0.1402	0.2893	0.9832	5	2.3593	2.3593	0.1711	0.3151	0.9357	2	2.4325	0.2112	0.3565	0.2723	0.9897	1	2.3780	2.4189	2.3780	2.3780	0.2357	4	2.3733	2.3961	2.4715	2.3733	2.3733	0	2.1174	2.1522	0.0301	2.1174	2.1174	5	3	WF
03_017 sx	2.0580	2.0580	0.1142	0.2703	0.7765	3	2.1031	2.1031	0.1544	0.3085	0.9354	2	2.2108	0.1900	0.3315	0.2488	0.9216	0	2.2490	2.2924	2.2490	2.2490	0.2331	0	2.4102	2.4374	2.3759	2.4102	2.4102	0	2.4241	2.4767	0.0356	2.4241	2.4241	5	1	NF
03_018 dx	2.2829	2.2829	0.1741	0.3283	1.2726	5	2.3978	2.3978	0.2223	0.3750	1.1916	5	2.5575	0.2880	0.4273	0.3576	1.2618	5	2.5704	2.6211	2.5704	2.5704	0.3344	5	2.7055	2.7361	2.7538	2.7055	2.7055	5	2.1534	2.1917	0.0322	2.1534	2.1534	5	6	WF
03_018 sx	2.5222	2.5222	0.1789	0.3428	1.3192	5	2.6472	2.6472	0.3507	0.4777	1.4569	5	2.9150	0.4148	0.5191	0.4345	1.5190	5	3.0139	3.0661	3.0139	3.0139	0.4660	5	3.2692	3.3011	3.6633	3.2692	3.2692	5	2.2362	2.2702	0.0268	2.2362	2.2362	5	6	WF
03_019 dx	2.2965	2.2965	0.1926	0.3502	0.8823	5	2.4868	2.4868	0.2737	0.4213	1.0665	5	2.7105	0.3431	0.4780	0.4065	1.1324	5	2.8133	2.8514	2.8133	2.8133	0.4018	5	3.0664	3.0906	3.5760	3.0664	3.0664	5	2.5906	2.6369	0.0283	2.5906	2.5906	5	6	WF
03_019 sx	2.1251	2.1251	0.1634	0.3239	1.0570	5	2.2625	2.2625	0.2156	0.3769	1.1239	5	2.4273	0.2645	0.4225	0.3409	1.1484	5	2.4877	2.5236	2.4877	2.4877	0.3044	5	2.6623	2.6864	2.8687	2.6623	2.6623	5	2.3561	2.3958	0.0284	2.3561	2.3561	5	6	WF
03_020 dx	1.9793	1.9793	0.1309	0.2829	0.8611	5	2.0955	2.0955	0.1772	0.3220	0.9865	3	2.2557	0.2179	0.3601	0.2763	1.0324	1	2.2897	2.3328	2.2897	2.2897	0.2710	5	2.4534	2.4803	2.4778	2.4534	2.4534	4	2.1001	2.1395	0.0346	2.1001	2.1001	2	4	WF
03_020 sx	2.1987	2.1987	0.1480	0.2882	1.2474	5	2.3441	2.3441	0.2218	0.3635	1.2097	5	2.5223	0.2723	0.4113	0.3272	1.2775	5	2.5549	2.6026	2.5549	2.5549	0.3246	5	2.7304	2.7597	2.8560	2.7304	2.7304	5	1.9664	2.0048	0.0382	1.9664	1.9664	0	5	WF
03_021 dx	2.2674	2.2674	0.1095	0.2622	0.7563	2	2.3280	2.3280	0.1516	0.3109	0.8990	2	2.4435	0.2189	0.3730	0.3212	0.9904	1	2.4797	2.5141	2.4797	2.4797	0.2926	5	2.6305	2.6508	2.7513	2.6305	2.6305	5	2.0044	2.0342	0.0288	2.0044	2.0044	1	2	NF
03_021 sx	1.8060	1.8060	0.0427	0.1634	0.2970	0	1.9260	1.9260	0.1561	0.3034	0.8863	0	2.1161	0.1973	0.3431	0.2682	0.9281	0	2.1952	2.2353	2.1952	2.1952	0.2391	0	2.4310	2.4573	2.4816	2.4310	2.4310	0	2.0029	2.0382	0.0340	2.0029	2.0029	1	0	NF
03_022 dx	2.2317	2.2317	0.1781	0.3319	1.2182	5	2.3886	2.3886	0.2246	0.3662	1.1535	5	2.5806	0.2713	0.4065	0.3158	1.1905	4	2.6180	2.6689	2.6180	2.6180	0.3169	5	2.7876	2.8183	2.8394	2.7876	2.7876	5	2.2775	2.3205	0.0325	2.2775	2.2775	5	6	WF
03_022 sx	2.1144	2.1144	0.1118	0.2783	0.7299	3	2.1632	2.1632	0.1800	0.3543	0.9921	4	2.3424	0.2853	0.4506	0.4242	1.1379	5	2.4263	2.4661	2.4263	2.4263	0.3380	5	2.6428	2.6689	2.8421	2.6428	2.6428	5	2.0228	2.0534	0.0290	2.0228	2.0228	1	3	WF
03_023 dx	1.8732	1.8732	0.1267	0.2799	0.8629	3	1.9647	1.9647	0.1724	0.3272	0.9965	0	2.0983	0.1975	0.3528	0.2623	0.9887	0	2.1247	2.1614	2.1247	2.1247	0.2248	0	2.2573	2.2800	2.2253	2.2573	2.2573	0	2.0675	2.1052	0.0341	2.0675	2.0675	1	0	NF
03_023 sx	1.8195	1.8195	0.1060	0.2469	0.7654	0	1.9528	1.9528	0.1340	0.2872	0.9014	0	2.1213	0.1848	0.3448	0.2738	1.0105	0	2.1585	2.2083	2.1585	2.1585	0.2340	0	2.3280	2.3593	2.2744	2.3280	2.3280	0	2.0131	2.0574	0.0418	2.0131	2.0131	0	0	NF
03_024 dx	2.0459	2.0459	0.1316	0.2929	1.0807	5	2.1320	2.1320	0.1903	0.3488	1.1013	5	2.2837	0.2175	0.3736	0.2941	1.0701	1	2.2940	2.3451	2.2940	2.2940	0.2623	4	2.4348	2.4661	2.3296	2.4348	2.4348	4	2.1022	2.1457	0.0380	2.1022	2.1022	4	5	WF
03_024 sx	2.0002	2.0002	0.1360	0.2890	0.9963	5	2.1129	2.1129	0.1830	0.3389	1.0538	5	2.2747	0.2138	0.3727	0.2997	1.0601	1	2.2796	2.3317	2.2796	2.2796	0.2489	4	2.3995	2.4306	2.3988	2.3995	2.3995	0	2.0032	2.0444	0.0394	2.0032	2.0032	0	3	WF
03_025 dx	2.1094	2.1094	0.1669	0.3178	1.2980	5	2.2293	2.2293	0.2077	0.3612	1.1461	5	2.3866	0.2520	0.4021	0.3422	1.1712	5	2.4119	2.4566	2.4119	2.4119	0.2965	5	2.5679	2.5950	2.5647	2.5679	2.5679	5	2.0506	2.0835	0.0304	2.0506	2.0506	1	5	WF
03_025 sx	2.0841	2.0841	0.1441	0.2991	1.1594	5	2.2448	2.2448	0.2211	0.3793	1.2231	5	2.4470	0.2785	0.4292	0.3568	1.2548	5	2.5079	2.5577	2.5079	2.5079	0.3365	5	2.7153	2.7466	2.7662	2.7153	2.7153	5	2.1058	2.1459	0.0350	2.1058	2.1058	5	6	WF
03_026 dx	2.0297	2.0297	0.0884	0.2169	0.6519	2	2.1172	2.1172	0.1468	0.2906	0.9126	2	2.2570	0.1802	0.3350	0.2652	0.9693	1	2.2715	2.3171	2.2715	2.2715	0.2415	4	2.4216	2.4495	2.3722	2.4216	2.4216	0	2.0179	2.0580	0.0379	2.0179	2.0179	0	1	NF
03_026 sx	1.8831	1.8831	0.1196	0.2870	0.8362	4	2.0058	2.0058	0.1818	0.3541	1.0922	5	2.1756	0.2251	0.3962	0.3571	1.1233	4	2.2101	2.2567	2.2101	2.2101	0.2777	1	2.3798	2.4088	2.4049	2.3798	2.3798	0	2.1072	2.1528	0.0395	2.1072	2.1072	4	3	WF
03_027 dx	1.9798	1.9798	0.1537	0.3233	0.9795	5	2.1694	2.1694	0.1968	0.3557	1.0564	5	2.3706	0.2389	0.3870	0.3178	1.0576	3	2.4385	2.4796	2.4385	2.4385	0.2795	5	2.6230	2.6487	2.7276	2.6230	2.6230	5	2.1852	2.2210	0.0294	2.1852	2.1852	5	6	WF
03_027 sx	2.0944	2.0944	0.1557	0.3053	1.4666	5	2.1927	2.1927	0.1918	0.3449	1.1852	5	2.3395	0.2333	0.3867	0.3263	1.1913	5	2.3264	2.3812	2.3264	2.3264	0.2712	5	2.4225	2.4546	2.4217	2.4225	2.4225	0	2.0370	2.0775	0.0375	2.0370	2.0370	0	4	WF
03_028 dx	1.9393	1.9393	0.1314	0.2805	1.1563	5	2.1047	2.1047	0.1905	0.3415	1.1453	5	2.3326	0.2474	0.3936	0.3247	1.2140	5	2.3950	2.4552	2.3950	2.3950	0.3026	5	2.6290	2.6679	2.5855	2.6290	2.6290	5	2.0396	2.0854	0.0422	2.0396	2.0396	0	5	WF
03_028 sx	1.9508	1.9508	0.1442	0.3025	1.1956	5	2.0589	2.0589	0.1777	0.3377	1.0795	5	2.2037	0.2105	0.3699	0.3155	1.0685	0	2.2223	2.2672	2.2223	2.2223	0.2544	0	2.3532	2.3805	2.2926	2.3532	2.3532	0	1.8513	1.8900	0.0435	1.8513	1.8513	0	2	NF
03_029 dx	1.9083	1.9083	0.0764	0.2352	0.6112	2	1.9339	1.9339	0.1220	0.2934	0.9347	0	2.0399	0.1903	0.3604	0.2854	1.0527	0	2.0466	2.0862	2.0466	2.0466	0.2256	0	2.1591	2.1837	2.1397	2.1591	2.1591	0	1.9974	2.0369	0.0382	1.9974	1.9974	0	0	NF

03_02 9 sx	2.20 72	2.2 072	0.1 832	0.33 53	1.7 002	5	2.30 56	2.3 056	0.2 207	0.37 51	1.27 80	5	2.4 489	0.2 612	0.41 59	0.34 39	1.28 91	5	2.45 32	2.5 020	2.4 532	2.4 532	0.3 262	5	2.58 30	2.6 125	2.7 159	2.5 830	2.5 830	5	2.16 83	2.2 217	0.0 437	2.1 683	2.1 683	4	6	WF
03_03 0 dx	2.64 14	2.6 414	0.1 515	0.32 07	1.1 119	5	2.64 48	2.6 448	0.1 916	0.36 28	1.15 96	5	2.7 544	0.2 648	0.42 41	0.36 61	1.23 77	5	2.78 15	2.8 360	2.7 815	2.7 815	0.3 848	5	3.02 94	3.0 647	3.5 524	3.0 294	3.0 294	5	2.34 69	2.3 998	0.0 378	2.3 469	2.3 469	4	6	WF
03_03 0 sx	2.14 17	2.1 417	0.0 916	0.25 11	0.8 121	2	2.13 41	2.1 341	0.1 198	0.28 99	0.95 93	2	2.2 188	0.1 600	0.33 47	0.30 37	1.00 81	0	2.17 37	2.2 249	2.1 737	2.1 737	0.2 176	0	2.24 66	2.2 774	2.2 202	2.2 466	2.2 466	0	1.90 75	1.9 518	0.0 465	1.9 075	1.9 075	0	0	NF
03_03 1 dx	2.12 26	2.1 226	0.0 872	0.23 84	0.6 622	2	2.13 66	2.1 366	0.1 326	0.29 81	0.94 54	2	2.2 648	0.2 501	0.40 47	0.34 49	1.16 60	5	2.30 16	2.3 419	2.3 016	2.3 016	0.2 855	5	2.43 29	2.4 575	2.5 621	2.4 329	2.4 329	1	2.16 92	2.2 062	0.0 306	2.1 692	2.1 692	5	3	WF
03_03 1 sx	1.94 08	1.9 408	0.1 208	0.27 99	1.0 077	5	2.01 08	2.0 108	0.1 631	0.32 74	1.06 85	3	2.1 312	0.2 061	0.37 16	0.28 86	1.08 76	0	2.13 55	2.1 807	2.1 355	2.1 355	0.2 453	0	2.25 51	2.2 824	2.3 198	2.2 551	2.2 551	0	1.89 74	1.9 396	0.0 449	1.8 974	1.8 974	0	1	NF
03_03 2 dx	1.86 49	1.8 649	0.1 176	0.26 32	0.8 094	1	1.98 54	1.9 854	0.1 506	0.30 15	0.91 27	0	2.1 477	0.1 828	0.33 67	0.26 32	0.93 51	0	2.17 64	2.2 243	2.1 764	2.1 764	0.2 305	0	2.33 98	2.3 699	2.2 667	2.3 398	2.3 398	0	2.09 67	2.1 423	0.0 399	2.0 967	2.0 967	0	0	NF
03_03 2 sx	1.84 22	1.8 422	0.1 007	0.23 60	0.7 757	0	1.95 55	1.9 555	0.1 456	0.28 90	0.91 35	0	2.1 193	0.1 770	0.32 63	0.25 28	0.94 20	0	2.14 96	2.1 963	2.1 496	2.1 496	0.2 239	0	2.32 04	2.3 500	2.2 424	2.3 204	2.3 204	0	1.96 90	2.0 063	0.0 371	1.9 690	1.9 690	0	0	NF
03_03 3 dx	1.95 74	1.9 574	0.0 848	0.22 89	0.5 980	2	1.92 52	1.9 252	0.1 896	0.34 35	1.02 15	3	2.1 014	0.2 197	0.37 31	0.31 24	1.03 19	0	2.17 02	2.2 077	2.1 702	2.1 702	0.2 497	0	2.36 92	2.3 943	2.4 210	2.3 692	2.3 692	0	2.14 03	2.1 773	0.0 314	2.1 403	2.1 403	5	1	NF
03_03 3 sx	1.87 54	1.8 754	0.1 303	0.28 50	0.8 815	3	2.01 43	2.0 143	0.1 706	0.33 18	1.00 13	2	2.1 848	0.2 218	0.38 28	0.27 22	1.07 13	1	2.22 69	2.2 739	2.2 269	2.2 269	0.2 610	0	2.39 92	2.4 303	2.4 592	2.3 992	2.3 992	0	2.01 06	2.0 493	0.0 369	2.0 106	2.0 106	0	0	NF
03_03 4 dx	2.05 14	2.0 514	0.1 278	0.27 32	0.9 856	5	2.15 38	2.1 538	0.1 834	0.33 19	1.07 86	4	2.2 917	0.2 226	0.37 26	0.29 27	1.11 28	3	2.31 67	2.3 542	2.3 167	2.3 167	0.2 548	4	2.43 54	2.4 581	2.4 301	2.4 354	2.4 354	3	2.21 08	2.2 460	0.0 283	2.2 108	2.2 108	5	5	WF
03_03 4 sx	2.51 88	2.5 188	0.2 518	0.40 98	1.7 417	5	2.72 48	2.7 248	0.3 337	0.46 96	1.45 56	5	2.9 506	0.4 010	0.51 69	0.44 44	1.50 81	5	3.02 01	3.0 706	3.0 201	3.0 201	0.4 645	5	3.23 55	3.2 675	3.5 990	3.2 355	3.2 355	5	2.49 42	2.5 400	0.0 297	2.4 942	2.4 942	5	6	WF
03_03 5 dx	2.38 74	2.3 874	0.2 016	0.36 69	1.4 333	5	2.53 21	2.5 321	0.2 657	0.42 20	1.36 29	5	2.7 301	0.3 315	0.47 39	0.41 97	1.39 84	5	2.73 61	2.8 025	2.7 361	2.7 361	0.3 801	5	2.89 07	2.9 313	3.1 141	2.8 907	2.8 907	5	2.34 89	2.4 063	0.0 407	2.3 489	2.3 489	5	6	WF
03_03 5 sx	2.05 08	2.0 508	0.1 741	0.33 65	1.2 430	5	2.23 59	2.2 359	0.2 190	0.37 70	1.19 41	5	2.4 510	0.2 681	0.41 98	0.35 51	1.21 69	5	2.51 54	2.5 654	2.5 154	2.5 154	0.3 151	5	2.72 20	2.7 547	2.8 260	2.7 220	2.7 220	5	2.44 54	2.5 129	0.0 446	2.4 454	2.4 454	5	6	WF
03_03 6 dx	1.89 82	1.8 982	0.0 906	0.24 69	0.6 880	2	1.94 31	1.9 431	0.1 505	0.31 90	1.01 58	1	2.0 367	0.1 915	0.36 61	0.33 77	1.04 78	0	2.06 60	2.1 069	2.0 660	2.0 660	0.2 607	0	2.24 52	2.2 708	2.2 767	2.2 452	2.2 452	0	1.98 54	2.0 280	0.0 414	1.9 854	1.9 854	0	0	NF
03_03 6 sx	1.86 75	1.8 675	0.1 207	0.26 65	0.8 184	1	1.97 80	1.9 780	0.1 669	0.32 38	0.98 24	0	2.1 377	0.2 135	0.37 28	0.32 03	1.05 02	0	2.18 64	2.2 278	2.1 864	2.1 864	0.2 617	0	2.36 42	2.3 910	2.4 404	2.3 642	2.3 642	0	1.98 83	2.0 241	0.0 349	1.9 883	1.9 883	1	0	NF
03_03 7 dx	1.97 05	1.9 705	0.1 496	0.30 18	0.8 833	5	2.09 80	2.0 980	0.1 829	0.33 42	0.94 63	4	2.2 460	0.2 176	0.36 82	0.27 41	0.98 66	1	2.28 44	2.3 199	2.2 844	2.2 844	0.2 531	4	2.42 38	2.4 453	2.5 197	2.4 238	2.4 238	1	2.22 49	2.2 608	0.0 285	2.2 249	2.2 249	5	3	WF
03_03 7 sx	1.87 95	1.8 795	0.0 550	0.19 42	0.3 823	2	1.91 11	1.9 111	0.1 467	0.30 03	0.91 92	0	2.0 779	0.1 686	0.32 14	0.22 05	0.85 53	0	2.13 00	2.1 691	2.1 300	2.1 300	0.1 993	0	2.29 94	2.3 238	2.3 289	2.2 994	2.2 994	0	2.17 17	2.2 158	0.0 362	2.1 717	2.1 717	5	1	NF
03_03 8 dx	2.24 95	2.2 495	0.1 021	0.26 75	0.6 946	2	1.97 25	1.9 725	0.1 183	0.29 44	0.84 89	0	2.0 722	0.2 016	0.36 50	0.27 57	0.98 38	0	2.11 72	2.1 586	2.1 172	2.1 172	0.2 394	0	2.29 43	2.3 210	2.2 452	2.2 943	2.2 943	0	1.88 33	1.9 221	0.0 421	1.8 833	1.8 833	0	0	NF
03_03 8 sx	2.06 54	2.0 654	0.1 267	0.29 19	0.9 343	5	2.18 09	2.1 809	0.1 935	0.35 16	1.10 48	5	2.3 816	0.2 481	0.39 66	0.33 37	1.15 25	5	2.42 64	2.4 842	2.4 264	2.4 264	0.2 875	5	2.63 74	2.6 751	2.6 696	2.6 374	2.6 374	5	1.96 80	2.0 044	0.0 363	1.9 680	1.9 680	1	5	WF
03_03 9 dx	1.81 17	1.8 117	0.0 760	0.22 01	0.5 916	0	1.86 55	1.8 655	0.1 260	0.28 29	0.88 51	0	1.9 803	0.1 744	0.33 67	0.26 13	0.95 62	0	2.00 37	2.0 395	2.0 037	2.0 037	0.2 154	0	2.13 30	2.1 548	2.0 880	2.1 330	2.1 330	0	1.93 32	1.9 643	0.0 323	1.9 332	1.9 332	1	0	NF
03_03 9 sx	1.80 99	1.8 099	0.1 264	0.28 28	0.9 396	3	1.93 17	1.9 317	0.1 723	0.33 53	1.02 30	2	2.0 929	0.2 154	0.37 90	0.34 15	1.05 16	1	2.13 19	2.1 749	2.1 319	2.1 319	0.2 560	0	2.30 05	2.3 272	2.3 465	2.3 005	2.3 005	0	2.01 61	2.0 549	0.0 369	2.0 161	2.0 161	0	0	NF
03_04 0 dx	1.78 91	1.7 891	0.1 186	0.27 85	0.8 867	3	1.88 34	1.8 834	0.1 576	0.32 14	0.99 85	0	2.0 404	0.2 255	0.38 36	0.26 34	1.13 99	2	2.10 45	2.1 442	2.1 045	2.1 045	0.2 607	0	2.29 02	2.3 160	2.2 545	2.2 902	2.2 902	0	1.95 36	1.9 901	0.0 369	1.9 536	1.9 536	0	0	NF
03_04 0 sx	2.16 24	2.1 624	0.1 729	0.32 69	1.3 711	5	2.33 38	2.3 338	0.2 093	0.35 08	1.13 06	5	2.5 478	0.2 517	0.38 15	0.27 92	1.11 13	3	2.60 49	2.6 574	2.6 049	2.6 049	0.3 017	5	2.81 52	2.8 474	2.7 651	2.8 152	2.8 152	5	1.96 65	1.9 952	0.0 288	1.9 665	1.9 665	1	5	WF
03_04 1 dx	2.33 37	2.3 337	0.2 079	0.36 79	2.2 790	5	2.55 11	2.5 511	0.2 843	0.43 39	1.61 97	5	2.8 399	0.3 615	0.49 36	0.41 40	1.64 84	5	2.90 54	2.9 861	2.9 054	2.9 054	0.4 371	5	3.20 11	3.2 535	3.3 083	3.2 011	3.2 011	5	1.96 09	2.0 097	0.0 483	1.9 609	1.9 609	0	5	WF
03_04 1 sx	1.95 49	1.9 549	0.1 513	0.31 13	1.1 416	5	2.14 67	2.1 467	0.2 070	0.36 44	1.15 63	5	2.3 789	0.2 648	0.41 45	0.33 98	1.18 86	5	2.47 19	2.5 212	2.4 719	2.4 719	0.3 233	5	2.74 49	2.7 772	2.9 634	2.7 449	2.7 449	5	2.08 51	2.1 259	0.0 362	2.0 851	2.0 851	1	5	WF
03_04 2 dx	1.58 70	1.5 870	0.1 129	0.26 92	0.8 875	1	1.71 65	1.7 165	0.1 356	0.29 78	0.89 78	0	1.8 948	0.1 619	0.32 52	0.27 61	0.89 19	0	1.96 22	1.9 998	1.9 622	1.9 622	0.1 903	0	2.15 56	2.1 803	2.0 637	2.1 556	2.1 556	0	1.77 83	1.8 099	0.0 390	1.7 783	1.7 783	0	0	NF
03_04 2 sx	1.82 90	1.8 290	0.0 722	0.22 65	0.5 196	0	1.93 95	1.9 395	0.1 680	0.33 41	1.03 36	2	2.0 979	0.2 182	0.38 16	0.33 75	1.07 48	1	2.13 91	2.1 808	2.1 391	2.1 391	0.2 531	0	2.29 90	2.3 253	2.3 316	2.2 990	2.2 990	0	2.05 17	2.0 899	0.0 350	2.0 517	2.0 517	1	0	NF
03_04 3 dx	2.17 23	2.1 723	0.1 590	0.30 47	0.9 344	5	2.31 43	2.3 143	0.2 115	0.35 88	1.07 37	5	2.4 760	0.2 568	0.40 26	0.31 99	1.13 35	4	2.52 35	2.5 612	2.5 235	2.5 235	0.3 109	5	2.69 05	2.7 144	2.9 707	2.6 905	2.6 905	5	2.19 72	2.2 312	0.0 276	2.1 972	2.1 972	5	6	WF
03_04 3 sx	1.90 75	1.9 075	0.1 310	0.28 21	0.9 747	5	2.05 37	2.0 537	0.1 867	0.34 00	1.04 93	5	2.2 395	0.2 284	0.37 99	0.31 55	1.04 30	2	2.30 26	2.3 452	2.3 026	2.3 026	0.2 709	5	2.50 10	2.5 288	2.6 269	2.5 010	2.5 010	5	2.15 53	2.1 946	0.0 329	2.1 553	2.1 553	5	5	WF
03_04 4 SX(dx)	2.37 36	2.3 736	0.2 257	0.37 45	1.9 589	5	2.58 82	2.5 882	0.2 854	0.42 37	1.44 93	5	2.8 330	0.3 514	0.47 36	0.40 02	1.48 40	5	2.90 36	2.9 598	2.9 036	2.9 036	0.4 275	5	3.13 62	3.1 707	3.3 148	3.1 362	3.1 362	5	2.24 93	2.2 899	0.0 315	2.2 493	2.2 493	5	6	WF
03_04 4 DX(sx)	2.38 21	2.3 821	0.1 519	0.31 99	1.0 879	5	2.49 69	2.4 969	0.2 431	0.39 28	1.25 81	5	2.6 573	0.2 987	0.44 26	0.38 37	1.28 71	5	2.66 30	2.7 163	2.6 630	2.6 630	0.3 706	5	2.79 01	2.8 215	3.0 960	2.7 901	2.7 901	5	2.22 14	2.2 659	0.0 352	2.2 214	2.2 214	5	6	WF
03_04 5 dx	2.00 21	2.0 021	0.1 851	0.35 38	1.4 645	5	2.16 44	2.1 644	0.2 198	0.38 72	1.25 34	5	2.3 638	0.2 659	0.42 47	0.37 84	1.22 25	5	2.41 65	2.4 651	2.4 165	2.4 165	0.3 072	5	2.61 13	2.6 408	2.7 303	2.6 113	2.6 113	5	2.27 59	2.3 227	0.0 354	2.2 759	2.2 759	5	6	WF
03_04 5 sx	2.08 53	2.0 853	0.1 612	0.31 22	0.9 384	5	2.28 30	2.2 830	0.2 196	0.36 96	1.04 89	5	2.5 138	0.2 800	0.42 45	0.34 86	1.13 85	5	2.59 31	2.6 451	2.5 931	2.5 931	0.3 401	5	2.83 72	2.8 716	3.1 219	2.8 372	2.8 372	5	2.36 17	2.4 059	0.0 314	2.3 617	2.3 617	5	6	WF

03_04 6 dx	2.1006	2.1006	0.1388	0.2909	1.2551	5	2.2236	2.2236	0.2036	0.3538	1.1909	5	2.3918	0.2564	0.4028	0.3254	1.2246	5	2.4216	2.4697	2.4216	2.4216	0.2986	5	2.5905	2.6201	2.5879	2.5905	2.5905	5	2.0842	2.1279	0.0388	2.0842	2.0842	0	5	WF
03_04 6 sx	1.8520	1.8520	0.0632	0.2086	0.4551	0	1.9152	1.9152	0.1498	0.3076	0.9390	0	2.0396	0.1782	0.3405	0.2784	0.9449	0	2.0580	2.0959	2.0580	2.0580	0.2212	0	2.1951	2.2188	2.1704	2.1951	2.1951	0	2.0598	2.0982	0.0350	2.0598	2.0598	1	0	NF
03_04 7 dx	2.3537	2.3537	0.2002	0.3510	1.3385	5	2.5732	2.5732	0.2721	0.4125	1.2673	5	2.8249	0.3454	0.4680	0.3814	1.3393	5	2.9212	2.9728	2.9212	2.9212	0.4180	5	3.1943	3.2270	3.6682	3.1943	3.1943	5	2.2481	2.2845	0.0283	2.2481	2.2481	5	6	WF
03_04 7 sx	2.7831	2.7831	0.2605	0.4351	1.2843	5	2.9102	2.9102	0.3370	0.4907	1.3362	5	3.0871	0.4275	0.5463	0.5129	1.4099	5	3.1281	3.1760	3.1281	3.1281	0.5230	5	3.3184	3.3472	4.2082	3.3184	3.3184	5	2.4223	2.4591	0.0252	2.4223	2.4223	5	6	WF
03_04 8 dx	1.7904	1.7904	0.0522	0.1951	0.3955	0	1.9310	1.9310	0.1586	0.3173	1.0134	0	2.1212	0.2121	0.3697	0.2928	1.0904	0	2.1909	2.2322	2.1909	2.1909	0.2509	0	2.4002	2.4267	2.3230	2.4002	2.4002	0	1.9218	1.9534	0.0332	1.9218	1.9218	1	0	NF
03_04 8 sx	1.8469	1.8469	0.0832	0.2378	0.6038	0	1.9584	1.9584	0.1688	0.3254	1.0059	0	2.1188	0.2055	0.3640	0.2941	1.0204	0	2.1629	2.2020	2.1629	2.1629	0.2431	0	2.3183	2.3422	2.3041	2.3183	2.3183	0	2.0257	2.0647	0.0366	2.0257	2.0257	0	0	NF
03_04 9 dx	2.3985	2.3985	0.1486	0.3090	1.0360	5	2.4626	2.4626	0.2483	0.3973	1.1653	5	2.6182	0.2913	0.4395	0.3904	1.1833	5	2.6442	2.6948	2.6442	2.6442	0.3696	5	2.8142	2.8451	3.4272	2.8142	2.8142	5	2.1682	2.2144	0.0381	2.1682	2.1682	4	6	WF
03_04 9 sx	2.1177	2.1177	0.1342	0.3086	0.7626	4	2.2599	2.2599	0.2580	0.4269	1.1938	5	2.4887	0.3025	0.4596	0.4305	1.2065	5	2.5633	2.6157	2.5633	2.5633	0.3410	5	2.8019	2.8363	3.0855	2.8019	2.8019	5	2.2473	2.2940	0.0361	2.2473	2.2473	5	5	WF
03_05 0 dx	1.7768	1.7768	0.1119	0.2597	0.6842	0	1.8799	1.8799	0.1552	0.3130	0.8639	0	2.0128	0.1897	0.3523	0.2722	0.9025	0	2.0522	2.0843	2.0522	2.0522	0.2179	0	2.1869	2.2068	2.3459	2.1869	2.1869	0	2.0935	2.1331	0.0350	2.0935	2.0935	1	0	NF
03_05 0 sx	2.5482	2.5482	0.2192	0.3789	1.3102	5	2.6580	2.6580	0.2701	0.4157	1.2088	5	2.8281	0.3302	0.4552	0.3502	1.2236	5	2.8102	2.8770	2.8102	2.8102	0.3666	5	2.9176	2.9569	3.2558	2.9176	2.9176	5	2.5080	2.5759	0.0431	2.5080	2.5080	4	6	WF
03_05 1 dx	1.8154	1.8154	0.1254	0.2777	0.8007	2	1.9557	1.9557	0.1501	0.3022	0.8660	0	2.1402	0.1885	0.3432	0.2850	0.9109	0	2.1993	2.2459	2.1993	2.1993	0.2438	0	2.4128	2.4438	2.5048	2.4128	2.4128	1	2.0920	2.1344	0.0374	2.0920	2.0920	0	0	NF
03_05 1 sx	1.7558	1.7558	0.0512	0.1949	0.3920	0	1.6983	1.6983	0.1394	0.3011	0.9511	0	1.8124	0.1582	0.3260	0.2400	0.9331	0	1.8149	1.8541	1.8149	1.8149	0.1779	0	1.9937	2.0205	1.9057	1.9937	1.9937	0	1.9685	2.0105	0.0415	1.9685	1.9685	0	0	NF
03_05 2 dx	2.0464	2.0464	0.1481	0.3012	1.0166	5	2.1796	2.1796	0.1974	0.3539	1.0743	5	2.3452	0.2379	0.3970	0.3323	1.1084	5	2.3668	2.4140	2.3668	2.3668	0.2777	5	2.5091	2.5375	2.6025	2.5091	2.5091	5	2.0239	2.0682	0.0414	2.0239	2.0239	4	6	WF
03_05 2 sx	2.2371	2.2371	0.1801	0.3416	1.4730	5	2.3732	2.3732	0.2743	0.4298	1.4001	5	2.5702	0.3217	0.4713	0.4146	1.4265	5	2.6219	2.6775	2.6219	2.6219	0.4015	5	2.8650	2.9003	3.0375	2.8650	2.8650	5	2.1149	2.1576	0.0369	2.1149	2.1149	4	6	WF
03_05 3 dx	1.9590	1.9590	0.1221	0.2813	0.8814	5	2.0210	2.0210	0.1706	0.3316	0.9816	2	2.1536	0.2128	0.3713	0.2791	0.9988	0	2.1823	2.2237	2.1823	2.1823	0.2483	0	2.3443	2.3704	2.4456	2.3443	2.3443	0	2.4288	2.4873	0.0393	2.4288	2.4288	4	2	NF
03_05 3 sx	1.9382	1.9382	0.1293	0.2835	0.9217	5	2.0454	2.0454	0.1650	0.3220	1.0077	2	2.2143	0.1917	0.3511	0.2635	1.0086	0	2.2446	2.2978	2.2446	2.2446	0.2466	1	2.4263	2.4595	2.4171	2.4263	2.4263	0	1.9648	2.0115	0.0461	1.9648	1.9648	0	1	NF
03_05 4 dx	3.0933	3.0933	0.3487	0.4779	3.3143	5	3.2938	3.2938	0.4397	0.5412	2.0076	5	3.5506	0.5755	0.6151	0.5101	2.1572	5	3.6003	3.6726	3.6003	3.6003	0.6620	5	3.8398	3.8836	4.3339	3.8398	3.8398	5	2.2366	2.2817	0.0352	2.2366	2.2366	5	6	WF
03_05 4 sx	1.8096	1.8096	0.1095	0.2694	0.7787	0	1.8822	1.8822	0.1446	0.3142	0.9206	0	1.9910	0.1713	0.3476	0.3227	0.9315	0	1.9877	2.0281	1.9877	1.9877	0.2112	0	2.1033	2.1285	2.0634	2.1033	2.1033	0	1.9525	1.9939	0.0416	1.9525	1.9525	0	0	NF
03_05 5 dx	2.3214	2.3214	0.1760	0.3420	1.2890	5	2.5136	2.5136	0.2710	0.4123	1.3439	5	2.7655	0.3436	0.4656	0.3768	1.4530	5	2.8358	2.8954	2.8358	2.8358	0.4013	5	3.0915	3.1282	3.2840	3.0915	3.0915	5	2.3635	2.4126	0.0348	2.3635	2.3635	5	6	WF
03_05 5 sx	2.3825	2.3825	0.1564	0.3399	0.9284	5	2.2608	2.2608	0.2557	0.4213	1.1289	5	2.4656	0.2918	0.4422	0.3898	1.1193	5	2.5305	2.5740	2.5305	2.5305	0.3275	5	2.7349	2.7620	3.1496	2.7349	2.7349	5	2.2480	2.3055	0.0441	2.2480	2.2480	4	6	WF
03_05 6 dx	2.1773	2.1773	0.1689	0.3294	1.0392	5	2.3118	2.3118	0.2260	0.3753	1.0939	5	2.4728	0.2675	0.4123	0.3392	1.1188	5	2.5338	2.5655	2.5338	2.5338	0.3118	5	2.7121	2.7318	2.8456	2.7121	2.7121	5	2.5328	2.5676	0.0222	2.5328	2.5328	5	6	WF
03_05 6 sx	2.1621	2.1621	0.2149	0.3787	1.2280	5	2.3554	2.3554	0.2778	0.4310	1.2094	5	2.5668	0.3282	0.4710	0.4224	1.2454	5	2.6446	2.6861	2.6446	2.6446	0.3775	5	2.8538	2.8795	3.0669	2.8538	2.8538	5	2.4407	2.4831	0.0286	2.4407	2.4407	5	6	WF
03_05 7 dx	2.3633	2.3633	0.1912	0.3352	1.3654	5	2.5390	2.5390	0.2674	0.3997	1.3138	5	2.7534	0.3223	0.4448	0.3441	1.3796	5	2.8002	2.8565	2.8002	2.8002	0.3757	5	2.9955	3.0304	3.1626	2.9955	2.9955	5	2.4851	2.5282	0.0283	2.4851	2.4851	5	6	WF
03_05 7 sx	1.9318	1.9318	0.1168	0.2711	0.7653	3	2.0089	2.0089	0.1608	0.3214	0.9647	2	2.1588	0.2002	0.3618	0.3110	1.0146	0	2.2158	2.2553	2.2158	2.2158	0.2489	0	2.4112	2.4363	2.3960	2.4112	2.4112	0	2.3054	2.3472	0.0310	2.3054	2.3054	5	1	NF
03_05 8 dx	2.0813	2.0813	0.1466	0.3040	0.8330	4	2.1655	2.1655	0.2071	0.3659	1.0364	5	2.2895	0.2494	0.4091	0.3680	1.0859	4	2.3222	2.3554	2.3222	2.3222	0.2967	5	2.4716	2.4926	2.7596	2.4716	2.4716	5	2.2001	2.2341	0.0275	2.2001	2.2001	5	5	WF
03_05 8 sx	2.2205	2.2205	0.1095	0.2840	0.8190	3	2.2064	2.2064	0.1510	0.3361	1.0472	4	2.2620	0.2108	0.3923	0.3649	1.1173	4	2.3161	2.3508	2.3161	2.3161	0.3216	5	2.5040	2.5257	2.6959	2.5040	2.5040	5	2.1857	2.2197	0.0279	2.1857	2.1857	5	4	WF
03_05 9 dx	2.1101	2.1101	0.1528	0.3296	0.7869	4	2.2354	2.2354	0.2473	0.4232	1.0486	5	2.4052	0.3413	0.5000	0.4632	1.1280	5	2.5081	2.5363	2.5081	2.5081	0.4132	5	2.7414	2.7595	3.1770	2.7414	2.7414	5	2.3885	2.4227	0.0240	2.3885	2.3885	5	5	WF
03_05 9 sx	2.5580	2.5580	0.2966	0.4379	1.2889	5	2.8275	2.8275	0.4120	0.5226	1.2269	5	3.1136	0.5053	0.5811	0.5201	1.2832	5	3.2503	3.2998	3.2503	3.2503	0.6097	5	3.5806	3.6139	4.5265	3.5806	3.5806	5	2.8524	2.9030	0.0266	2.8524	2.8524	5	6	WF
03_06 0 dx	2.1407	2.1407	0.1216	0.3009	0.8595	5	2.0710	2.0710	0.1438	0.3099	0.9924	2	2.2555	0.2222	0.3757	0.3068	1.1055	3	2.3113	2.3603	2.3113	2.3113	0.2804	5	2.5056	2.5368	2.5639	2.5056	2.5056	5	1.8545	1.8893	0.0391	1.8545	1.8545	0	4	WF
03_06 0 sx	2.0482	2.0482	0.1147	0.2687	0.8043	3	2.2220	2.2220	0.1877	0.3333	1.0413	5	2.4239	0.2733	0.4105	0.3227	1.2097	4	2.4998	2.5441	2.4998	2.4998	0.3222	5	2.7229	2.7509	2.9159	2.7229	2.7229	5	2.3826	2.4251	0.0298	2.3826	2.3826	5	5	WF
03_06 1 dx	1.6907	1.6907	0.0943	0.2340	0.6589	0	1.8249	1.8249	0.1636	0.3257	0.9885	0	2.0300	0.1918	0.3497	0.2731	0.9720	0	2.1055	2.1502	2.1055	2.1055	0.2230	0	2.3250	2.3542	2.2639	2.3250	2.3250	0	1.9499	1.9872	0.0378	1.9499	1.9499	0	0	NF
03_06 1 sx	2.0079	2.0079	0.0994	0.2438	0.7734	2	2.1587	2.1587	0.1655	0.3123	0.9806	2	2.3745	0.2335	0.3749	0.2981	1.0856	2	2.4327	2.4901	2.4327	2.4327	0.2773	5	2.6634	2.7005	2.6984	2.6634	2.6634	5	2.4260	2.4951	0.0462	2.4260	2.4260	4	3	WF
03_06 2 dx	2.3191	2.3191	0.1464	0.3271	1.0252	5	2.4284	2.4284	0.2253	0.3947	1.2025	5	2.5952	0.2840	0.4395	0.3867	1.2369	5	2.6063	2.6601	2.6063	2.6063	0.3538	5	2.7655	2.7983	2.9482	2.7655	2.7655	5	1.9609	2.0017	0.0407	1.9609	1.9609	0	5	WF
03_06 2 sx	2.8244	2.8244	0.2047	0.3733	2.0568	5	2.9071	2.9071	0.2621	0.4176	1.5026	5	3.0613	0.3566	0.4851	0.4231	1.5857	5	3.0394	3.1061	3.0394	3.0394	0.4294	5	3.1624	3.2021	3.4433	3.1624	3.1624	5	2.0976	2.1423	0.0391	2.0976	2.0976	1	5	WF

03_06 3 dx	2.1712	2.1712	0.0994	0.2625	0.6747	2	2.1956	2.1956	0.1641	0.3377	1.0024	3	2.2725	0.2795	0.4343	0.3957	1.1360	5	2.3646	2.4083	2.3646	2.3646	0.3293	5	2.6086	2.6367	2.8956	2.6086	2.6086	5	2.3535	2.4020	0.0346	2.3535	2.3535	5	4	WF
03_06 3 sx	2.9344	2.9344	0.3264	0.4676	2.8115	5	3.1582	3.1582	0.4060	0.5188	1.7711	5	3.4475	0.5138	0.5814	0.4854	1.8716	5	3.5066	3.5897	3.5066	3.5066	0.5996	5	3.7762	3.8276	4.2381	3.7762	3.7762	5	2.3931	2.4398	0.0324	2.3931	2.3931	5	6	WF
03_06 4 dx	1.9603	1.9603	0.0990	0.2588	0.5942	2	2.0028	2.0028	0.1962	0.3568	1.0495	3	2.1644	0.2350	0.3955	0.2851	1.0787	2	2.2183	2.2556	2.2183	2.2183	0.2888	1	2.3916	2.4151	2.5378	2.3916	2.3916	1	1.9314	1.9662	0.0360	1.9314	1.9314	1	0	NF
03_06 4 sx	1.7723	1.7723	0.0833	0.2336	0.6184	0	1.8311	1.8311	0.1267	0.2865	0.8727	0	1.9375	0.1599	0.3226	0.2835	0.8947	0	1.9479	1.9855	1.9479	1.9479	0.1924	0	2.0521	2.0754	2.0033	2.0521	2.0521	0	1.8576	1.8930	0.0397	1.8576	1.8576	0	0	NF
