# Supplementary material for: A multicentric, single arm, prospective, stratified clinical investigation to evaluate MammoWave’s ability in breast lesions detection
Source: PLoS One. 2023 Jul 14;18(7):e0288312. doi: 10.1371/journal.pone.0288312 (PMC10348515; doi:10.1371/journal.pone.0288312)
Supplement: S1 Appendix — (DOCX) [file pone.0288312.s002.docx]

**Appendix**

**MammoWave device and MammoWave analysis description**

MammoWave’s first prototype was developed and successfully tested on phantoms in 2015. Since then, the device has undergone optimization cycles until the current clinically ready prototype was constructed. The device’s image and configurations can be seen in Fig. 1. It comprises of two antennas, one acting as transmitter and the other as receiver of microwave signals. Both antennas always operate in air (without the need for any matching liquid or medium), at a frequency band of 1-9 GHz, and are positioned on the same vertical height. The antennas are connected to a 2-port vector network analyzer (Copper Mountain Technologies, Indianapolis, IN) and are contained by a cylindrical hub internally surrounded by microwave absorbers. This cylindrical hub includes a cup placed inside a hole, which permits the insertion of the patient’s whole breast in a prone position, as sketched in Fig. 1.

Both antennas rotate around azimuthally, collecting the signals in a multi-bistatic manner. Specifically, for each transmitting position, the receiver rotates with a step of 4.5°, measuring the signals at 80 receiving positions all around the breast. In total, 15 transmitting positions, divided into 5 triplet sections centered at 0°, 72°, 144°, 216°, and 288°, are used in the acquisition configuration (Fig. 1); in each section, the 3 transmitting positions are separated by 4.5° from each other. MammoWave acquisition time is approximately 10 minutes (per breast).

To process the received signals, we use our previously developed Huygens principle-based imaging algorithm [15, 16], which has demonstrated promising preliminary results [17-19]. This algorithm has the capability of reconstructing images of a target in a background medium by measuring only the field on the external surface of the object of study. This measured field is then back propagated inside the breast through the Green’s function [15, 16] to reconstruct the internal field. Finally, we construct the intensity image through incoherent summation of the contributions from all the transmitting positions and all the frequency points.

MammoWave acquisition is made just once, and then a set of five conductivity weighted microwave images is produced, using sigma values 0.3, 0.4, 0.5, 0.6 and 0.8 S/m. Referring to sigma=0.6 S/m, we obtained microwave images employing a rotation subtraction artefact removal of both 4.5° and 9° degrees, while for all the other sigma values we only employed a rotation subtraction artefact removal of 9°. Rotation subtraction artefact removal is performed through subtracting the received signals obtained from two adjacent transmitting positions (here 4.5° or 9° apart). This results in a total of six microwave images, i.e., *methods* for each breast; such *methods* have been selected via a feasibility study, following a procedure similar to what given in [19]. Specifically, we name:

*Method1:* sigma= 0.3 S/m; rotation subtraction artefact removal of 9°;

*Method2:* sigma= 0.4 S/m; rotation subtraction artefact removal of 9°;

*Method3:* sigma= 0.5 S/m; rotation subtraction artefact removal of 9°;

*Method4:* sigma= 0.6 S/m; rotation subtraction artefact removal of 9°;

*Method5:* sigma= 0.8 S/m; rotation subtraction artefact removal of 9°;

*Method6:* sigma= 0.6 S/m; rotation subtraction artefact removal of 4.5°.

Images obtained using the proposed apparatus are intensity maps, given in linear arbitrary units, representing the homogeneity of tissues’ dielectric properties. Images are maximum intensity projection coronal 2D maps of the entire breast volume. Images are divided into four quadrants corresponding to breast’s Upper-Outer (UO) quadrant; Upper-Inner (UI) quadrant; Lower-Outer (LO) quadrant; Lower-Inner (LI) quadrant. To allow inter and intra-subject comparison, all images are normalized to unitary average of the intensity.

For allowing a quantification of the non-homogenous behaviour of the microwave images, we introduce the following parameters, i.e., *features*:

MAX = Maximum value of the image;

MIN = Minimum value of the image;

MEA = Mean value of the image;

MED = Median value of the image;

VAR = Variance of the image;

MAD0 = Mean absolute deviation of the image;

MAD1 = Median absolute deviation of the image;

M2AVG = (MAX)/(MEA);

ROS1 = (MAX-MIN)/(MEA-MIN);

ROS2 = (MAX-MIN)/(MED-MIN).

For each *conductivity weighted* image, the previous features are calculated on the full domain of the image, where they are denoted with the subscript “*_i*”. In addition, for each *conductivity weighted* image, all the features listed above excluding ROS1, ROS2 are calculated on the peak region (a region which is centered in the maximum of the image, and it extends to MAX/√2), where they are denoted with the subscript “*_p*”; and on its complementary, where they are denoted with the subscript “*_c*”. The ratios between features calculated on the peak region and on its complementary are considered as added features, and they are denoted with the subscript “*_r*”.

Next, following the results of a feasibility study [19], we selected 5 features for each method, as follows:

*Method1*: M2MEA_i, MAX_n, VAR_p, MAD0_p, VAR_r;

*Method2*: M2MEA_i, MAX_n, VAR_p, MAD0_p, MAD0_r;

*Method3*: ROS1_i, VAR_p, MAD0_p, MAD1_p, MAD0_r;

*Method4:* M2MEA_i, ROS1_i, MAX_n, MAX_p, VAR_p;

*Method5*: M2MEA_i, ROS1_i, ROS2_i, MAX_n, MAX_p;

*Method6*: M2MEA_i, ROS1_i, MIN_n, MAX_n, MAX_p.

For each breast and for all selected *methods* and *features*, we introduce a binary score S defined as follows:


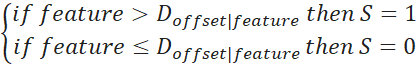


The threshold *D_offset|feature_* is calculated for each feature, using the median value obtained in each site after recruiting the first 15 subjects, all having breasts without any lesion.

The binary score S is then used for establishing an empirical *rule-of-thumb* allowing assessment of MammoWave images. Specifically, following a procedure similar to what is given in [19], the final assessment of MammoWave examination is performed as follows:

If at least 3 of the following 6 statements are verified, MammoWave’s final assessment is “Breast with finding (WF), i.e., with lesion (positive)”:

in *method1*: (number of S=1 occurrence) >= 5;

in *method2*: (number of S=1 occurrence) >= 5;

in *method3*: (number of S=1 occurrence) >= 3;

in *method4*: (number of S=1 occurrence) >= 3;

in *method5*: (number of S=1 occurrence) >= 3;

in *method6*: (number of S=1 occurrence) >= 2.

Conversely, if the previous statements are not verified, MammoWave’s final assessment is “Breast with no finding (NF), i.e., with no lesion (negative)”.

Note that, in case MammoWave’s final assessment is WF, the location of the peak is considered to represent the lesion’s location in terms of quadrant.
